# Transcriptomic analysis reveals the oncogenic role of S6K1 in hepatocellular carcinoma

**DOI:** 10.7150/jca.40726

**Published:** 2020-02-19

**Authors:** Keng Po Lai, Angela Cheung, Cheuk Hin Ho, Nathan Yi-Kan Tam, Jing Woei Li, Xiao Lin, Ting Fung Chan, Nikki Pui-Yue Lee, Rong Li

**Affiliations:** 1 Guanxi Key Laboratory of Tumor Immunology and Microenvironmental Regulation, Guilin Medical University, Guilin, PR China;; 2 Department of Chemistry, City University of Hong Kong, Hong Kong SAR, China;; 3 School of Life Sciences, The Chinese University of Hong Kong, Hong Kong SAR, China;; 4 State Key Laboratory of Agrobiotechnology, Chinese University of Hong Kong, Hong Kong SAR, China;; 5 Department of Surgery, University of Hong Kong, Hong Kong SAR, China.

**Keywords:** S6K1, hepatocellular carcinoma, proliferation, tumorigenicity, transcriptome

## Abstract

The p70 ribosomal protein S6 kinase 1 (S6K1), a serine/threonine kinase, is commonly overexpressed in a variety of cancers. However, its expression level and functional roles in hepatocellular carcinoma (HCC), which ranks as the third leading cause of cancer-related death worldwide, is still largely unknown. In the current report, we show the *in vivo* and *in vitro* overexpression of S6K1 in HCC. In the functional analysis, we demonstrate that S6K1 is required for the proliferation and colony formation abilities in HCC. By using comparative transcriptomic analysis followed by gene ontology enrichment analysis and Ingenuity Pathway Analysis, we find that the depletion of S6K1 can elevate the expression of a cluster of apoptotic genes, tumor suppressor genes and immune responsive genes. Moreover, the knockdown of S6K1 is predicted to reduce the tumorigenicity of HCC through the regulation of hubs of genes including STAT1, HDAC4, CEBPA and ONECUT1. In conclusion, we demonstrate the oncogenic role of S6K1 in HCC, suggesting the possible use of S6K1 as a therapeutic target for HCC treatment.

## Introduction

Hepatocellular carcinoma (HCC) is one of the most prevalent malignancies, ranking as the third leading cause of cancer-related death worldwide. The main reason for the high mortality rate is due to its poor prognosis and the lack of satisfactory curative pharmacological treatment 1. Sorafenib, a small multi-kinase inhibitor, is the only FDA approved drug-based therapy for advanced HCC 2. Therefore, it is necessary to identify an alternative approach for HCC treatment. It has been reported that a number of cell signaling pathways such as NF-κB, TAK, ZFX, PI3K, PKB and GSK-3β play important roles in the tumorigenicity of HCC 3-7. A better understanding on the mechanism underlying the cell signaling which regulates the development and growth of HCC may help us to develop a novel molecular therapeutic target for HCC treatment.

PI3K-Akt-mTOR pathway is a major tumor-initiating pathway in hepatocellular carcinoma, and is elevated in up to 50% of tumors 8. The p70 ribosomal protein S6 kinase 1 (S6K1), a serine/threonine kinase, is a downstream effector of the mTOR pathway. S6K1 has been reported to be commonly overexpressed in a variety of cancers such as ovarian cancer, prostate cancer and leukemia 9-11. Additionally, we have previously demonstrated that S6K1 acts as a multifaceted regulator of the Mdm2-p53 pathway 12. Also, p53 is important key mediator which controls cell proliferation, apoptosis and cell cycle in HCC 13-14, suggesting that S6K1 might also play an important functional role in the development of HCC. In this report, we showed that S6K1 is commonly overexpressed in HCC both *in vivo* and *in vitro*. In addition, we depleted the expression of S6K1 in three HCC cell lines including Hep3B, PLC5 and HepG2, followed by functional and transcriptomic analyses, to understand the role of S6K1 in HCC and the mechanisms underlying the hepatocarcinogenicity of S6K1, respectively. We showed that the knockdown of S6K1 could reduce the cell proliferation and colony formation abilities of HCC cells. In the comparative transcriptomic analysis, we demonstrated that the depletion of S6K1 recused the expression of tumor suppressor genes and cell apoptotic genes. The Ingenuity Pathways Analysis (IPA) further highlighted that the depletion of S6K1 could reduce the tumorigenicity of HCC through the regulation of gene cluster including STAT1, HDAC4, CEBPA and ONECUT1. In this study, the identification and functional characterization of S6K1 in HCC provides new insights into the tumorigenic role of S6K1 in HCC and the possible use of S6K1 as a therapeutic target for HCC treatment.

## Materials and Methods

### Patient sample collection

Retrospectively collected clinical specimens were used in this study. Patient consents have been obtained. IRB has approved on the use of clinical specimens for research. Tissue specimens were preserved immediately in RNAlater (for RNA extraction) or fixed in formalin for preparation of paraffin blocks. The collection and processing of clinical specimens were performed by Surgical Tissue Bank staff in Department of Surgery, The University of Hong Kong. Patients treated with tumor resection in Queen Mary Hospital were included. Patients treated with neoadjuvant or adjuvant treatment were excluded. Clinico-pathological parameters required for this study are available from Surgical Tissue Bank.

### Cell culture

The human HCC cell lines Hep3B, PLC5 and HepG2 were cultured in DMEM medium (Invitrogen) supplemented with 10% fetal bovine serum. The cells were supplied with 95% of air and 5% of carbon dioxide (CO_2_), and were kept at 37°C.

### Lentiviral packaging and transduction

HEK293FT cells were transfected with packaging plasmids pCMV-VSV-G, pRSV-Rev and pMDLg/pRRE along with MISSION® non-target shRNA plasmid or MISSION® shS6K1 plasmid (Sigma) using the Lipofectamine 2000 transfection reagent (Intvitrogen). After 48 hours post-transfection, the viral supernatant was precipitated with PEG-it Virus Precipitation Solution at a 1:4 ratio to produce a concentrated viral stock. Hep3B, PLC5 and HepG2 were plated 1 day before transduction. After 48 hr post-transduction, cells were selected in medium containing 2 µg/ml puromycin for 10 days. The knockdown efficiency of S6K1 was examined using qPCR and Western Blotting.

### cDNA synthesis and quantitative RT-PCR (qRT-PCR)

RNA was isolated from the cell lines using Trizol reagent (Invitrogen) according to the manufacturer's instruction. For each sample, 1 µg of total RNA was subjected to first strand cDNA synthesis using SuperScript® VILO™ cDNA Synthesis Kit (Thermo Fisher). The expression levels of the target genes were determined using the KAPA SYBR FAST qPCR kit. The primer sequences were listed in **Supplementary Table S1**. To adjust for variations in starting template, gene expression was normalized against GAPDH.

### Western Blot Analysis

50 µg of protein was resolved on 8% sodium dodecyl sulfate-polyacrylamide gel electrophoresis (SDS-PAGE) and transferred electrophoretically onto a polyvinylidene fluoride membrane. The blots were incubated with specified antibodies against S6K1 (1:1000; Cell Signaling), or beta-actin (1:10000; Chemicon). After incubation with secondary antibody conjugated to horseradish peroxidase, the results were visualized using enhanced chemiluminescence detection (GE Healthcare, Piscataway, NJ).

### Cell proliferation assays

Stable transfected HCC cells were seeded in a 96-well plate at a cell density of 3 x 10^3^ cells per well, with 8 replicate wells. The cells were allowed to incubate for 1, 3 and 7 days. After the incubation, cell proliferation was measured by the WST-1 assay (Sigma). The colorimetric product formed was measured at an absorbance of 570nm.

### Colony formation assay

In order to determine the role of S6K1 in HCC tumorigenicity, colony formation was conducted as previously described 3. Briefly, stable transfected HCC cells were seeded in a 6-well plate (200 cells per well). After an incubation period of 14 days, the cells were stained with 1% crystal violet (in 100% methanol) for 15 min, followed by detaining with water. Colonies (more than 20 cells/colony) were counted.

### Comparative transcriptome analysis

Total RNA was isolated from the stable transfected cells (shCtrl and shS6K1) from the Hep3B and PLC5 cell lines using the mirVanaTM RNA isolation kit (Applied Biosystems). The samples with a RNA Integrity Number (RIN) greater than 8 were used for RNA library construction. The RNA library was sequenced by the Beijing Genomics Institute (Wuhan, China). Single-end reads with a 50 bp read-length were sequenced on the BGISEQ-500RS sequencer. Sequence-reads were in turn dynamically trimmed according to BWA's - q algorithm*.* All downstream analyses were based on quality-trimmed reads 15,16. Quality-trimmed sequence reads were mapped to the Human genome reference hg19 using STAR v2.3.0e. Read-counts of genes were quantified against Gencode v19 using HTSeq-count (v0.6.0). Read-count data were then subjected to differential expression analysis using the edgeR package 17. Genes with a |log2 (fold change: shS6K1/shCtrl)| > 0.6 were considered as differentially expressed genes (DEGs).

### Statistical Analysis

Quantitative data was described as the means with standard deviation (SD). Wilcoxon matched-pairs signed rank test was used to compare expression level difference of S6K1 between studied groups. In the functional study, the effect of S6K1 was compared using the paired Student t-test. A *p* value < 0.05 was considered as statistically significant. All statistical analyses were performed using GraphPad Prism 3.02 (GraphPad Software Inc., San Diego, CA).

## Results

### S6K1 is commonly overexpressed in human HCC

**Figure 1:**
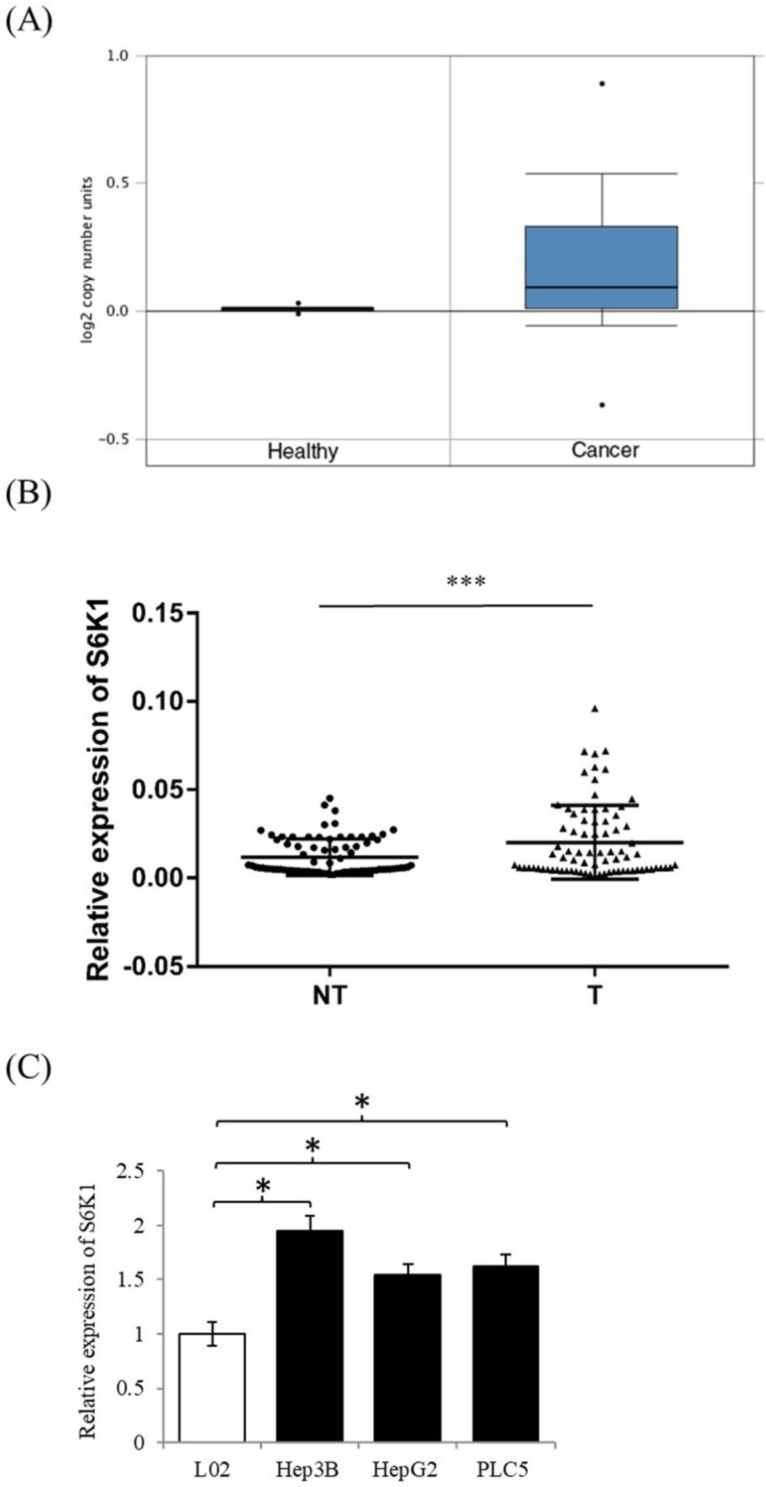
***In vivo* and *in vitro* overexpression of S6K1 in HCC.** (A) copy-number-gain of S6K1 in human hepatocellular carcinoma. DNA copy-number variation of TCGA data is obtained from Oncomine (https://www.oncomine.org). (B) Overexpression of S6K1 transcript in a cohort of 80 HCC tumors (T) and paired adjacent non-tumoral livers (NT). (C) Upregulation of S6K1 in HCC cell lines, Hep3B, PLC5 and HepG2 as compared to the normal liver cell line L02. The mRNA expression level of S6K1 was determined by qRT-PCR. Data are presented as the mean ± SD values (***p = 0.0001, *p < 0.05).

In order to examine the expression level of S6K1 in HCC, we firstly used the publicly available datasets from Oncomine(available at: https://www.oncomine.org) to survey DNA copy-number variation of S6K1 in HCC. There was a significant amplification in the copy-number-gain of S6K1 in HCC as compared to normal liver tissue (**Figure 1A**). We then conducted qPCR analysis to determine the expression of S6K1 in a cohort of 80 HCC tumors (T) and paired adjacent non-tumoral livers (NT). Our results demonstrated a significant induction of S6K1 in HCC tumors (42.5%), as compared with their adjacent nonmalignant livers (log10 T/NT > 0.2, *p* = 0.0001) (**Figure 1B**). We also attempted to correlate the expression of S6K1 in HCC tumors with the clinicopathological features of patients. But no significant correlation was observed (**Supplementary Table S2**). Additionally, we demonstrated that HCC cell lines (Hep3B, HepG2 and PLC5) had elevated S6K1 expression as compared to the normal liver cell line L02 (**Figure 1C**). Taken together, our results showed that S6K1 is commonly overexpressed in HCC both *in vivo* and *in vitro*.

### Depletion of S6K1 suppresses HCC cell proliferation

**Figure 2:**
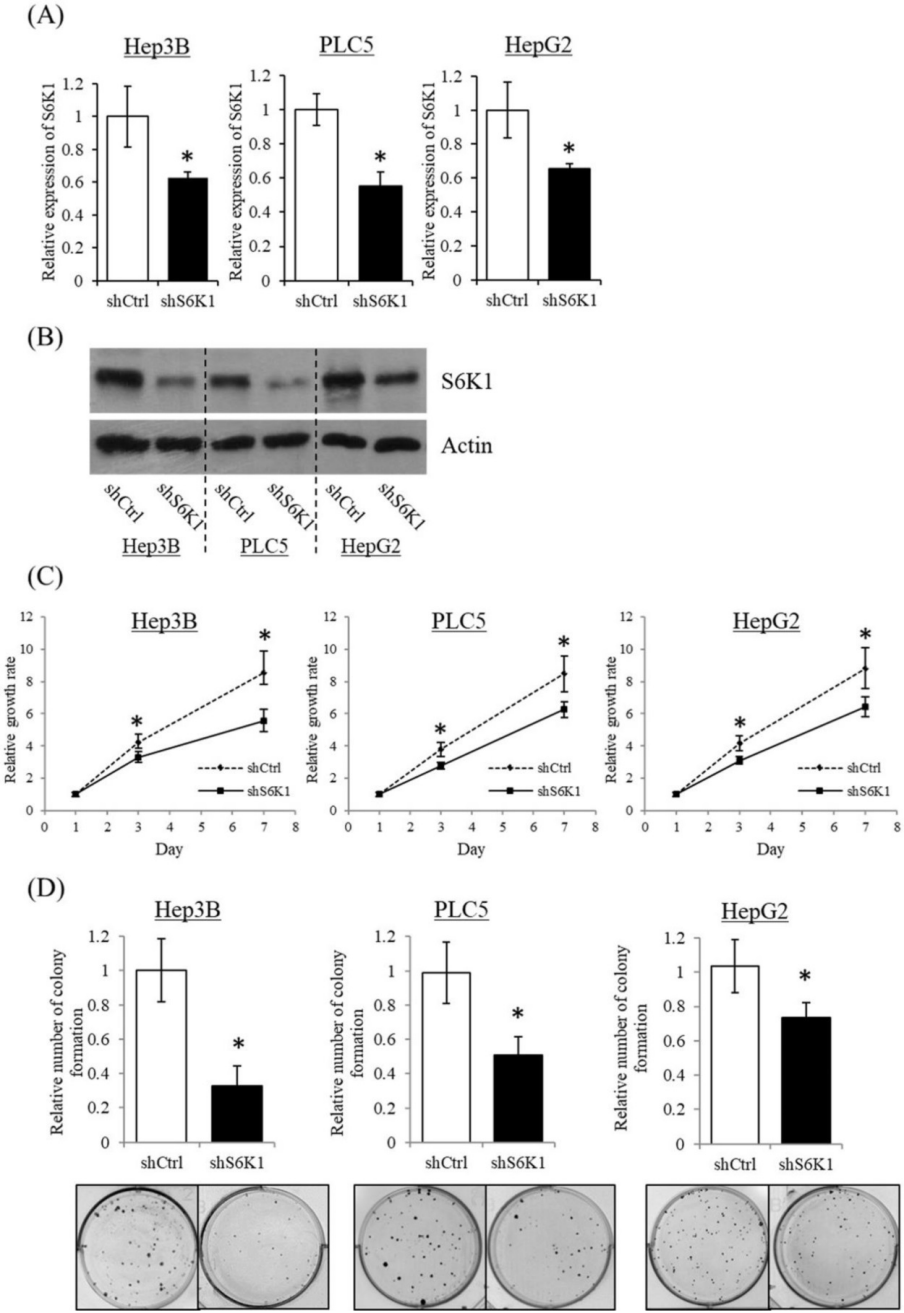
** Depletion of S6K1 suppresses* in vitro* proliferation of HCC cells.** (A) Knockdown of S6K1 in HCC cell lines. qRT-PCR and western blotting demonstrated the knockdown efficiency of S6K1 in S6K1-depleted Hep3B, PLC5 and HepG2. (B-C) Depletion of S6K1 suppressed *in vitro* cell proliferation of HCC. The cell proliferation of S6K1-depleted cell line was determined by (B) WST-1 assay and (C) colony formation assay. Data are presented as the mean ± s.e.m. values (*p < 0.05).

To gain insight into the functional role of increased S6K1 expression in HCC, three HCC cell lines (Hep3B, HepG2 and PLC5) showing elevated S6K1 expression were used to establish S6K1-deficient stable clones (shS6K1) by lentiviral transfection. The knockdown efficiency of S6K1 was determined by qRT-PCR (**Figure 2A**) and Western blot (**Figure 2B**). Our results demonstrated the successful depletion of S6K1 in these 3 HCC cell lines. In the functional analysis, the deficiency of S6K1 significantly reduced the *in vitro* proliferation of HCC in the WST-1 assay (**Figure 2C**). In addition, smaller-sized and smaller numbers of colonies were formed in S6K1-depleted cells in the colony formation assay (**Figure 2D**). Collectively, our results suggested that S6K1 is required for the proliferation and growth of HCC.

### S6K1 is required for the expression of apoptotic and tumor suppressor genes in HCC

**Figure 3:**
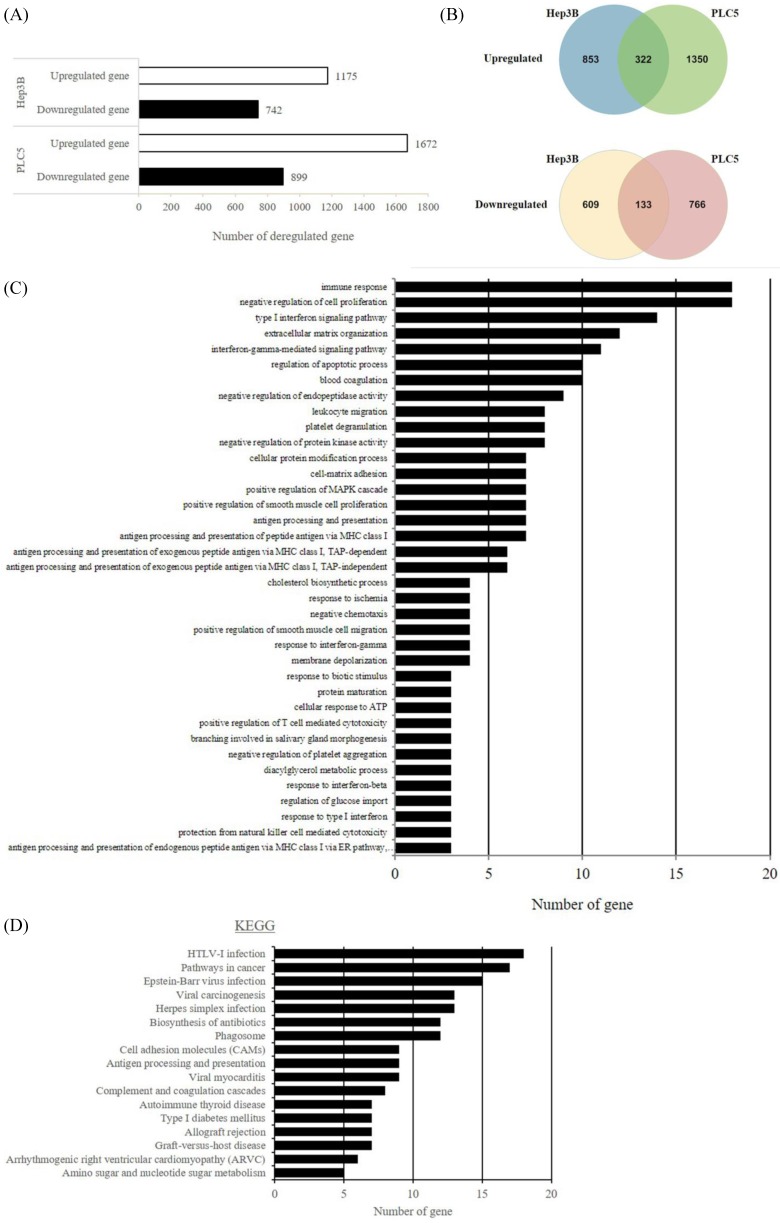
** Depletion of S6K1 reduces the proliferation and regains the apoptosis in HCC.** (A) Gene-annotation enrichment analysis using The Database for Annotation, Visualization and Integrated Discovery (DAVID). Biological processes with *p* < 0.05 were considered statistically significant. (B) KEGG pathway analysis using DAVID.

**Figure 4:**
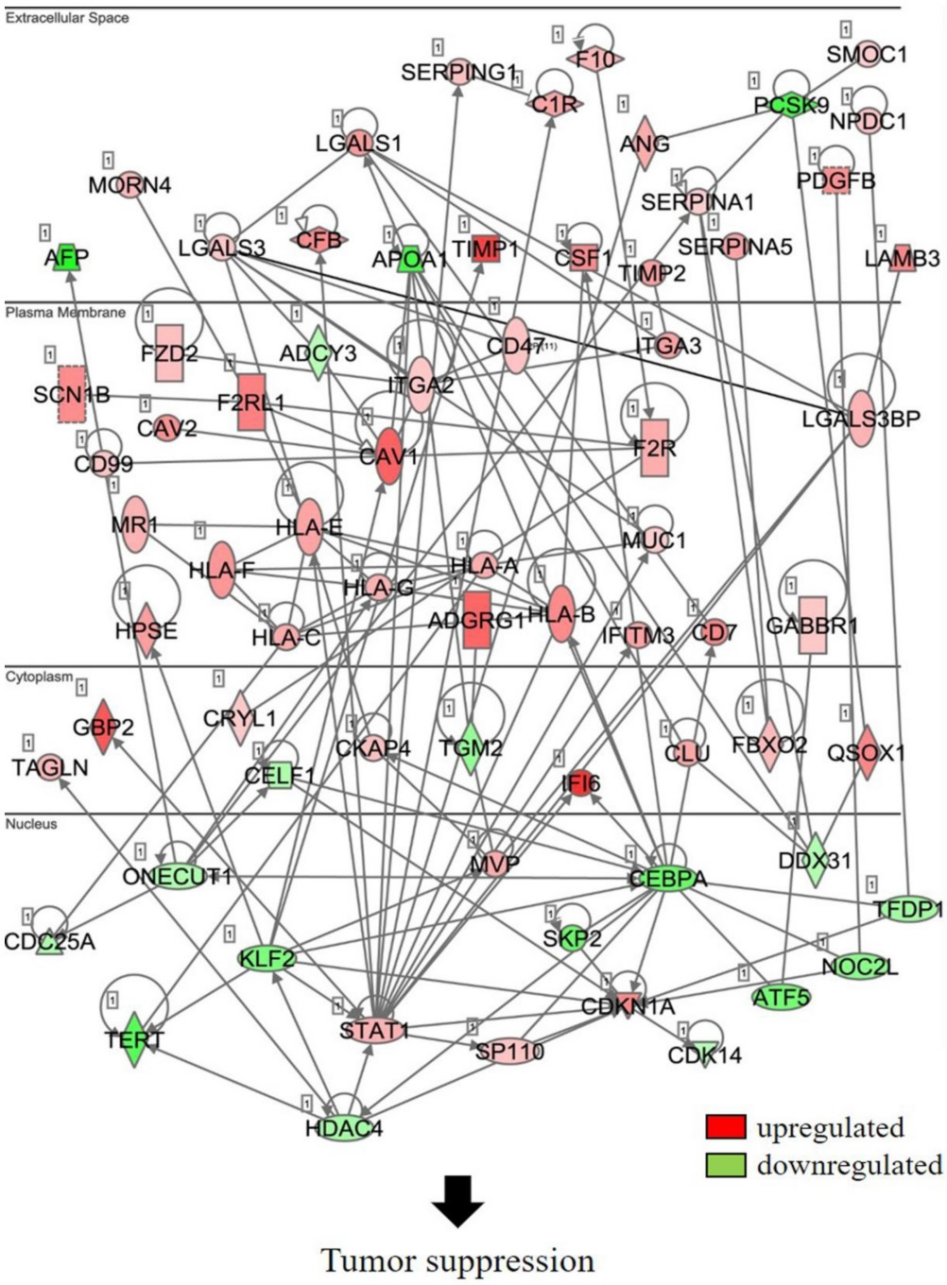
Depletion of S6K1 regulates the gene network to suppress the tumor development.

**Figure 5:**
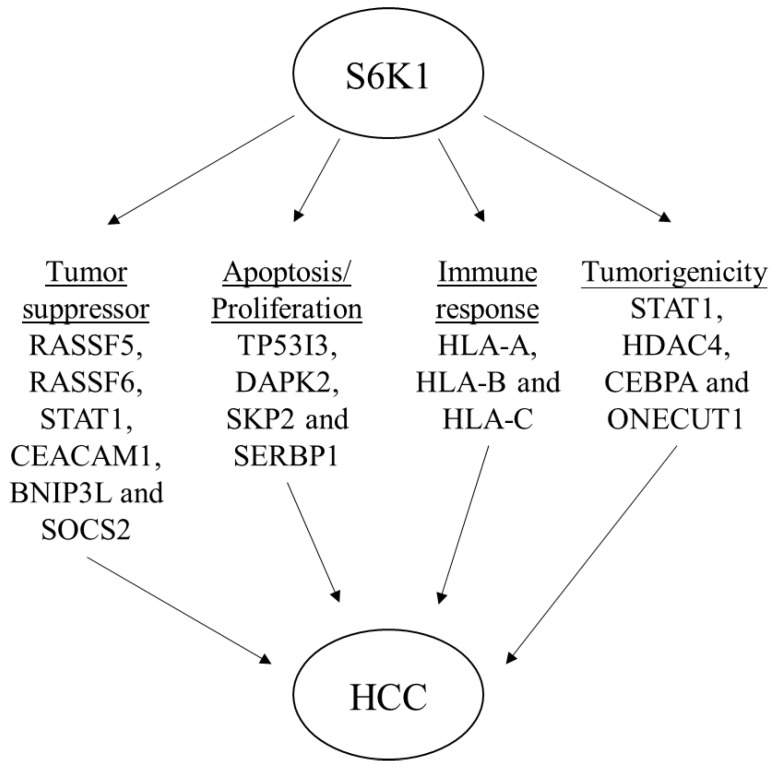
Schematic diagram to summarize the oncogenic role of S6K1 in HCC.

In an attempt to understand the mechanism underlying the hepatocarcinogenicity of S6K1, comparative transcriptomic analysis (shCtrl vs shS6K1) was conducted on the Hep3B and PLC5 cells. 1917 and 2571 differentially expressed genes (DEGs) were found in Hep3B (**Figure 3A, Supplementary Table S3**) and PLC5 (**Figure 3A, Supplementary Table S4**), respectively after the depletion of S6K1. Of these genes, 322 upregulated and 133 downregulated genes were shared in both Hep3B and PLC5 with S6K1 knockdown (**Figure 3B, Supplementary Table S5**). The gene set enrichment analysis on these 455 DEGs using the DAVID tool highlighted 37 biological processes that were controlled by S6K1 *(p* < 0.05) (**Figure 3C, Supplementary Table S6**). It included immune response, antigen processing, interferon-related cell signaling pathways, negative regulation of cell proliferation, and regulation of apoptotic process. We also found that S6K1 is required for the regulation of the apoptotic process through a cluster of genes such as *TP53I3*, *DAPK2*, *SKP2* and *SERBP1* in HCC cells (**Table 1**). In addition, the depletion of S6K1 would stimulate the immune response by overexpressing the major histocompatibility complex (MHC) class I cell surface receptor family such as human leukocyte antigen HLA-A, HLA-B and HLA-C. KEGG pathway analysis further highlighted the role of S6K1 in cancer progression (**Figure 3D, Supplementary Table S7**), in which the depletion of S6K1 could re-express a group of tumor suppressor genes including *RASSF5, RASSF6, STAT1, CEACAM1, BNIP3L* and* SOCS2* (**Table 1**). More importantly, the results of the Ingenuity Pathway Analysis (IPA) in diseases or functions annotation demonstrated that the depletion of SK61 could reduce the tumorigenicity of HCC through the control of hubs of gene including STAT1, HDAC4, CEBPA and ONECUT1 (**Figure 4**), which is reflected by the inactivation of several cancers including liver tumor (**Figure 5 and Table 2, Supplementary Table S8**). Taken together, our results suggested the anti-apoptotic and oncogenic role of S6K1 in the HCC.

### Validation of transcriptome sequencing using qPCR analysis

**Figure 6:**
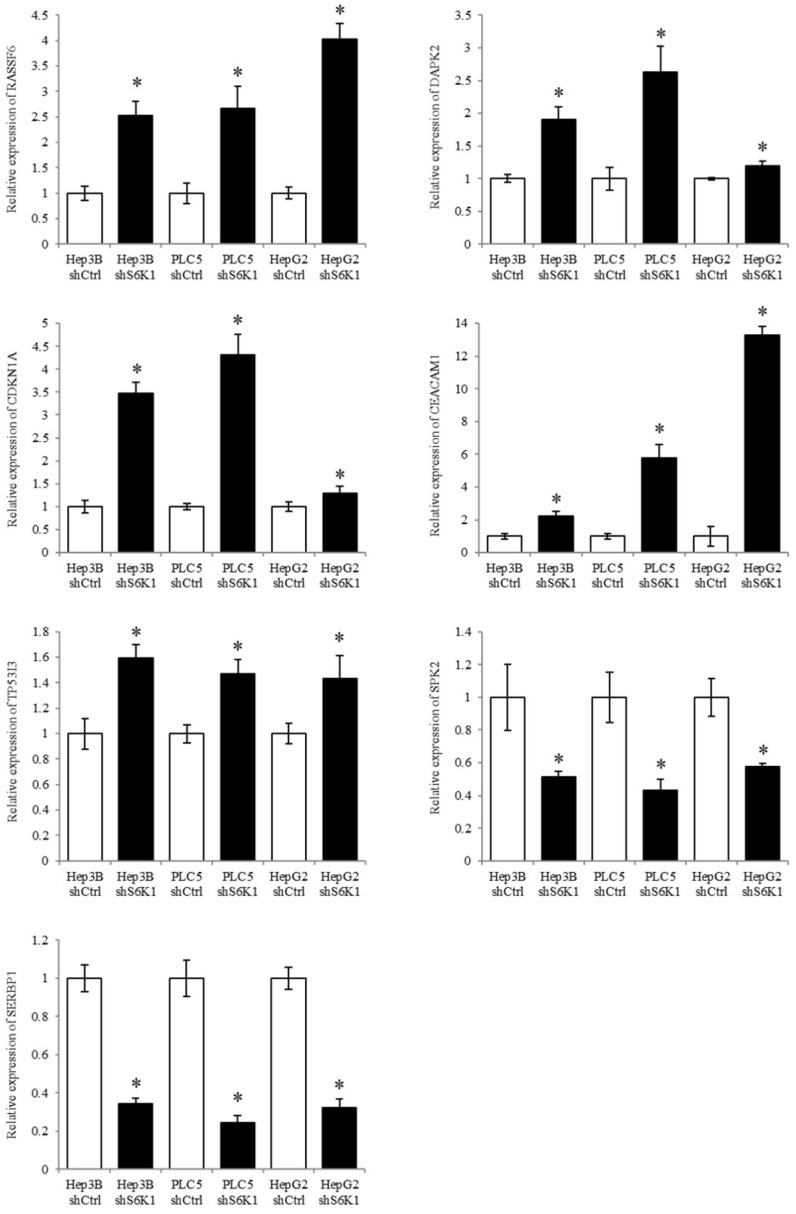
** Quantitative PCR analysis validates the result of transcriptome sequencing.** The expression of tumor suppressor genes, apoptotic genes and cell proliferative genes were determined in S6K1-depleted HCC cell lines, as compared to shCtrl cells using qPCR. Data are presented as the mean ± s.e.m. values (*p < 0.05) (n = 8).

S6K1 controlled tumor suppressor, cell proliferative and cell apoptotic genes (*RASSF6, DAPK2, CDKN1A, CEACAM1, TP53I3, SPK2,* and* SERBP1*) were selected to be validated in the Hep3B, PLC5 and HepG2 cell lines using qPCR analysis. The result of qPCR analysis agreed with the transcriptome sequencing data that *RASSF6, DAPK2, CDKN1A, CEACAM1* and* TP53I3* were induced in the S6K1-depleted HCC cell lines, and *SPK2* and* SERBP1* were suppressed under the S6K1 knockdown (**Figure 6**).

## Discussion

p70 ribosomal protein S6 kinase 1 (S6K1), an important downstream kinase of Akt-mTOR pathway, has been reported to play roles including tumor growth, chemoresistance and self-renewal ability in numerous of cancers such as leukemia, bladder cancer, colorectal carcinomas, neuroblastoma and breast cancer 9, 18-20. But its expression level and functional role in hepatocellular carcinoma (HCC) is still largely unknown. Here, we retrieved the data from Oncomine 21, a cancer microarray database and web-based data-mining platform of genome-wide expression, to show the copy number gain of S6K1 in HCC. Further clinic analysis demonstrated the overexpression of S6K1 transcript in patients with HCC. These results suggested that the upregulation of S6K1 expression in HCC may be due to the chromosomal aberration in genomic level. DNA copy number changes are commonly found in HCC. For instance, microarray-based comparative genomic hybridization (array CGH) analysis has demonstrated the amplification of chromosome 1q21 - q22 and 8q24 regions in HCC which is associated with the promotion of cell motility and epithelial-to-mesenchymal transition in HCC, respectively 22, 23.

Then, we conducted functional and mechanistic characterization of S6K1 in 3 HCC cell lines including Hep3B, HepG2 and PLC5, which covered HCC with different cell properties and ethnic origins 24. HepG2 is hepatitis B virus negative and non-tumorigenic, but Hep3B is hepatitis B virus positive and tumorigenic 25, 26. PLC5 is also hepatitis virus B 27. In the loss-of-functional analysis, we showed the depletion of S6K1 could reduce the proliferation of HCC cells, suggesting that S6K1 is necessary for the growth of HCC. In the mechanistic analysis, we performed comparative transcriptome sequencing to dissect gene networks and pathways related to the tumorigenicity of HCC which are controlled by S6K1. Interestingly, the depletion of S6K1 could alter a number of immune responses such as the activation of MHC class I cell surface receptor members, and the alteration of antigen processing and interferon signaling. The result of this finding suggested a possible use of S6K1 inhibitor together with bevacizumab (anti-VEGF antibody) for regulating immune response against HCC and immunotherapeutic approaches for HCC 28, 29.

Due to the importance of S6K1 in the growth of HCC, we then further looked at the pathways and gene clusters related to cell apoptosis and proliferation in the DVAID analysis. In which, we discovered 2 clusters of gene were controlled by S6K1; one is related to cell apoptosis or cell proliferation including *TP53I3, DAPK2, SKP2* and* SERBP1*. The other cluster including *RASSF5, RASSF6, STAT1, CEACAM1, BNIP3L* and* SOCS2* is related to tumor suppression. S-phase kinase-associated protein 2 (*SKP2*), forms a feedback loop with p21 and p27 that control cell cycle entry and G1/S transition 26. It has been reported that the inhibition of SKP2 resulted in cell cycle arrest in HCC 27, 28. Serpine mRNA binding protein 1 (*SERBP1*) is a protein that binds to the plasminogen activator inhibitor type 1 (PAI-1) mRNA, leading to the regulation of plasminogen activation 29. Previous report showed that the overexpression of SERBP1 in human breast cancer is correlated with favorable prognosis 30. In addition, the activation of *SERBP1* promotes cell proliferation, migration, and invasion in prostate cancer 31. So the suppression of *SERBP1* in S6K1 depletion cells may result in the decrease in tumorigenicity of HCC. Besides, the S6K1 knockdown could re-express a group of apoptotic genes. For instance, tumor protein p53 inducible protein 3 (*TP53I3*), a downstream molecule of the tumor suppressor p53, is reported to activate apoptosis in many cancers such as glioblastoma, papillary thyroid cancer and lung adenoma 32-34. In addition, the re-expression of DAPK2 may be able to reduce the drug resistance of HCC 35, 36.

By using both functional characterization and comparative transcriptomic analysis, we have demonstrated the importance of S6K1 in the proliferation and tumorigenicity of HCC. The discovered regulatory role of S6K1 on the apoptotic and tumor suppressive gene clusters further support that S6K1 is a potential functional target for the application in HCC therapeutics.

## Supplementary Material

Supplementary tables.

## Figures and Tables

**Table 1:** S6K1 controlled apoptotic and tumor suppressor genes in HCC.

Gene symbol	Gene name	Reference
RASSF5	Ras Association Domain Family Member 5	41,42
RASSF6	Ras Association Domain Family Member 6	43-45
TP53I3	Tumor Protein P53 Inducible Protein 3	38,46-48
LGALS1	Galectin 1	49-51
BNIP3L	BCL2 Interacting Protein 3 Like	52-55
NDRG1	N-Myc Downstream Regulated 1	56-59
DAPK2	Death Associated Protein Kinase 2	60-63
STAT1	Signal Transducer And Activator Of Transcription 1	64-66
SERBP1	SERPINE1 MRNA Binding Protein 1	34,67
SKP2	S-Phase Kinase-Associated Protein 2	32,68-70
CEACAM1	carcinoembryonic antigen related cell adhesion molecule 1	68-73
SOCS2	suppressor of cytokine signaling 2	74-76

**Table 2:** Depletion of S6K1 reduced the tumorigenicity of HCC.

Diseases or Functions Annotation	p-value	Predicted Activation State	Activation z-score
lymphocytic cancer	0.00000862	Decreased	-2.183
breast or colorectal cancer	0.0000549	Decreased	-2.18
endocrine gland tumor	0.000176	Decreased	-2.219
neuroendocrine tumor	0.000196	Decreased	-2.219
lymphoid cancer	0.000291	Decreased	-2.183
breast cancer	0.000916	Decreased	-2.415
breast or ovarian cancer	0.00104	Decreased	-2.415
lymphoproliferative malignancy	0.00126	Decreased	-2.371
liver tumor	0.00264	Decreased	-2.109
