# Supplementary material for: Transcriptomic analysis reveals the oncogenic role of S6K1 in hepatocellular carcinoma
Source: J Cancer. 2020 Feb 19;11(9):2645–55. doi: 10.7150/jca.40726 (PMC7065997; doi:10.7150/jca.40726)
Supplement: Supplementary file 1 — Supplementary tables. [file jcav11p2645s1.pdf]

Supplementary Table 1

Gene	primer sequence
S6K1_F	taaagggggctatggaaagg
S6K1_R	ttaagcaccttcatggcaaat
RASSF6_F	cccaggattttgctcttcac
RASSF6_R	cctctgcagtagcggaatgt
CDKNIA_F	ccgaagtcagttccttgtgg
CDKNIA_R	catgggttctgacggacat
DAPK2_F	acgtggtgctcatccttga
DAPK2_R	tggcctcctcctcactca
TP53I3_F	tctctgaagcaacgctgaaa
TP53I3_R	gtaggatccgcctatgcagt
SKP2_F	ctgtctcaaggggtgattgc
SKP2_R	ttcgataggtccatgtgctg
SERBP1_F	gcagcaggaccgacaagt
SERBP1_R	tgtcttatggcatccagttaagc
CEACAM1_F	cccatcatgctgaacgtaaa
CEACAM1_R	agggccactactccaatcac
GAPDH_F	ccccggtttctataaattgagc
GAPDH_R	cgaacaggaggagcagagag

Supplementary Table 2

Clinicopathological features	Frequency (%)	S6K1 overexpression Mean ± SD/Number of cases		p value*
		Negative	Positive	
Age (year)				
<59	32 (46.4%)	21	11	0.508
≥59	37 (53.6%)	27	10	
Sex				
Male	51 (73.9%)	38	13	0.133
Female	18 (26.1%)	10	8	
Family History				
Yes	12 (17.4%)	8	4	0.81
No	57 (82.6%)	40	17	
Smoke				
No	37 (53.6%)	22	15	0.05
Yes	32 (46.4%)	26	6	
Drink				
No	47 (68.1%)	31	16	0.341
Yes	22 (31.9%)	17	5	
HBV carrier				
No	16 (23.2%)	11	5	0.936
Yes	53 (76.8%)	37	16	
Albumin				
(g/l)		39.0±4.5	39.1±5.5	0.942
HBsAg				
Positive	61 (88.4%)	42	19	0.722
Negative	8 (11.6%)	6	2	
Tumor recurrence				
Absence	20 (29.0%)	14	6	0.96
Presence	49 (71.0%)	34	15	
Tumor size (cm)				
		8.0±4.9	8.8±6.1	0.599
Number of tumor nodules				
1	46 (66.7%)	31	15	0.579
≥2	23 (33.3%)	17	6	
Differentiation**				
Well	9 (13.4%)	4	5	0.092
Moderately	42 (70.5%)	29	13	
Poorly	15 (16.1%)	13	2	
Venous infiltration**				
Absence	34 (51.5%)	20	14	0.092
Presence	32 (48.5%)	25	7	
TNM				
I/II	31 (44.9%)	19	12	0.177
III/IV	38 (55.1%)	29	9	
AJCC				
I/II	40 (58.0%)	27	13	0.661
III/IV	29 (42.0%)	21	8	

Supplementary Table 3

Gene symbol	log2 fold change (shS6K1/shCtrl)	log2 counts per million	Gene ID	Gene description
MMP7	10.78	2.70	ENSG00000137673	matrix metallopeptidase 7
LCN2	7.06	2.15	ENSG00000148346	lipocalin 2
PIRT	6.19	2.21	ENSG00000233670	phosphoinositide interacting regulator of transient receptor potential channels
GAS7	6.01	2.58	ENSG00000007237	growth arrest specific 7
TM4SF1	5.27	4.29	ENSG00000169908	transmembrane 4 L six family member 1
EHF	5.14	2.13	ENSG00000135373	ETS homologous factor
TPPP3	4.96	1.04	ENSG00000159713	tubulin polymerization promoting protein family member 3
TNF	4.68	1.32	ENSG00000230108	tumor necrosis factor
ZNF385C	4.59	2.73	ENSG00000187595	zinc finger protein 385C
SULT1E1	4.48	2.28	ENSG00000109193	sulfotransferase family 1E member 1
FEZ1	4.46	3.02	ENSG00000149557	fasciculation and elongation protein zeta 1
RP11-79H23.3	4.36	1.71	ENSG00000261618	N/A
PLAT	4.20	2.02	ENSG00000104368	plasminogen activator, tissue type
HAS2	4.05	3.55	ENSG00000170961	hyaluronan synthase 2
ITGAM	3.95	2.42	ENSG00000169896	integrin subunit alpha M
ITGB6	3.82	1.21	ENSG00000115221	integrin subunit beta 6
BIRC3	3.77	6.99	ENSG00000023445	baculoviral IAP repeat containing 3
AZGP1	3.70	2.72	ENSG00000160862	alpha-2-glycoprotein 1, zinc-binding
ZDHHC1	3.65	1.87	ENSG00000159714	zinc finger DHHC-type containing 1
TNFRSF9	3.59	1.82	ENSG00000049249	TNF receptor superfamily member 9
FOS	3.56	3.04	ENSG00000170345	Fos proto-oncogene, AP-1 transcription factor subunit
COL1A2	3.44	2.30	ENSG00000164692	collagen type I alpha 2 chain
WNT10A	3.43	1.51	ENSG00000135925	Wnt family member 10A
CDC20B	3.35	4.15	ENSG00000164287	cell division cycle 20B
PLAU	3.33	3.53	ENSG00000122861	plasminogen activator, urokinase
PLXDC2	3.30	4.99	ENSG00000120594	plexin domain containing 2
TLR1	3.24	1.90	ENSG00000174125	toll like receptor 1
SHISA4	3.23	1.77	ENSG00000198892	shisa family member 4
GBP2	3.19	3.49	ENSG00000162645	guanylate binding protein 2
POU2F2	3.12	3.87	ENSG00000028277	POU class 2 homeobox 2

CTNNA2	3.08	3.05	ENSG00000066032	catenin alpha 2
PLA1A	3.08	2.47	ENSG00000144837	phospholipase A1 member A
OR12D2	3.07	2.24	ENSG00000235966	olfactory receptor family 12 subfamily D member 2 (gene/pseudogene)
MRC2	3.04	1.61	ENSG00000011028	mannose receptor C type 2
SLFN11	3.04	3.10	ENSG00000172716	schlafen family member 11
CXCL3	3.00	5.15	ENSG00000163734	C-X-C motif chemokine ligand 3
NT5E	2.97	6.48	ENSG00000135318	5'-nucleotidase ecto
CXCL2	2.96	4.72	ENSG00000081041	C-X-C motif chemokine ligand 2
CADPS	2.95	2.79	ENSG00000163618	calcium dependent secretion activator
STAT4	2.92	2.11	ENSG00000138378	signal transducer and activator of transcription 4
COL11A2	2.89	2.56	ENSG00000206290	collagen type XI alpha 2 chain
S100A11	2.87	5.90	ENSG00000163191	S100 calcium binding protein A11
CTSS	2.85	2.47	ENSG00000163131	cathepsin S
CAV1	2.84	4.71	ENSG00000105974	caveolin 1
GALNT6	2.81	1.14	ENSG00000139629	polypeptide N-acetylgalactosaminyltransferase 6
UNC93A	2.81	1.74	ENSG00000112494	unc-93 homolog A (C. elegans)
LOXL2	2.81	5.27	ENSG00000134013	lysyl oxidase like 2
TIMP1	2.80	3.35	ENSG00000102265	TIMP metallopeptidase inhibitor 1
ACTA1	2.79	2.15	ENSG00000143632	actin, alpha 1, skeletal muscle
UGT2B15	2.78	2.58	ENSG00000277132	UDP glucuronosyltransferase family 2 member B15
MMP19	2.73	1.94	ENSG00000123342	matrix metallopeptidase 19
OLFML2A	2.73	3.18	ENSG00000185585	olfactomedin like 2A
LTB	2.72	8.05	ENSG00000223448	lymphotoxin beta
C7	2.71	3.36	ENSG00000112936	complement C7
NEURL3	2.69	2.97	ENSG00000163121	neuralized E3 ubiquitin protein ligase 3
KIAA0319	2.65	1.61	ENSG00000137261	KIAA0319
RHOJ	2.65	1.79	ENSG00000126785	ras homolog family member J
PLCXD3	2.64	4.22	ENSG00000182836	phosphatidylinositol specific phospholipase C X domain containing 3
MTMR11	2.60	2.49	ENSG00000014914	myotubularin related protein 11
INHA	2.59	1.10	ENSG00000123999	inhibin alpha subunit
QPCT	2.58	1.45	ENSG00000115828	glutaminyl-peptide cyclotransferase
IFI6	2.58	4.57	ENSG00000126709	interferon alpha inducible protein 6

S1PR1	2.46	1.35	ENSG00000170989	sphingosine-1-phosphate receptor 1
IL7	2.44	1.23	ENSG00000104432	interleukin 7
CACNG4	2.42	5.27	ENSG00000075461	calcium voltage-gated channel auxiliary subunit gamma 4
RND1	2.41	5.29	ENSG00000172602	Rho family GTPase 1
EGR1	2.40	7.59	ENSG00000120738	early growth response 1
CXCL10	2.37	2.92	ENSG00000169245	C-X-C motif chemokine ligand 10
TNFAIP3	2.35	6.15	ENSG00000118503	TNF alpha induced protein 3
SAMD14	2.34	2.27	ENSG00000167100	sterile alpha motif domain containing 14
PLXNB3	2.34	2.67	ENSG00000198753	plexin B3
LYZ	2.33	5.26	ENSG00000090382	lysozyme
AKR1D1	2.32	1.52	ENSG00000122787	aldo-keto reductase family 1 member D1
FAM46C	2.31	2.39	ENSG00000183508	family with sequence similarity 46 member C
HLA-DMA	2.31	1.75	ENSG00000243215	major histocompatibility complex, class II, DM alpha
BIK	2.30	1.41	ENSG00000100290	BCL2 interacting killer
SCUBE2	2.29	1.32	ENSG00000175356	signal peptide, CUB domain and EGF like domain containing 2
TUBA1A	2.28	5.83	ENSG00000167552	tubulin alpha 1a
VNN3	2.25	4.09	ENSG00000093134	vanin 3
FRMD4A	2.23	3.86	ENSG00000151474	FERM domain containing 4A
STRA6	2.21	4.69	ENSG00000137868	stimulated by retinoic acid 6
ZC3H12A	2.20	5.81	ENSG00000163874	zinc finger CCCH-type containing 12A
RP11-47L3.1	2.20	5.02	ENSG00000267364	N/A
LAMC2	2.19	4.17	ENSG00000058085	laminin subunit gamma 2
EFNA5	2.16	1.12	ENSG00000184349	ephrin A5
PTAFR	2.16	1.40	ENSG00000169403	platelet activating factor receptor
RP11-20I20.4	2.15	4.74	ENSG00000273179	N/A
UGT3A1	2.14	4.22	ENSG00000145626	UDP glycosyltransferase family 3 member A1
CD109	2.14	2.11	ENSG00000156535	CD109 molecule
SLC44A2	2.13	2.55	ENSG00000129353	solute carrier family 44 member 2
LINC00511	2.11	4.94	ENSG00000227036	long intergenic non-protein coding RNA 511
CSF1	2.11	5.82	ENSG00000184371	colony stimulating factor 1
ODAM	2.09	2.12	ENSG00000109205	odontogenic, ameloblast asssociated

PCSK5	2.08	5.70	ENSG00000099139	proprotein convertase subtilisin/kexin type 5
ICAM1	2.08	8.83	ENSG00000090339	intercellular adhesion molecule 1
BAALC	2.08	1.15	ENSG00000164929	brain and acute leukemia, cytoplasmic
MAP3K8	2.07	4.13	ENSG00000107968	mitogen-activated protein kinase kinase kinase 8
RP11-103J17.2	2.06	3.19	ENSG00000261761	N/A
IP6K3	2.05	1.62	ENSG00000161896	inositol hexakisphosphate kinase 3
HMCN1	2.05	1.81	ENSG00000143341	hemicentin 1
QSOX1	2.05	7.20	ENSG00000116260	quiescin sulfhydryl oxidase 1
SGK1	2.04	6.54	ENSG00000118515	serum/glucocorticoid regulated kinase 1
CXCL6	2.02	5.93	ENSG00000124875	C-X-C motif chemokine ligand 6
NCOA7	1.99	6.18	ENSG00000111912	nuclear receptor coactivator 7
GXYLT2	1.98	2.57	ENSG00000172986	glucoside xylosyltransferase 2
PDGFB	1.97	4.76	ENSG00000100311	platelet derived growth factor subunit B
C6	1.97	4.03	ENSG00000039537	complement C6
PDK4	1.96	1.15	ENSG00000004799	pyruvate dehydrogenase kinase 4
SP140L	1.96	2.20	ENSG00000185404	SP140 nuclear body protein like
COL7A1	1.94	1.95	ENSG00000114270	collagen type VII alpha 1 chain
UGT2A3	1.92	3.70	ENSG00000135220	UDP glucuronosyltransferase family 2 member A3
HOXD1	1.91	3.87	ENSG00000128645	homeobox D1
GABARAPL1	1.90	4.19	ENSG00000139112	GABA type A receptor associated protein like 1
C3orf70	1.90	1.51	ENSG00000187068	chromosome 3 open reading frame 70
EMP3	1.90	1.28	ENSG00000142227	epithelial membrane protein 3
CACNA1D	1.89	3.28	ENSG00000157388	calcium voltage-gated channel subunit alpha1 D
SV2A	1.88	4.91	ENSG00000159164	synaptic vesicle glycoprotein 2A
FSTL1	1.88	4.64	ENSG00000163430	follistatin like 1
PDIA2	1.88	1.26	ENSG00000185615	protein disulfide isomerase family A member 2
C1R	1.88	3.42	ENSG00000159403	complement C1r
CA9	1.87	2.34	ENSG00000107159	carbonic anhydrase 9
GPX8	1.86	2.41	ENSG00000164294	glutathione peroxidase 8 (putative)
MOXD1	1.86	4.98	ENSG00000079931	monooxygenase DBH like 1
SDC4	1.85	8.89	ENSG00000124145	syndecan 4

C8orf4	1.85	6.41	ENSG00000176907	chromosome 8 open reading frame 4
SNAP91	1.83	3.36	ENSG00000065609	synaptosome associated protein 91
PRG4	1.82	2.18	ENSG00000116690	proteoglycan 4
UNC5CL	1.82	1.63	ENSG00000124602	unc-5 family C-terminal like
LRP4	1.81	4.66	ENSG00000134569	LDL receptor related protein 4
DACH1	1.80	1.21	ENSG00000276644	dachshund family transcription factor 1
SEMA3D	1.80	3.70	ENSG00000153993	semaphorin 3D
RHOH	1.80	1.03	ENSG00000168421	ras homolog family member H
GUCY1B3	1.80	3.58	ENSG00000061918	guanylate cyclase 1 soluble subunit beta
MUC6	1.80	2.46	ENSG00000283350	mucin 6, oligomeric mucus/gel-forming
COTL1	1.79	6.06	ENSG00000103187	coactosin like F-actin binding protein 1
FRMD4B	1.79	2.12	ENSG00000114541	FERM domain containing 4B
SPON2	1.79	8.07	ENSG00000159674	spondin 2
DLGAP1-AS2	1.78	2.64	ENSG00000262001	DLGAP1 antisense RNA 2
SOD3	1.77	2.02	ENSG00000109610	superoxide dismutase 3, extracellular
FAXDC2	1.77	3.18	ENSG00000170271	fatty acid hydroxylase domain containing 2
FGF14	1.76	2.14	ENSG00000102466	fibroblast growth factor 14
HLA-B	1.74	5.33	ENSG00000206450	major histocompatibility complex, class I, B
PNMAL2	1.74	1.31	ENSG00000204851	paraneoplastic Ma antigen family like 2
RASSF2	1.73	1.69	ENSG00000101265	Ras association domain family member 2
BHLHE41	1.73	1.74	ENSG00000123095	basic helix-loop-helix family member e41
SOD2	1.73	8.97	ENSG00000112096	superoxide dismutase 2, mitochondrial
CLDN11	1.72	3.78	ENSG00000013297	claudin 11
TNFAIP2	1.72	8.33	ENSG00000185215	TNF alpha induced protein 2
PIK3AP1	1.71	6.58	ENSG00000155629	phosphoinositide-3-kinase adaptor protein 1
SOCS1	1.71	1.73	ENSG00000185338	suppressor of cytokine signaling 1
PDGFRA	1.70	5.04	ENSG00000134853	platelet derived growth factor receptor alpha
HKDC1	1.70	4.78	ENSG00000156510	hexokinase domain containing 1
PIK3IP1	1.69	4.24	ENSG00000100100	phosphoinositide-3-kinase interacting protein 1
CXCL1	1.69	7.68	ENSG00000163739	C-X-C motif chemokine ligand 1

C10orf10	1.68	5.12	ENSG00000165507	chromosome 10 open reading frame 10
KLKB1	1.68	1.03	ENSG00000164344	kallikrein B1
SPOCK2	1.68	6.30	ENSG00000107742	SPARC/osteonectin, cwcv and kazal like domains proteoglycan 2
JAM2	1.67	4.33	ENSG00000154721	junctional adhesion molecule 2
CAV2	1.67	5.26	ENSG00000105971	caveolin 2
NFATC4	1.66	3.86	ENSG00000100968	nuclear factor of activated T-cells 4
AKAP7	1.66	2.84	ENSG00000118507	A-kinase anchoring protein 7
PDLIM3	1.65	2.66	ENSG00000154553	PDZ and LIM domain 3
ZNF610	1.65	2.68	ENSG00000167554	zinc finger protein 610
POPDC2	1.65	1.09	ENSG00000121577	popeye domain containing 2
ADPRH	1.64	2.00	ENSG00000144843	ADP-ribosylarginine hydrolase
RP11-284F21.9	1.64	2.09	ENSG00000272068	N/A
HLA-E	1.63	5.68	ENSG00000225201	major histocompatibility complex, class I, E
DACH2	1.62	2.33	ENSG00000126733	dachshund family transcription factor 2
RNF150	1.62	2.12	ENSG00000170153	ring finger protein 150
UBE2L6	1.62	3.74	ENSG00000156587	ubiquitin conjugating enzyme E2 L6
COL4A4	1.61	1.94	ENSG00000081052	collagen type IV alpha 4 chain
HR	1.61	4.39	ENSG00000168453	hair growth associated
WWTR1	1.60	6.44	ENSG00000018408	WW domain containing transcription regulator 1
PAX6	1.59	1.84	ENSG00000007372	paired box 6
CDC42EP2	1.59	3.43	ENSG00000149798	CDC42 effector protein 2
BMF	1.59	7.44	ENSG00000104081	Bcl2 modifying factor
CCL20	1.59	8.78	ENSG00000115009	C-C motif chemokine ligand 20
APLP1	1.59	3.58	ENSG00000105290	amyloid beta precursor like protein 1
GATA6-AS1	1.59	1.74	ENSG00000266010	GATA6 antisense RNA 1 (head to head)
TTYH1	1.59	5.45	ENSG00000167614	tweety family member 1
TNFRSF11A	1.57	2.15	ENSG00000141655	TNF receptor superfamily member 11a
CXCL12	1.57	7.93	ENSG00000107562	C-X-C motif chemokine ligand 12
DENND6B	1.57	1.19	ENSG00000205593	DENN domain containing 6B
DNAH1	1.56	2.22	ENSG00000114841	dynein axonemal heavy chain 1
PDE5A	1.56	5.80	ENSG00000138735	phosphodiesterase 5A
ADAM8	1.56	3.78	ENSG00000151651	ADAM metallopeptidase domain 8
SOX5	1.55	3.42	ENSG00000134532	SRY-box 5
CCDC71L	1.55	1.94	ENSG00000253276	coiled-coil domain containing 71-like

TCEAL3	1.55	2.37	ENSG00000196507	transcription elongation factor A like 3
MVP	1.54	3.27	ENSG00000013364	major vault protein
ADH6	1.54	3.69	ENSG00000172955	alcohol dehydrogenase 6 (class V)
PTGER4	1.54	4.91	ENSG00000171522	prostaglandin E receptor 4
SUSD5	1.54	2.06	ENSG00000173705	sushi domain containing 5
SPDYE3	1.54	1.49	ENSG00000214300	speedy/RINGO cell cycle regulator family member E3
HLA-F	1.54	1.09	ENSG00000229698	major histocompatibility complex, class I, F
MAP2	1.54	5.80	ENSG00000078018	microtubule associated protein 2
PPP1R3C	1.53	2.48	ENSG00000119938	protein phosphatase 1 regulatory subunit 3C
ALDH8A1	1.52	3.93	ENSG00000118514	aldehyde dehydrogenase 8 family member A1
COL9A2	1.52	6.12	ENSG00000049089	collagen type IX alpha 2 chain
RP1-193H18.2	1.52	1.15	ENSG00000267194	N/A
TMEM59L	1.51	3.60	ENSG00000105696	transmembrane protein 59 like
TMEM151A	1.51	2.65	ENSG00000179292	transmembrane protein 151A
CPM	1.51	3.36	ENSG00000135678	carboxypeptidase M
F2RL1	1.50	4.51	ENSG00000164251	F2R like trypsin receptor 1
MTND1P23	1.50	7.82	ENSG00000225972	mitochondrially encoded NADH:ubiquinone oxidoreductase core subunit 1 pseudogene 23
ENO2	1.50	3.15	ENSG00000111674	enolase 2
TAP1	1.50	2.84	ENSG00000232367	transporter 1, ATP binding cassette subfamily B member
ABTB2	1.50	3.63	ENSG00000166016	ankyrin repeat and BTB domain containing 2
RP5-1085F17.3	1.48	3.46	ENSG00000260257	N/A
RFX6	1.48	2.93	ENSG00000185002	regulatory factor X6
SLC27A1	1.48	2.09	ENSG00000130304	solute carrier family 27 member 1
DZIP1L	1.47	4.73	ENSG00000158163	DAZ interacting zinc finger protein 1 like
FHOD3	1.47	3.95	ENSG00000134775	formin homology 2 domain containing 3
EBLN2	1.46	1.84	ENSG00000255423	endogenous Bornavirus-like nucleoprotein 2
SLFN13	1.46	1.84	ENSG00000154760	schlafen family member 13

ATP10D	1.46	5.19	ENSG00000145246	ATPase phospholipid transporting 10D (putative)
NPY1R	1.46	1.49	ENSG00000164128	neuropeptide Y receptor Y1
IQCK	1.45	4.52	ENSG00000174628	IQ motif containing K
CACNB3	1.45	2.95	ENSG00000167535	calcium voltage-gated channel auxiliary subunit beta 3
HLA-C	1.45	6.17	ENSG00000204525	major histocompatibility complex, class I, C
HS3ST1	1.44	3.04	ENSG00000002587	heparan sulfate-glucosamine 3-sulfotransferase 1
WDR54	1.44	3.23	ENSG00000005448	WD repeat domain 54
LAMB3	1.44	1.30	ENSG00000196878	laminin subunit beta 3
RIMKLA	1.43	1.41	ENSG00000177181	ribosomal modification protein rimK like family member A
PARM1	1.43	2.44	ENSG00000169116	prostate androgen-regulated mucin-like protein 1
SLCO2A1	1.43	2.44	ENSG00000174640	solute carrier organic anion transporter family member 2A1
DISP2	1.42	1.01	ENSG00000140323	dispatched RND transporter family member 2
FBP1	1.42	1.71	ENSG00000165140	fructose-bisphosphatase 1
IL32	1.42	7.72	ENSG00000008517	interleukin 32
RP11-620J15.3	1.41	1.56	ENSG00000257698	N/A
MAML2	1.41	3.01	ENSG00000184384	mastermind like transcriptional coactivator 2
CTC-297N7.9	1.40	1.07	ENSG00000264016	N/A
SIRPA	1.40	3.99	ENSG00000198053	signal regulatory protein alpha
ZNF571-AS1	1.40	3.20	ENSG00000267470	ZNF571 antisense RNA 1
LGR5	1.40	6.48	ENSG00000139292	leucine rich repeat containing G protein-coupled receptor 5
PRRT2	1.39	2.09	ENSG00000167371	proline rich transmembrane protein 2
SCN1B	1.39	1.82	ENSG00000105711	sodium voltage-gated channel beta subunit 1
RFTN2	1.38	1.38	ENSG00000162944	raftlin family member 2
ZNF560	1.38	2.05	ENSG00000198028	zinc finger protein 560
P2RX4	1.38	4.22	ENSG00000135124	purinergic receptor P2X 4
ETS1	1.37	1.05	ENSG00000134954	ETS proto-oncogene 1, transcription factor

MRAP2	1.37	1.05	ENSG00000135324	melanocortin 2 receptor accessory protein 2
NYAP1	1.37	1.19	ENSG00000166924	neuronal tyrosine phosphorylated phosphoinositide-3-kinase adaptor 1
CBX7	1.37	1.31	ENSG00000100307	chromobox 7
COLCA2	1.37	3.58	ENSG00000214290	colorectal cancer associated 2
FZD9	1.37	3.10	ENSG00000188763	frizzled class receptor 9
B3GALT1	1.37	4.90	ENSG00000172318	beta-1,3-galactosyltransferase 1
JAK1	1.36	7.71	ENSG00000162434	Janus kinase 1
TLR2	1.35	2.71	ENSG00000137462	toll like receptor 2
CAPS	1.34	4.52	ENSG00000105519	calcyphosine
LRRC23	1.34	1.84	ENSG00000010626	leucine rich repeat containing 23
HLA-A	1.34	8.38	ENSG00000224320	major histocompatibility complex, class I, A
ANXA6	1.33	3.53	ENSG00000197043	annexin A6
EXTL3	1.33	7.57	ENSG00000012232	exostosin like glycosyltransferase 3
SNAP25	1.33	4.19	ENSG00000132639	synaptosome associated protein 25
IFIH1	1.33	4.55	ENSG00000115267	interferon induced with helicase C domain 1
NPHP1	1.32	1.74	ENSG00000144061	nephrocystin 1
KYNU	1.32	1.94	ENSG00000115919	kynureninase
DUSP10	1.32	5.12	ENSG00000143507	dual specificity phosphatase 10
ADAMTS10	1.31	2.91	ENSG00000142303	ADAM metallopeptidase with thrombospondin type 1 motif 10
CEBPD	1.31	6.41	ENSG00000221869	CCAAT/enhancer binding protein delta
RASSF4	1.31	5.71	ENSG00000107551	Ras association domain family member 4
NYNRIN	1.31	2.91	ENSG00000205978	NYN domain and retroviral integrase containing
ANO9	1.30	5.55	ENSG00000185101	anoctamin 9
ANG	1.30	1.07	ENSG00000214274	angiogenin
ALPK1	1.29	3.29	ENSG00000073331	alpha kinase 1
THBS3	1.29	3.00	ENSG00000169231	thrombospondin 3
OAS3	1.29	3.15	ENSG00000111331	2'-5'-oligoadenylate synthetase 3
GPR161	1.28	5.29	ENSG00000143147	G protein-coupled receptor 161
TNIP1	1.27	6.66	ENSG00000145901	TNFAIP3 interacting protein 1
TRIM50	1.27	2.65	ENSG00000146755	tripartite motif containing 50

CSGALNACT1	1.27	3.59	ENSG00000147408	chondroitin sulfate N-acetylgalactosaminyltransferase 1
EYA1	1.27	1.05	ENSG00000104313	EYA transcriptional coactivator and phosphatase 1
FMO5	1.27	2.99	ENSG00000131781	flavin containing monooxygenase 5
LRRC27	1.26	1.30	ENSG00000148814	leucine rich repeat containing 27
PBXIP1	1.26	6.48	ENSG00000163346	PBX homeobox interacting protein 1
PTPRD	1.26	2.55	ENSG00000282932	protein tyrosine phosphatase, receptor type D
RP1-257A7.4	1.25	1.50	ENSG00000215022	N/A
NFKBIA	1.25	7.72	ENSG00000100906	NFKB inhibitor alpha
RP11-486A14.1	1.25	1.35	ENSG00000257512	N/A
STK39	1.25	6.21	ENSG00000198648	serine/threonine kinase 39
ZNF608	1.25	4.94	ENSG00000168916	zinc finger protein 608
MSRB3	1.25	2.74	ENSG00000174099	methionine sulfoxide reductase B3
TAGLN	1.25	4.80	ENSG00000149591	transgelin
TCTN2	1.25	4.35	ENSG00000168778	tectonic family member 2
TSPAN15	1.24	5.61	ENSG00000099282	tetraspanin 15
CX3CL1	1.24	7.23	ENSG00000006210	C-X3-C motif chemokine ligand 1
ARHGEF40	1.24	5.43	ENSG00000165801	Rho guanine nucleotide exchange factor 40
CDKN1A	1.24	2.44	ENSG00000124762	cyclin dependent kinase inhibitor 1A
PGM2L1	1.24	4.31	ENSG00000165434	phosphoglucomutase 2 like 1
TGFB2	1.23	7.47	ENSG00000092969	transforming growth factor beta 2
ZFP36L1	1.23	8.43	ENSG00000185650	ZFP36 ring finger protein like 1
AKT3	1.23	2.21	ENSG00000117020	AKT serine/threonine kinase 3
GPRC5B	1.22	7.74	ENSG00000167191	G protein-coupled receptor class C group 5 member B
PKLR	1.22	1.15	ENSG00000143627	pyruvate kinase, liver and RBC
RARRES1	1.22	2.47	ENSG00000118849	retinoic acid receptor responder 1
SLC8B1	1.22	3.43	ENSG00000089060	solute carrier family 8 member B1
SH3BGRL	1.21	4.55	ENSG00000131171	SH3 domain binding glutamate rich protein like
NPR1	1.21	1.43	ENSG00000169418	natriuretic peptide receptor 1
ETV6	1.21	4.67	ENSG00000139083	ETS variant 6
RASSF5	1.21	3.49	ENSG00000266094	Ras association domain family member 5

TMEM51	1.21	5.54	ENSG00000171729	transmembrane protein 51
PTK2B	1.21	1.82	ENSG00000120899	protein tyrosine kinase 2 beta
GDNF	1.21	4.28	ENSG00000168621	glial cell derived neurotrophic factor
MYL9	1.21	4.85	ENSG00000101335	myosin light chain 9
LRRC49	1.21	3.31	ENSG00000137821	leucine rich repeat containing 49
FAM102A	1.21	4.69	ENSG00000167106	family with sequence similarity 102 member A
LRRN1	1.20	4.86	ENSG00000175928	leucine rich repeat neuronal 1
SLC5A12	1.20	1.70	ENSG00000148942	solute carrier family 5 member 12
GABRA2	1.20	4.18	ENSG00000151834	gamma-aminobutyric acid type A receptor alpha2 subunit
ITGA3	1.20	2.16	ENSG00000005884	integrin subunit alpha 3
SEMA3G	1.20	3.65	ENSG00000010319	semaphorin 3G
LINC00174	1.20	1.31	ENSG00000179406	long intergenic non-protein coding RNA 174
TNFRSF19	1.20	5.74	ENSG00000127863	TNF receptor superfamily member 19
GATA6	1.20	5.75	ENSG00000141448	GATA binding protein 6
CTHRC1	1.20	5.33	ENSG00000164932	collagen triple helix repeat containing 1
SEMA6A	1.20	6.01	ENSG00000092421	semaphorin 6A
GPR176	1.19	3.02	ENSG00000166073	G protein-coupled receptor 176
C10orf11	1.19	2.38	ENSG00000148655	chromosome 10 open reading frame 11
IFITM3	1.19	8.46	ENSG00000142089	interferon induced transmembrane protein 3
BTG1	1.19	6.29	ENSG00000133639	BTG anti-proliferation factor 1
MR1	1.19	3.81	ENSG00000153029	major histocompatibility complex, class I-related
EPDR1	1.19	4.57	ENSG00000086289	ependymin related 1
CEACAM1	1.19	5.80	ENSG00000079385	carcinoembryonic antigen related cell adhesion molecule 1
FAM214B	1.18	3.38	ENSG00000005238	family with sequence similarity 214 member B
BTN3A3	1.18	3.47	ENSG00000111801	butyrophilin subfamily 3 member A3
C6orf163	1.18	1.24	ENSG00000203872	chromosome 6 open reading frame 163
A4GALT	1.18	3.74	ENSG00000128274	alpha 1,4-galactosyltransferase
GS1-358P8.4	1.18	1.55	ENSG00000260822	N/A
DYRK3	1.18	1.30	ENSG00000143479	dual specificity tyrosine phosphorylation regulated kinase 3

ADAMTS6	1.17	1.98	ENSG00000049192	ADAM metallopeptidase with thrombospondin type 1 motif 6
ABR	1.17	6.73	ENSG00000278741	active BCR-related
QRICH2	1.16	3.34	ENSG00000129646	glutamine rich 2
NKD1	1.16	5.19	ENSG00000140807	naked cuticle homolog 1
SPATA7	1.15	1.22	ENSG00000042317	spermatogenesis associated 7
NFIX	1.15	6.93	ENSG00000008441	nuclear factor I X
DDR1	1.15	6.40	ENSG00000230456	discoidin domain receptor tyrosine kinase 1
HUNK	1.15	4.93	ENSG00000142149	hormonally up-regulated Neu-associated kinase
AKAP6	1.15	2.12	ENSG00000151320	A-kinase anchoring protein 6
COL9A3	1.14	7.73	ENSG00000092758	collagen type IX alpha 3 chain
SLC12A2	1.14	9.14	ENSG00000064651	solute carrier family 12 member 2
CCDC28B	1.14	3.59	ENSG00000160050	coiled-coil domain containing 28B
CXXC4	1.14	1.03	ENSG00000168772	CXXC finger protein 4
MAPRE3	1.13	2.81	ENSG00000084764	microtubule associated protein RP/EB family member 3
EHD3	1.13	2.17	ENSG00000013016	EH domain containing 3
MSH5	1.13	1.21	ENSG00000235569	mutS homolog 5
TBC1D32	1.12	2.44	ENSG00000146350	TBC1 domain family member 32
ZNF836	1.12	1.64	ENSG00000196267	zinc finger protein 836
CYB5R2	1.12	1.68	ENSG00000166394	cytochrome b5 reductase 2
DKK3	1.12	3.50	ENSG00000050165	dickkopf WNT signaling pathway inhibitor 3
TMEM45A	1.12	4.10	ENSG00000181458	transmembrane protein 45A
PGC	1.12	4.41	ENSG00000096088	progastricsin
CXCL5	1.12	9.37	ENSG00000163735	C-X-C motif chemokine ligand 5
TRIM31	1.12	2.36	ENSG00000226402	tripartite motif containing 31
RP9P	1.12	2.77	ENSG00000205763	retinitis pigmentosa 9 pseudogene
ABCA11P	1.12	1.36	ENSG00000251595	ATP binding cassette subfamily A member 11, pseudogene
PRICKLE2	1.11	4.14	ENSG00000163637	prickle planar cell polarity protein 2
MORN4	1.11	1.46	ENSG00000171160	MORN repeat containing 4
SYT12	1.11	3.31	ENSG00000173227	synaptotagmin 12
RP11-38M8.1	1.10	1.01	ENSG00000273297	N/A
ZNF808	1.10	2.09	ENSG00000198482	zinc finger protein 808

SSBP3	1.10	6.33	ENSG00000157216	single stranded DNA binding protein 3
OR2I1P	1.10	6.37	ENSG00000231973	olfactory receptor family 2 subfamily I member 1 pseudogene
FREM1	1.10	1.19	ENSG00000164946	FRAS1 related extracellular matrix 1
PCAT6	1.10	1.19	ENSG00000228288	prostate cancer associated transcript 6 (non-protein coding)
ATP1A1	1.10	9.10	ENSG00000163399	ATPase Na+/K+ transporting subunit alpha 1
LGALS1	1.10	7.50	ENSG00000100097	galectin 1
PCDHB10	1.09	1.24	ENSG00000120324	protocadherin beta 10
IGFBP3	1.09	7.98	ENSG00000146674	insulin like growth factor binding protein 3
RASL11B	1.09	1.93	ENSG00000128045	RAS like family 11 member B
SEMA6C	1.09	4.20	ENSG00000143434	semaphorin 6C
RFTN1	1.09	2.97	ENSG00000131378	raftlin, lipid raft linker 1
ZNF391	1.09	3.20	ENSG00000124613	zinc finger protein 391
CKAP4	1.09	8.13	ENSG00000136026	cytoskeleton associated protein 4
EMB	1.09	3.06	ENSG00000170571	embigin
TMOD1	1.09	1.49	ENSG00000136842	tropomodulin 1
LITAF	1.08	6.37	ENSG00000189067	lipopolysaccharide induced TNF factor
C2CD4C	1.08	2.17	ENSG00000183186	C2 calcium dependent domain containing 4C
CCDC53	1.08	2.63	ENSG00000120860	coiled-coil domain containing 53
SLC1A4	1.08	6.93	ENSG00000115902	solute carrier family 1 member 4
SOCS2	1.08	2.30	ENSG00000120833	suppressor of cytokine signaling 2
CYP1B1	1.08	1.74	ENSG00000138061	cytochrome P450 family 1 subfamily B member 1
CTSC	1.07	9.20	ENSG00000109861	cathepsin C
HLA-G	1.07	2.63	ENSG00000235680	major histocompatibility complex, class I, G
HERC6	1.07	1.05	ENSG00000138642	HECT and RLD domain containing E3 ubiquitin protein ligase family member 6
TJP3	1.07	3.76	ENSG00000105289	tight junction protein 3
CBX6	1.07	6.42	ENSG00000183741	chromobox 6
PIK3CD	1.07	3.78	ENSG00000171608	phosphatidylinositol-4,5-bisphosphate 3-kinase catalytic subunit delta
IL17RB	1.07	7.10	ENSG00000056736	interleukin 17 receptor B

RP11-244H3.1	1.07	1.17	ENSG00000241014	N/A
NPIPP1	1.07	1.28	ENSG00000188599	nuclear pore complex interacting protein pseudogene 1
RNF103	1.07	5.75	ENSG00000239305	ring finger protein 103
BTN2A2	1.06	4.92	ENSG00000124508	butyrophilin subfamily 2 member A2
DLK1	1.06	3.08	ENSG00000185559	delta like non-canonical Notch ligand 1
PRAME	1.06	1.43	ENSG00000185686	preferentially expressed antigen in melanoma
YJEFN3	1.06	1.43	ENSG00000250067	YjeF N-terminal domain containing 3
C8orf48	1.06	1.47	ENSG00000164743	chromosome 8 open reading frame 48
ENTPD2	1.06	3.34	ENSG00000054179	ectonucleoside triphosphate diphosphohydrolase 2
ALPL	1.06	1.68	ENSG00000162551	alkaline phosphatase, liver/bone/kidney
SAMD9	1.06	1.72	ENSG00000205413	sterile alpha motif domain containing 9
PLEKHA4	1.06	4.30	ENSG00000105559	pleckstrin homology domain containing A4
FLRT2	1.06	7.88	ENSG00000185070	fibronectin leucine rich transmembrane protein 2
MOB3B	1.05	4.29	ENSG00000120162	MOB kinase activator 3B
RP11-890B15.3	1.05	2.41	ENSG00000255455	N/A
AGPAT4	1.05	2.79	ENSG00000026652	1-acylglycerol-3-phosphate O-acyltransferase 4
RAB13	1.05	4.60	ENSG00000143545	RAB13, member RAS oncogene family
MBOAT1	1.05	3.04	ENSG00000172197	membrane bound O-acyltransferase domain containing 1
LPAR6	1.04	2.72	ENSG00000139679	lysophosphatidic acid receptor 6
TP53TG1	1.04	2.58	ENSG00000182165	TP53 target 1 (non-protein coding)
DMRTA1	1.04	2.23	ENSG00000176399	DMRT like family A1
IKBKE	1.04	5.89	ENSG00000263528	inhibitor of kappa light polypeptide gene enhancer in B-cells, kinase epsilon
GRIK4	1.04	1.46	ENSG00000149403	glutamate ionotropic receptor kainate type subunit 4
MAP3K7CL	1.04	1.46	ENSG00000156265	MAP3K7 C-terminal like
HNRNPCP1	1.04	1.41	ENSG00000258900	heterogeneous nuclear ribonucleoprotein C pseudogene 1
CCDC157	1.04	1.21	ENSG00000187860	coiled-coil domain containing 157
BTN3A1	1.04	4.91	ENSG00000026950	butyrophilin subfamily 3 member A1

NUDT4	1.04	6.59	ENSG00000173598	nudix hydrolase 4
ZSWIM4	1.03	4.92	ENSG00000132003	zinc finger SWIM-type containing 4
STAT1	1.03	7.83	ENSG00000115415	signal transducer and activator of transcription 1
TM4SF4	1.03	8.01	ENSG00000169903	transmembrane 4 L six family member 4
ELK3	1.03	4.92	ENSG00000111145	ELK3, ETS transcription factor
DSE	1.03	7.10	ENSG00000111817	dermatan sulfate epimerase
ZNF287	1.02	2.01	ENSG00000141040	zinc finger protein 287
TMEM86A	1.02	1.88	ENSG00000151117	transmembrane protein 86A
RP11-448G15.3	1.02	1.77	ENSG00000261490	N/A
RTN2	1.02	3.77	ENSG00000125744	reticulon 2
APBA1	1.02	3.06	ENSG00000276497	amyloid beta precursor protein binding family A member 1
EFCAB12	1.02	1.62	ENSG00000172771	EF-hand calcium binding domain 12
CTD-2545H1.2	1.02	1.49	ENSG00000262445	N/A
SYT1	1.01	1.44	ENSG00000067715	synaptotagmin 1
BIRC2	1.01	6.72	ENSG00000110330	baculoviral IAP repeat containing 2
C7orf31	1.01	4.64	ENSG00000153790	chromosome 7 open reading frame 31
ZNF214	1.01	1.39	ENSG00000149050	zinc finger protein 214
RP11-101E13.5	1.01	3.16	ENSG00000272579	N/A
SGCB	1.01	4.85	ENSG00000163069	sarcoglycan beta
AC007228.9	1.01	1.24	ENSG00000268568	N/A
FZD6	1.01	4.56	ENSG00000164930	frizzled class receptor 6
UGT2B7	1.01	4.39	ENSG00000171234	UDP glucuronosyltransferase family 2 member B7
CD83	1.01	4.84	ENSG00000112149	CD83 molecule
RGMB-AS1	1.01	1.07	ENSG00000246763	RGMB antisense RNA 1
DGKA	1.00	3.61	ENSG00000065357	diacylglycerol kinase alpha
ST6GALNAC3	1.00	1.01	ENSG00000184005	ST6 N-acetylgalactosaminide alpha-2,6-sialyltransferase 3
SLFN5	1.00	5.05	ENSG00000166750	schlafen family member 5
RP11-4O1.2	1.00	2.38	ENSG00000259953	N/A
LRRC1	1.00	5.98	ENSG00000137269	leucine rich repeat containing 1
S100A6	1.00	5.57	ENSG00000197956	S100 calcium binding protein A6
GDPD5	1.00	4.07	ENSG00000158555	glycerophosphodiester phosphodiesterase domain containing 5
TFAP2A	1.00	6.57	ENSG00000137203	transcription factor AP-2 alpha

DAPK2	1.00	2.25	ENSG00000035664	death associated protein kinase 2
SLC37A1	1.00	4.72	ENSG00000160190	solute carrier family 37 member 1
MYH3	1.00	3.08	ENSG00000109063	myosin heavy chain 3
FBXO36	0.99	2.08	ENSG00000153832	F-box protein 36
FAM229B	0.99	2.47	ENSG00000203778	family with sequence similarity 229 member B
KCTD1	0.99	3.02	ENSG00000134504	potassium channel tetramerization domain containing 1
CASC15	0.99	2.44	ENSG00000272168	cancer susceptibility candidate 15 (non-protein coding)
GOLM1	0.99	3.33	ENSG00000135052	golgi membrane protein 1
S100A4	0.99	1.43	ENSG00000196154	S100 calcium binding protein A4
VNN2	0.99	3.45	ENSG00000112303	vanin 2
NBPF20	0.99	1.93	ENSG00000162825	neuroblastoma breakpoint family member 20
PDK2	0.99	3.84	ENSG00000005882	pyruvate dehydrogenase kinase 2
HPSE	0.99	2.59	ENSG00000173083	heparanase
LURAP1L	0.99	3.54	ENSG00000153714	leucine rich adaptor protein 1 like
CEACAM19	0.99	1.33	ENSG00000186567	carcinoembryonic antigen related cell adhesion molecule 19
LMLN	0.99	3.00	ENSG00000185621	leishmanolysin like peptidase
BTN3A2	0.98	4.73	ENSG00000186470	butyrophilin subfamily 3 member A2
CNTNAP1	0.98	4.77	ENSG00000108797	contactin associated protein 1
IRF5	0.98	1.22	ENSG00000128604	interferon regulatory factor 5
UGT3A2	0.98	3.53	ENSG00000168671	UDP glycosyltransferase family 3 member A2
LMCD1	0.98	5.25	ENSG00000071282	LIM and cysteine rich domains 1
BCL2L11	0.98	5.40	ENSG00000153094	BCL2 like 11
VRK2	0.98	5.87	ENSG00000028116	vaccinia related kinase 2
MPPED2	0.98	3.18	ENSG00000066382	metallophosphoesterase domain containing 2
NUCB2	0.98	5.00	ENSG00000070081	nucleobindin 2
PSENEN	0.98	5.18	ENSG00000205155	presenilin enhancer gamma-secretase subunit
GATS	0.97	3.08	ENSG00000239521	GATS, stromal antigen 3 opposite strand
GFRA3	0.97	1.95	ENSG00000146013	GDNF family receptor alpha 3
SQRDL	0.97	3.79	ENSG00000137767	sulfide quinone reductase-like (yeast)

C8orf49	0.97	1.55	ENSG00000255394	chromosome 8 open reading frame 49
ZSWIM5	0.97	3.68	ENSG00000162415	zinc finger SWIM-type containing 5
RP11-278L15.6	0.97	1.85	ENSG00000244503	N/A
RRAS	0.97	4.62	ENSG00000126458	related RAS viral (r-ras) oncogene homolog
SERPINE2	0.97	5.98	ENSG00000135919	serpin family E member 2
PARP14	0.97	4.94	ENSG00000173193	poly(ADP-ribose) polymerase family member 14
MPP1	0.97	3.74	ENSG00000130830	membrane palmitoylated protein 1
C14orf105	0.97	3.97	ENSG00000100557	chromosome 14 open reading frame 105
SLC18B1	0.96	6.94	ENSG00000146409	solute carrier family 18 member B1
FAM89B	0.96	4.75	ENSG00000176973	family with sequence similarity 89 member B
RTN4RL1	0.96	4.51	ENSG00000185924	reticulon 4 receptor like 1
HAS3	0.96	2.28	ENSG00000103044	hyaluronan synthase 3
ACSF2	0.96	3.97	ENSG00000167107	acyl-CoA synthetase family member 2
RP11-403I13.8	0.96	2.96	ENSG00000272419	N/A
RP11-479G22.8	0.96	1.70	ENSG00000273038	N/A
NCEH1	0.96	6.36	ENSG00000144959	neutral cholesterol ester hydrolase 1
CTSF	0.96	3.68	ENSG00000174080	cathepsin F
NFE2L3	0.96	6.99	ENSG00000050344	nuclear factor, erythroid 2 like 3
LIMCH1	0.96	4.72	ENSG00000064042	LIM and calponin homology domains 1
ARHGAP40	0.96	2.74	ENSG00000124143	Rho GTPase activating protein 40
FAXC	0.95	3.99	ENSG00000146267	failed axon connections homolog
CBWD5	0.95	1.21	ENSG00000147996	COBW domain containing 5
RAB42	0.95	1.21	ENSG00000188060	RAB42, member RAS oncogene family
RP11-5C23.1	0.95	1.21	ENSG00000272760	N/A
C11orf49	0.95	5.63	ENSG00000149179	chromosome 11 open reading frame 49
CCL15	0.95	1.57	ENSG00000275718	C-C motif chemokine ligand 15
TLR3	0.95	1.57	ENSG00000164342	toll like receptor 3
NEBL	0.95	2.94	ENSG00000078114	nebulette
RP11-977G19.5	0.95	1.53	ENSG00000258199	N/A
CROT	0.95	3.27	ENSG00000005469	carnitine O-octanoyltransferase
FNDC4	0.95	2.91	ENSG00000115226	fibronectin type III domain containing 4
ARL3	0.95	3.89	ENSG00000138175	ADP ribosylation factor like GTPase 3
SLC30A3	0.95	2.62	ENSG00000115194	solute carrier family 30 member 3
METTL7A	0.95	3.24	ENSG00000185432	methyltransferase like 7A

PHLDA1	0.94	7.82	ENSG00000139289	pleckstrin homology like domain family A member 1
SNX10	0.94	4.54	ENSG00000086300	sorting nexin 10
VAMP2	0.94	4.60	ENSG00000220205	vesicle associated membrane protein 2
SERPINA10	0.94	3.03	ENSG00000278767	serpin family A member 10
TRAM2-AS1	0.94	1.39	ENSG00000225791	TRAM2 antisense RNA 1 (head to head)
TUBB3	0.94	7.00	ENSG00000258947	tubulin beta 3 class III
HMSD	0.94	2.64	ENSG00000221887	histocompatibility minor serpin domain containing
TRIM52-AS1	0.94	2.47	ENSG00000248275	TRIM52 antisense RNA 1 (head to head)
NFKB2	0.94	6.72	ENSG00000077150	nuclear factor kappa B subunit 2
MFAP2	0.93	7.07	ENSG00000117122	microfibrillar associated protein 2
SLC41A2	0.93	3.32	ENSG00000136052	solute carrier family 41 member 2
NRP2	0.93	3.11	ENSG00000118257	neuropilin 2
UBD	0.93	2.53	ENSG00000224654	ubiquitin D
CDC42EP4	0.93	8.07	ENSG00000179604	CDC42 effector protein 4
PBX3	0.93	3.50	ENSG00000167081	PBX homeobox 3
FCHSD1	0.93	3.27	ENSG00000197948	FCH and double SH3 domains 1
FHDC1	0.93	4.99	ENSG00000137460	FH2 domain containing 1
GJC1	0.93	7.56	ENSG00000182963	gap junction protein gamma 1
RASSF6	0.93	1.24	ENSG00000169435	Ras association domain family member 6
ZNF582-AS1	0.93	1.24	ENSG00000267454	ZNF582 antisense RNA 1 (head to head)
CRIP1	0.93	2.47	ENSG00000213145	cysteine rich protein 1
NBL1	0.92	2.59	ENSG00000158747	neuroblastoma 1, DAN family BMP antagonist
SP100	0.92	4.72	ENSG00000067066	SP100 nuclear antigen
CD58	0.92	3.47	ENSG00000116815	CD58 molecule
FAM114A1	0.92	5.40	ENSG00000197712	family with sequence similarity 114 member A1
DNAH5	0.92	1.91	ENSG00000039139	dynein axonemal heavy chain 5
RP11-418J17.1	0.92	2.09	ENSG00000231365	N/A
GARNL3	0.92	2.64	ENSG00000136895	GTPase activating Rap/RanGAP domain like 3
RP11-465N4.5	0.92	1.13	ENSG00000273478	N/A
KIF3C	0.92	4.97	ENSG00000084731	kinesin family member 3C
CD74	0.92	5.80	ENSG00000019582	CD74 molecule

BBS12	0.91	1.38	ENSG00000181004	Bardet-Biedl syndrome 12
HNF1A-AS1	0.91	1.07	ENSG00000241388	HNF1A antisense RNA 1
RNF24	0.91	4.84	ENSG00000101236	ring finger protein 24
HNF1B	0.91	7.22	ENSG00000275410	HNF1 homeobox B
PINK1-AS	0.91	3.12	ENSG00000117242	PINK1 antisense RNA
MARK1	0.91	3.51	ENSG00000116141	microtubule affinity regulating kinase 1
CDK19	0.91	6.52	ENSG00000155111	cyclin dependent kinase 19
HERPUD2	0.91	5.61	ENSG00000122557	HERPUD family member 2
RRAGC	0.91	4.74	ENSG00000116954	Ras related GTP binding C
RP11-498C9.15	0.91	1.77	ENSG00000263731	N/A
LARP6	0.91	4.26	ENSG00000166173	La ribonucleoprotein domain family member 6
MAFB	0.91	1.01	ENSG00000204103	MAF bZIP transcription factor B
DCAF6	0.91	7.43	ENSG00000143164	DDB1 and CUL4 associated factor 6
BTBD9	0.91	3.98	ENSG00000183826	BTB domain containing 9
TMEM25	0.91	3.15	ENSG00000149582	transmembrane protein 25
RNF19A	0.91	6.51	ENSG00000034677	ring finger protein 19A, RBR E3 ubiquitin protein ligase
CD47	0.91	6.28	ENSG00000196776	CD47 molecule
ERBB4	0.91	3.21	ENSG00000178568	erb-b2 receptor tyrosine kinase 4
FOXQ1	0.90	4.93	ENSG00000164379	forkhead box Q1
TRIM69	0.90	4.15	ENSG00000278211	tripartite motif containing 69
CLVS1	0.90	2.22	ENSG00000177182	clavesin 1
CA5B	0.90	3.56	ENSG00000169239	carbonic anhydrase 5B
SPATA13	0.90	5.98	ENSG00000182957	spermatogenesis associated 13
TRIM59	0.90	2.45	ENSG00000213186	tripartite motif containing 59
MICB	0.90	5.01	ENSG00000231372	MHC class I polypeptide-related sequence B
GABBR1	0.90	1.70	ENSG00000237112	gamma-aminobutyric acid type B receptor subunit 1
CD99	0.90	6.80	ENSG00000002586	CD99 molecule
REEP2	0.90	1.46	ENSG00000132563	receptor accessory protein 2
UACA	0.90	6.37	ENSG00000137831	uveal autoantigen with coiled-coil domains and ankyrin repeats
CYP27A1	0.90	5.73	ENSG00000135929	cytochrome P450 family 27 subfamily A member 1
SOBP	0.89	6.97	ENSG00000112320	sine oculis binding protein homolog

PQLC3	0.89	2.26	ENSG00000162976	PQ loop repeat containing 3
IER3	0.89	8.26	ENSG00000230128	immediate early response 3
NOD1	0.89	1.41	ENSG00000106100	nucleotide binding oligomerization domain containing 1
SNX22	0.89	3.27	ENSG00000157734	sorting nexin 22
SCPEP1	0.89	3.96	ENSG00000121064	serine carboxypeptidase 1
LRRC46	0.89	1.36	ENSG00000141294	leucine rich repeat containing 46
SLC34A2	0.89	4.02	ENSG00000157765	solute carrier family 34 member 2
RAB36	0.89	1.11	ENSG00000100228	RAB36, member RAS oncogene family
RP11-757F18.5	0.89	1.11	ENSG00000261488	N/A
DPYSL2	0.89	5.79	ENSG00000092964	dihydropyrimidinase like 2
GPRASP1	0.89	2.18	ENSG00000198932	G protein-coupled receptor associated sorting protein 1
PRLR	0.88	4.31	ENSG00000113494	prolactin receptor
MORN2	0.88	2.28	ENSG00000188010	MORN repeat containing 2
PLA2G4C	0.88	3.79	ENSG00000105499	phospholipase A2 group IVC
IGIP	0.88	1.31	ENSG00000182700	IgA inducing protein
COLGALT2	0.88	2.37	ENSG00000198756	collagen beta(1-O)galactosyltransferase 2
VMAC	0.88	2.97	ENSG00000187650	vimentin-type intermediate filament associated coiled-coil protein
ADAP2	0.88	1.86	ENSG00000184060	ArfGAP with dual PH domains 2
SLC11A2	0.88	6.17	ENSG00000110911	solute carrier family 11 member 2
SSTR1	0.88	2.12	ENSG00000139874	somatostatin receptor 1
ZNF577	0.88	2.73	ENSG00000161551	zinc finger protein 577
SH3BP5-AS1	0.88	2.96	ENSG00000224660	SH3BP5 antisense RNA 1
ZFHX2	0.88	5.33	ENSG00000136367	zinc finger homeobox 2
PITPNC1	0.88	4.16	ENSG00000154217	phosphatidylinositol transfer protein, cytoplasmic 1
TSPAN7	0.88	1.82	ENSG00000156298	tetraspanin 7
SRCIN1	0.88	4.45	ENSG00000277363	SRC kinase signaling inhibitor 1
ARMC2	0.88	1.26	ENSG00000118690	armadillo repeat containing 2
OAF	0.87	6.89	ENSG00000184232	out at first homolog
RP11-159D12.2	0.87	4.88	ENSG00000264112	N/A
PRAP1	0.87	4.34	ENSG00000165828	proline rich acidic protein 1
CA14	0.87	2.17	ENSG00000118298	carbonic anhydrase 14

KCNIP4	0.87	2.27	ENSG00000185774	potassium voltage-gated channel interacting protein 4
ISL1	0.87	3.35	ENSG00000016082	ISL LIM homeobox 1
CFB	0.87	3.62	ENSG00000243649	complement factor B
TESC	0.87	6.40	ENSG00000088992	tescalcin
GKAP1	0.87	1.39	ENSG00000165113	G kinase anchoring protein 1
SPATA6	0.86	3.09	ENSG00000132122	spermatogenesis associated 6
RP11-283I3.6	0.86	3.35	ENSG00000261799	N/A
KRBA1	0.86	4.13	ENSG00000133619	KRAB-A domain containing 1
PARP10	0.86	1.71	ENSG00000178685	poly(ADP-ribose) polymerase family member 10
SLC2A8	0.86	6.27	ENSG00000136856	solute carrier family 2 member 8
FUCA1	0.86	4.23	ENSG00000179163	fucosidase, alpha-L- 1, tissue
DCLK2	0.86	1.15	ENSG00000170390	doublecortin like kinase 2
RP11-452F19.3	0.86	2.31	ENSG00000228106	N/A
SORD	0.86	5.67	ENSG00000140263	sorbitol dehydrogenase
CREB3L2	0.86	6.69	ENSG00000182158	cAMP responsive element binding protein 3 like 2
APBB1	0.86	4.65	ENSG00000166313	amyloid beta precursor protein binding family B member 1
HECA	0.86	3.68	ENSG00000112406	hdc homolog, cell cycle regulator
TMA7	0.86	2.49	ENSG00000232112	translation machinery associated 7 homolog
STAT5A	0.86	3.75	ENSG00000126561	signal transducer and activator of transcription 5A
LGALS3	0.86	3.17	ENSG00000131981	galectin 3
POU6F1	0.86	2.19	ENSG00000184271	POU class 6 homeobox 1
CD59	0.86	8.13	ENSG00000085063	CD59 molecule
MYLIP	0.86	2.29	ENSG00000007944	myosin regulatory light chain interacting protein
NDRG1	0.86	6.22	ENSG00000104419	N-myc downstream regulated 1
CREB3L3	0.86	1.67	ENSG00000060566	cAMP responsive element binding protein 3 like 3
RP11-420L9.5	0.86	1.67	ENSG00000270504	N/A
RP11-755F10.1	0.86	1.67	ENSG00000255320	N/A
RP11-120K24.5	0.86	3.93	ENSG00000269376	N/A

NDUFA13	0.86	3.79	ENSG00000186010	NADH:ubiquinone oxidoreductase subunit A13
GJB2	0.85	2.96	ENSG00000165474	gap junction protein beta 2
EFNB1	0.85	3.13	ENSG00000090776	ephrin B1
BGN	0.85	7.14	ENSG00000182492	biglycan
TBC1D19	0.85	2.88	ENSG00000109680	TBC1 domain family member 19
CAMK2N1	0.85	4.40	ENSG00000162545	calcium/calmodulin dependent protein kinase II inhibitor 1
NRARP	0.85	4.13	ENSG00000198435	NOTCH-regulated ankyrin repeat protein
CLEC16A	0.85	6.49	ENSG00000038532	C-type lectin domain family 16 member A
YPEL3	0.85	5.52	ENSG00000090238	yippee like 3
RERE	0.85	8.16	ENSG00000142599	arginine-glutamic acid dipeptide repeats
NEK11	0.85	2.21	ENSG00000114670	NIMA related kinase 11
STX7	0.85	6.73	ENSG00000079950	syntaxin 7
SEMA3B	0.85	4.09	ENSG00000012171	semaphorin 3B
AC009948.5	0.85	3.07	ENSG00000223960	N/A
BCL3	0.85	6.28	ENSG00000069399	B-cell CLL/lymphoma 3
ZC2HC1A	0.84	3.20	ENSG00000104427	zinc finger C2HC-type containing 1A
ERAP1	0.84	5.34	ENSG00000164307	endoplasmic reticulum aminopeptidase 1
STPG1	0.84	2.79	ENSG00000001460	sperm tail PG-rich repeat containing 1
TRIM68	0.84	2.72	ENSG00000167333	tripartite motif containing 68
ATP9A	0.84	8.04	ENSG00000054793	ATPase phospholipid transporting 9A (putative)
NAGA	0.84	5.32	ENSG00000198951	alpha-N-acetylgalactosaminidase
AGBL3	0.84	2.90	ENSG00000146856	ATP/GTP binding protein like 3
TGFB3	0.84	2.50	ENSG00000119699	transforming growth factor beta 3
ZNF57	0.84	2.50	ENSG00000171970	zinc finger protein 57
SP110	0.84	4.07	ENSG00000135899	SP110 nuclear body protein
HLA-H	0.84	2.12	ENSG00000223463	major histocompatibility complex, class I, H (pseudogene)
ODF2L	0.84	4.59	ENSG00000122417	outer dense fiber of sperm tails 2 like
TOX	0.84	2.68	ENSG00000198846	thymocyte selection associated high mobility group box
NTAN1	0.84	5.75	ENSG00000275779	N-terminal asparagine amidase
SPRY2	0.83	4.43	ENSG00000136158	sprouty RTK signaling antagonist 2

ACTRT3	0.83	3.03	ENSG00000184378	actin related protein T3
ZNF416	0.83	3.03	ENSG00000083817	zinc finger protein 416
PRDM4	0.83	5.76	ENSG00000110851	PR/SET domain 4
RIPPLY3	0.83	1.33	ENSG00000183145	ripply transcriptional repressor 3
LYPD6	0.83	4.73	ENSG00000187123	LY6/PLAUR domain containing 6
ZNF135	0.83	4.85	ENSG00000176293	zinc finger protein 135
SLC2A6	0.83	6.37	ENSG00000281165	solute carrier family 2 member 6
SLC16A12	0.83	1.13	ENSG00000152779	solute carrier family 16 member 12
USP27X	0.83	1.13	ENSG00000273820	ubiquitin specific peptidase 27, X-linked
TRPV4	0.83	5.93	ENSG00000111199	transient receptor potential cation channel subfamily V member 4
FAM174B	0.83	3.53	ENSG00000185442	family with sequence similarity 174 member B
IRF1	0.83	3.59	ENSG00000125347	interferon regulatory factor 1
CALCOCO1	0.83	4.01	ENSG00000012822	calcium binding and coiled-coil domain 1
ZNF362	0.83	5.47	ENSG00000160094	zinc finger protein 362
WDR60	0.83	2.93	ENSG00000126870	WD repeat domain 60
OPTN	0.83	5.93	ENSG00000123240	optineurin
TESK2	0.82	1.57	ENSG00000070759	testis-specific kinase 2
FMO1	0.82	3.25	ENSG00000010932	flavin containing monooxygenase 1
ROBO1	0.82	9.07	ENSG00000169855	roundabout guidance receptor 1
DUSP1	0.82	6.52	ENSG00000120129	dual specificity phosphatase 1
SERPINA6	0.82	5.88	ENSG00000170099	serpin family A member 6
ELF3	0.82	7.00	ENSG00000163435	E74 like ETS transcription factor 3
SLC6A16	0.82	2.85	ENSG00000063127	solute carrier family 6 member 16
CP	0.82	7.48	ENSG00000047457	ceruloplasmin
PLXNA2	0.82	4.35	ENSG00000076356	plexin A2
RP11-1148L6.5	0.82	1.41	ENSG00000228343	N/A
PTGFRN	0.82	6.44	ENSG00000134247	prostaglandin F2 receptor inhibitor
RELB	0.82	5.86	ENSG00000104856	RELB proto-oncogene, NF-kB subunit
PKIG	0.82	3.62	ENSG00000168734	protein kinase (cAMP-dependent, catalytic) inhibitor gamma
KLF6	0.82	8.15	ENSG00000067082	Kruppel like factor 6
C16orf62	0.82	1.78	ENSG00000103544	chromosome 16 open reading frame 62
CYB5RL	0.82	1.78	ENSG00000215883	cytochrome b5 reductase like
RP11-34P13.15	0.82	1.53	ENSG00000268903	N/A

CTC-559E9.5	0.82	1.22	ENSG00000267481	N/A
TTC30A	0.82	2.08	ENSG00000197557	tetratricopeptide repeat domain 30A
SLC38A11	0.82	2.74	ENSG00000169507	solute carrier family 38 member 11
RHPN2	0.82	5.97	ENSG00000131941	rhophilin Rho GTPase binding protein 2
H1F0	0.81	8.43	ENSG00000189060	H1 histone family member 0
KIF1A	0.81	2.16	ENSG00000130294	kinesin family member 1A
STK17B	0.81	3.55	ENSG00000081320	serine/threonine kinase 17b
ACSL1	0.81	5.91	ENSG00000151726	acyl-CoA synthetase long-chain family member 1
PHF21A	0.81	6.06	ENSG00000135365	PHD finger protein 21A
TTLL7	0.81	3.87	ENSG00000137941	tubulin tyrosine ligase like 7
FUT8	0.81	2.60	ENSG00000033170	fucosyltransferase 8
FZD2	0.81	4.84	ENSG00000180340	frizzled class receptor 2
PXDC1	0.81	5.13	ENSG00000168994	PX domain containing 1
MNS1	0.81	2.65	ENSG00000138587	meiosis specific nuclear structural 1
FBN2	0.81	7.25	ENSG00000138829	fibrillin 2
TIMP2	0.81	6.84	ENSG00000035862	TIMP metallopeptidase inhibitor 2
TLDC1	0.81	2.57	ENSG00000140950	TBC/LysM-associated domain containing 1
RP11-706C16.7	0.81	1.60	ENSG00000253196	N/A
OSER1-AS1	0.81	2.63	ENSG00000223891	OSER1 antisense RNA 1 (head to head)
AF131215.9	0.81	2.49	ENSG00000269918	N/A
IL22RA1	0.81	3.54	ENSG00000142677	interleukin 22 receptor subunit alpha 1
ARMC9	0.81	2.90	ENSG00000135931	armadillo repeat containing 9
FAR2	0.81	1.71	ENSG00000064763	fatty acyl-CoA reductase 2
GSTM2	0.80	2.10	ENSG00000213366	glutathione S-transferase mu 2
LINC00641	0.80	4.63	ENSG00000258441	long intergenic non-protein coding RNA 641
ZIK1	0.80	4.09	ENSG00000171649	zinc finger protein interacting with K protein 1
RP11-218M22.1	0.80	1.81	ENSG00000177406	N/A
ZNF883	0.80	3.76	ENSG00000228623	zinc finger protein 883
ZDHHC8	0.80	3.35	ENSG00000099904	zinc finger DHHC-type containing 8
DAZAP2	0.80	7.22	ENSG00000183283	DAZ associated protein 2
CYSTM1	0.80	6.11	ENSG00000120306	cysteine rich transmembrane module containing 1

PTGDS	0.80	3.07	ENSG00000107317	prostaglandin D2 synthase
SORBS2	0.80	4.19	ENSG00000154556	sorbin and SH3 domain containing 2
SFR1	0.80	1.99	ENSG00000156384	SWI5 dependent homologous recombination repair protein 1
CDK18	0.80	4.32	ENSG00000117266	cyclin dependent kinase 18
EFNA1	0.80	6.13	ENSG00000169242	ephrin A1
ICOSLG	0.80	4.32	ENSG00000160223	inducible T-cell costimulator ligand
ZNF703	0.80	5.17	ENSG00000183779	zinc finger protein 703
METTL25	0.79	2.43	ENSG00000127720	methyltransferase like 25
ERN1	0.79	4.98	ENSG00000178607	endoplasmic reticulum to nucleus signaling 1
CLCF1	0.79	4.05	ENSG00000175505	cardiotrophin-like cytokine factor 1
AMZ2	0.79	5.26	ENSG00000196704	archaelysin family metallopeptidase 2
HDX	0.79	2.96	ENSG00000165259	highly divergent homeobox
TNFSF9	0.79	2.66	ENSG00000125657	tumor necrosis factor superfamily member 9
IL1R1	0.79	5.06	ENSG00000115594	interleukin 1 receptor type 1
PAQR7	0.79	2.76	ENSG00000182749	progestin and adipoQ receptor family member 7
ETV1	0.79	4.84	ENSG00000006468	ETS variant 1
BBS9	0.79	3.66	ENSG00000122507	Bardet-Biedl syndrome 9
BRE	0.79	4.52	ENSG00000158019	brain and reproductive organ-expressed (TNFRSF1A modulator)
NBR1	0.79	6.69	ENSG00000188554	NBR1, autophagy cargo receptor
LRRC8C	0.79	2.99	ENSG00000171488	leucine rich repeat containing 8 family member C
DLX4	0.79	1.92	ENSG00000108813	distal-less homeobox 4
RP11-395I6.3	0.79	1.92	ENSG00000260296	N/A
HTATSF1	0.79	5.84	ENSG00000102241	HIV-1 Tat specific factor 1
FGFR1	0.79	6.10	ENSG00000077782	fibroblast growth factor receptor 1
COMMD6	0.79	2.93	ENSG00000188243	COMM domain containing 6
SORBS1	0.79	5.96	ENSG00000095637	sorbin and SH3 domain containing 1
TFPI	0.79	6.10	ENSG00000003436	tissue factor pathway inhibitor
PLEK2	0.79	3.66	ENSG00000100558	pleckstrin 2
MAPK8	0.79	4.89	ENSG00000107643	mitogen-activated protein kinase 8
PANX2	0.79	3.25	ENSG00000073150	pannexin 2
BNIP3L	0.79	4.62	ENSG00000104765	BCL2 interacting protein 3 like

CREB3L4	0.78	3.54	ENSG00000143578	cAMP responsive element binding protein 3 like 4
METTL24	0.78	1.70	ENSG00000053328	methyltransferase like 24
HOMER2	0.78	5.70	ENSG00000103942	homer scaffolding protein 2
SH3RF2	0.78	2.60	ENSG00000156463	SH3 domain containing ring finger 2
MAOB	0.78	7.18	ENSG00000069535	monoamine oxidase B
KIAA1217	0.78	5.40	ENSG00000120549	KIAA1217
VAV3	0.78	5.47	ENSG00000134215	vav guanine nucleotide exchange factor 3
SYNGAP1	0.78	4.16	ENSG00000227460	synaptic Ras GTPase activating protein 1
FLVCR2	0.78	3.53	ENSG00000119686	feline leukemia virus subgroup C cellular receptor family member 2
RP11-67L2.2	0.78	2.19	ENSG00000273033	N/A
PIGZ	0.78	4.13	ENSG00000119227	phosphatidylinositol glycan anchor biosynthesis class Z
RP11-50C13.1	0.78	1.66	ENSG00000259250	N/A
TMSB10P1	0.78	1.66	ENSG00000228499	thymosin beta 10 pseudogene 1
TRIM45	0.78	3.58	ENSG00000134253	tripartite motif containing 45
ZER1	0.78	5.40	ENSG00000160445	zyg-11 related cell cycle regulator
ZNF233	0.78	2.91	ENSG00000159915	zinc finger protein 233
ZNF486	0.77	3.51	ENSG00000256229	zinc finger protein 486
PPP1R15A	0.77	6.79	ENSG00000087074	protein phosphatase 1 regulatory subunit 15A
TGIF1	0.77	5.70	ENSG00000177426	TGFB induced factor homeobox 1
ZNF204P	0.77	2.30	ENSG00000204789	zinc finger protein 204, pseudogene
CTSL	0.77	4.26	ENSG00000135047	cathepsin L
TCF7L1	0.77	7.16	ENSG00000152284	transcription factor 7 like 1
ITGA2	0.77	5.92	ENSG00000164171	integrin subunit alpha 2
CERS5	0.77	4.42	ENSG00000139624	ceramide synthase 5
GLB1L	0.77	1.62	ENSG00000163521	galactosidase beta 1 like
TMCO4	0.77	3.33	ENSG00000162542	transmembrane and coiled-coil domains 4
IQCG	0.77	2.41	ENSG00000114473	IQ motif containing G
PLOD1	0.77	7.87	ENSG00000083444	procollagen-lysine,2-oxoglutarate 5-dioxygenase 1
CRYL1	0.77	2.87	ENSG00000165475	crystallin lambda 1

SMAD3	0.77	6.91	ENSG00000166949	SMAD family member 3
DAB2IP	0.77	7.03	ENSG00000136848	DAB2 interacting protein
NUAK1	0.77	6.24	ENSG00000074590	NUAK family kinase 1
SYTL4	0.77	3.28	ENSG00000102362	synaptotagmin like 4
RP11-120K24.4	0.76	1.09	ENSG00000269356	N/A
JUN	0.76	7.38	ENSG00000177606	Jun proto-oncogene, AP-1 transcription factor subunit
OCA2	0.76	5.21	ENSG00000277361	OCA2 melanosomal transmembrane protein
PTPRH	0.76	3.70	ENSG00000080031	protein tyrosine phosphatase, receptor type H
PLSCR4	0.76	2.66	ENSG00000114698	phospholipid scramblase 4
NFKBIL1	0.76	5.06	ENSG00000236196	NFKB inhibitor like 1
PLEKHB1	0.76	7.21	ENSG00000021300	pleckstrin homology domain containing B1
LRIG3	0.76	5.58	ENSG00000139263	leucine rich repeats and immunoglobulin like domains 3
TMEM178A	0.76	3.58	ENSG00000152154	transmembrane protein 178A
ZCWPW1	0.76	1.57	ENSG00000078487	zinc finger CW-type and PWWP domain containing 1
TMEM81	0.76	2.43	ENSG00000174529	transmembrane protein 81
LIPA	0.76	5.17	ENSG00000107798	lipase A, lysosomal acid type
TANC1	0.76	6.03	ENSG00000115183	tetratricopeptide repeat, ankyrin repeat and coiled-coil containing 1
TSPAN12	0.76	4.41	ENSG00000106025	tetraspanin 12
PELI2	0.76	4.34	ENSG00000139946	pellino E3 ubiquitin protein ligase family member 2
FRMD6	0.76	5.42	ENSG00000139926	FERM domain containing 6
TMPO-AS1	0.76	2.52	ENSG00000257167	TMPO antisense RNA 1
FAM161B	0.76	1.33	ENSG00000156050	family with sequence similarity 161 member B
LIPE	0.76	1.33	ENSG00000079435	lipase E, hormone sensitive type
WAC-AS1	0.76	3.56	ENSG00000254635	WAC antisense RNA 1 (head to head)
RAB4B	0.76	3.26	ENSG00000167578	RAB4B, member RAS oncogene family
ZNF251	0.76	4.28	ENSG00000278062	zinc finger protein 251
PRRT1	0.76	1.75	ENSG00000229071	proline rich transmembrane protein 1

NOTUM	0.76	4.58	ENSG00000185269	NOTUM, palmitoleoyl-protein carboxylesterase
ANKRD6	0.76	3.29	ENSG00000135299	ankyrin repeat domain 6
IL18BP	0.75	3.22	ENSG00000137496	interleukin 18 binding protein
CTD-2124B8.2	0.75	1.03	ENSG00000270558	N/A
KCNH3	0.75	1.03	ENSG00000135519	potassium voltage-gated channel subfamily H member 3
SCG5	0.75	1.53	ENSG00000281931	secretogranin V
CTC-444N24.8	0.75	2.20	ENSG00000268713	N/A
LYNX1	0.75	4.51	ENSG00000180155	Ly6/neurotoxin 1
SPOCK3	0.75	1.81	ENSG00000196104	SPARC/osteonectin, cwcv and kazal like domains proteoglycan 3
PORCN	0.75	2.04	ENSG00000102312	porcupine homolog (Drosophila)
ERBB3	0.75	7.64	ENSG00000065361	erb-b2 receptor tyrosine kinase 3
UBE2H	0.75	6.68	ENSG00000186591	ubiquitin conjugating enzyme E2 H
PACS1	0.75	9.12	ENSG00000175115	phosphofurin acidic cluster sorting protein 1
GABARAP	0.75	4.32	ENSG00000170296	GABA type A receptor-associated protein
TMEM54	0.75	3.39	ENSG00000121900	transmembrane protein 54
RPL39L	0.75	4.82	ENSG00000163923	ribosomal protein L39 like
PRSS12	0.74	4.50	ENSG00000164099	protease, serine 12
DDX60L	0.74	2.29	ENSG00000181381	DEAD-box helicase 60-like
HNRNPUL2	0.74	5.99	ENSG00000214753	heterogeneous nuclear ribonucleoprotein U like 2
RP1-257A7.5	0.74	1.13	ENSG00000272379	N/A
FAM131C	0.74	2.39	ENSG00000185519	family with sequence similarity 131 member C
MIF4GD	0.74	3.43	ENSG00000125457	MIF4G domain containing
ZNF528	0.74	4.28	ENSG00000167555	zinc finger protein 528
LGALS3BP	0.74	8.99	ENSG00000108679	galectin 3 binding protein
FABP1	0.74	5.03	ENSG00000163586	fatty acid binding protein 1
CD7	0.74	4.16	ENSG00000173762	CD7 molecule
EFNA4	0.74	4.61	ENSG00000243364	ephrin A4
PROX1-AS1	0.74	2.64	ENSG00000272167	N/A
PLLP	0.74	3.53	ENSG00000102934	plasmolipin

ENPP5	0.74	1.67	ENSG00000112796	ectonucleotide pyrophosphatase/phosphodiesterase 5 (putative)
LRRC31	0.74	1.67	ENSG00000114248	leucine rich repeat containing 31
TNIK	0.74	5.90	ENSG00000154310	TRAF2 and NCK interacting kinase
PCLO	0.74	2.06	ENSG00000186472	piccolo presynaptic cytomatrix protein
JUNB	0.74	6.97	ENSG00000171223	JunB proto-oncogene, AP-1 transcription factor subunit
LYN	0.74	5.15	ENSG00000254087	LYN proto-oncogene, Src family tyrosine kinase
HSPA1A	0.74	4.76	ENSG00000234475	heat shock protein family A (Hsp70) member 1A
TRIM2	0.74	3.41	ENSG00000109654	tripartite motif containing 2
TACC1	0.74	7.76	ENSG00000147526	transforming acidic coiled-coil containing protein 1
AMACR	0.74	1.56	ENSG00000242110	alpha-methylacyl-CoA racemase
CCDC148	0.74	1.56	ENSG00000153237	coiled-coil domain containing 148
FMO4	0.74	2.19	ENSG00000076258	flavin containing monooxygenase 4
GINM1	0.74	3.76	ENSG00000055211	glycoprotein integral membrane 1
ZNF93	0.74	4.77	ENSG00000184635	zinc finger protein 93
LCA5	0.74	4.31	ENSG00000135338	LCA5, lebercilin
FSTL3	0.74	7.28	ENSG00000070404	follistatin like 3
MAP3K12	0.74	1.22	ENSG00000139625	mitogen-activated protein kinase kinase kinase 12
SIDT2	0.74	3.84	ENSG00000149577	SID1 transmembrane family member 2
RP11-421F16.3	0.74	3.39	ENSG00000247903	N/A
VIM	0.73	9.03	ENSG00000026025	vimentin
RARG	0.73	1.89	ENSG00000172819	retinoic acid receptor gamma
KIFAP3	0.73	5.01	ENSG00000075945	kinesin associated protein 3
RCOR2	0.73	4.42	ENSG00000167771	REST corepressor 2
TMEM179B	0.73	5.08	ENSG00000185475	transmembrane protein 179B
TPBG	0.73	5.91	ENSG00000283085	trophoblast glycoprotein
RAB30-AS1	0.73	1.63	ENSG00000246067	RAB30 antisense RNA 1 (head to head)
PPP1R26	0.73	4.67	ENSG00000196422	protein phosphatase 1 regulatory subunit 26
CD99P1	0.73	1.07	ENSG00000223773	CD99 molecule pseudogene 1

RABL2A	0.73	2.28	ENSG00000144134	RAB, member of RAS oncogene family-like 2A
CKAP2L	0.73	5.73	ENSG00000169607	cytoskeleton associated protein 2 like
GUCY1A2	0.73	5.18	ENSG00000152402	guanylate cyclase 1 soluble subunit alpha 2
CRTC2	0.73	6.37	ENSG00000160741	CREB regulated transcription coactivator 2
GRAMD1C	0.73	2.08	ENSG00000178075	GRAM domain containing 1C
FBXO2	0.73	4.73	ENSG00000116661	F-box protein 2
SERPINA1	0.73	10.82	ENSG00000197249	serpin family A member 1
PCDHGB4	0.73	1.52	ENSG00000253953	protocadherin gamma subfamily B, 4
AIFM3	0.73	1.70	ENSG00000183773	apoptosis inducing factor, mitochondria associated 3
LYPD6B	0.73	5.37	ENSG00000150556	LY6/PLAUR domain containing 6B
RHBG	0.73	2.13	ENSG00000132677	Rh family B glycoprotein (gene/pseudogene)
TPST1	0.73	5.20	ENSG00000169902	tyrosylprotein sulfotransferase 1
PAPLN	0.73	2.86	ENSG00000100767	papilin, proteoglycan like sulfated glycoprotein
OS9	0.72	7.36	ENSG00000135506	OS9, endoplasmic reticulum lectin
LINC00694	0.72	1.17	ENSG00000225873	long intergenic non-protein coding RNA 694
PRKD1	0.72	4.45	ENSG00000184304	protein kinase D1
SPA17	0.72	2.18	ENSG00000064199	sperm autoantigenic protein 17
FOXO3	0.72	4.52	ENSG00000118689	forkhead box O3
ATP2B4	0.72	3.46	ENSG00000058668	ATPase plasma membrane Ca2+ transporting 4
SPTLC3	0.72	2.95	ENSG00000172296	serine palmitoyltransferase long chain base subunit 3
FURIN	0.72	9.47	ENSG00000140564	furin, paired basic amino acid cleaving enzyme
MFSD6	0.72	5.79	ENSG00000151690	major facilitator superfamily domain containing 6
NKIRAS1	0.72	3.17	ENSG00000197885	NFKB inhibitor interacting Ras like 1
LINC00315	0.72	1.01	ENSG00000184274	long intergenic non-protein coding RNA 315
CPLX1	0.72	3.94	ENSG00000168993	complexin 1
LMAN2L	0.72	6.15	ENSG00000114988	lectin, mannose binding 2 like

ZNF879	0.72	2.27	ENSG00000234284	zinc finger protein 879
ARRDC2	0.71	3.82	ENSG00000105643	arrestin domain containing 2
BTC	0.71	2.31	ENSG00000174808	betacellulin
TRAK2	0.71	5.52	ENSG00000115993	trafficking kinesin protein 2
BTG3	0.71	4.56	ENSG00000281484	BTG anti-proliferation factor 3
RASSF3	0.71	6.41	ENSG00000153179	Ras association domain family member 3
PPM1H	0.71	6.15	ENSG00000111110	protein phosphatase, Mg2+/Mn2+ dependent 1H
SYS1	0.71	5.80	ENSG00000204070	SYS1, golgi trafficking protein
FAM76A	0.71	2.76	ENSG00000009780	family with sequence similarity 76 member A
RP11-488C13.5	0.71	1.11	ENSG00000258301	N/A
RP11-384K6.6	0.71	1.78	ENSG00000260404	N/A
CCDC88B	0.71	2.24	ENSG00000168071	coiled-coil domain containing 88B
HLA-L	0.71	2.87	ENSG00000243753	major histocompatibility complex, class I, L (pseudogene)
CTBP2	0.71	5.34	ENSG00000175029	C-terminal binding protein 2
CTD-2366F13.1	0.71	1.42	ENSG00000247796	N/A
TP53INP2	0.71	5.91	ENSG00000078804	tumor protein p53 inducible nuclear protein 2
DHRS12	0.71	1.20	ENSG00000102796	dehydrogenase/reductase 12
C4orf19	0.71	5.14	ENSG00000154274	chromosome 4 open reading frame 19
KLHL13	0.70	3.05	ENSG00000003096	kelch like family member 13
CDH7	0.70	2.47	ENSG00000081138	cadherin 7
RFX2	0.70	4.24	ENSG00000087903	regulatory factor X2
PRRG2	0.70	1.50	ENSG00000126460	proline rich and Gla domain 2
FLRT3	0.70	5.99	ENSG00000125848	fibronectin leucine rich transmembrane protein 3
MAPKAPK2	0.70	7.09	ENSG00000162889	mitogen-activated protein kinase-activated protein kinase 2
ZNF613	0.70	2.83	ENSG00000176024	zinc finger protein 613
INSIG2	0.70	5.39	ENSG00000125629	insulin induced gene 2
ZNF827	0.70	4.52	ENSG00000151612	zinc finger protein 827
WDPCP	0.70	2.41	ENSG00000143951	WD repeat containing planar cell polarity effector

C17orf97	0.70	3.12	ENSG00000187624	chromosome 17 open reading frame 97
LINC00176	0.70	1.29	ENSG00000196421	long intergenic non-protein coding RNA 176
ZNF345	0.70	1.29	ENSG00000251247	zinc finger protein 345
SOX2	0.70	3.83	ENSG00000181449	SRY-box 2
NBEA	0.70	1.57	ENSG00000172915	neurobeachin
BCL11A	0.70	3.25	ENSG00000119866	B-cell CLL/lymphoma 11A
GPR137B	0.70	2.19	ENSG00000077585	G protein-coupled receptor 137B
MIR24-2	0.70	2.19	ENSG00000267519	microRNA 24-2
HID1	0.70	6.42	ENSG00000167861	HID1 domain containing
B4GALT5	0.70	6.79	ENSG00000158470	beta-1,4-galactosyltransferase 5
REC8	0.70	1.38	ENSG00000100918	REC8 meiotic recombination protein
RP11-96K19.2	0.70	1.38	ENSG00000232811	N/A
IRF2	0.70	4.80	ENSG00000168310	interferon regulatory factor 2
ZNF132	0.70	1.64	ENSG00000131849	zinc finger protein 132
MOK	0.70	1.87	ENSG00000080823	MOK protein kinase
TSPAN33	0.70	3.51	ENSG00000158457	tetraspanin 33
TNFAIP8	0.70	4.93	ENSG00000145779	TNF alpha induced protein 8
RNF43	0.69	6.61	ENSG00000108375	ring finger protein 43
CDC42SE2	0.69	6.15	ENSG00000158985	CDC42 small effector 2
C14orf28	0.69	1.92	ENSG00000179476	chromosome 14 open reading frame 28
RP4-635E18.8	0.69	1.71	ENSG00000271895	N/A
MUC1	0.69	1.15	ENSG00000185499	mucin 1, cell surface associated
EFNA2	0.69	6.50	ENSG00000099617	ephrin A2
ZNF69	0.69	3.32	ENSG00000198429	zinc finger protein 69
F10	0.69	5.49	ENSG00000126218	coagulation factor X
NMI	0.69	2.63	ENSG00000123609	N-myc and STAT interactor
AC005355.2	0.69	1.77	ENSG00000251169	N/A
SNAPC1	0.69	1.77	ENSG00000023608	small nuclear RNA activating complex polypeptide 1
C4BPA	0.69	4.80	ENSG00000123838	complement component 4 binding protein alpha
SRP14-AS1	0.69	1.53	ENSG00000248508	SRP14 antisense RNA1 (head to head)
ARSG	0.69	1.24	ENSG00000141337	arylsulfatase G
ERCC5	0.69	4.56	ENSG00000134899	ERCC excision repair 5, endonuclease
SRGAP1	0.69	2.57	ENSG00000196935	SLIT-ROBO Rho GTPase activating protein 1

EFEMP1	0.69	2.84	ENSG00000115380	EGF containing fibulin like extracellular matrix protein 1
THRA	0.69	5.04	ENSG00000126351	thyroid hormone receptor, alpha
EPB41L2	0.69	8.16	ENSG00000079819	erythrocyte membrane protein band 4.1 like 2
LEPROT	0.69	3.51	ENSG00000213625	leptin receptor overlapping transcript
LIN37	0.69	1.60	ENSG00000267796	lin-37 DREAM MuvB core complex component
DHRS3	0.69	5.44	ENSG00000162496	dehydrogenase/reductase 3
TRIOBP	0.69	4.03	ENSG00000100106	TRIO and F-actin binding protein
GPR153	0.68	3.46	ENSG00000158292	G protein-coupled receptor 153
YPEL2	0.68	5.01	ENSG00000175155	yippee like 2
TMEM217	0.68	2.13	ENSG00000172738	transmembrane protein 217
GRAMD3	0.68	3.57	ENSG00000155324	GRAM domain containing 3
GCNT4	0.68	3.94	ENSG00000176928	glucosaminyl (N-acetyl) transferase 4, core 2
RBP1	0.68	5.06	ENSG00000114115	retinol binding protein 1
SCAMP5	0.68	5.99	ENSG00000198794	secretory carrier membrane protein 5
IL27RA	0.68	4.28	ENSG00000104998	interleukin 27 receptor subunit alpha
C3	0.68	12.11	ENSG00000125730	complement C3
ZNF585B	0.68	3.61	ENSG00000245680	zinc finger protein 585B
WWP1	0.68	6.40	ENSG00000123124	WW domain containing E3 ubiquitin protein ligase 1
KIAA1462	0.68	4.28	ENSG00000165757	KIAA1462
SSTR2	0.68	3.72	ENSG00000180616	somatostatin receptor 2
AK9	0.68	2.49	ENSG00000155085	adenylate kinase 9
SMURF1	0.68	5.85	ENSG00000198742	SMAD specific E3 ubiquitin protein ligase 1
KSR1	0.68	3.58	ENSG00000141068	kinase suppressor of ras 1
SAT1	0.68	5.55	ENSG00000130066	spermidine/spermine N1-acetyltransferase 1
STIM2	0.68	6.75	ENSG00000109689	stromal interaction molecule 2
AC074289.1	0.68	1.86	ENSG00000225889	N/A
PACRGL	0.68	4.83	ENSG00000163138	PARK2 coregulated like
AC022007.5	0.67	1.91	ENSG00000206567	N/A
RP11-465N4.4	0.67	1.91	ENSG00000234678	N/A
TNNI1	0.67	2.92	ENSG00000159173	troponin I1, slow skeletal type

ITGB1P1	0.67	1.19	ENSG00000269378	integrin subunit beta 1 pseudogene 1
WBP1	0.67	2.60	ENSG00000239779	WW domain binding protein 1
SLC39A7	0.67	8.30	ENSG00000227402	solute carrier family 39 member 7
CSPP1	0.67	5.63	ENSG00000104218	centrosome and spindle pole associated protein 1
C11orf74	0.67	5.18	ENSG00000166352	chromosome 11 open reading frame 74
RBM3	0.67	6.29	ENSG00000102317	RNA binding motif (RNP1, RRM) protein 3
KLHL24	0.67	5.62	ENSG00000114796	kelch like family member 24
COL12A1	0.67	3.14	ENSG00000111799	collagen type XII alpha 1 chain
SYNGR1	0.67	3.39	ENSG00000100321	synaptogyrin 1
TOX3	0.67	6.87	ENSG00000103460	TOX high mobility group box family member 3
HINT3	0.67	4.43	ENSG00000111911	histidine triad nucleotide binding protein 3
VPS45	0.67	5.81	ENSG00000136631	vacuolar protein sorting 45 homolog
TM6SF1	0.67	2.07	ENSG00000136404	transmembrane 6 superfamily member 1
PERP	0.67	7.62	ENSG00000112378	PERP, TP53 apoptosis effector
IQCE	0.67	6.38	ENSG00000106012	IQ motif containing E
GMDS-AS1	0.67	1.36	ENSG00000250903	GMDS antisense RNA 1 (head to head)
RP11-119F7.5	0.67	2.12	ENSG00000260400	N/A
SMPDL3B	0.67	2.98	ENSG00000130768	sphingomyelin phosphodiesterase acid like 3B
TBPL1	0.67	4.77	ENSG00000028839	TATA-box binding protein like 1
AP001062.7	0.67	1.82	ENSG00000184441	N/A
ZNF793	0.67	3.15	ENSG00000188227	zinc finger protein 793
STYXL1	0.67	4.80	ENSG00000127952	serine/threonine/tyrosine interacting like 1
AKTIP	0.67	2.17	ENSG00000166971	AKT interacting protein
MYO16	0.67	1.44	ENSG00000282848	myosin XVI
RP3-508I15.9	0.67	1.44	ENSG00000228274	N/A
PDGFA	0.67	6.20	ENSG00000197461	platelet derived growth factor subunit A
C15orf52	0.67	6.41	ENSG00000188549	chromosome 15 open reading frame 52
RHOBTB1	0.66	5.17	ENSG00000072422	Rho related BTB domain containing 1
CAPN5	0.66	3.92	ENSG00000149260	calpain 5
NEK2	0.66	5.65	ENSG00000117650	NIMA related kinase 2

NPDC1	0.66	3.84	ENSG00000107281	neural proliferation, differentiation and control 1
ZNF432	0.66	3.40	ENSG00000256087	zinc finger protein 432
C8orf37	0.66	1.99	ENSG00000156172	chromosome 8 open reading frame 37
SLC22A3	0.66	4.32	ENSG00000146477	solute carrier family 22 member 3
DAZAP2P1	0.66	1.59	ENSG00000223825	DAZ associated protein 2 pseudogene 1
SNAI2	0.66	1.59	ENSG00000019549	snail family transcriptional repressor 2
DYNLT3	0.66	4.41	ENSG00000165169	dynein light chain Tctex-type 3
HIST1H2BD	0.66	3.63	ENSG00000158373	histone cluster 1 H2B family member d
ARHGEF38	0.66	1.03	ENSG00000236699	Rho guanine nucleotide exchange factor 38
RPGRIP1	0.66	1.03	ENSG00000092200	retinitis pigmentosa GTPase regulator interacting protein 1
CTD-2561J22.5	0.66	2.14	ENSG00000268119	N/A
IL17RC	0.66	4.99	ENSG00000163702	interleukin 17 receptor C
CTSO	0.66	3.01	ENSG00000256043	cathepsin O
IER5	0.66	4.63	ENSG00000162783	immediate early response 5
CDH6	0.66	8.10	ENSG00000113361	cadherin 6
LRCH1	0.66	3.26	ENSG00000136141	leucine rich repeats and calponin homology domain containing 1
ELK4	0.66	5.74	ENSG00000158711	ELK4, ETS transcription factor
SERPINA5	0.66	8.55	ENSG00000188488	serpin family A member 5
C14orf79	0.66	3.28	ENSG00000140104	chromosome 14 open reading frame 79
GLP2R	0.66	2.28	ENSG00000065325	glucagon like peptide 2 receptor
TMEM37	0.66	6.78	ENSG00000171227	transmembrane protein 37
KIF6	0.65	1.90	ENSG00000164627	kinesin family member 6
NDRG4	0.65	3.68	ENSG00000103034	NDRG family member 4
AP2B1	0.65	8.20	ENSG00000006125	adaptor related protein complex 2 beta 1 subunit
CYB561	0.65	5.82	ENSG00000008283	cytochrome b561
SUCO	0.65	7.89	ENSG00000094975	SUN domain containing ossification factor
B4GALT4	0.65	4.47	ENSG00000121578	beta-1,4-galactosyltransferase 4
ECE1	0.65	7.18	ENSG00000117298	endothelin converting enzyme 1
FN1	0.65	11.10	ENSG00000115414	fibronectin 1
ZMIZ2	0.65	7.59	ENSG00000122515	zinc finger MIZ-type containing 2
RP11-428J1.5	0.65	1.47	ENSG00000272142	N/A

NPNT	0.65	5.47	ENSG00000168743	nephronectin
CRMP1	0.65	3.35	ENSG00000072832	collapsin response mediator protein 1
SMAD7	0.65	5.52	ENSG00000101665	SMAD family member 7
TMEM127	0.65	6.88	ENSG00000135956	transmembrane protein 127
PTPN12	0.65	7.31	ENSG00000127947	protein tyrosine phosphatase, non-receptor type 12
LRIG1	0.65	5.13	ENSG00000282243	leucine rich repeats and immunoglobulin like domains 1
MALAT1	0.65	6.51	ENSG00000278217	Metastasis associated lung adenocarcinoma transcript 1
MSN	0.65	7.76	ENSG00000147065	moesin
NXPH4	0.65	2.66	ENSG00000182379	neurexophilin 4
RHBDF2	0.65	6.82	ENSG00000129667	rhomboid 5 homolog 2
MRPL53	0.65	1.68	ENSG00000204822	mitochondrial ribosomal protein L53
TMEM237	0.65	5.82	ENSG00000155755	transmembrane protein 237
PAQR8	0.65	8.43	ENSG00000170915	progestin and adipoQ receptor family member 8
TMCC3	0.65	3.08	ENSG00000057704	transmembrane and coiled-coil domain family 3
RP11-568K15.1	0.65	3.10	ENSG00000242193	N/A
CHODL	0.65	5.41	ENSG00000154645	chondrolectin
PPCS	0.65	4.49	ENSG00000127125	phosphopantothenoylcysteine synthetase
ZNF667-AS1	0.64	4.12	ENSG00000166770	ZNF667 antisense RNA 1 (head to head)
CDON	0.64	5.43	ENSG00000064309	cell adhesion associated, oncogene regulated
ZNF90	0.64	2.60	ENSG00000213988	zinc finger protein 90
BLOC1S1	0.64	4.09	ENSG00000135441	biogenesis of lysosomal organelles complex 1 subunit 1
MAN2B2	0.64	6.36	ENSG00000013288	mannosidase alpha class 2B member 2
ITGA7	0.64	2.08	ENSG00000135424	integrin subunit alpha 7
PDE3A	0.64	2.08	ENSG00000172572	phosphodiesterase 3A
SMOC1	0.64	5.09	ENSG00000198732	SPARC related modular calcium binding 1
CCNG2	0.64	4.74	ENSG00000138764	cyclin G2
WWC1	0.64	7.69	ENSG00000113645	WW and C2 domain containing 1
CLMN	0.64	5.98	ENSG00000165959	calmin
OPLAH	0.64	3.82	ENSG00000178814	5-oxoprolinase (ATP-hydrolysing)

TIFA	0.64	4.51	ENSG00000145365	TRAF interacting protein with forkhead associated domain
MFAP4	0.64	1.42	ENSG00000166482	microfibrillar associated protein 4
GRN	0.64	8.42	ENSG00000030582	granulin precursor
ABCB4	0.64	2.58	ENSG00000005471	ATP binding cassette subfamily B member 4
GALNT11	0.64	4.70	ENSG00000178234	polypeptide N-acetylgalactosaminyltransferase 11
PTBP3	0.64	7.77	ENSG00000119314	polypyrimidine tract binding protein 3
RNF215	0.64	1.64	ENSG00000099999	ring finger protein 215
ZNF571	0.64	1.71	ENSG00000180479	zinc finger protein 571
DLGAP1-AS1	0.64	3.46	ENSG00000177337	DLGAP1 antisense RNA 1
DEGS1	0.64	6.56	ENSG00000143753	delta 4-desaturase, sphingolipid 1
UPP1	0.64	3.52	ENSG00000183696	uridine phosphorylase 1
TCF7L2	0.64	5.84	ENSG00000148737	transcription factor 7 like 2
AP3S2	0.64	3.57	ENSG00000157823	adaptor related protein complex 3 sigma 2 subunit
ZNF347	0.64	2.98	ENSG00000197937	zinc finger protein 347
FUT4	0.64	4.03	ENSG00000196371	fucosyltransferase 4
RPGR	0.64	3.01	ENSG00000156313	retinitis pigmentosa GTPase regulator
IFITM2	0.63	5.50	ENSG00000185201	interferon induced transmembrane protein 2
TTLL1	0.63	3.25	ENSG00000100271	tubulin tyrosine ligase like 1
C11orf80	0.63	5.49	ENSG00000173715	chromosome 11 open reading frame 80
ATXN1	0.63	4.09	ENSG00000124788	ataxin 1
PTX3	0.63	4.53	ENSG00000163661	pentraxin 3
TNFRSF10B	0.63	7.00	ENSG00000120889	TNF receptor superfamily member 10b
ATF3	0.63	5.03	ENSG00000162772	activating transcription factor 3
PCSK1N	0.63	2.66	ENSG00000102109	proprotein convertase subtilisin/kexin type 1 inhibitor
ZNF773	0.63	2.82	ENSG00000152439	zinc finger protein 773
SNX18	0.63	6.55	ENSG00000178996	sorting nexin 18
IFNLR1	0.63	4.37	ENSG00000185436	interferon lambda receptor 1
MYH9	0.63	8.76	ENSG00000100345	myosin heavy chain 9
PLCD4	0.63	3.30	ENSG00000115556	phospholipase C delta 4
TMEM69	0.63	4.99	ENSG00000159596	transmembrane protein 69
PSD4	0.63	4.54	ENSG00000125637	pleckstrin and Sec7 domain containing 4

STK38L	0.63	5.40	ENSG00000211455	serine/threonine kinase 38 like
ATL3	0.63	5.64	ENSG00000184743	atlastin GTPase 3
F2R	0.63	4.35	ENSG00000181104	coagulation factor II thrombin receptor
ORAI3	0.63	2.83	ENSG00000175938	ORAI calcium release-activated calcium modulator 3
ZKSCAN7	0.63	2.77	ENSG00000281894	zinc finger with KRAB and SCAN domains 7
PFN2	0.63	6.18	ENSG00000070087	profilin 2
SPINK1	0.63	1.97	ENSG00000164266	serine peptidase inhibitor, Kazal type 1
PTPRB	0.63	1.60	ENSG00000127329	protein tyrosine phosphatase, receptor type B
C6orf226	0.62	1.20	ENSG00000221821	chromosome 6 open reading frame 226
RP11-25K19.1	0.62	1.01	ENSG00000167912	N/A
TMEM87B	0.62	4.74	ENSG00000153214	transmembrane protein 87B
SHFM1	0.62	6.15	ENSG00000127922	split hand/foot malformation (ectrodactyly) type 1
ZSCAN16	0.62	4.05	ENSG00000196812	zinc finger and SCAN domain containing 16
SATB1	0.62	3.85	ENSG00000182568	SATB homeobox 1
WASF1	0.62	6.21	ENSG00000112290	WAS protein family member 1
C1QTNF1	0.62	3.68	ENSG00000173918	C1q and tumor necrosis factor related protein 1
ZBTB22	0.62	4.53	ENSG00000206280	zinc finger and BTB domain containing 22
CAB39	0.62	7.07	ENSG00000135932	calcium binding protein 39
LRP12	0.62	4.18	ENSG00000147650	LDL receptor related protein 12
AGMO	0.62	5.16	ENSG00000187546	alkylglycerol monooxygenase
TAPBP	0.62	6.46	ENSG00000236490	TAP binding protein
KRT17	0.62	3.47	ENSG00000128422	keratin 17
MICALL2	0.62	4.43	ENSG00000164877	MICAL like 2
NOTCH3	0.62	6.32	ENSG00000074181	notch 3
IFT52	0.62	4.95	ENSG00000101052	intraflagellar transport 52
CTD-2368P22.1	0.62	3.39	ENSG00000176593	N/A
ANPEP	0.62	6.96	ENSG00000166825	alanyl aminopeptidase, membrane
SUMO2	0.62	6.94	ENSG00000188612	small ubiquitin-like modifier 2

ALDH3B1	0.62	1.88	ENSG00000006534	aldehyde dehydrogenase 3 family member B1
GPRC5C	0.62	7.40	ENSG00000170412	G protein-coupled receptor class C group 5 member C
ZNF300	0.61	5.16	ENSG00000145908	zinc finger protein 300
ZNF331	0.61	5.12	ENSG00000130844	zinc finger protein 331
ZSCAN18	0.61	5.05	ENSG00000121413	zinc finger and SCAN domain containing 18
CLU	0.61	10.57	ENSG00000120885	clusterin
CASK	0.61	5.72	ENSG00000147044	calcium/calmodulin dependent serine protein kinase
PAK1	0.61	5.40	ENSG00000149269	p21 (RAC1) activated kinase 1
ADPRHL1	0.61	2.34	ENSG00000153531	ADP-ribosylhydrolase like 1
RP11-864N7.2	0.61	2.34	ENSG00000227615	N/A
ABCB1	0.61	5.95	ENSG00000085563	ATP binding cassette subfamily B member 1
BAMBI	0.61	6.40	ENSG00000095739	BMP and activin membrane bound inhibitor
JOSD2	0.61	3.43	ENSG00000161677	Josephin domain containing 2
SLC25A45	0.61	4.35	ENSG00000162241	solute carrier family 25 member 45
ZFYVE1	0.61	4.60	ENSG00000165861	zinc finger FYVE-type containing 1
ST6GALNAC6	0.61	2.62	ENSG00000160408	ST6 N-acetylgalactosaminide alpha-2,6-sialyltransferase 6
COX16	0.61	3.55	ENSG00000133983	COX16, cytochrome c oxidase assembly homolog
XPR1	0.61	7.18	ENSG00000143324	xenotropic and polytropic retrovirus receptor 1
POMT2	0.61	5.60	ENSG00000009830	protein O-mannosyltransferase 2
STMN1	0.61	8.94	ENSG00000117632	stathmin 1
KDELR3	0.61	5.53	ENSG00000100196	KDEL endoplasmic reticulum protein retention receptor 3
C20orf196	0.61	1.05	ENSG00000171984	chromosome 20 open reading frame 196
OIP5	0.61	3.63	ENSG00000104147	Opa interacting protein 5
LINC00094	0.61	3.97	ENSG00000235106	long intergenic non-protein coding RNA 94
AGBL2	0.61	1.90	ENSG00000165923	ATP/GTP binding protein like 2
MAP3K1	0.60	5.80	ENSG00000095015	mitogen-activated protein kinase kinase kinase 1

TMEM182	0.60	2.31	ENSG00000170417	transmembrane protein 182
LIPH	0.60	2.70	ENSG00000163898	lipase H
ZFP90	0.60	4.43	ENSG00000184939	ZFP90 zinc finger protein
PCMTD1	0.60	4.18	ENSG00000168300	protein-L-isoaspartate (D-aspartate) O-methyltransferase domain containing 1
GRINA	0.60	8.31	ENSG00000178719	glutamate ionotropic receptor NMDA type subunit associated protein 1
SERPING1	0.60	6.09	ENSG00000149131	serpin family G member 1
TCTN1	0.60	3.14	ENSG00000204852	tectonic family member 1
TP53I3	0.60	4.80	ENSG00000115129	tumor protein p53 inducible protein 3
ZNF502	0.60	2.52	ENSG00000281448	zinc finger protein 502
CLTB	0.60	6.66	ENSG00000175416	clathrin light chain B
LZTFL1	0.60	3.26	ENSG00000163818	leucine zipper transcription factor like 1
EPHX1	0.60	6.87	ENSG00000143819	epoxide hydrolase 1
SNRPN	0.60	6.12	ENSG00000128739	small nuclear ribonucleoprotein polypeptide N
FTL	-0.60	11.88	ENSG00000087086	ferritin light chain
TFAP4	-0.60	4.94	ENSG00000090447	transcription factor AP-4
ZMYND19	-0.60	5.68	ENSG00000165724	zinc finger MYND-type containing 19
CDK14	-0.60	5.91	ENSG00000058091	cyclin dependent kinase 14
TCHP	-0.60	3.55	ENSG00000139437	trichoplein keratin filament binding
FAT4	-0.60	3.61	ENSG00000196159	FAT atypical cadherin 4
AVPI1	-0.60	3.86	ENSG00000119986	arginine vasopressin induced 1
TMEM97	-0.60	8.16	ENSG00000109084	transmembrane protein 97
NCBP2-AS2	-0.60	5.15	ENSG00000270170	NCBP2 antisense RNA 2 (head to head)
AC113189.5	-0.61	2.35	ENSG00000233223	N/A
ETNK1	-0.61	6.11	ENSG00000139163	ethanolamine kinase 1
OAT	-0.61	3.76	ENSG00000065154	ornithine aminotransferase
MAGEA6	-0.61	1.77	ENSG00000197172	MAGE family member A6
BTAF1	-0.61	5.66	ENSG00000095564	B-TFIID TATA-box binding protein associated factor 1
PHLDA2	-0.61	4.98	ENSG00000274538	pleckstrin homology like domain family A member 2
DLC1	-0.61	6.62	ENSG00000164741	DLC1 Rho GTPase activating protein
C22orf29	-0.61	5.35	ENSG00000215012	chromosome 22 open reading frame 29
FAM83H-AS1	-0.61	2.31	ENSG00000203499	FAM83H antisense RNA 1 (head to head)

SLC30A1	-0.61	4.70	ENSG00000170385	solute carrier family 30 member 1
ARHGAP24	-0.61	2.71	ENSG00000138639	Rho GTPase activating protein 24
ADAP1	-0.61	3.97	ENSG00000105963	ArfGAP with dual PH domains 1
RBP5	-0.61	1.45	ENSG00000139194	retinol binding protein 5
VN1R81P	-0.61	1.92	ENSG00000268357	vomeronasal 1 receptor 81 pseudogene
SFXN2	-0.61	3.62	ENSG00000156398	sideroflexin 2
AEN	-0.61	5.25	ENSG00000181026	apoptosis enhancing nuclease
IMP3	-0.61	5.94	ENSG00000177971	IMP3, U3 small nucleolar ribonucleoprotein
TRABD	-0.61	6.04	ENSG00000170638	TraB domain containing
PIAS2	-0.61	4.23	ENSG00000078043	protein inhibitor of activated STAT 2
EMC6	-0.61	2.23	ENSG00000127774	ER membrane protein complex subunit 6
NLE1	-0.61	5.36	ENSG00000073536	notchless homolog 1
PRKDC	-0.61	9.78	ENSG00000253729	protein kinase, DNA-activated, catalytic polypeptide
GAMT	-0.61	6.40	ENSG00000130005	guanidinoacetate N-methyltransferase
PLXNA1	-0.61	8.02	ENSG00000114554	plexin A1
BCYRN1	-0.61	1.04	ENSG00000236824	brain cytoplasmic RNA 1
DPF3	-0.61	1.04	ENSG00000205683	double PHD fingers 3
TSR1	-0.61	6.84	ENSG00000167721	TSR1, ribosome maturation factor
TIMM8A	-0.62	2.78	ENSG00000126953	translocase of inner mitochondrial membrane 8 homolog A (yeast)
ABHD16A	-0.62	1.57	ENSG00000230475	abhydrolase domain containing 16A
SNHG6	-0.62	4.04	ENSG00000245910	small nucleolar RNA host gene 6
IL17RE	-0.62	2.13	ENSG00000163701	interleukin 17 receptor E
TMEM164	-0.62	5.54	ENSG00000157600	transmembrane protein 164
EEF1DP1	-0.62	1.29	ENSG00000228887	eukaryotic translation elongation factor 1 delta pseudogene 1
ASGR1	-0.62	6.89	ENSG00000141505	asialoglycoprotein receptor 1
ZFP69B	-0.62	1.74	ENSG00000187801	ZFP69 zinc finger protein B
MBLAC2	-0.62	2.08	ENSG00000176055	metallo-beta-lactamase domain containing 2
CHCHD5	-0.62	3.80	ENSG00000125611	coiled-coil-helix-coiled-coil-helix domain containing 5
KATNB1	-0.62	4.94	ENSG00000140854	katanin regulatory subunit B1
OPN3	-0.62	5.07	ENSG00000054277	opsin 3
MDN1	-0.62	7.23	ENSG00000112159	midasin AAA ATPase 1

WDR12	-0.62	5.96	ENSG00000138442	WD repeat domain 12
C1orf106	-0.62	5.89	ENSG00000163362	chromosome 1 open reading frame 106
FOXN2	-0.62	5.30	ENSG00000170802	forkhead box N2
AC009403.2	-0.63	1.61	ENSG00000216895	N/A
COL4A1	-0.63	1.61	ENSG00000187498	collagen type IV alpha 1 chain
BMP2	-0.63	6.54	ENSG00000125845	bone morphogenetic protein 2
GSTZ1	-0.63	2.35	ENSG00000100577	glutathione S-transferase zeta 1
NDUFAF4	-0.63	4.91	ENSG00000123545	NADH:ubiquinone oxidoreductase complex assembly factor 4
ONECUT1	-0.63	4.94	ENSG00000169856	one cut homeobox 1
DIS3L	-0.63	4.39	ENSG00000166938	DIS3 like exosome 3'-5' exoribonuclease
DDX21	-0.63	7.89	ENSG00000165732	DExD-box helicase 21
CLDN1	-0.63	10.75	ENSG00000163347	claudin 1
EBPL	-0.63	3.62	ENSG00000123179	emopamil binding protein like
PRDM5	-0.63	3.41	ENSG00000138738	PR/SET domain 5
GSTO2	-0.63	2.30	ENSG00000065621	glutathione S-transferase omega 2
ARTN	-0.63	1.54	ENSG00000117407	artemin
CXCR4	-0.63	4.85	ENSG00000121966	C-X-C motif chemokine receptor 4
ARRDC3	-0.63	2.88	ENSG00000113369	arrestin domain containing 3
RBM47	-0.63	5.37	ENSG00000163694	RNA binding motif protein 47
CTA-221G9.11	-0.63	1.71	ENSG00000279298	N/A
DGCR6	-0.63	2.01	ENSG00000183628	DiGeorge syndrome critical region gene 6
SLC25A19	-0.63	3.99	ENSG00000125454	solute carrier family 25 member 19
ALDH16A1	-0.63	5.71	ENSG00000161618	aldehyde dehydrogenase 16 family member A1
PAWR	-0.63	6.45	ENSG00000177425	pro-apoptotic WT1 regulator
DNAJC3-AS1	-0.63	1.65	ENSG00000247400	DNAJC3 antisense RNA 1 (head to head)
IFI27L1	-0.64	2.22	ENSG00000276880	interferon alpha inducible protein 27 like 1
ADCY9	-0.64	6.74	ENSG00000162104	adenylate cyclase 9
AC009404.2	-0.64	2.62	ENSG00000236255	N/A
TTC39C	-0.64	5.23	ENSG00000168234	tetratricopeptide repeat domain 39C
ANKS6	-0.64	4.08	ENSG00000165138	ankyrin repeat and sterile alpha motif domain containing 6
UNG	-0.64	6.04	ENSG00000076248	uracil DNA glycosylase

WWC2	-0.64	6.44	ENSG00000151718	WW and C2 domain containing 2
ALS2CL	-0.64	2.29	ENSG00000178038	ALS2 C-terminal like
RAB17	-0.64	6.30	ENSG00000124839	RAB17, member RAS oncogene family
NOL6	-0.64	6.39	ENSG00000165271	nucleolar protein 6
SNTB1	-0.64	5.69	ENSG00000172164	syntrophin beta 1
SMIM4	-0.64	2.81	ENSG00000168273	small integral membrane protein 4
DIP2A	-0.64	6.27	ENSG00000160305	disco interacting protein 2 homolog A
KCTD21-AS1	-0.64	1.39	ENSG00000246174	KCTD21 antisense RNA 1
MIXL1	-0.64	1.39	ENSG00000185155	Mix paired-like homeobox
SLC25A42	-0.64	5.02	ENSG00000181035	solute carrier family 25 member 42
HDAC4	-0.64	4.51	ENSG00000068024	histone deacetylase 4
ADCY3	-0.64	2.97	ENSG00000138031	adenylate cyclase 3
PARVB	-0.64	5.49	ENSG00000188677	parvin beta
ATF5	-0.64	5.31	ENSG00000169136	activating transcription factor 5
ARMC6	-0.64	6.54	ENSG00000105676	armadillo repeat containing 6
UAP1L1	-0.64	5.96	ENSG00000197355	UDP-N-acetylglucosamine pyrophosphorylase 1 like 1
CCDC134	-0.64	3.18	ENSG00000100147	coiled-coil domain containing 134
RP11-958N24.1	-0.64	2.07	ENSG00000281965	N/A
PDE4D	-0.65	4.57	ENSG00000113448	phosphodiesterase 4D
PRPF4	-0.65	5.71	ENSG00000136875	pre-mRNA processing factor 4
DPH2	-0.65	5.49	ENSG00000132768	DPH2 homolog
SNHG9	-0.65	1.06	ENSG00000255198	small nucleolar RNA host gene 9
APOC3	-0.65	3.13	ENSG00000110245	apolipoprotein C3
ALG10B	-0.65	2.34	ENSG00000175548	ALG10B, alpha-1,2-glucosyltransferase
NIPSNAP3A	-0.65	4.00	ENSG00000136783	nipsnap homolog 3A
FCF1P2	-0.65	1.22	ENSG00000228638	FCF1 pseudogene 2
HNRNPKP4	-0.65	1.22	ENSG00000243547	heterogeneous nuclear ribonucleoprotein K pseudogene 4
PSMG4	-0.65	3.71	ENSG00000180822	proteasome assembly chaperone 4
IGSF9	-0.65	1.55	ENSG00000085552	immunoglobulin superfamily member 9
TGM2	-0.65	4.53	ENSG00000198959	transglutaminase 2
AJUBA	-0.65	7.21	ENSG00000129474	ajuba LIM protein
SHMT2	-0.65	7.57	ENSG00000182199	serine hydroxymethyltransferase 2
OBSCN	-0.66	2.92	ENSG00000154358	obscurin, cytoskeletal calmodulin and titin-interacting RhoGEF
HYAL3	-0.66	2.43	ENSG00000186792	hyaluronoglucosaminidase 3

CPNE7	-0.66	4.53	ENSG00000178773	copine 7
ANKMY1	-0.66	4.22	ENSG00000144504	ankyrin repeat and MYND domain containing 1
UHRF1BP1L	-0.66	5.06	ENSG00000111647	UHRF1 binding protein 1 like
GNPNAT1	-0.66	5.22	ENSG00000100522	glucosamine-phosphate N-acetyltransferase 1
DOHH	-0.66	5.66	ENSG00000129932	deoxyhypusine hydroxylase/monooxygenase
SHPRH	-0.66	4.42	ENSG00000146414	SNF2 histone linker PHD RING helicase
CDK5R1	-0.66	3.73	ENSG00000176749	cyclin dependent kinase 5 regulatory subunit 1
ISPD	-0.66	2.28	ENSG00000214960	isoprenoid synthase domain containing
PROC	-0.66	4.17	ENSG00000115718	protein C, inactivator of coagulation factors Va and VIIIa
MZT2A	-0.66	5.06	ENSG00000173272	mitotic spindle organizing protein 2A
STARD4	-0.66	5.29	ENSG00000164211	StAR related lipid transfer domain containing 4
LYPLA1	-0.66	5.78	ENSG00000120992	lysophospholipase I
PNPLA3	-0.66	4.70	ENSG00000100344	patatin like phospholipase domain containing 3
RBM15	-0.66	4.80	ENSG00000162775	RNA binding motif protein 15
AC010761.8	-0.66	1.95	ENSG00000264577	N/A
ELMO3	-0.66	1.95	ENSG00000102890	engulfment and cell motility 3
C8orf82	-0.66	5.14	ENSG00000213563	chromosome 8 open reading frame 82
DHX37	-0.66	6.34	ENSG00000150990	DEAH-box helicase 37
PDHB	-0.66	5.03	ENSG00000168291	pyruvate dehydrogenase (lipoamide) beta
GLS2	-0.66	1.70	ENSG00000135423	glutaminase 2
PLK3	-0.67	3.22	ENSG00000173846	polo like kinase 3
KRT8	-0.67	10.14	ENSG00000170421	keratin 8
ZNF316	-0.67	4.86	ENSG00000205903	zinc finger protein 316
ESRP2	-0.67	6.42	ENSG00000103067	epithelial splicing regulatory protein 2
PRR5	-0.67	4.04	ENSG00000186654	proline rich 5
FDFT1	-0.67	7.89	ENSG00000079459	farnesyl-diphosphate farnesyltransferase 1
MID1IP1	-0.67	5.24	ENSG00000165175	MID1 interacting protein 1
KRT15	-0.67	1.52	ENSG00000171346	keratin 15

NAV3	-0.67	1.02	ENSG00000067798	neuron navigator 3
PLCXD2	-0.67	1.02	ENSG00000240891	phosphatidylinositol specific phospholipase C X domain containing 2
EGLN3	-0.67	3.75	ENSG00000129521	egl-9 family hypoxia inducible factor 3
MAMLD1	-0.67	5.79	ENSG00000013619	mastermind like domain containing 1
FBXL15	-0.67	2.49	ENSG00000107872	F-box and leucine rich repeat protein 15
CELF1	-0.67	7.03	ENSG00000149187	CUGBP, Elav-like family member 1
RUSC1-AS1	-0.67	3.98	ENSG00000225855	RUSC1 antisense RNA 1
SEH1L	-0.67	5.44	ENSG00000085415	SEH1 like nucleoporin
SOCS7	-0.67	3.73	ENSG00000274229	suppressor of cytokine signaling 7
PITPNM2	-0.67	3.40	ENSG00000090975	phosphatidylinositol transfer protein membrane associated 2
C1orf210	-0.67	3.26	ENSG00000253313	chromosome 1 open reading frame 210
TSNARE1	-0.67	2.46	ENSG00000171045	t-SNARE domain containing 1
ACSS2	-0.67	4.87	ENSG00000131069	acyl-CoA synthetase short-chain family member 2
ADM5	-0.67	2.52	ENSG00000224420	adrenomedullin 5 (putative)
FKBP5	-0.67	6.03	ENSG00000096060	FK506 binding protein 5
NRP1	-0.67	6.96	ENSG00000099250	neuropilin 1
EIF4EBP1	-0.68	6.99	ENSG00000187840	eukaryotic translation initiation factor 4E binding protein 1
PM20D2	-0.68	5.90	ENSG00000146281	peptidase M20 domain containing 2
PRMT3	-0.68	5.03	ENSG00000185238	protein arginine methyltransferase 3
POLR3E	-0.68	6.04	ENSG00000058600	RNA polymerase III subunit E
FRY	-0.68	5.52	ENSG00000073910	FRY microtubule binding protein
AFF1	-0.68	5.65	ENSG00000172493	AF4/FMR2 family member 1
IL17D	-0.68	2.73	ENSG00000172458	interleukin 17D
SCD	-0.68	10.18	ENSG00000099194	stearoyl-CoA desaturase
ARID5A	-0.68	4.78	ENSG00000196843	AT-rich interaction domain 5A
QTRT1	-0.68	4.85	ENSG00000213339	queuine tRNA-ribosyltransferase catalytic subunit 1
MAF	-0.68	3.35	ENSG00000178573	MAF bZIP transcription factor
POLR3G	-0.68	2.92	ENSG00000113356	RNA polymerase III subunit G
SMCR8	-0.68	5.27	ENSG00000176994	Smith-Magenis syndrome chromosome region, candidate 8
ACLY	-0.68	9.34	ENSG00000131473	ATP citrate lyase

PI4KAP1	-0.68	1.78	ENSG00000274602	phosphatidylinositol 4-kinase alpha pseudogene 1
DHCR7	-0.68	7.47	ENSG00000172893	7-dehydrocholesterol reductase
TRMT61A	-0.68	4.98	ENSG00000166166	tRNA methyltransferase 61A
CTD-2196E14.9	-0.68	3.53	ENSG00000260482	N/A
RP11-424C20.2	-0.68	1.87	ENSG00000256663	N/A
SNCG	-0.68	2.44	ENSG00000173267	synuclein gamma
UGDH	-0.68	7.65	ENSG00000109814	UDP-glucose 6-dehydrogenase
FMR1	-0.68	5.36	ENSG00000102081	fragile X mental retardation 1
FAM207A	-0.68	4.46	ENSG00000160256	family with sequence similarity 207 member A
KCNJ11	-0.68	1.37	ENSG00000187486	potassium voltage-gated channel subfamily J member 11
POLR1B	-0.68	6.33	ENSG00000125630	RNA polymerase I subunit B
ST3GAL5	-0.68	3.50	ENSG00000115525	ST3 beta-galactoside alpha-2,3-sialyltransferase 5
TRAP1	-0.68	6.54	ENSG00000126602	TNF receptor associated protein 1
KHK	-0.69	2.91	ENSG00000138030	ketohexokinase
DUSP5	-0.69	2.40	ENSG00000138166	dual specificity phosphatase 5
AIMP2	-0.69	6.12	ENSG00000106305	aminoacyl tRNA synthetase complex interacting multifunctional protein 2
PAQR9	-0.69	5.41	ENSG00000188582	progestin and adipoQ receptor family member 9
PNP	-0.69	6.27	ENSG00000198805	purine nucleoside phosphorylase
SLC1A5	-0.69	7.96	ENSG00000105281	solute carrier family 1 member 5
IDI1	-0.69	6.53	ENSG00000067064	isopentenyl-diphosphate delta isomerase 1
CYP7B1	-0.69	3.17	ENSG00000172817	cytochrome P450 family 7 subfamily B member 1
MAFK	-0.69	5.84	ENSG00000198517	MAF bZIP transcription factor K
LSM11	-0.69	3.44	ENSG00000155858	LSM11, U7 small nuclear RNA associated
YOD1	-0.69	5.89	ENSG00000180667	YOD1 deubiquitinase
PRR22	-0.69	2.81	ENSG00000212123	proline rich 22
SMC5	-0.69	5.89	ENSG00000198887	structural maintenance of chromosomes 5

FOXA3	-0.69	6.21	ENSG00000170608	forkhead box A3
APOH	-0.69	7.89	ENSG00000091583	apolipoprotein H
SLC30A10	-0.69	4.72	ENSG00000196660	solute carrier family 30 member 10
GNB4	-0.69	3.70	ENSG00000114450	G protein subunit beta 4
PPM1L	-0.69	4.43	ENSG00000163590	protein phosphatase, Mg2+/Mn2+ dependent 1L
GCH1	-0.69	4.52	ENSG00000131979	GTP cyclohydrolase 1
EXPH5	-0.69	2.68	ENSG00000110723	exophilin 5
SLC7A6OS	-0.69	2.87	ENSG00000103061	solute carrier family 7 member 6 opposite strand
TMEM170B	-0.70	4.54	ENSG00000205269	transmembrane protein 170B
SCML1	-0.70	4.55	ENSG00000047634	sex comb on midleg-like 1 (Drosophila)
AC007405.6	-0.70	2.28	ENSG00000239467	N/A
MON1A	-0.70	4.10	ENSG00000164077	MON1 homolog A, secretory trafficking associated
SLC23A2	-0.70	6.34	ENSG00000089057	solute carrier family 23 member 2
LRP3	-0.70	6.77	ENSG00000130881	LDL receptor related protein 3
CDS1	-0.70	2.71	ENSG00000163624	CDP-diacylglycerol synthase 1
TTLL11	-0.70	1.34	ENSG00000175764	tubulin tyrosine ligase like 11
ZIC4	-0.70	3.54	ENSG00000174963	Zic family member 4
P2RY11	-0.70	3.09	ENSG00000244165	purinergic receptor P2Y11
ENTPD1-AS1	-0.70	2.37	ENSG00000226688	ENTPD1 antisense RNA 1
WDR35	-0.70	4.46	ENSG00000118965	WD repeat domain 35
RP11-500C11.3	-0.70	4.24	ENSG00000272269	N/A
ADAT3	-0.70	3.46	ENSG00000213638	adenosine deaminase, tRNA specific 3
MSMO1	-0.71	6.19	ENSG00000052802	methylsterol monooxygenase 1
KBTBD8	-0.71	2.00	ENSG00000163376	kelch repeat and BTB domain containing 8
ACACB	-0.71	1.92	ENSG00000076555	acetyl-CoA carboxylase beta
SPAST	-0.71	4.18	ENSG00000021574	spastin
PPAT	-0.71	6.30	ENSG00000128059	phosphoribosyl pyrophosphate amidotransferase
CASC10	-0.71	1.82	ENSG00000204682	cancer susceptibility candidate 10
CCDC80	-0.71	4.04	ENSG00000091986	coiled-coil domain containing 80
GLIS3	-0.71	5.85	ENSG00000107249	GLIS family zinc finger 3
NARS2	-0.71	4.77	ENSG00000137513	asparaginyl-tRNA synthetase 2, mitochondrial (putative)

SDCCAG3	-0.71	6.88	ENSG00000165689	serologically defined colon cancer antigen 3
CEBPG	-0.71	6.92	ENSG00000153879	CCAAT/enhancer binding protein gamma
RNASEH1	-0.71	3.70	ENSG00000171865	ribonuclease H1
CHCHD10	-0.71	5.26	ENSG00000273607	coiled-coil-helix-coiled-coil-helix domain containing 10
NTMT1	-0.71	5.02	ENSG00000148335	N-terminal Xaa-Pro-Lys N-methyltransferase 1
SOSTDC1	-0.71	3.01	ENSG00000171243	sclerostin domain containing 1
CRIM1	-0.71	6.42	ENSG00000277354	cysteine rich transmembrane BMP regulator 1
NF1	-0.71	7.26	ENSG00000196712	neurofibromin 1
NUDT6	-0.71	1.10	ENSG00000170917	nudix hydrolase 6
ACTR3B	-0.71	2.74	ENSG00000133627	ARP3 actin related protein 3 homolog B
PPAN	-0.72	3.12	ENSG00000130810	peter pan homolog (Drosophila)
PET117	-0.72	1.66	ENSG00000232838	PET117 homolog
MFSD3	-0.72	5.14	ENSG00000167700	major facilitator superfamily domain containing 3
CLTCL1	-0.72	5.21	ENSG00000070371	clathrin heavy chain like 1
EPS8L1	-0.72	3.04	ENSG00000131037	EPS8 like 1
MTG1	-0.72	3.82	ENSG00000148824	mitochondrial ribosome associated GTPase 1
CHCHD4	-0.72	4.48	ENSG00000163528	coiled-coil-helix-coiled-coil-helix domain containing 4
EEF2	-0.72	11.88	ENSG00000167658	eukaryotic translation elongation factor 2
YDJC	-0.72	5.57	ENSG00000161179	YdjC homolog (bacterial)
SLC5A3	-0.72	5.82	ENSG00000198743	solute carrier family 5 member 3
E2F7	-0.72	5.12	ENSG00000165891	E2F transcription factor 7
PGS1	-0.72	4.91	ENSG00000087157	phosphatidylglycerophosphate synthase 1
PEBP1	-0.72	7.99	ENSG00000089220	phosphatidylethanolamine binding protein 1
ODC1	-0.72	7.18	ENSG00000115758	ornithine decarboxylase 1
LYST	-0.72	3.88	ENSG00000143669	lysosomal trafficking regulator
CCDC85B	-0.72	4.07	ENSG00000175602	coiled-coil domain containing 85B
ALOX12	-0.72	1.59	ENSG00000108839	arachidonate 12-lipoxygenase, 12S type

PRR7	-0.72	2.75	ENSG00000131188	proline rich 7, synaptic
RSAD1	-0.72	4.54	ENSG00000136444	radical S-adenosyl methionine domain containing 1
DDX31	-0.72	5.42	ENSG00000125485	DEAD-box helicase 31
ANKRD16	-0.72	2.57	ENSG00000134461	ankyrin repeat domain 16
PHLDB2	-0.73	6.72	ENSG00000144824	pleckstrin homology like domain family B member 2
FAM173B	-0.73	4.22	ENSG00000150756	family with sequence similarity 173 member B
LRRC58	-0.73	6.46	ENSG00000163428	leucine rich repeat containing 58
AGRN	-0.73	9.33	ENSG00000188157	agrin
UNKL	-0.73	5.03	ENSG00000059145	unkempt family like zinc finger
PPIF	-0.73	6.76	ENSG00000108179	peptidylprolyl isomerase F
PLD6	-0.73	2.59	ENSG00000179598	phospholipase D family member 6
POMK	-0.73	1.96	ENSG00000185900	protein-O-mannose kinase
CAPN10-AS1	-0.73	1.35	ENSG00000260942	CAPN10 antisense RNA 1 (head to head)
UHRF1	-0.73	6.10	ENSG00000276043	ubiquitin like with PHD and ring finger domains 1
C8orf76	-0.73	1.64	ENSG00000189376	chromosome 8 open reading frame 76
HMGCR	-0.73	7.66	ENSG00000113161	3-hydroxy-3-methylglutaryl-CoA reductase
ARHGEF26	-0.73	5.83	ENSG00000114790	Rho guanine nucleotide exchange factor 26
ACACA	-0.73	7.58	ENSG00000278540	acetyl-CoA carboxylase alpha
ASS1	-0.73	5.38	ENSG00000130707	argininosuccinate synthase 1
AC093673.5	-0.73	2.49	ENSG00000232533	N/A
MNX1	-0.74	3.33	ENSG00000130675	motor neuron and pancreas homeobox 1
DCP2	-0.74	5.98	ENSG00000172795	decapping mRNA 2
DCAF4	-0.74	2.57	ENSG00000119599	DDB1 and CUL4 associated factor 4
SYDE1	-0.74	5.56	ENSG00000105137	synapse defective Rho GTPase homolog 1
BLOC1S4	-0.74	3.95	ENSG00000186222	biogenesis of lysosomal organelles complex 1 subunit 4
PUS7	-0.74	5.43	ENSG00000091127	pseudouridylate synthase 7 (putative)
LIPC	-0.74	5.33	ENSG00000166035	lipase C, hepatic type
TRAPPC6A	-0.74	2.39	ENSG00000007255	trafficking protein particle complex 6A

UTP20	-0.74	6.38	ENSG00000120800	UTP20, small subunit processome component
TFDP1	-0.74	8.35	ENSG00000198176	transcription factor Dp-1
NR2C2AP	-0.74	3.85	ENSG00000184162	nuclear receptor 2C2 associated protein
TSPAN4	-0.74	6.03	ENSG00000214063	tetraspanin 4
RRP12	-0.74	6.08	ENSG00000052749	ribosomal RNA processing 12 homolog
RARRES2	-0.74	5.32	ENSG00000106538	retinoic acid receptor responder 2
CDC25A	-0.74	5.63	ENSG00000164045	cell division cycle 25A
MIR17HG	-0.75	3.58	ENSG00000215417	miR-17-92a-1 cluster host gene
APOM	-0.75	3.28	ENSG00000227567	apolipoprotein M
FBXL6	-0.75	4.90	ENSG00000182325	F-box and leucine rich repeat protein 6
NSDHL	-0.75	6.07	ENSG00000147383	NAD(P) dependent steroid dehydrogenase-like
PWP2	-0.75	6.14	ENSG00000241945	PWP2 periodic tryptophan protein homolog (yeast)
NDUFS7	-0.75	5.66	ENSG00000115286	NADH:ubiquinone oxidoreductase core subunit S7
RGS20	-0.75	1.32	ENSG00000147509	regulator of G-protein signaling 20
NPM3	-0.75	4.99	ENSG00000107833	nucleophosmin/nucleoplasmin 3
WDR81	-0.75	6.44	ENSG00000167716	WD repeat domain 81
MVK	-0.75	5.33	ENSG00000110921	mevalonate kinase
RP13-1032I1.7	-0.75	1.65	ENSG00000262049	N/A
ZNF281	-0.75	7.36	ENSG00000162702	zinc finger protein 281
ITPRIP	-0.76	3.26	ENSG00000148841	inositol 1,4,5-trisphosphate receptor interacting protein
SLC20A2	-0.76	7.46	ENSG00000168575	solute carrier family 20 member 2
NPAS3	-0.76	1.37	ENSG00000151322	neuronal PAS domain protein 3
ZNF8	-0.76	1.37	ENSG00000278129	zinc finger protein 8
POLR1E	-0.76	4.63	ENSG00000137054	RNA polymerase I subunit E
ZHX1	-0.76	6.36	ENSG00000165156	zinc fingers and homeoboxes 1
AC004967.7	-0.76	1.54	ENSG00000243554	N/A
DUS3L	-0.76	5.66	ENSG00000141994	dihydrouridine synthase 3 like
SLC7A2	-0.76	6.83	ENSG00000003989	solute carrier family 7 member 2
ANXA3	-0.76	6.99	ENSG00000138772	annexin A3
ZNF714	-0.76	5.04	ENSG00000160352	zinc finger protein 714
DGKD	-0.76	5.69	ENSG00000280873	diacylglycerol kinase delta

PAM16	-0.76	1.58	ENSG00000217930	presequence translocase associated motor 16 homolog
ATP6V1E2	-0.76	1.86	ENSG00000250565	ATPase H+ transporting V1 subunit E2
FTLP3	-0.76	2.77	ENSG00000226608	ferritin light chain pseudogene 3
TMEM160	-0.77	4.03	ENSG00000130748	transmembrane protein 160
CD3EAP	-0.77	3.24	ENSG00000117877	CD3e molecule associated protein
DNAJA3	-0.77	6.39	ENSG00000276726	DnaJ heat shock protein family (Hsp40) member A3
RAP2B	-0.77	6.56	ENSG00000181467	RAP2B, member of RAS oncogene family
GCSH	-0.77	2.59	ENSG00000140905	glycine cleavage system protein H
JAG2	-0.77	5.60	ENSG00000184916	jagged 2
SERPIND1	-0.77	6.57	ENSG00000099937	serpin family D member 1
MOSPD1	-0.77	4.95	ENSG00000101928	motile sperm domain containing 1
C2	-0.77	2.96	ENSG00000231543	complement C2
SNHG8	-0.77	4.05	ENSG00000269893	small nucleolar RNA host gene 8
WDR4	-0.78	4.17	ENSG00000160193	WD repeat domain 4
HIST1H2AG	-0.78	1.34	ENSG00000196787	histone cluster 1 H2A family member g
LYRM1	-0.78	5.31	ENSG00000102897	LYR motif containing 1
WTIP	-0.78	5.17	ENSG00000142279	Wilms tumor 1 interacting protein
IKBKG	-0.78	2.65	ENSG00000269335	inhibitor of kappa light polypeptide gene enhancer in B-cells, kinase gamma
RP11-834C11.4	-0.78	5.61	ENSG00000250742	N/A
CXCL16	-0.78	6.99	ENSG00000161921	C-X-C motif chemokine ligand 16
ZNF556	-0.78	1.55	ENSG00000172000	zinc finger protein 556
IDH3A	-0.78	3.50	ENSG00000166411	isocitrate dehydrogenase 3 (NAD(+)) alpha
AAK1	-0.78	4.13	ENSG00000115977	AP2 associated kinase 1
MAP3K14	-0.78	3.79	ENSG00000006062	mitogen-activated protein kinase kinase kinase 14
CHML	-0.78	5.96	ENSG00000203668	CHM like, Rab escort protein 2
RAMP2-AS1	-0.79	1.20	ENSG00000197291	RAMP2 antisense RNA 1
RANGRF	-0.79	1.59	ENSG00000108961	RAN guanine nucleotide release factor
COMTD1	-0.79	3.36	ENSG00000165644	catechol-O-methyltransferase domain containing 1
CISH	-0.79	2.49	ENSG00000114737	cytokine inducible SH2 containing protein

ZNF467	-0.79	1.43	ENSG00000181444	zinc finger protein 467
ZNF324B	-0.79	2.84	ENSG00000249471	zinc finger protein 324B
URB2	-0.79	5.87	ENSG00000135763	URB2 ribosome biogenesis 2 homolog (S. cerevisiae)
DHRS13	-0.79	2.68	ENSG00000167536	dehydrogenase/reductase 13
ADAMTS19	-0.79	3.05	ENSG00000145808	ADAM metallopeptidase with thrombospondin type 1 motif 19
LIMK1	-0.79	5.73	ENSG00000106683	LIM domain kinase 1
SLC2A1	-0.79	4.81	ENSG00000117394	solute carrier family 2 member 1
CRYM	-0.79	1.25	ENSG00000103316	crystallin mu
TYSND1	-0.79	4.56	ENSG00000156521	trypsin domain containing 1
PYCR1	-0.79	6.30	ENSG00000183010	pyrroline-5-carboxylate reductase 1
AMN	-0.79	4.21	ENSG00000166126	amnion associated transmembrane protein
CTB-55O6.12	-0.79	1.48	ENSG00000267169	N/A
SIGMAR1	-0.79	6.31	ENSG00000147955	sigma non-opioid intracellular receptor 1
ASB9	-0.79	2.63	ENSG00000102048	ankyrin repeat and SOCS box containing 9
MAK16	-0.80	5.89	ENSG00000198042	MAK16 homolog
STOM	-0.80	6.25	ENSG00000148175	stomatin
INSIG1	-0.80	5.09	ENSG00000186480	insulin induced gene 1
ZYG11A	-0.80	2.82	ENSG00000203995	zyg-11 family member A, cell cycle regulator
POLR3K	-0.80	3.82	ENSG00000161980	RNA polymerase III subunit K
CHD9	-0.80	3.52	ENSG00000177200	chromodomain helicase DNA binding protein 9
TRIM56	-0.80	4.15	ENSG00000169871	tripartite motif containing 56
PCYT2	-0.80	7.09	ENSG00000185813	phosphate cytidylyltransferase 2, ethanolamine
TATDN2P2	-0.80	2.59	ENSG00000218226	TatD DNase domain containing 2 pseudogene 2
JAG1	-0.80	4.36	ENSG00000101384	jagged 1
SLFNL1	-0.80	1.10	ENSG00000171790	schlafen like 1
MRPS24	-0.80	1.75	ENSG00000062582	mitochondrial ribosomal protein S24
FAM110C	-0.80	4.76	ENSG00000184731	family with sequence similarity 110 member C
NUDT8	-0.80	2.06	ENSG00000167799	nudix hydrolase 8

SCML2	-0.81	3.67	ENSG00000102098	sex comb on midleg-like 2 (Drosophila)
PUS7L	-0.81	3.92	ENSG00000129317	pseudouridylate synthase 7 like
PUS1	-0.81	4.93	ENSG00000177192	pseudouridylate synthase 1
CPN2	-0.81	1.79	ENSG00000178772	carboxypeptidase N subunit 2
AASS	-0.81	3.07	ENSG00000008311	aminoadipate-semialdehyde synthase
FAM72D	-0.81	1.16	ENSG00000215784	family with sequence similarity 72 member D
ACSL3	-0.81	8.56	ENSG00000123983	acyl-CoA synthetase long-chain family member 3
NOC3L	-0.81	5.40	ENSG00000173145	NOC3 like DNA replication regulator
UGT2B4	-0.81	4.22	ENSG00000156096	UDP glucuronosyltransferase family 2 member B4
FBLN5	-0.81	3.83	ENSG00000140092	fibulin 5
AMOT	-0.81	4.53	ENSG00000126016	angiomotin
MYO9A	-0.81	4.07	ENSG00000066933	myosin IXA
SLC35E2B	-0.81	6.54	ENSG00000189339	solute carrier family 35 member E2B
BOP1	-0.82	5.87	ENSG00000261236	block of proliferation 1
S1PR2	-0.82	2.30	ENSG00000267534	sphingosine-1-phosphate receptor 2
ING5	-0.82	5.13	ENSG00000168395	inhibitor of growth family member 5
KLF11	-0.82	1.73	ENSG00000172059	Kruppel like factor 11
PIP4K2B	-0.82	6.87	ENSG00000276293	phosphatidylinositol-5-phosphate 4-kinase type 2 beta
SDC2	-0.82	6.93	ENSG00000169439	syndecan 2
CFD	-0.82	3.16	ENSG00000274619	complement factor D
TRIM71	-0.82	7.13	ENSG00000206557	tripartite motif containing 71
TLN2	-0.83	5.46	ENSG00000171914	talin 2
RASSF9	-0.83	2.94	ENSG00000198774	Ras association domain family member 9
ZFHX4	-0.83	6.22	ENSG00000091656	zinc finger homeobox 4
SSTR5	-0.83	2.16	ENSG00000162009	somatostatin receptor 5
ASIC1	-0.83	1.06	ENSG00000110881	acid sensing ion channel subunit 1
AMZ1	-0.83	2.45	ENSG00000174945	archaelysin family metallopeptidase 1
HOXA-AS2	-0.83	2.57	ENSG00000253552	HOXA cluster antisense RNA 2
CCDC86	-0.83	6.42	ENSG00000110104	coiled-coil domain containing 86
ABCB10	-0.84	5.19	ENSG00000135776	ATP binding cassette subfamily B member 10

PHKA2	-0.84	5.90	ENSG00000044446	phosphorylase kinase regulatory subunit alpha 2
FLNB	-0.84	8.53	ENSG00000136068	filamin B
KNG1	-0.84	4.63	ENSG00000113889	kininogen 1
LIN7A	-0.84	5.81	ENSG00000111052	lin-7 homolog A, crumbs cell polarity complex component
PITPNM3	-0.84	5.34	ENSG00000091622	PITPNM family member 3
NME3	-0.84	4.00	ENSG00000103024	NME/NM23 nucleoside diphosphate kinase 3
BOK	-0.84	5.30	ENSG00000176720	BOK, BCL2 family apoptosis regulator
C17orf58	-0.84	2.93	ENSG00000186665	chromosome 17 open reading frame 58
SYT7	-0.84	3.70	ENSG00000011347	synaptotagmin 7
SRGAP2B	-0.84	1.97	ENSG00000196369	SLIT-ROBO Rho GTPase activating protein 2B
RPS23P8	-0.84	1.23	ENSG00000230629	ribosomal protein S23 pseudogene 8
PHYH	-0.85	4.12	ENSG00000107537	phytanoyl-CoA 2-hydroxylase
DCHS2	-0.85	3.94	ENSG00000197410	dachsous cadherin-related 2
RDH13	-0.85	4.00	ENSG00000274418	retinol dehydrogenase 13
FTCD	-0.85	1.34	ENSG00000160282	formimidoyltransferase cyclodeaminase
TMEM192	-0.85	4.83	ENSG00000170088	transmembrane protein 192
GOLT1A	-0.85	5.80	ENSG00000174567	golgi transport 1A
GLCCI1	-0.85	6.13	ENSG00000106415	glucocorticoid induced 1
FASN	-0.86	10.43	ENSG00000169710	fatty acid synthase
C17orf82	-0.86	1.39	ENSG00000187013	chromosome 17 open reading frame 82
IGFBP4	-0.86	1.39	ENSG00000141753	insulin like growth factor binding protein 4
ROBO2	-0.86	2.15	ENSG00000185008	roundabout guidance receptor 2
PKP2	-0.86	6.04	ENSG00000057294	plakophilin 2
AC093162.5	-0.86	1.43	ENSG00000246575	N/A
MPC1	-0.86	5.58	ENSG00000060762	mitochondrial pyruvate carrier 1
NOSTRIN	-0.86	3.70	ENSG00000163072	nitric oxide synthase trafficking
ADCY10	-0.86	3.20	ENSG00000143199	adenylate cyclase 10, soluble
HPX	-0.86	4.73	ENSG00000110169	hemopexin
P2RY2	-0.87	1.52	ENSG00000175591	purinergic receptor P2Y2
KLHL29	-0.87	5.56	ENSG00000119771	kelch like family member 29
NUDT17	-0.87	2.93	ENSG00000186364	nudix hydrolase 17
RASAL2	-0.87	6.08	ENSG00000075391	RAS protein activator like 2

FRRS1	-0.87	4.20	ENSG00000156869	ferric chelate reductase 1
L3HYPDH	-0.87	3.52	ENSG00000126790	trans-L-3-hydroxyproline dehydratase
NUDT14	-0.87	3.55	ENSG00000183828	nudix hydrolase 14
SHANK2	-0.87	5.00	ENSG00000162105	SH3 and multiple ankyrin repeat domains 2
CTU1	-0.87	3.64	ENSG00000142544	cytosolic thiouridylase subunit 1
SLC16A1	-0.87	6.39	ENSG00000281917	solute carrier family 16 member 1
RAPH1	-0.87	2.41	ENSG00000173166	Ras association (RalGDS/AF-6) and pleckstrin homology domains 1
MARS2	-0.87	4.99	ENSG00000247626	methionyl-tRNA synthetase 2, mitochondrial
CD37	-0.87	3.83	ENSG00000104894	CD37 molecule
PTAR1	-0.88	6.08	ENSG00000188647	protein prenyltransferase alpha subunit repeat containing 1
SULT2A1	-0.88	1.99	ENSG00000105398	sulfotransferase family 2A member 1
FDPS	-0.88	7.61	ENSG00000160752	farnesyl diphosphate synthase
SORL1	-0.88	4.57	ENSG00000137642	sortilin related receptor 1
MACROD2	-0.88	1.35	ENSG00000172264	MACRO domain containing 2
MRPS26	-0.88	5.74	ENSG00000125901	mitochondrial ribosomal protein S26
MTHFD2	-0.88	6.18	ENSG00000065911	methylenetetrahydrofolate dehydrogenase (NADP+ dependent) 2, methenyltetrahydrofolate cyclohydrolase
HEG1	-0.88	6.22	ENSG00000173706	heart development protein with EGF like domains 1
BDH1	-0.88	2.37	ENSG00000275544	3-hydroxybutyrate dehydrogenase, type 1
SNHG7	-0.88	4.65	ENSG00000233016	small nucleolar RNA host gene 7
MASP1	-0.88	5.60	ENSG00000127241	mannan binding lectin serine peptidase 1
CGA	-0.89	3.31	ENSG00000135346	glycoprotein hormones, alpha polypeptide
CYTH3	-0.89	5.56	ENSG00000008256	cytohesin 3
IGF2-AS	-0.89	2.91	ENSG00000099869	IGF2 antisense RNA
ADORA2A-AS1	-0.89	1.04	ENSG00000178803	ADORA2A antisense RNA 1
TLL1	-0.89	1.58	ENSG00000038295	tolloid like 1
SESN3	-0.89	5.88	ENSG00000149212	sestrin 3
HIST1H2BJ	-0.89	1.10	ENSG00000124635	histone cluster 1 H2B family member j

ITPKA	-0.90	3.78	ENSG00000137825	inositol-trisphosphate 3-kinase A
HBQ1	-0.90	1.22	ENSG00000086506	hemoglobin subunit theta 1
CCND1	-0.90	6.71	ENSG00000110092	cyclin D1
SMIM14	-0.90	4.45	ENSG00000163683	small integral membrane protein 14
ZCCHC24	-0.90	4.59	ENSG00000165424	zinc finger CCHC-type containing 24
AGMAT	-0.90	6.33	ENSG00000116771	agmatinase
SLCO2B1	-0.90	1.81	ENSG00000137491	solute carrier organic anion transporter family member 2B1
NT5C2	-0.90	5.78	ENSG00000076685	5'-nucleotidase, cytosolic II
GLDC	-0.90	6.66	ENSG00000178445	glycine decarboxylase
SLC7A1	-0.91	6.42	ENSG00000139514	solute carrier family 7 member 1
AGO2	-0.91	5.34	ENSG00000123908	argonaute 2, RISC catalytic component
TST	-0.91	4.44	ENSG00000128311	thiosulfate sulfurtransferase
MTX3	-0.91	3.88	ENSG00000177034	metaxin 3
HSPB8	-0.91	4.03	ENSG00000152137	heat shock protein family B (small) member 8
WNK4	-0.91	3.36	ENSG00000126562	WNK lysine deficient protein kinase 4
SERTAD1	-0.91	2.43	ENSG00000197019	SERTA domain containing 1
TNNC1	-0.91	3.42	ENSG00000114854	troponin C1, slow skeletal and cardiac type
XXYLT1	-0.91	5.82	ENSG00000173950	xyloside xylosyltransferase 1
PAXIP1-AS1	-0.91	2.50	ENSG00000273344	PAXIP1 antisense RNA 1 (head to head)
EPAS1	-0.92	8.25	ENSG00000116016	endothelial PAS domain protein 1
AGXT	-0.92	1.79	ENSG00000172482	alanine-glyoxylate aminotransferase
AC011290.5	-0.92	1.00	ENSG00000236015	N/A
RP11-894P9.1	-0.92	1.00	ENSG00000246451	N/A
GFOD1	-0.92	1.99	ENSG00000145990	glucose-fructose oxidoreductase domain containing 1
CICP14	-0.92	1.12	ENSG00000281490	capicua transcriptional repressor pseudogene 14
APOC2	-0.93	1.23	ENSG00000234906	apolipoprotein C2
TRPC4	-0.93	1.23	ENSG00000133107	transient receptor potential cation channel subfamily C member 4
CCDC85C	-0.93	5.72	ENSG00000205476	coiled-coil domain containing 85C
HK2	-0.93	7.06	ENSG00000159399	hexokinase 2
PEG10	-0.93	5.27	ENSG00000242265	paternally expressed 10
RGS2	-0.93	5.67	ENSG00000116741	regulator of G-protein signaling 2

BAIAP2	-0.93	4.09	ENSG00000175866	BAI1 associated protein 2
C2orf54	-0.93	4.33	ENSG00000172478	chromosome 2 open reading frame 54
GULP1	-0.93	4.16	ENSG00000144366	GULP, engulfment adaptor PTB domain containing 1
GLUD1	-0.93	7.34	ENSG00000148672	glutamate dehydrogenase 1
ACAT2	-0.94	7.01	ENSG00000120437	acetyl-CoA acetyltransferase 2
NUP43	-0.94	5.92	ENSG00000120253	nucleoporin 43
REV3L	-0.94	4.68	ENSG00000009413	REV3 like, DNA directed polymerase zeta catalytic subunit
DDIT4L	-0.94	2.15	ENSG00000145358	DNA damage inducible transcript 4 like
MTMR4	-0.95	6.25	ENSG00000108389	myotubularin related protein 4
CXorf57	-0.95	3.00	ENSG00000147231	chromosome X open reading frame 57
SCN9A	-0.95	6.96	ENSG00000169432	sodium voltage-gated channel alpha subunit 9
RP11-449P15.2	-0.95	3.14	ENSG00000273151	N/A
EPPK1	-0.95	5.36	ENSG00000261150	epiplakin 1
BCL2L14	-0.95	1.02	ENSG00000281449	BCL2 like 14
NAB2	-0.95	4.30	ENSG00000166886	NGFI-A binding protein 2
FUT9	-0.95	1.20	ENSG00000172461	fucosyltransferase 9
MRPL23-AS1	-0.96	1.45	ENSG00000226416	MRPL23 antisense RNA 1
NEU3	-0.96	2.91	ENSG00000162139	neuraminidase 3
MVD	-0.96	6.82	ENSG00000167508	mevalonate diphosphate decarboxylase
FBXL18	-0.96	5.31	ENSG00000155034	F-box and leucine rich repeat protein 18
RPS6KB1	-0.96	5.54	ENSG00000108443	ribosomal protein S6 kinase B1
RRS1	-0.97	5.07	ENSG00000179041	ribosome biogenesis regulator homolog
LSS	-0.97	7.79	ENSG00000281289	lanosterol synthase (2,3-oxidosqualene-lanosterol cyclase)
MBP	-0.97	5.58	ENSG00000197971	myelin basic protein
URB1	-0.97	6.22	ENSG00000142207	URB1 ribosome biogenesis 1 homolog (S. cerevisiae)
MARC1	-0.97	6.07	ENSG00000186205	mitochondrial amidoxime reducing component 1
RNF152	-0.97	2.20	ENSG00000176641	ring finger protein 152
PCK2	-0.97	4.35	ENSG00000100889	phosphoenolpyruvate carboxykinase 2, mitochondrial
EDN1	-0.97	4.52	ENSG00000078401	endothelin 1
RP11-469A15.2	-0.97	1.79	ENSG00000230623	N/A

C9orf40	-0.98	4.05	ENSG00000135045	chromosome 9 open reading frame 40
SCNN1A	-0.98	1.55	ENSG00000111319	sodium channel epithelial 1 alpha subunit
C1orf109	-0.98	3.59	ENSG00000116922	chromosome 1 open reading frame 109
KBTBD6	-0.98	3.84	ENSG00000165572	kelch repeat and BTB domain containing 6
NCOA2	-0.98	6.60	ENSG00000140396	nuclear receptor coactivator 2
MCC	-0.98	5.32	ENSG00000171444	mutated in colorectal cancers
CRNDE	-0.98	1.22	ENSG00000245694	colorectal neoplasia differentially expressed (non-protein coding)
P2RY1	-0.98	1.22	ENSG00000169860	purinergic receptor P2Y1
ORM2	-0.98	3.55	ENSG00000228278	orosomucoid 2
CDH4	-0.99	1.10	ENSG00000179242	cadherin 4
PDXP	-0.99	1.87	ENSG00000241360	pyridoxal phosphatase
ABCA1	-0.99	5.06	ENSG00000165029	ATP binding cassette subfamily A member 1
PSAT1	-0.99	7.13	ENSG00000135069	phosphoserine aminotransferase 1
NQO1	-1.00	3.32	ENSG00000181019	NAD(P)H quinone dehydrogenase 1
SPIN4	-1.00	3.66	ENSG00000186767	spindlin family member 4
PCDH9	-1.00	4.68	ENSG00000184226	protocadherin 9
RP11-264B17.2	-1.01	1.34	ENSG00000260853	N/A
ST3GAL6	-1.01	3.65	ENSG00000064225	ST3 beta-galactoside alpha-2,3-sialyltransferase 6
ORM1	-1.01	4.93	ENSG00000229314	orosomucoid 1
MCOLN2	-1.01	1.74	ENSG00000153898	mucolipin 2
CD14	-1.02	1.12	ENSG00000170458	CD14 molecule
GCLM	-1.02	4.69	ENSG00000023909	glutamate-cysteine ligase modifier subunit
SHPK	-1.02	5.66	ENSG00000197417	sedoheptulokinase
NADK2	-1.05	7.25	ENSG00000152620	NAD kinase 2, mitochondrial
TRIP13	-1.05	6.05	ENSG00000071539	thyroid hormone receptor interactor 13
ADCY7	-1.05	3.17	ENSG00000121281	adenylate cyclase 7
PROSER2	-1.05	5.25	ENSG00000148426	proline and serine rich 2
GPD1L	-1.05	5.88	ENSG00000152642	glycerol-3-phosphate dehydrogenase 1-like
GNL3L	-1.05	4.12	ENSG00000130119	G protein nucleolar 3 like
C12orf66	-1.06	2.20	ENSG00000174206	chromosome 12 open reading frame 66

MMP16	-1.06	2.70	ENSG00000156103	matrix metallopeptidase 16
KCNAB2	-1.06	3.91	ENSG00000069424	potassium voltage-gated channel subfamily A regulatory beta subunit 2
SYBU	-1.06	3.13	ENSG00000147642	syntabulin
NOC2L	-1.06	7.04	ENSG00000188976	NOC2 like nucleolar associated transcriptional repressor
RBMS2	-1.06	4.19	ENSG00000076067	RNA binding motif single stranded interacting protein 2
RP11-80H5.5	-1.06	1.61	ENSG00000249962	N/A
ZNF696	-1.07	3.56	ENSG00000185730	zinc finger protein 696
SPSB4	-1.07	2.42	ENSG00000175093	splA/ryanodine receptor domain and SOCS box containing 4
C1orf64	-1.07	2.18	ENSG00000183888	chromosome 1 open reading frame 64
MICAL2	-1.08	4.90	ENSG00000133816	microtubule associated monooxygenase, calponin and LIM domain containing 2
TXNIP	-1.08	4.74	ENSG00000265972	thioredoxin interacting protein
SGTB	-1.08	3.15	ENSG00000197860	small glutamine rich tetratricopeptide repeat containing beta
CCAT1	-1.08	3.95	ENSG00000247844	colon cancer associated transcript 1 (non-protein coding)
GDA	-1.08	1.38	ENSG00000119125	guanine deaminase
AIM1	-1.08	4.76	ENSG00000112297	absent in melanoma 1
LGI2	-1.08	3.19	ENSG00000153012	leucine rich repeat LGI family member 2
CPS1	-1.08	1.04	ENSG00000021826	carbamoyl-phosphate synthase 1
MDGA1	-1.08	1.04	ENSG00000112139	MAM domain containing glycosylphosphatidylinositol anchor 1
KRT23	-1.09	8.66	ENSG00000108244	keratin 23
APOB	-1.09	11.77	ENSG00000084674	apolipoprotein B
DTNA	-1.09	5.73	ENSG00000134769	dystrobrevin alpha
APOBEC3F	-1.09	2.10	ENSG00000128394	apolipoprotein B mRNA editing enzyme catalytic subunit 3F
ADAM11	-1.10	1.49	ENSG00000073670	ADAM metallopeptidase domain 11
MIR503HG	-1.10	1.23	ENSG00000223749	MIR503 host gene
PDGFRB	-1.10	3.00	ENSG00000113721	platelet derived growth factor receptor beta
CHST13	-1.10	6.04	ENSG00000180767	carbohydrate sulfotransferase 13

MGAT4A	-1.11	6.16	ENSG00000071073	mannosyl (alpha-1,3-)-glycoprotein beta-1,4-N-acetylglucosaminyltransferase, isozyme A
METTL3	-1.11	6.03	ENSG00000165819	methyltransferase like 3
AC006273.5	-1.11	1.12	ENSG00000267530	N/A
LRP8	-1.12	3.56	ENSG00000157193	LDL receptor related protein 8
C8G	-1.13	3.94	ENSG00000176919	complement C8 gamma chain
AHNAK	-1.13	7.19	ENSG00000124942	AHNAK nucleoprotein
MYO10	-1.13	5.55	ENSG00000145555	myosin X
C17orf51	-1.14	2.87	ENSG00000212719	chromosome 17 open reading frame 51
REEP4	-1.14	5.48	ENSG00000168476	receptor accessory protein 4
LIN28B	-1.14	6.19	ENSG00000187772	lin-28 homolog B
PDSS1	-1.14	3.89	ENSG00000148459	prenyl (decaprenyl) diphosphate synthase, subunit 1
AP1M2	-1.15	2.68	ENSG00000129354	adaptor related protein complex 1 mu 2 subunit
FAM150A	-1.16	1.87	ENSG00000196711	family with sequence similarity 150 member A
FAM46B	-1.16	1.58	ENSG00000158246	family with sequence similarity 46 member B
ABCG2	-1.16	2.05	ENSG00000118777	ATP binding cassette subfamily G member 2 (Junior blood group)
APOOL	-1.17	2.65	ENSG00000155008	apolipoprotein O like
ZC3HAV1L	-1.17	2.89	ENSG00000146858	zinc finger CCCH-type containing, antiviral 1 like
NCR3LG1	-1.17	2.63	ENSG00000188211	natural killer cell cytotoxicity receptor 3 ligand 1
HAGHL	-1.17	1.49	ENSG00000103253	hydroxyacylglutathione hydrolase-like
CEBPA-AS1	-1.17	2.21	ENSG00000267296	CEBPA antisense RNA 1 (head to head)
BCAT1	-1.17	5.89	ENSG00000060982	branched chain amino acid transaminase 1
SLC39A5	-1.18	4.02	ENSG00000139540	solute carrier family 39 member 5
CCSER1	-1.18	2.49	ENSG00000184305	coiled-coil serine rich protein 1
SLC43A3	-1.18	6.87	ENSG00000134802	solute carrier family 43 member 3
PLCD3	-1.19	5.01	ENSG00000161714	phospholipase C delta 3
GPX2	-1.19	5.90	ENSG00000176153	glutathione peroxidase 2

VASN	-1.19	4.88	ENSG00000168140	vasorin
SOWAHC	-1.19	5.57	ENSG00000198142	sosondowah ankyrin repeat domain family member C
SKP2	-1.20	7.58	ENSG00000145604	S-phase kinase associated protein 2
WDR66	-1.20	3.83	ENSG00000158023	WD repeat domain 66
CA2	-1.20	1.82	ENSG00000104267	carbonic anhydrase 2
FOXJ1	-1.20	4.97	ENSG00000129654	forkhead box J1
AIM1L	-1.20	3.44	ENSG00000176092	absent in melanoma 1-like
MLXIPL	-1.21	4.53	ENSG00000009950	MLX interacting protein like
TTR	-1.21	5.57	ENSG00000118271	transthyretin
FGF19	-1.21	3.71	ENSG00000162344	fibroblast growth factor 19
CNNM1	-1.21	6.02	ENSG00000119946	cyclin and CBS domain divalent metal cation transport mediator 1
ENDOG	-1.21	3.16	ENSG00000167136	endonuclease G
WNT3	-1.21	3.18	ENSG00000277626	Wnt family member 3
RTN4R	-1.21	4.33	ENSG00000040608	reticulon 4 receptor
VWA5B2	-1.21	3.16	ENSG00000145198	von Willebrand factor A domain containing 5B2
KLF15	-1.21	3.55	ENSG00000163884	Kruppel like factor 15
RBP4	-1.21	8.12	ENSG00000138207	retinol binding protein 4
MEIOB	-1.21	4.06	ENSG00000282650	meiosis specific with OB domains
UCA1	-1.22	4.66	ENSG00000273782	Urothelial cancer associated 1 conserved region
TMEM52	-1.22	1.37	ENSG00000178821	transmembrane protein 52
SLC7A5	-1.22	8.15	ENSG00000103257	solute carrier family 7 member 5
SEMA5A	-1.23	4.22	ENSG00000112902	semaphorin 5A
ADARB1	-1.23	4.91	ENSG00000197381	adenosine deaminase, RNA specific B1
CHST9	-1.24	3.81	ENSG00000154080	carbohydrate sulfotransferase 9
HMGCS1	-1.24	8.77	ENSG00000112972	3-hydroxy-3-methylglutaryl-CoA synthase 1
TMEM238	-1.25	2.20	ENSG00000233493	transmembrane protein 238
GPT	-1.25	1.34	ENSG00000167701	glutamic--pyruvic transaminase
LFNG	-1.25	2.01	ENSG00000106003	LFNG O-fucosylpeptide 3-beta-N-acetylglucosaminyltransferase
RP11-259N19.1	-1.26	2.05	ENSG00000272711	N/A
KANK4	-1.26	2.92	ENSG00000132854	KN motif and ankyrin repeat domains 4
ARHGDIB	-1.26	1.40	ENSG00000111348	Rho GDP dissociation inhibitor beta

HAL	-1.27	3.97	ENSG00000084110	histidine ammonia-lyase
TMC6	-1.27	6.38	ENSG00000141524	transmembrane channel like 6
SLC19A3	-1.28	5.02	ENSG00000135917	solute carrier family 19 member 3
TEAD4	-1.29	5.37	ENSG00000197905	TEA domain transcription factor 4
MOGAT3	-1.29	1.52	ENSG00000106384	monoacylglycerol O-acyltransferase 3
DDIT4	-1.29	3.72	ENSG00000168209	DNA damage inducible transcript 4
ALB	-1.29	13.58	ENSG00000163631	albumin
LONRF1	-1.30	2.21	ENSG00000154359	LON peptidase N-terminal domain and ring finger 1
KLF2	-1.30	3.96	ENSG00000127528	Kruppel like factor 2
KRT19	-1.32	8.23	ENSG00000171345	keratin 19
CHAC1	-1.32	4.35	ENSG00000128965	ChaC glutathione specific gamma-glutamylcyclotransferase 1
ZBED6	-1.32	3.70	ENSG00000257315	zinc finger BED-type containing 6
DUSP4	-1.33	4.60	ENSG00000120875	dual specificity phosphatase 4
B3GALT2	-1.33	1.14	ENSG00000162630	beta-1,3-galactosyltransferase 2
SYNE3	-1.34	1.21	ENSG00000176438	spectrin repeat containing nuclear envelope family member 3
MRM1	-1.35	3.96	ENSG00000274853	mitochondrial rRNA methyltransferase 1
SYDE2	-1.35	2.40	ENSG00000097096	synapse defective Rho GTPase homolog 2
INPP5F	-1.35	4.30	ENSG00000198825	inositol polyphosphate-5-phosphatase F
SLC22A15	-1.35	4.01	ENSG00000163393	solute carrier family 22 member 15
IGDCC3	-1.36	6.28	ENSG00000174498	immunoglobulin superfamily DCC subclass member 3
NBEAL1	-1.37	4.56	ENSG00000144426	neurobeachin like 1
SARDH	-1.37	3.35	ENSG00000123453	sarcosine dehydrogenase
PDP2	-1.39	3.97	ENSG00000172840	pyruvate dehyrogenase phosphatase catalytic subunit 2
LEAP2	-1.39	1.25	ENSG00000164406	liver enriched antimicrobial peptide 2
ARHGAP23	-1.39	5.93	ENSG00000273780	Rho GTPase activating protein 23
NAT8	-1.40	4.20	ENSG00000144035	N-acetyltransferase 8 (putative)
TPPP	-1.41	1.20	ENSG00000171368	tubulin polymerization promoting protein
TM4SF5	-1.42	2.99	ENSG00000142484	transmembrane 4 L six family member 5
ANKRD1	-1.42	7.10	ENSG00000148677	ankyrin repeat domain 1
STC2	-1.43	4.47	ENSG00000113739	stanniocalcin 2

GOT1	-1.43	7.15	ENSG00000120053	glutamic-oxaloacetic transaminase 1
CHCHD2P2	-1.43	1.46	ENSG00000275346	coiled-coil-helix-coiled-coil-helix domain containing 2 pseudogene 2
HPD	-1.43	3.57	ENSG00000158104	4-hydroxyphenylpyruvate dioxygenase
LCOR	-1.44	3.73	ENSG00000196233	ligand dependent nuclear receptor corepressor
FLNC	-1.44	5.55	ENSG00000128591	filamin C
VGLL3	-1.44	4.95	ENSG00000206538	vestigial like family member 3
TMEM200A	-1.44	1.28	ENSG00000164484	transmembrane protein 200A
OSGIN1	-1.45	3.40	ENSG00000140961	oxidative stress induced growth inhibitor 1
FLI1	-1.45	2.18	ENSG00000151702	Fli-1 proto-oncogene, ETS transcription factor
HES6	-1.46	4.47	ENSG00000144485	hes family bHLH transcription factor 6
ATP2B2	-1.46	5.11	ENSG00000157087	ATPase plasma membrane Ca2+ transporting 2
NR1H4	-1.46	3.06	ENSG00000012504	nuclear receptor subfamily 1 group H member 4
MCTP1	-1.47	3.16	ENSG00000175471	multiple C2 and transmembrane domain containing 1
RP11-103J8.1	-1.48	1.84	ENSG00000270792	N/A
CNN2	-1.49	8.33	ENSG00000064666	calponin 2
DDN	-1.50	1.45	ENSG00000181418	dendrin
ASNS	-1.50	5.70	ENSG00000070669	asparagine synthetase (glutamine-hydrolyzing)
GLTPD2	-1.50	4.80	ENSG00000182327	glycolipid transfer protein domain containing 2
FEM1A	-1.51	1.12	ENSG00000141965	fem-1 homolog A
NPAS1	-1.52	3.10	ENSG00000130751	neuronal PAS domain protein 1
BHLHA15	-1.52	2.94	ENSG00000180535	basic helix-loop-helix family member a15
IQCJ-SCHIP1	-1.52	1.63	ENSG00000283154	IQCJ-SCHIP1 readthrough
TRABD2A	-1.53	5.35	ENSG00000186854	TraB domain containing 2A
RP11-132A1.4	-1.53	2.31	ENSG00000232445	N/A
CNIH3	-1.54	1.89	ENSG00000143786	cornichon family AMPA receptor auxiliary protein 3
PHGDH	-1.54	5.41	ENSG00000092621	phosphoglycerate dehydrogenase

DTX4	-1.55	4.55	ENSG00000110042	deltex E3 ubiquitin ligase 4
RP11-404G16.2	-1.55	3.97	ENSG00000244062	N/A
SH2D5	-1.56	2.83	ENSG00000189410	SH2 domain containing 5
MVB12B	-1.56	5.52	ENSG00000196814	multivesicular body subunit 12B
MATN3	-1.56	3.88	ENSG00000132031	matrilin 3
KLHL11	-1.59	1.10	ENSG00000178502	kelch like family member 11
CYP1A1	-1.60	5.24	ENSG00000140465	cytochrome P450 family 1 subfamily A member 1
PDF	-1.62	2.93	ENSG00000258429	peptide deformylase (mitochondrial)
MMP17	-1.62	1.76	ENSG00000198598	matrix metallopeptidase 17
SLC16A7	-1.63	4.38	ENSG00000118596	solute carrier family 16 member 7
COL5A2	-1.64	7.44	ENSG00000204262	collagen type V alpha 2 chain
C2orf16	-1.64	2.36	ENSG00000221843	chromosome 2 open reading frame 16
KAZALD1	-1.65	1.72	ENSG00000107821	Kazal type serine peptidase inhibitor domain 1
F2	-1.66	5.68	ENSG00000180210	coagulation factor II, thrombin
CUX2	-1.66	5.17	ENSG00000111249	cut like homeobox 2
FGA	-1.67	5.90	ENSG00000171560	fibrinogen alpha chain
TRIB3	-1.67	5.96	ENSG00000101255	tribbles pseudokinase 3
VSNL1	-1.67	4.69	ENSG00000163032	visinin like 1
FGG	-1.69	3.38	ENSG00000171557	fibrinogen gamma chain
ELF5	-1.71	2.66	ENSG00000135374	E74 like ETS transcription factor 5
FGD3	-1.72	1.41	ENSG00000127084	FYVE, RhoGEF and PH domain containing 3
PPM1K	-1.79	3.95	ENSG00000163644	protein phosphatase, Mg2+/Mn2+ dependent 1K
LONRF3	-1.81	2.76	ENSG00000175556	LON peptidase N-terminal domain and ring finger 3
EDNRB	-1.85	3.29	ENSG00000136160	endothelin receptor type B
RTN4RL2	-1.88	5.27	ENSG00000186907	reticulon 4 receptor-like 2
SLC7A11	-1.90	4.22	ENSG00000151012	solute carrier family 7 member 11
PCSK9	-1.91	5.94	ENSG00000169174	proprotein convertase subtilisin/kexin type 9
NTS	-1.94	8.16	ENSG00000133636	neurotensin
CEBPA	-1.96	7.42	ENSG00000245848	CCAAT/enhancer binding protein alpha
SERBP1	-1.96	7.71	ENSG00000142864	SERPINE1 mRNA binding protein 1
BCAS1	-1.98	1.98	ENSG00000064787	breast carcinoma amplified sequence 1

CYP24A1	-2.00	1.14	ENSG00000019186	cytochrome P450 family 24 subfamily A member 1
PPARGC1B	-2.01	1.49	ENSG00000155846	PPARG coactivator 1 beta
SLC1A3	-2.02	4.55	ENSG00000079215	solute carrier family 1 member 3
CYP51A1	-2.07	3.39	ENSG00000001630	cytochrome P450 family 51 subfamily A member 1
SLC6A12	-2.12	3.09	ENSG00000111181	solute carrier family 6 member 12
TERT	-2.13	1.80	ENSG00000164362	telomerase reverse transcriptase
C10orf54	-2.31	3.72	ENSG00000107738	chromosome 10 open reading frame 54
AHSG	-2.35	9.32	ENSG00000145192	alpha 2-HS glycoprotein
APOA1	-2.39	7.02	ENSG00000118137	apolipoprotein A1
SYN3	-2.41	3.64	ENSG00000185666	synapsin III
HFE2	-2.44	1.67	ENSG00000168509	hemochromatosis type 2 (juvenile)
DUSP9	-2.45	7.25	ENSG00000130829	dual specificity phosphatase 9
PDE4B	-2.48	1.41	ENSG00000184588	phosphodiesterase 4B
CDH12	-2.48	4.99	ENSG00000154162	cadherin 12
TENM4	-2.88	1.64	ENSG00000149256	teneurin transmembrane protein 4
FGB	-2.99	4.02	ENSG00000171564	fibrinogen beta chain
MYBPC3	-3.24	1.69	ENSG00000134571	myosin binding protein C, cardiac
ANGPTL3	-3.89	4.78	ENSG00000132855	angiopoietin like 3
NPPB	-4.29	3.22	ENSG00000120937	natriuretic peptide B
AFP	-4.58	7.58	ENSG00000081051	alpha fetoprotein

Supplementary Table 4

Gene symbol	log2 fold change (shS6K1/shCtrl)	log2 counts per million	Gene ID	Gene description
APOA4	4.36	1.86	ENSG00000110244	apolipoprotein A4
CAPN8	4.20	3.42	ENSG00000203697	calpain 8
SCGN	4.19	3.72	ENSG00000079689	secretagogin, EF-hand calcium binding protein
SERPINA4	4.00	2.62	ENSG00000100665	serpin family A member 4
IFITM10	3.88	3.28	ENSG00000281618	interferon induced transmembrane protein 10
FOLR1	3.85	2.35	ENSG00000110195	folate receptor 1
RP11-379K22.3	3.61	2.13	ENSG00000270919	N/A
TFF1	3.51	2.94	ENSG00000160182	trefoil factor 1
IFI27	3.43	1.27	ENSG00000275214	interferon alpha inducible protein 27
HLA-DPB1	3.36	2.37	ENSG00000229295	major histocompatibility complex, class II, DP beta 1
GAL3ST1	3.32	1.87	ENSG00000128242	galactose-3-O-sulfotransferase 1
PHLDA3	3.31	1.16	ENSG00000174307	pleckstrin homology like domain family A member 3
FXYD3	3.26	2.86	ENSG00000089356	FXYD domain containing ion transport regulator 3
TSPO2	3.25	1.50	ENSG00000112212	translocator protein 2
C1orf228	3.25	2.17	ENSG00000198520	chromosome 1 open reading frame 228
KRT81	3.22	2.15	ENSG00000205426	keratin 81
IFI6	3.22	6.37	ENSG00000126709	interferon alpha inducible protein 6
QPCT	3.17	1.04	ENSG00000115828	glutaminyl-peptide cyclotransferase
TMEM59L	3.04	1.88	ENSG00000105696	transmembrane protein 59 like
FABP6	2.97	2.39	ENSG00000170231	fatty acid binding protein 6
ARHGDIB	2.92	2.34	ENSG00000111348	Rho GDP dissociation inhibitor beta
OLFML2A	2.88	2.66	ENSG00000185585	olfactomedin like 2A
S100A3	2.86	1.84	ENSG00000188015	S100 calcium binding protein A3
S100A4	2.85	2.43	ENSG00000196154	S100 calcium binding protein A4
RP11-10A14.5	2.83	1.81	ENSG00000248538	N/A
TTR	2.82	3.49	ENSG00000118271	transthyretin
EMP3	2.81	4.83	ENSG00000142227	epithelial membrane protein 3
CLDN2	2.81	2.17	ENSG00000165376	claudin 2

GPR4	2.72	1.92	ENSG00000177464	G protein-coupled receptor 4
SLC22A17	2.72	3.37	ENSG00000283151	solute carrier family 22 member 17
EEF1A2	2.69	4.74	ENSG00000101210	eukaryotic translation elongation factor 1 alpha 2
CA9	2.68	3.53	ENSG00000107159	carbonic anhydrase 9
LINC00173	2.67	1.78	ENSG00000196668	long intergenic non-protein coding RNA 173
HLA-DPA1	2.67	2.47	ENSG00000231389	major histocompatibility complex, class II, DP alpha 1
THSD4	2.65	1.16	ENSG00000187720	thrombospondin type 1 domain containing 4
CA12	2.64	1.65	ENSG00000074410	carbonic anhydrase 12
LGI4	2.62	2.81	ENSG00000153902	leucine rich repeat LGI family member 4
CD7	2.61	1.27	ENSG00000173762	CD7 molecule
HTRA1	2.60	3.20	ENSG00000166033	HtrA serine peptidase 1
SERINC2	2.56	7.00	ENSG00000168528	serine incorporator 2
TNFRSF1B	2.53	1.85	ENSG00000028137	TNF receptor superfamily member 1B
VSIG1	2.53	1.20	ENSG00000101842	V-set and immunoglobulin domain containing 1
SPINT1	2.50	1.53	ENSG00000166145	serine peptidase inhibitor, Kunitz type 1
GPSM3	2.49	2.33	ENSG00000234243	G-protein signaling modulator 3
C15orf48	2.48	3.03	ENSG00000166920	chromosome 15 open reading frame 48
UGT2B11	2.48	1.71	ENSG00000213759	UDP glucuronosyltransferase family 2 member B11
SEPT4	2.47	2.18	ENSG00000108387	septin 4
TIMP1	2.46	5.58	ENSG00000102265	TIMP metallopeptidase inhibitor 1
SLC22A9	2.45	1.39	ENSG00000149742	solute carrier family 22 member 9
MOXD1	2.43	4.44	ENSG00000079931	monooxygenase DBH like 1
AP001626.2	2.41	1.11	ENSG00000235023	N/A
IL2RG	2.41	3.21	ENSG00000147168	interleukin 2 receptor subunit gamma
CD74	2.40	1.74	ENSG00000019582	CD74 molecule
SNCG	2.40	2.76	ENSG00000173267	synuclein gamma
DPP4	2.40	2.36	ENSG00000197635	dipeptidyl peptidase 4

RP11-130L8.2	2.39	1.82	ENSG00000269976	N/A
RP11-16E12.2	2.39	2.04	ENSG00000259772	N/A
CRIP2	2.37	2.16	ENSG00000182809	cysteine rich protein 2
SPTSSB	2.37	4.62	ENSG00000196542	serine palmitoyltransferase small subunit B
LDHD	2.37	2.64	ENSG00000166816	lactate dehydrogenase D
SYCE1L	2.36	1.20	ENSG00000205078	synaptonemal complex central element protein 1 like
EBF4	2.35	1.70	ENSG00000088881	early B-cell factor 4
CFB	2.34	4.51	ENSG00000243649	complement factor B
HPGD	2.34	2.37	ENSG00000164120	hydroxyprostaglandin dehydrogenase 15-(NAD)
SULT1C2	2.34	1.18	ENSG00000198203	sulfotransferase family 1C member 2
CCDC153	2.33	3.01	ENSG00000248712	coiled-coil domain containing 153
CIDEC	2.33	4.24	ENSG00000187288	cell death inducing DFFA like effector c
FAM131C	2.32	2.18	ENSG00000185519	family with sequence similarity 131 member C
PIGZ	2.32	3.88	ENSG00000119227	phosphatidylinositol glycan anchor biosynthesis class Z
COL6A2	2.31	5.49	ENSG00000142173	collagen type VI alpha 2 chain
DKKL1	2.30	1.90	ENSG00000104901	dickkopf like acrosomal protein 1
NPY6R	2.30	1.74	ENSG00000226306	neuropeptide Y receptor Y6 (pseudogene)
CYP4F22	2.29	1.82	ENSG00000171954	cytochrome P450 family 4 subfamily F member 22
PDZD3	2.29	1.01	ENSG00000172367	PDZ domain containing 3
CDH17	2.28	2.43	ENSG00000079112	cadherin 17
ACVRL1	2.28	1.13	ENSG00000139567	activin A receptor like type 1
IFI27L2	2.27	3.93	ENSG00000276879	interferon alpha inducible protein 27 like 2
RP11-206M11.7	2.26	1.63	ENSG00000244468	N/A
FXYD5	2.26	5.47	ENSG00000089327	FXYD domain containing ion transport regulator 5
NRAP	2.22	2.48	ENSG00000197893	nebulin related anchoring protein
CDK18	2.19	6.04	ENSG00000117266	cyclin dependent kinase 18
OVGP1	2.19	2.02	ENSG00000085465	oviductal glycoprotein 1

AC079466.1	2.18	1.17	ENSG00000266976	N/A
LINC00511	2.18	2.70	ENSG00000227036	long intergenic non-protein coding RNA 511
CEACAM1	2.16	3.70	ENSG00000079385	carcinoembryonic antigen related cell adhesion molecule 1
NFATC4	2.16	1.86	ENSG00000100968	nuclear factor of activated T-cells 4
KRT7	2.16	1.79	ENSG00000135480	keratin 7
IFIT1	2.15	1.36	ENSG00000185745	interferon induced protein with tetratricopeptide repeats 1
SLC5A12	2.15	1.26	ENSG00000148942	solute carrier family 5 member 12
TMEM92	2.14	3.25	ENSG00000167105	transmembrane protein 92
ENO2	2.14	1.98	ENSG00000111674	enolase 2
TMEM229B	2.13	1.53	ENSG00000198133	transmembrane protein 229B
SLC5A11	2.13	2.09	ENSG00000158865	solute carrier family 5 member 11
RP11-441O15.3	2.12	1.01	ENSG00000224934	N/A
CORO6	2.11	2.86	ENSG00000167549	coronin 6
PRTN3	2.11	1.95	ENSG00000196415	proteinase 3
OASL	2.11	1.89	ENSG00000135114	2'-5'-oligoadenylate synthetase like
PAQR8	2.10	6.00	ENSG00000170915	progestin and adipoQ receptor family member 8
CLDN4	2.10	1.22	ENSG00000189143	claudin 4
SOCS2-AS1	2.09	2.17	ENSG00000246985	SOCS2 antisense RNA 1
NDRG1	2.08	6.89	ENSG00000104419	N-myc downstream regulated 1
GALNT6	2.07	1.30	ENSG00000139629	polypeptide N-acetylgalactosaminyltransferase 6
LINC00694	2.07	3.11	ENSG00000225873	long intergenic non-protein coding RNA 694
ISG15	2.07	6.47	ENSG00000187608	ISG15 ubiquitin-like modifier
PRAP1	2.06	1.79	ENSG00000165828	proline rich acidic protein 1
RAB3B	2.06	5.31	ENSG00000169213	RAB3B, member RAS oncogene family
LMF1	2.06	1.08	ENSG00000103227	lipase maturation factor 1
MYEOV	2.06	3.57	ENSG00000172927	myeloma overexpressed
BTN1A1	2.06	1.91	ENSG00000124557	butyrophilin subfamily 1 member A1
TM4SF1-AS1	2.06	1.91	ENSG00000240541	TM4SF1 antisense RNA 1
GLIPR2	2.04	1.96	ENSG00000122694	GLI pathogenesis related 2

HES7	2.04	3.31	ENSG00000179111	hes family bHLH transcription factor 7
NOVA2	2.03	1.83	ENSG00000104967	NOVA alternative splicing regulator 2
TMEM91	2.03	1.83	ENSG00000142046	transmembrane protein 91
RUNDC3A	2.03	1.36	ENSG00000108309	RUN domain containing 3A
ECM1	2.03	2.12	ENSG00000143369	extracellular matrix protein 1
RP11-798K3.2	2.03	3.05	ENSG00000259347	N/A
CDKN1A	2.02	4.51	ENSG00000124762	cyclin dependent kinase inhibitor 1A
HRCT1	2.02	1.26	ENSG00000196196	histidine rich carboxyl terminus 1
DUSP13	2.01	2.88	ENSG00000079393	dual specificity phosphatase 13
F2RL1	2.01	2.78	ENSG00000164251	F2R like trypsin receptor 1
AXL	2.01	4.59	ENSG00000167601	AXL receptor tyrosine kinase
CD44	1.99	1.80	ENSG00000026508	CD44 molecule (Indian blood group)
GLIPR1	1.99	1.03	ENSG00000139278	GLI pathogenesis related 1
PAGE1	1.99	1.92	ENSG00000068985	PAGE family member 1
NPC1L1	1.99	1.24	ENSG00000015520	NPC1 like intracellular cholesterol transporter 1
ACSL5	1.99	5.16	ENSG00000197142	acyl-CoA synthetase long-chain family member 5
KCNN1	1.99	4.45	ENSG00000105642	potassium calcium-activated channel subfamily N member 1
AP001626.1	1.97	2.42	ENSG00000225431	N/A
RP4-758J18.10	1.97	1.71	ENSG00000225285	N/A
GNG12	1.96	2.72	ENSG00000172380	G protein subunit gamma 12
C10orf10	1.96	1.22	ENSG00000165507	chromosome 10 open reading frame 10
ZNF208	1.96	1.01	ENSG00000160321	zinc finger protein 208
SERPINE1	1.96	2.96	ENSG00000106366	serpin family E member 1
CSTA	1.95	1.63	ENSG00000121552	cystatin A
ARSD	1.95	1.83	ENSG00000006756	arylsulfatase D
RP11-44N11.3	1.93	1.46	ENSG00000272043	N/A
SERPINE2	1.93	3.30	ENSG00000135919	serpin family E member 2
TMEM86A	1.93	2.24	ENSG00000151117	transmembrane protein 86A
CACNG6	1.92	3.50	ENSG00000130433	calcium voltage-gated channel auxiliary subunit gamma 6
TMEM74B	1.92	2.71	ENSG00000125895	transmembrane protein 74B

BEST3	1.90	1.17	ENSG00000127325	bestrophin 3
VWCE	1.90	1.44	ENSG00000167992	von Willebrand factor C and EGF domains
SERPINA5	1.90	4.30	ENSG00000188488	serpin family A member 5
VNN2	1.89	2.59	ENSG00000112303	vanin 2
LINC00886	1.88	1.05	ENSG00000240875	long intergenic non-protein coding RNA 886
SLC4A3	1.87	1.49	ENSG00000114923	solute carrier family 4 member 3
LAMB3	1.87	3.97	ENSG00000196878	laminin subunit beta 3
BMF	1.87	4.10	ENSG00000104081	Bcl2 modifying factor
RAB27B	1.85	2.37	ENSG00000041353	RAB27B, member RAS oncogene family
LHPP	1.85	5.66	ENSG00000107902	phospholysine phosphohistidine inorganic pyrophosphate phosphatase
SLC2A9	1.85	4.13	ENSG00000109667	solute carrier family 2 member 9
P2RY6	1.84	1.54	ENSG00000171631	pyrimidinergic receptor P2Y6
IFITM2	1.83	4.00	ENSG00000185201	interferon induced transmembrane protein 2
CAMKV	1.83	1.21	ENSG00000164076	CaM kinase like vesicle associated
CCNG2	1.83	1.68	ENSG00000138764	cyclin G2
NOTCH2NL	1.83	1.30	ENSG00000264343	notch 2 N-terminal like
RP11-356M20.1	1.83	1.30	ENSG00000259674	N/A
TIMP2	1.82	4.44	ENSG00000035862	TIMP metallopeptidase inhibitor 2
ABCG4	1.82	2.80	ENSG00000172350	ATP binding cassette subfamily G member 4
ATOH8	1.82	1.96	ENSG00000168874	atonal bHLH transcription factor 8
VWA5B2	1.82	3.93	ENSG00000145198	von Willebrand factor A domain containing 5B2
RAB17	1.82	4.05	ENSG00000124839	RAB17, member RAS oncogene family
SEMA4A	1.80	2.24	ENSG00000196189	semaphorin 4A
RP11-54O7.3	1.79	2.36	ENSG00000223764	N/A
CLU	1.78	11.65	ENSG00000120885	clusterin
PLEKHH2	1.78	2.82	ENSG00000152527	pleckstrin homology, MyTH4 and FERM domain containing H2
DUSP15	1.77	2.35	ENSG00000149599	dual specificity phosphatase 15
JAK3	1.77	3.15	ENSG00000105639	Janus kinase 3

SCN1B	1.77	4.78	ENSG00000105711	sodium voltage-gated channel beta subunit 1
CDH23	1.77	3.28	ENSG00000107736	cadherin related 23
KPNA7	1.76	3.05	ENSG00000185467	karyopherin subunit alpha 7
PCDH1	1.76	2.72	ENSG00000156453	protocadherin 1
SLC12A5	1.76	2.92	ENSG00000124140	solute carrier family 12 member 5
GSN	1.75	5.73	ENSG00000148180	gelsolin
RP11-617F23.1	1.75	2.62	ENSG00000259291	N/A
TMEM159	1.74	2.01	ENSG00000011638	transmembrane protein 159
LDLRAD4	1.74	1.96	ENSG00000168675	low density lipoprotein receptor class A domain containing 4
TCN2	1.74	1.91	ENSG00000185339	transcobalamin 2
CACNG7	1.74	1.15	ENSG00000105605	calcium voltage-gated channel auxiliary subunit gamma 7
APOC1P1	1.74	1.05	ENSG00000214855	apolipoprotein C1 pseudogene 1
RP11-44N11.2	1.74	3.40	ENSG00000272384	N/A
CRLF2	1.74	2.57	ENSG00000205755	cytokine receptor-like factor 2
TP53INP1	1.73	3.78	ENSG00000164938	tumor protein p53 inducible nuclear protein 1
SPEG	1.73	1.84	ENSG00000072195	SPEG complex locus
AGAP2	1.72	1.66	ENSG00000135439	ArfGAP with GTPase domain, ankyrin repeat and PH domain 2
DSC2	1.71	2.62	ENSG00000134755	desmocollin 2
RP11-466H18.1	1.71	1.21	ENSG00000244398	N/A
TUBA4B	1.71	2.45	ENSG00000243910	tubulin alpha 4b
EVA1C	1.71	1.03	ENSG00000166979	eva-1 homolog C
ADAMTS7	1.70	1.64	ENSG00000136378	ADAM metallopeptidase with thrombospondin type 1 motif 7
ANXA3	1.70	3.91	ENSG00000138772	annexin A3
HPSE	1.69	2.32	ENSG00000173083	heparanase
MFGE8	1.69	6.47	ENSG00000140545	milk fat globule-EGF factor 8 protein
RP11-480I12.5	1.69	1.81	ENSG00000214796	N/A
PAGE2B	1.68	1.28	ENSG00000238269	PAGE family member 2B
VGF	1.68	3.87	ENSG00000128564	VGF nerve growth factor inducible
LGALS1	1.68	7.74	ENSG00000100097	galectin 1
DAPK2	1.68	3.50	ENSG00000035664	death associated protein kinase 2
RP11-284F21.9	1.68	3.89	ENSG00000272068	N/A

CRYBB2	1.67	1.00	ENSG00000244752	crystallin beta B2
CRYAB	1.67	3.37	ENSG00000109846	crystallin alpha B
ADSSL1	1.67	2.14	ENSG00000185100	adenylosuccinate synthase like 1
GLT8D2	1.67	2.14	ENSG00000120820	glycosyltransferase 8 domain containing 2
MBNL2	1.66	5.10	ENSG00000139793	muscleblind like splicing regulator 2
PPP1R3C	1.66	3.00	ENSG00000119938	protein phosphatase 1 regulatory subunit 3C
EMB	1.64	1.66	ENSG00000170571	embigin
F2R	1.64	1.66	ENSG00000181104	coagulation factor II thrombin receptor
RASSF2	1.63	2.07	ENSG00000101265	Ras association domain family member 2
DAO	1.63	2.90	ENSG00000110887	D-amino acid oxidase
FER1L4	1.63	5.76	ENSG00000088340	fer-1 like family member 4, pseudogene
TRAF3IP2	1.62	1.23	ENSG00000056972	TRAF3 interacting protein 2
DOC2GP	1.62	2.31	ENSG00000231793	double C2 domain gamma pseudogene
RP4-710M16.2	1.62	3.02	ENSG00000223956	N/A
HMGCS2	1.62	2.87	ENSG00000134240	3-hydroxy-3-methylglutaryl-CoA synthase 2
TTLL6	1.61	1.81	ENSG00000170703	tubulin tyrosine ligase like 6
POU2AF1	1.60	1.30	ENSG00000110777	POU class 2 associating factor 1
RP11-6B4.1	1.60	1.91	ENSG00000243694	N/A
FKBP1B	1.60	3.41	ENSG00000119782	FK506 binding protein 1B
GPRC5A	1.60	2.54	ENSG00000013588	G protein-coupled receptor class C group 5 member A
RPL10L	1.59	1.43	ENSG00000165496	ribosomal protein L10 like
GNG7	1.59	3.04	ENSG00000176533	G protein subunit gamma 7
ZDHHC19	1.58	1.12	ENSG00000163958	zinc finger DHHC-type containing 19
AGR2	1.58	7.93	ENSG00000106541	anterior gradient 2, protein disulphide isomerase family member
SOX9	1.58	2.65	ENSG00000125398	SRY-box 9
RP11-495P10.6	1.58	2.24	ENSG00000234283	N/A
TMEM37	1.57	6.57	ENSG00000171227	transmembrane protein 37

SMIM6	1.56	1.41	ENSG00000259120	small integral membrane protein 6
USH1C	1.56	4.72	ENSG00000006611	USH1 protein network component harmonin
APOBEC3B	1.56	2.34	ENSG00000179750	apolipoprotein B mRNA editing enzyme catalytic subunit 3B
NRG2	1.56	1.19	ENSG00000158458	neuregulin 2
C11orf70	1.56	1.72	ENSG00000137691	chromosome 11 open reading frame 70
TMEM54	1.56	5.47	ENSG00000121900	transmembrane protein 54
COL1A1	1.56	2.57	ENSG00000108821	collagen type I alpha 1 chain
EHD2	1.56	4.40	ENSG00000024422	EH domain containing 2
TNNT1	1.55	6.63	ENSG00000105048	troponin T1, slow skeletal type
MSRB2	1.55	6.07	ENSG00000148450	methionine sulfoxide reductase B2
SEL1L3	1.55	5.64	ENSG00000091490	SEL1L family member 3
TTC9	1.55	2.14	ENSG00000133985	tetratricopeptide repeat domain 9
AF127936.7	1.55	1.10	ENSG00000281903	N/A
PRKCG	1.54	4.99	ENSG00000126583	protein kinase C gamma
GREM2	1.54	1.86	ENSG00000180875	gremlin 2, DAN family BMP antagonist
TP53I11	1.54	5.75	ENSG00000175274	tumor protein p53 inducible protein 11
PROCR	1.54	2.86	ENSG00000101000	protein C receptor
SSUH2	1.54	5.42	ENSG00000125046	ssu-2 homolog (C. elegans)
DUSP5	1.54	5.17	ENSG00000138166	dual specificity phosphatase 5
VIM	1.54	7.07	ENSG00000026025	vimentin
CAV1	1.54	2.96	ENSG00000105974	caveolin 1
HPN	1.53	7.24	ENSG00000105707	hepsin
MEIS3	1.53	4.66	ENSG00000105419	Meis homeobox 3
C2CD4B	1.53	1.52	ENSG00000205502	C2 calcium dependent domain containing 4B
NRGN	1.53	3.73	ENSG00000154146	neurogranin
CYP3A5	1.53	1.90	ENSG00000106258	cytochrome P450 family 3 subfamily A member 5
TCF7L1	1.53	3.13	ENSG00000152284	transcription factor 7 like 1
CCDC159	1.53	4.80	ENSG00000183401	coiled-coil domain containing 159
DNAH5	1.53	1.17	ENSG00000039139	dynein axonemal heavy chain 5
IQSEC2	1.53	3.20	ENSG00000124313	IQ motif and Sec7 domain 2

SLC17A7	1.53	3.10	ENSG00000104888	solute carrier family 17 member 7
ATP2A3	1.52	3.86	ENSG00000074370	ATPase sarcoplasmic/endoplasmic reticulum Ca2+ transporting 3
IQCD	1.52	3.18	ENSG00000166578	IQ motif containing D
VNN3	1.52	1.45	ENSG00000093134	vanin 3
CTD-2545H1.2	1.51	1.23	ENSG00000262445	N/A
KLHL7-AS1	1.51	1.23	ENSG00000230658	KLHL7 antisense RNA 1 (head to head)
BTBD16	1.51	1.98	ENSG00000138152	BTB domain containing 16
PLEKHA2	1.51	2.23	ENSG00000169499	pleckstrin homology domain containing A2
RAB26	1.51	5.79	ENSG00000167964	RAB26, member RAS oncogene family
PPP1R3F	1.50	1.73	ENSG00000049769	protein phosphatase 1 regulatory subunit 3F
SRRM3	1.50	4.03	ENSG00000177679	serine/arginine repetitive matrix 3
MMP11	1.50	2.56	ENSG00000099953	matrix metallopeptidase 11
MACROD1	1.50	4.72	ENSG00000133315	MACRO domain containing 1
TEPP	1.49	1.56	ENSG00000159648	testis, prostate and placenta expressed
TBX19	1.48	2.69	ENSG00000143178	T-box 19
HLA-B	1.48	6.30	ENSG00000206450	major histocompatibility complex, class I, B
PCAT6	1.48	4.72	ENSG00000228288	prostate cancer associated transcript 6 (non-protein coding)
B3GNT5	1.48	1.35	ENSG00000176597	UDP-GlcNAc:betaGal beta-1,3-N-acetylglucosaminyltransferase 5
PCED1B	1.48	4.43	ENSG00000179715	PC-esterase domain containing 1B
GSDMB	1.47	6.05	ENSG00000073605	gasdermin B
CCDC88B	1.47	4.54	ENSG00000168071	coiled-coil domain containing 88B
C2orf81	1.47	3.22	ENSG00000159239	chromosome 2 open reading frame 81
DENND6B	1.47	2.08	ENSG00000205593	DENN domain containing 6B
UCP2	1.46	7.04	ENSG00000175567	uncoupling protein 2
DOCK11	1.46	1.70	ENSG00000147251	dedicator of cytokinesis 11
XPNPEP2	1.46	2.64	ENSG00000122121	X-prolyl aminopeptidase 2
SP100	1.46	2.87	ENSG00000067066	SP100 nuclear antigen

HIST1H2BD	1.45	2.59	ENSG00000158373	histone cluster 1 H2B family member d
AC004012.1	1.45	2.07	ENSG00000233942	N/A
PBXIP1	1.45	6.70	ENSG00000163346	PBX homeobox interacting protein 1
GBP2	1.45	5.53	ENSG00000162645	guanylate binding protein 2
KIAA1107	1.45	1.19	ENSG00000069712	KIAA1107
TPD52L1	1.45	2.74	ENSG00000111907	tumor protein D52-like 1
ANGPTL4	1.44	5.94	ENSG00000167772	angiopoietin like 4
RP11-1398P2.1	1.44	1.52	ENSG00000244459	N/A
SPATA6L	1.44	1.52	ENSG00000106686	spermatogenesis associated 6 like
PDCD4	1.44	4.68	ENSG00000150593	programmed cell death 4 (neoplastic transformation inhibitor)
HLA-DMA	1.44	5.06	ENSG00000243215	major histocompatibility complex, class II, DM alpha
LRRC8C	1.44	1.39	ENSG00000171488	leucine rich repeat containing 8 family member C
AZU1	1.43	1.25	ENSG00000172232	azurocidin 1
RP11-848P1.7	1.43	1.25	ENSG00000264148	N/A
COL6A1	1.43	5.72	ENSG00000142156	collagen type VI alpha 1 chain
UGT1A10	1.43	3.32	ENSG00000242515	UDP glucuronosyltransferase family 1 member A10
LY6E	1.43	7.22	ENSG00000160932	lymphocyte antigen 6 complex, locus E
TXNIP	1.43	1.73	ENSG00000265972	thioredoxin interacting protein
CITED4	1.43	3.38	ENSG00000179862	Cbp/p300 interacting transactivator with Glu/Asp rich carboxy-terminal domain 4
GFPT2	1.43	2.85	ENSG00000131459	glutamine-fructose-6-phosphate transaminase 2
TSPAN10	1.42	1.31	ENSG00000182612	tetraspanin 10
MAMSTR	1.42	3.53	ENSG00000176909	MEF2 activating motif and SAP domain containing transcriptional regulator
RP11-467L13.5	1.42	5.41	ENSG00000223722	N/A
S100A14	1.42	4.93	ENSG00000189334	S100 calcium binding protein A14
NEURL3	1.42	2.13	ENSG00000163121	neuralized E3 ubiquitin protein ligase 3
CRYM	1.42	3.22	ENSG00000103316	crystallin mu

RP11-390P2.4	1.42	3.48	ENSG00000225177	N/A
AC144652.1	1.41	1.17	ENSG00000273117	N/A
EDA	1.41	1.17	ENSG00000158813	ectodysplasin A
RAC1P2	1.41	1.17	ENSG00000249936	ras-related C3 botulinum toxin substrate 1 pseudogene 2
SOCS2	1.41	3.37	ENSG00000120833	suppressor of cytokine signaling 2
SLC7A10	1.41	2.67	ENSG00000130876	solute carrier family 7 member 10
SPOCK2	1.41	2.53	ENSG00000107742	SPARC/osteonectin, cwcv and kazal like domains proteoglycan 2
RP11-462G2.1	1.41	4.85	ENSG00000237643	N/A
AC005355.2	1.41	2.08	ENSG00000251169	N/A
TRPV2	1.41	1.72	ENSG00000187688	transient receptor potential cation channel subfamily V member 2
LINC00634	1.40	1.23	ENSG00000205704	long intergenic non-protein coding RNA 634
SLC1A6	1.40	4.78	ENSG00000105143	solute carrier family 1 member 6
CABP1	1.40	1.60	ENSG00000157782	calcium binding protein 1
CGA	1.40	8.19	ENSG00000135346	glycoprotein hormones, alpha polypeptide
TMSB10P1	1.40	2.76	ENSG00000228499	thymosin beta 10 pseudogene 1
SLC29A4	1.39	8.54	ENSG00000164638	solute carrier family 29 member 4
APLF	1.39	1.07	ENSG00000169621	aprataxin and PNKP like factor
BSPRY	1.39	1.07	ENSG00000119411	B-box and SPRY domain containing
SHBG	1.39	1.07	ENSG00000129214	sex hormone binding globulin
RP6-65G23.3	1.39	2.94	ENSG00000259153	N/A
NINJ2	1.39	3.29	ENSG00000171840	ninjurin 2
HLA-F	1.39	3.76	ENSG00000229698	major histocompatibility complex, class I, F
APH1B	1.38	3.04	ENSG00000138613	aph-1 homolog B, gamma-secretase subunit
RHEBL1	1.38	1.14	ENSG00000167550	Ras homolog enriched in brain like 1
MCOLN2	1.38	1.75	ENSG00000153898	mucolipin 2
RBP5	1.38	1.75	ENSG00000139194	retinol binding protein 5
WDR31	1.38	1.75	ENSG00000148225	WD repeat domain 31
IL1R2	1.37	5.24	ENSG00000115590	interleukin 1 receptor type 2
C1QTNF1	1.37	6.06	ENSG00000173918	C1q and tumor necrosis factor related protein 1

CAV2	1.37	3.11	ENSG00000105971	caveolin 2
CREB3L1	1.37	3.99	ENSG00000157613	cAMP responsive element binding protein 3 like 1
USP2-AS1	1.37	2.31	ENSG00000245248	USP2 antisense RNA 1 (head to head)
AQP3	1.37	3.15	ENSG00000165272	aquaporin 3 (Gill blood group)
C11orf86	1.36	1.47	ENSG00000173237	chromosome 11 open reading frame 86
RNASET2	1.36	6.58	ENSG00000026297	ribonuclease T2
CAPN5	1.36	5.57	ENSG00000149260	calpain 5
CDHR2	1.36	4.21	ENSG00000074276	cadherin related family member 2
DKK3	1.36	5.79	ENSG00000050165	dickkopf WNT signaling pathway inhibitor 3
PKD1L2	1.36	2.00	ENSG00000166473	polycystin 1 like 2 (gene/pseudogene)
HSD17B7P2	1.36	1.52	ENSG00000099251	hydroxysteroid 17-beta dehydrogenase 7 pseudogene 2
REEP2	1.35	2.63	ENSG00000132563	receptor accessory protein 2
ATP1B2	1.35	1.33	ENSG00000129244	ATPase Na+/K+ transporting subunit beta 2
CACNB3	1.35	4.66	ENSG00000167535	calcium voltage-gated channel auxiliary subunit beta 3
RWDD2B	1.35	4.57	ENSG00000156253	RWD domain containing 2B
SRPX	1.35	2.67	ENSG00000101955	sushi repeat containing protein, X-linked
IFITM3	1.35	9.76	ENSG00000142089	interferon induced transmembrane protein 3
UGT2B7	1.35	1.12	ENSG00000171234	UDP glucuronosyltransferase family 2 member B7
ITGA3	1.34	4.82	ENSG00000005884	integrin subunit alpha 3
WDR25	1.34	3.39	ENSG00000176473	WD repeat domain 25
UBE2L6	1.34	4.87	ENSG00000156587	ubiquitin conjugating enzyme E2 L6
KCNH3	1.34	1.82	ENSG00000135519	potassium voltage-gated channel subfamily H member 3
HCP5	1.34	1.45	ENSG00000237105	HLA complex P5 (non-protein coding)

KCNAB2	1.34	1.67	ENSG00000069424	potassium voltage-gated channel subfamily A regulatory beta subunit 2
F10	1.34	6.47	ENSG00000126218	coagulation factor X
TUBAL3	1.34	2.18	ENSG00000178462	tubulin alpha like 3
CFH	1.33	2.21	ENSG00000000971	complement factor H
RAD51-AS1	1.33	1.50	ENSG00000245849	RAD51 antisense RNA 1 (head to head)
WBSCR27	1.33	1.50	ENSG00000165171	Williams Beuren syndrome chromosome region 27
RIN2	1.33	1.25	ENSG00000132669	Ras and Rab interactor 2
NPAS1	1.33	4.30	ENSG00000130751	neuronal PAS domain protein 1
MYO15B	1.32	4.10	ENSG00000266714	myosin XVB
FAM105A	1.32	1.31	ENSG00000145569	family with sequence similarity 105 member A
BST2	1.32	8.12	ENSG00000130303	bone marrow stromal cell antigen 2
ITIH1	1.32	4.13	ENSG00000055957	inter-alpha-trypsin inhibitor heavy chain 1
DLG5-AS1	1.32	2.02	ENSG00000233871	DLG5 antisense RNA 1
LINC00467	1.32	2.34	ENSG00000153363	long intergenic non-protein coding RNA 467
EFHD1	1.32	2.20	ENSG00000115468	EF-hand domain family member D1
ALDH3B1	1.32	3.20	ENSG00000006534	aldehyde dehydrogenase 3 family member B1
LGALS3BP	1.32	9.23	ENSG00000108679	galectin 3 binding protein
PCDH17	1.31	1.37	ENSG00000118946	protocadherin 17
TTC39B	1.31	1.37	ENSG00000155158	tetratricopeptide repeat domain 39B
SLC27A1	1.31	4.71	ENSG00000130304	solute carrier family 27 member 1
GARNL3	1.31	1.09	ENSG00000136895	GTPase activating Rap/RanGAP domain like 3
RPLP0P2	1.31	1.09	ENSG00000243742	ribosomal protein lateral stalk subunit P0 pseudogene 2
LTB	1.31	5.82	ENSG00000223448	lymphotoxin beta
EFCAB12	1.31	2.67	ENSG00000172771	EF-hand calcium binding domain 12
RP11-977G19.5	1.31	3.48	ENSG00000258199	N/A
RP11-356B19.11	1.31	1.43	ENSG00000271833	N/A
HABP2	1.31	8.26	ENSG00000148702	hyaluronan binding protein 2

TGFBR3L	1.31	1.70	ENSG00000260001	transforming growth factor beta receptor 3 like
SECTM1	1.31	1.93	ENSG00000141574	secreted and transmembrane 1
CYP27A1	1.31	2.59	ENSG00000135929	cytochrome P450 family 27 subfamily A member 1
ATP5EP2	1.30	1.16	ENSG00000180389	ATP synthase, H+ transporting, mitochondrial F1 complex, epsilon subunit pseudogene 2
C15orf52	1.30	1.16	ENSG00000188549	chromosome 15 open reading frame 52
STK39	1.30	1.16	ENSG00000198648	serine/threonine kinase 39
SEPT3	1.30	1.48	ENSG00000100167	septin 3
CTXN1	1.30	5.13	ENSG00000178531	cortexin 1
HERC6	1.30	3.43	ENSG00000138642	HECT and RLD domain containing E3 ubiquitin protein ligase family member 6
PPM1J	1.29	1.87	ENSG00000155367	protein phosphatase, Mg2+/Mn2+ dependent 1J
LINC00648	1.29	2.29	ENSG00000259129	long intergenic non-protein coding RNA 648
REC8	1.29	3.20	ENSG00000100918	REC8 meiotic recombination protein
SPTBN2	1.29	3.20	ENSG00000173898	spectrin beta, non-erythrocytic 2
MCOLN3	1.29	3.00	ENSG00000055732	mucolipin 3
NEBL	1.29	3.76	ENSG00000078114	nebulette
OAS1	1.29	4.94	ENSG00000089127	2'-5'-oligoadenylate synthetase 1
PDZD7	1.29	2.11	ENSG00000186862	PDZ domain containing 7
TMEM182	1.29	2.11	ENSG00000170417	transmembrane protein 182
CTSH	1.29	4.66	ENSG00000103811	cathepsin H
PGM2L1	1.29	2.78	ENSG00000165434	phosphoglucomutase 2 like 1
PXDC1	1.29	3.66	ENSG00000168994	PX domain containing 1
NXPH4	1.28	3.61	ENSG00000182379	neurexophilin 4
B4GALNT3	1.28	1.73	ENSG00000139044	beta-1,4-N-acetyl-galactosaminyltransferase 3
CADM4	1.28	5.03	ENSG00000105767	cell adhesion molecule 4
AP001048.4	1.28	3.33	ENSG00000223975	N/A
FAM133A	1.28	3.01	ENSG00000179083	family with sequence similarity 133 member A

CACNA1G	1.28	2.03	ENSG00000006283	calcium voltage-gated channel subunit alpha1 G
HTR1D	1.28	2.28	ENSG00000179546	5-hydroxytryptamine receptor 1D
QSOX1	1.27	8.41	ENSG00000116260	quiescin sulfhydryl oxidase 1
PAGE5	1.27	4.30	ENSG00000158639	PAGE family member 5
NBL1	1.27	4.38	ENSG00000158747	neuroblastoma 1, DAN family BMP antagonist
LINC00482	1.27	3.64	ENSG00000185168	long intergenic non-protein coding RNA 482
SMKR1	1.27	1.57	ENSG00000240204	small lysine rich protein 1
NR0B1	1.27	2.17	ENSG00000169297	nuclear receptor subfamily 0 group B member 1
ME3	1.26	1.94	ENSG00000151376	malic enzyme 3
RP5-1148A21.3	1.26	1.94	ENSG00000266680	N/A
PLEKHB1	1.26	4.53	ENSG00000021300	pleckstrin homology domain containing B1
FUNDC2	1.26	6.33	ENSG00000165775	FUN14 domain containing 2
B4GALNT4	1.26	3.97	ENSG00000182272	beta-1,4-N-acetyl-galactosaminyltransferase 4
KANK3	1.26	2.02	ENSG00000186994	KN motif and ankyrin repeat domains 3
VIL1	1.26	7.27	ENSG00000127831	villin 1
COL5A2	1.26	4.57	ENSG00000204262	collagen type V alpha 2 chain
COL4A5	1.25	3.66	ENSG00000188153	collagen type IV alpha 5 chain
PDE9A	1.25	5.65	ENSG00000160191	phosphodiesterase 9A
MATN2	1.25	3.29	ENSG00000132561	matrilin 2
PSMB8	1.25	2.19	ENSG00000235715	proteasome subunit beta 8
P2RX4	1.25	6.51	ENSG00000135124	purinergic receptor P2X 4
RP11-497G19.1	1.24	1.55	ENSG00000257883	N/A
MIR210HG	1.24	3.31	ENSG00000282810	MIR210 host gene
LRRC56	1.24	2.32	ENSG00000161328	leucine rich repeat containing 56
RP11-465B22.3	1.24	2.81	ENSG00000217801	N/A
SNCB	1.24	3.34	ENSG00000074317	synuclein beta
RP11-620J15.3	1.24	3.73	ENSG00000257698	N/A
ARHGAP40	1.24	1.60	ENSG00000124143	Rho GTPase activating protein 40
C2CD4C	1.24	1.60	ENSG00000183186	C2 calcium dependent domain containing 4C

COL11A1	1.24	1.60	ENSG00000060718	collagen type XI alpha 1 chain
CNFN	1.24	4.06	ENSG00000105427	cornifelin
RP11-254F7.2	1.24	1.05	ENSG00000260077	N/A
RP11-571F15.3	1.24	1.05	ENSG00000233668	N/A
TSPAN5	1.24	3.25	ENSG00000168785	tetraspanin 5
TP53TG1	1.23	3.57	ENSG00000182165	TP53 target 1 (non-protein coding)
ADH1A	1.23	2.11	ENSG00000187758	alcohol dehydrogenase 1A (class I), alpha polypeptide
TUBB3	1.23	3.85	ENSG00000258947	tubulin beta 3 class III
RSPH9	1.23	3.34	ENSG00000172426	radial spoke head 9 homolog
TSNAXIP1	1.23	1.74	ENSG00000102904	translin associated factor X interacting protein 1
HIST1H1C	1.23	4.35	ENSG00000187837	histone cluster 1 H1 family member c
GPR35	1.23	2.18	ENSG00000178623	G protein-coupled receptor 35
UACA	1.23	5.41	ENSG00000137831	uveal autoantigen with coiled-coil domains and ankyrin repeats
VAMP8	1.23	8.28	ENSG00000118640	vesicle associated membrane protein 8
CTB-171A8.1	1.23	3.30	ENSG00000266903	N/A
GIPR	1.22	3.17	ENSG00000010310	gastric inhibitory polypeptide receptor
OSCP1	1.22	2.69	ENSG00000116885	organic solute carrier partner 1
FLVCR2	1.22	1.37	ENSG00000119686	feline leukemia virus subgroup C cellular receptor family member 2
ANKDD1A	1.22	2.39	ENSG00000166839	ankyrin repeat and death domain containing 1A
EGLN3	1.22	3.13	ENSG00000129521	egl-9 family hypoxia inducible factor 3
FBLL1	1.22	2.13	ENSG00000188573	fibrillarin-like 1
RGS9BP	1.22	1.53	ENSG00000186326	regulator of G-protein signaling 9 binding protein
NPTXR	1.21	1.77	ENSG00000221890	neuronal pentraxin receptor
C2	1.21	2.38	ENSG00000231543	complement C2
RP11-30J20.1	1.21	1.82	ENSG00000254101	N/A
C4orf48	1.21	6.81	ENSG00000243449	chromosome 4 open reading frame 48

SLC6A16	1.20	1.94	ENSG00000063127	solute carrier family 6 member 16
KCNQ1OT1	1.20	2.55	ENSG00000269821	KCNQ1 opposite strand/antisense transcript 1 (non-protein coding)
LYPD3	1.20	3.30	ENSG00000124466	LY6/PLAUR domain containing 3
CD82	1.20	2.57	ENSG00000085117	CD82 molecule
C12orf75	1.20	4.73	ENSG00000235162	chromosome 12 open reading frame 75
CLIC2	1.20	1.02	ENSG00000155962	chloride intracellular channel 2
CTB-33G10.1	1.20	1.09	ENSG00000243829	N/A
SEMA6B	1.19	5.91	ENSG00000167680	semaphorin 6B
TAX1BP3	1.19	4.18	ENSG00000213977	Tax1 binding protein 3
BDKRB1	1.19	1.29	ENSG00000100739	bradykinin receptor B1
CD19	1.19	1.41	ENSG00000177455	CD19 molecule
CPM	1.19	5.27	ENSG00000135678	carboxypeptidase M
MFSD6	1.19	3.70	ENSG00000151690	major facilitator superfamily domain containing 6
RASSF6	1.19	1.57	ENSG00000169435	Ras association domain family member 6
PRELID2	1.19	3.31	ENSG00000186314	PRELI domain containing 2
GCA	1.19	3.85	ENSG00000115271	grancalcin
AC027612.6	1.18	4.03	ENSG00000143429	N/A
CLDN9	1.18	1.96	ENSG00000213937	claudin 9
APBB1	1.18	5.21	ENSG00000166313	amyloid beta precursor protein binding family B member 1
GOLM1	1.18	4.53	ENSG00000135052	golgi membrane protein 1
MTMR11	1.18	4.61	ENSG00000014914	myotubularin related protein 11
CERS1	1.18	2.24	ENSG00000223802	ceramide synthase 1
BMP1	1.18	5.48	ENSG00000168487	bone morphogenetic protein 1
ESPN	1.18	4.55	ENSG00000187017	espin
TMC4	1.18	2.61	ENSG00000277667	transmembrane channel like 4
ZNF385A	1.17	6.29	ENSG00000161642	zinc finger protein 385A
ALDOC	1.17	4.93	ENSG00000109107	aldolase, fructose-bisphosphate C
KIF17	1.17	4.06	ENSG00000117245	kinesin family member 17
DNM1	1.16	4.64	ENSG00000106976	dynamin 1
TMSB10	1.16	11.39	ENSG00000034510	thymosin beta 10
SERPINI1	1.16	3.04	ENSG00000163536	serpin family I member 1

LINC01057	1.16	2.29	ENSG00000224081	long intergenic non-protein coding RNA 1057
ANKRD1	1.16	2.03	ENSG00000148677	ankyrin repeat domain 1
ZNF175	1.16	1.70	ENSG00000105497	zinc finger protein 175
ZNF397	1.16	1.50	ENSG00000186812	zinc finger protein 397
HAPLN3	1.16	1.27	ENSG00000140511	hyaluronan and proteoglycan link protein 3
RP11-244H3.1	1.16	1.27	ENSG00000241014	N/A
GPR153	1.16	4.16	ENSG00000158292	G protein-coupled receptor 153
RP11-421L21.3	1.16	1.21	ENSG00000233184	N/A
RP11-235E17.6	1.16	1.14	ENSG00000262903	N/A
CHKB-AS1	1.16	1.07	ENSG00000205559	CHKB antisense RNA 1 (head to head)
RRAS	1.16	7.31	ENSG00000126458	related RAS viral (r-ras) oncogene homolog
HHLA3	1.16	5.02	ENSG00000197568	HERV-H LTR-associating 3
BNIP3L	1.15	4.68	ENSG00000104765	BCL2 interacting protein 3 like
RAB37	1.15	4.20	ENSG00000172794	RAB37, member RAS oncogene family
PLCB1	1.15	2.73	ENSG00000182621	phospholipase C beta 1
FBXO2	1.15	5.75	ENSG00000116661	F-box protein 2
PPDPF	1.15	8.05	ENSG00000125534	pancreatic progenitor cell differentiation and proliferation factor
LIMD2	1.15	6.21	ENSG00000136490	LIM domain containing 2
CYP1A1	1.15	7.29	ENSG00000140465	cytochrome P450 family 1 subfamily A member 1
TCEA1	1.14	4.11	ENSG00000187735	transcription elongation factor A1
SAT1	1.14	7.58	ENSG00000130066	spermidine/spermine N1-acetyltransferase 1
GPR89A	1.14	1.77	ENSG00000117262	G protein-coupled receptor 89A
NT5E	1.14	5.08	ENSG00000135318	5'-nucleotidase ecto
VKORC1	1.14	7.02	ENSG00000167397	vitamin K epoxide reductase complex subunit 1
TPBGL	1.14	1.63	ENSG00000261594	trophoblast glycoprotein-like
PLCD1	1.14	3.98	ENSG00000187091	phospholipase C delta 1
DMTN	1.13	3.70	ENSG00000158856	dematin actin binding protein

FTH1P15	1.13	1.53	ENSG00000218980	ferritin heavy chain 1 pseudogene 15
MMP19	1.13	1.53	ENSG00000123342	matrix metallopeptidase 19
SLC6A8	1.13	5.18	ENSG00000130821	solute carrier family 6 member 8
TOM1L2	1.13	4.29	ENSG00000175662	target of myb1 like 2 membrane trafficking protein
DOK6	1.13	1.48	ENSG00000206052	docking protein 6
ITGA10	1.13	1.37	ENSG00000143127	integrin subunit alpha 10
SAMD9	1.13	1.31	ENSG00000205413	sterile alpha motif domain containing 9
SLC46A3	1.13	2.14	ENSG00000139508	solute carrier family 46 member 3
TMC8	1.13	1.18	ENSG00000167895	transmembrane channel like 8
ZNF197	1.13	1.18	ENSG00000281709	zinc finger protein 197
H19	1.12	6.43	ENSG00000130600	H19, imprinted maternally expressed transcript (non-protein coding)
AC010894.3	1.12	2.04	ENSG00000226853	N/A
CSF1	1.12	5.34	ENSG00000184371	colony stimulating factor 1
EGFL7	1.12	6.99	ENSG00000172889	EGF like domain multiple 7
RP11-655M14.13	1.12	1.12	ENSG00000255318	N/A
KREMEN2	1.12	3.22	ENSG00000131650	kringle containing transmembrane protein 2
RPL39	1.12	5.88	ENSG00000198918	ribosomal protein L39
KLC3	1.12	2.49	ENSG00000104892	kinesin light chain 3
NUPR1	1.12	8.47	ENSG00000176046	nuclear protein 1, transcriptional regulator
PTGES3L	1.12	1.04	ENSG00000267060	prostaglandin E synthase 3 like
RP11-368P15.3	1.12	1.04	ENSG00000258985	N/A
RP5-894A10.2	1.12	1.04	ENSG00000244701	N/A
SLC6A10P	1.12	1.88	ENSG00000214617	solute carrier family 6 member 10, pseudogene
NACA3P	1.12	2.41	ENSG00000121089	NACA family member 3 pseudogene
RCN3	1.12	7.03	ENSG00000142552	reticulocalbin 3
SLC1A3	1.12	2.38	ENSG00000079215	solute carrier family 1 member 3
C2orf54	1.12	2.32	ENSG00000172478	chromosome 2 open reading frame 54
LPXN	1.11	2.26	ENSG00000110031	leupaxin
ASPH	1.11	7.35	ENSG00000198363	aspartate beta-hydroxylase

ORAI3	1.11	4.56	ENSG00000175938	ORAI calcium release-activated calcium modulator 3
CYP4F12	1.11	4.38	ENSG00000186204	cytochrome P450 family 4 subfamily F member 12
SYT3	1.11	2.51	ENSG00000213023	synaptotagmin 3
AGPAT4	1.11	3.27	ENSG00000026652	1-acylglycerol-3-phosphate O-acyltransferase 4
AC106876.2	1.11	1.57	ENSG00000222001	N/A
HYAL1	1.11	5.49	ENSG00000114378	hyaluronoglucosaminidase 1
SIGIRR	1.11	6.26	ENSG00000185187	single Ig and TIR domain containing
RP5-1033H22.2	1.10	1.46	ENSG00000224093	N/A
DHRS3	1.10	7.82	ENSG00000162496	dehydrogenase/reductase 3
CD63	1.10	9.89	ENSG00000135404	CD63 molecule
BCAM	1.10	8.61	ENSG00000187244	basal cell adhesion molecule (Lutheran blood group)
SLC9A2	1.10	1.95	ENSG00000115616	solute carrier family 9 member A2
NDUFC2	1.10	6.57	ENSG00000151366	NADH:ubiquinone oxidoreductase subunit C2
RRAGD	1.10	5.04	ENSG00000025039	Ras related GTP binding D
LBP	1.10	3.94	ENSG00000129988	lipopolysaccharide binding protein
STX8	1.10	5.06	ENSG00000170310	syntaxin 8
PFKP	1.10	7.46	ENSG00000067057	phosphofructokinase, platelet
FXYD2	1.10	3.78	ENSG00000137731	FXYD domain containing ion transport regulator 2
POLE4	1.10	4.88	ENSG00000115350	DNA polymerase epsilon 4, accessory subunit
GPX3	1.10	7.66	ENSG00000211445	glutathione peroxidase 3
PDGFB	1.10	3.64	ENSG00000100311	platelet derived growth factor subunit B
TUBA1A	1.10	3.15	ENSG00000167552	tubulin alpha 1a
APLP1	1.10	5.94	ENSG00000105290	amyloid beta precursor like protein 1
FAM174B	1.09	5.81	ENSG00000185442	family with sequence similarity 174 member B
ZDHHC1	1.09	2.15	ENSG00000159714	zinc finger DHHC-type containing 1
PGF	1.09	2.92	ENSG00000119630	placental growth factor
SCNN1A	1.09	2.12	ENSG00000111319	sodium channel epithelial 1 alpha subunit

MYL9	1.09	5.86	ENSG00000101335	myosin light chain 9
CLYBL	1.09	3.38	ENSG00000125246	citrate lyase beta like
UCA1	1.09	8.77	ENSG00000273782	Urothelial cancer associated 1 conserved region
COL21A1	1.09	2.08	ENSG00000124749	collagen type XXI alpha 1 chain
FTH1P23	1.09	1.16	ENSG00000242960	ferritin heavy chain 1 pseudogene 23
ARHGAP18	1.09	2.84	ENSG00000146376	Rho GTPase activating protein 18
CAPG	1.09	1.09	ENSG00000042493	capping actin protein, gelsolin like
RP11-587P21.2	1.09	2.30	ENSG00000257893	N/A
RP13-104F24.3	1.08	1.60	ENSG00000265298	N/A
LINC00205	1.08	3.71	ENSG00000223768	long intergenic non-protein coding RNA 205
COMMD6	1.08	6.27	ENSG00000188243	COMM domain containing 6
STAT1	1.08	6.99	ENSG00000115415	signal transducer and activator of transcription 1
SH2D3A	1.08	2.71	ENSG00000125731	SH2 domain containing 3A
FCGBP	1.08	1.94	ENSG00000281123	Fc fragment of IgG binding protein
CYB5R2	1.08	1.02	ENSG00000166394	cytochrome b5 reductase 2
FAM86B3P	1.08	1.02	ENSG00000173295	family with sequence similarity 86 member B3, pseudogene
SLC30A3	1.08	4.10	ENSG00000115194	solute carrier family 30 member 3
LINC00649	1.08	3.04	ENSG00000237945	long intergenic non-protein coding RNA 649
NRCAM	1.08	2.47	ENSG00000091129	neuronal cell adhesion molecule
SYT17	1.08	2.47	ENSG00000103528	synaptotagmin 17
OCIAD2	1.08	1.50	ENSG00000145247	OCIA domain containing 2
C14orf132	1.08	4.84	ENSG00000227051	chromosome 14 open reading frame 132
PGBD4	1.08	1.86	ENSG00000182405	piggyBac transposable element derived 4
SERPING1	1.08	8.01	ENSG00000149131	serpin family G member 1
RP11-297L17.2	1.07	3.90	ENSG00000260963	N/A
VAMP5	1.07	3.95	ENSG00000168899	vesicle associated membrane protein 5
GOLGA7B	1.07	4.74	ENSG00000155265	golgin A7 family member B
AP001469.9	1.07	2.89	ENSG00000239415	N/A

EFEMP2	1.07	3.84	ENSG00000172638	EGF containing fibulin like extracellular matrix protein 2
ASMTL-AS1	1.07	2.04	ENSG00000236017	ASMTL antisense RNA 1
TF	1.07	12.08	ENSG00000091513	transferrin
DSCR4-IT1	1.07	1.73	ENSG00000223608	DSCR4 intronic transcript 1
XXyac-YM21GA2.4	1.07	1.33	ENSG00000234460	N/A
GPX8	1.07	3.71	ENSG00000164294	glutathione peroxidase 8 (putative)
HK1	1.06	4.33	ENSG00000156515	hexokinase 1
PLXNA2	1.06	4.48	ENSG00000076356	plexin A2
FBXO25	1.06	4.42	ENSG00000147364	F-box protein 25
ITGA2B	1.06	1.27	ENSG00000005961	integrin subunit alpha 2b
RP11-420A23.1	1.06	2.20	ENSG00000251432	N/A
RP11-140K17.3	1.06	3.94	ENSG00000272288	N/A
HCFC1R1	1.06	7.70	ENSG00000103145	host cell factor C1 regulator 1
ZSWIM4	1.06	4.03	ENSG00000132003	zinc finger SWIM-type containing 4
THBS3	1.06	3.83	ENSG00000169231	thrombospondin 3
HDX	1.06	1.20	ENSG00000165259	highly divergent homeobox
RP11-686O6.2	1.06	1.20	ENSG00000273456	N/A
RP11-408P14.1	1.06	1.58	ENSG00000243199	N/A
RNF181	1.06	7.80	ENSG00000168894	ring finger protein 181
TSPAN8	1.06	4.89	ENSG00000127324	tetraspanin 8
PLA2G12B	1.06	3.17	ENSG00000138308	phospholipase A2 group XIIB
LIPH	1.05	5.47	ENSG00000163898	lipase H
RPL39L	1.05	4.01	ENSG00000163923	ribosomal protein L39 like
DSG2	1.05	3.75	ENSG00000046604	desmoglein 2
APOL6	1.05	2.06	ENSG00000221963	apolipoprotein L6
RHOC	1.05	8.70	ENSG00000155366	ras homolog family member C
DTX3	1.05	3.18	ENSG00000178498	deltex E3 ubiquitin ligase 3
SPINT2	1.05	6.91	ENSG00000167642	serine peptidase inhibitor, Kunitz type 2
TMED6	1.05	1.99	ENSG00000157315	transmembrane p24 trafficking protein 6
ETNK2	1.04	4.77	ENSG00000143845	ethanolamine kinase 2
CLVS1	1.04	2.52	ENSG00000177182	clavesin 1
CORO7	1.04	4.31	ENSG00000262246	coronin 7
SLC2A14	1.04	1.95	ENSG00000173262	solute carrier family 2 member 14

LTBP2	1.04	3.32	ENSG00000119681	latent transforming growth factor beta binding protein 2
CTSD	1.04	10.13	ENSG00000117984	cathepsin D
ATP2B4	1.04	2.49	ENSG00000058668	ATPase plasma membrane Ca2+ transporting 4
SERPINC1	1.04	1.91	ENSG00000117601	serpin family C member 1
SMIM1	1.04	3.65	ENSG00000235169	small integral membrane protein 1 (Vel blood group)
MYOF	1.04	3.90	ENSG00000138119	myoferlin
FCHSD1	1.04	5.05	ENSG00000197948	FCH and double SH3 domains 1
FOXQ1	1.04	2.59	ENSG00000164379	forkhead box Q1
EMID1	1.04	2.56	ENSG00000186998	EMI domain containing 1
ABTB1	1.03	4.71	ENSG00000114626	ankyrin repeat and BTB domain containing 1
ABCC3	1.03	3.35	ENSG00000108846	ATP binding cassette subfamily C member 3
SH3BGRL3	1.03	8.29	ENSG00000142669	SH3 domain binding glutamate rich protein like 3
DOK1	1.03	2.21	ENSG00000115325	docking protein 1
MAP4K1	1.03	1.57	ENSG00000282928	mitogen-activated protein kinase kinase kinase kinase 1
RP11-218M22.1	1.03	1.57	ENSG00000177406	N/A
HLA-G	1.03	2.76	ENSG00000235680	major histocompatibility complex, class I, G
MFSD7	1.03	1.25	ENSG00000169026	major facilitator superfamily domain containing 7
HOTAIRM1	1.03	6.06	ENSG00000233429	HOXA transcript antisense RNA, myeloid-specific 1
FOXN3-AS1	1.03	2.17	ENSG00000258920	FOXN3 antisense RNA 1
GNAZ	1.03	2.17	ENSG00000128266	G protein subunit alpha z
TXNRD3	1.02	2.29	ENSG00000197763	thioredoxin reductase 3
HSPB1	1.02	9.88	ENSG00000106211	heat shock protein family B (small) member 1
NTN5	1.02	1.18	ENSG00000142233	netrin 5
RGS9	1.02	1.18	ENSG00000108370	regulator of G-protein signaling 9
CERCAM	1.02	6.57	ENSG00000167123	cerebral endothelial cell adhesion molecule
DNAAF3	1.02	3.58	ENSG00000167646	dynein axonemal assembly factor 3

C20orf196	1.02	2.26	ENSG00000171984	chromosome 20 open reading frame 196
KCNK6	1.02	2.87	ENSG00000099337	potassium two pore domain channel subfamily K member 6
FAHD2CP	1.02	2.53	ENSG00000231584	fumarylacetoacetate hydrolase domain containing 2C, pseudogene
GDA	1.02	5.62	ENSG00000119125	guanine deaminase
KRT19	1.02	3.28	ENSG00000171345	keratin 19
FAXDC2	1.02	2.07	ENSG00000170271	fatty acid hydroxylase domain containing 2
PLEKHG5	1.02	3.03	ENSG00000171680	pleckstrin homology and RhoGEF domain containing G5
LINC00862	1.02	1.11	ENSG00000203721	long intergenic non-protein coding RNA 862
CLDN3	1.02	3.18	ENSG00000165215	claudin 3
RDH12	1.01	1.85	ENSG00000139988	retinol dehydrogenase 12 (all-trans/9-cis/11-cis)
PRRX2	1.01	2.20	ENSG00000167157	paired related homeobox 2
HILPDA	1.01	5.46	ENSG00000135245	hypoxia inducible lipid droplet associated
RBM11	1.01	2.71	ENSG00000185272	RNA binding motif protein 11
PDZD11	1.01	7.30	ENSG00000120509	PDZ domain containing 11
UCHL3	1.01	3.68	ENSG00000118939	ubiquitin C-terminal hydrolase L3
TMEM8B	1.01	4.40	ENSG00000137103	transmembrane protein 8B
SYT5	1.01	5.68	ENSG00000129990	synaptotagmin 5
DEPTOR	1.01	2.89	ENSG00000155792	DEP domain containing MTOR-interacting protein
TMEM187	1.01	2.89	ENSG00000177854	transmembrane protein 187
ST3GAL1	1.01	3.80	ENSG00000008513	ST3 beta-galactoside alpha-2,3-sialyltransferase 1
TM4SF5	1.01	9.01	ENSG00000142484	transmembrane 4 L six family member 5
ADRB1	1.01	1.04	ENSG00000043591	adrenoceptor beta 1
GUCA2A	1.01	1.04	ENSG00000197273	guanylate cyclase activator 2A
RP11-10K16.1	1.01	1.04	ENSG00000245213	N/A
C12orf57	1.01	6.31	ENSG00000111678	chromosome 12 open reading frame 57
TNS1	1.01	3.83	ENSG00000079308	tensin 1

NDUFA13	1.01	5.17	ENSG00000186010	NADH:ubiquinone oxidoreductase subunit A13
OSER1-AS1	1.01	1.96	ENSG00000223891	OSER1 antisense RNA 1 (head to head)
SEPT5	1.01	2.63	ENSG00000184702	septin 5
SLC44A2	1.01	5.91	ENSG00000129353	solute carrier family 44 member 2
TMCC2	1.01	3.23	ENSG00000133069	transmembrane and coiled-coil domain family 2
TPM2	1.00	10.78	ENSG00000198467	tropomyosin 2 (beta)
IGSF1	1.00	3.16	ENSG00000147255	immunoglobulin superfamily member 1
SLC4A4	1.00	3.80	ENSG00000080493	solute carrier family 4 member 4
VASH2	1.00	2.25	ENSG00000143494	vasohibin 2
ERCC6	1.00	2.09	ENSG00000225830	ERCC excision repair 6, chromatin remodeling factor
MICAL2	1.00	2.49	ENSG00000133816	microtubule associated monooxygenase, calponin and LIM domain containing 2
IGFBP4	1.00	4.75	ENSG00000141753	insulin like growth factor binding protein 4
CROCCP3	1.00	1.29	ENSG00000080947	ciliary rootlet coiled-coil, rootletin pseudogene 3
MAP1B	1.00	1.29	ENSG00000131711	microtubule associated protein 1B
ZCCHC24	1.00	3.20	ENSG00000165424	zinc finger CCHC-type containing 24
SULT2B1	1.00	3.99	ENSG00000088002	sulfotransferase family 2B member 1
NPDC1	1.00	2.88	ENSG00000107281	neural proliferation, differentiation and control 1
RP11-503C24.6	1.00	3.55	ENSG00000224417	N/A
RGS20	1.00	3.02	ENSG00000147509	regulator of G-protein signaling 20
NUDT14	1.00	4.19	ENSG00000183828	nudix hydrolase 14
TCEAL3	1.00	1.88	ENSG00000196507	transcription elongation factor A like 3
ANG	1.00	4.99	ENSG00000214274	angiogenin
PARM1	1.00	4.18	ENSG00000169116	prostate androgen-regulated mucin-like protein 1
RP11-758M4.4	0.99	1.22	ENSG00000253706	N/A
ZNF362	0.99	2.98	ENSG00000160094	zinc finger protein 362

RP11-469A15.2	0.99	2.15	ENSG00000230623	N/A
NUDT18	0.99	4.24	ENSG00000275074	nudix hydrolase 18
SLC2A4	0.99	4.03	ENSG00000181856	solute carrier family 2 member 4
SDC3	0.99	5.25	ENSG00000162512	syndecan 3
SLC22A31	0.99	4.71	ENSG00000259803	solute carrier family 22 member 31
VMAC	0.99	1.63	ENSG00000187650	vimentin-type intermediate filament associated coiled-coil protein
SPA17	0.99	2.12	ENSG00000064199	sperm autoantigenic protein 17
ZP3	0.99	3.96	ENSG00000188372	zona pellucida glycoprotein 3
RASGRP3	0.99	3.08	ENSG00000152689	RAS guanyl releasing protein 3
LMX1B	0.99	1.80	ENSG00000136944	LIM homeobox transcription factor 1 beta
GPRASP2	0.99	1.16	ENSG00000158301	G protein-coupled receptor associated sorting protein 2
SYNE1	0.99	2.35	ENSG00000131018	spectrin repeat containing nuclear envelope protein 1
WIPF3	0.99	3.97	ENSG00000122574	WAS/WASL interacting protein family member 3
MFSD2B	0.98	1.39	ENSG00000205639	major facilitator superfamily domain containing 2B
PCSK1N	0.98	8.74	ENSG00000102109	proprotein convertase subtilisin/kexin type 1 inhibitor
GAS2L3	0.98	4.93	ENSG00000139354	growth arrest specific 2 like 3
RTN4RL1	0.98	1.58	ENSG00000185924	reticulon 4 receptor like 1
ABHD12	0.98	7.66	ENSG00000100997	abhydrolase domain containing 12
NES	0.98	3.86	ENSG00000132688	nestin
ARHGEF25	0.98	4.43	ENSG00000240771	Rho guanine nucleotide exchange factor 25
RP11-1L9.1	0.98	2.17	ENSG00000226434	N/A
FSD1	0.98	4.26	ENSG00000105255	fibronectin type III and SPRY domain containing 1
ARG1	0.98	1.09	ENSG00000118520	arginase 1
IGDCC4	0.98	1.09	ENSG00000103742	immunoglobulin superfamily DCC subclass member 4
TIMD4	0.98	1.09	ENSG00000145850	T-cell immunoglobulin and mucin domain containing 4
IDUA	0.98	5.03	ENSG00000127415	iduronidase, alpha-L-

TMEM256	0.98	5.86	ENSG00000205544	transmembrane protein 256
SLC4A11	0.98	5.75	ENSG00000088836	solute carrier family 4 member 11
C12orf60	0.98	1.87	ENSG00000182993	chromosome 12 open reading frame 60
RBM24	0.98	1.87	ENSG00000112183	RNA binding motif protein 24
S100A13	0.97	3.95	ENSG00000189171	S100 calcium binding protein A13
TSPAN15	0.97	5.36	ENSG00000099282	tetraspanin 15
TNFRSF10C	0.97	3.53	ENSG00000173535	TNF receptor superfamily member 10c
UCHL1	0.97	7.05	ENSG00000154277	ubiquitin C-terminal hydrolase L1
CYP2C18	0.97	2.23	ENSG00000108242	cytochrome P450 family 2 subfamily C member 18
KALRN	0.97	1.97	ENSG00000160145	kalirin, RhoGEF kinase
MAPRE3	0.97	4.90	ENSG00000084764	microtubule associated protein RP/EB family member 3
FMO5	0.97	4.18	ENSG00000131781	flavin containing monooxygenase 5
CTD-2162K18.4	0.97	1.27	ENSG00000267260	N/A
IGIP	0.97	1.27	ENSG00000182700	IgA inducing protein
OVOL2	0.97	1.27	ENSG00000125850	ovo like zinc finger 2
C1QL1	0.97	7.76	ENSG00000131094	complement C1q like 1
CD70	0.97	4.35	ENSG00000125726	CD70 molecule
PKD2	0.96	4.05	ENSG00000118762	polycystin 2, transient receptor potential cation channel
RP1-266L20.2	0.96	1.93	ENSG00000281625	N/A
RP13-516M14.1	0.96	2.39	ENSG00000260563	N/A
LAMA3	0.96	5.90	ENSG00000053747	laminin subunit alpha 3
TGFBI	0.96	9.15	ENSG00000120708	transforming growth factor beta induced
RGS10	0.96	4.05	ENSG00000148908	regulator of G-protein signaling 10
PARVA	0.96	3.06	ENSG00000197702	parvin alpha
CDS1	0.96	2.71	ENSG00000163624	CDP-diacylglycerol synthase 1
RIMKLA	0.96	3.11	ENSG00000177181	ribosomal modification protein rimK like family member A
PRRG2	0.96	3.04	ENSG00000126460	proline rich and Gla domain 2
PPM1N	0.96	3.21	ENSG00000213889	protein phosphatase, Mg2+/Mn2+ dependent 1N (putative)

CIB2	0.96	5.86	ENSG00000136425	calcium and integrin binding family member 2
DNASE1	0.95	3.92	ENSG00000213918	deoxyribonuclease 1
AKR1C3	0.95	6.90	ENSG00000196139	aldo-keto reductase family 1 member C3
GPC4	0.95	1.37	ENSG00000076716	glypican 4
TRIM55	0.95	1.37	ENSG00000147573	tripartite motif containing 55
PPP3CA	0.95	4.25	ENSG00000138814	protein phosphatase 3 catalytic subunit alpha
GPR157	0.95	6.56	ENSG00000180758	G protein-coupled receptor 157
NOXA1	0.95	2.79	ENSG00000188747	NADPH oxidase activator 1
FZD2	0.95	5.27	ENSG00000180340	frizzled class receptor 2
SH3RF2	0.95	4.70	ENSG00000156463	SH3 domain containing ring finger 2
CACNG8	0.95	3.15	ENSG00000142408	calcium voltage-gated channel auxiliary subunit gamma 8
S100A11	0.95	7.57	ENSG00000163191	S100 calcium binding protein A11
MR1	0.94	4.12	ENSG00000153029	major histocompatibility complex, class I-related
UPK1B	0.94	3.59	ENSG00000114638	uroplakin 1B
RP11-319G6.1	0.94	1.31	ENSG00000248932	N/A
RAB4B	0.94	5.23	ENSG00000167578	RAB4B, member RAS oncogene family
NPC2	0.94	7.82	ENSG00000119655	NPC intracellular cholesterol transporter 2
DRD4	0.94	2.43	ENSG00000069696	dopamine receptor D4
SEMA7A	0.94	1.77	ENSG00000138623	semaphorin 7A (John Milton Hagen blood group)
TCEAL8	0.94	1.77	ENSG00000180964	transcription elongation factor A like 8
EDN1	0.94	3.68	ENSG00000078401	endothelin 1
RNF39	0.94	1.07	ENSG00000230467	ring finger protein 39
RASIP1	0.94	1.88	ENSG00000105538	Ras interacting protein 1
DGAT2	0.94	4.86	ENSG00000062282	diacylglycerol O-acyltransferase 2
LRRC73	0.93	2.70	ENSG00000204052	leucine rich repeat containing 73
ETFB	0.93	6.38	ENSG00000105379	electron transfer flavoprotein beta subunit
BCHE	0.93	1.24	ENSG00000114200	butyrylcholinesterase

LPCAT2	0.93	1.24	ENSG00000087253	lysophosphatidylcholine acyltransferase 2
ODF3B	0.93	1.24	ENSG00000177989	outer dense fiber of sperm tails 3B
ABLIM2	0.93	2.54	ENSG00000163995	actin binding LIM protein family member 2
HEXB	0.93	7.66	ENSG00000049860	hexosaminidase subunit beta
SLC43A2	0.93	4.46	ENSG00000278550	solute carrier family 43 member 2
CD81	0.93	8.26	ENSG00000110651	CD81 molecule
CGREF1	0.93	3.83	ENSG00000138028	cell growth regulator with EF-hand domain 1
NPNT	0.93	3.86	ENSG00000168743	nephronectin
PLEKHO1	0.93	4.45	ENSG00000023902	pleckstrin homology domain containing O1
RECK	0.93	3.09	ENSG00000122707	reversion inducing cysteine rich protein with kazal motifs
RP4-639F20.1	0.93	3.52	ENSG00000235501	N/A
ISYNA1	0.92	7.22	ENSG00000105655	inositol-3-phosphate synthase 1
ATOX1	0.92	7.65	ENSG00000177556	antioxidant 1 copper chaperone
NDUFB11	0.92	9.01	ENSG00000147123	NADH:ubiquinone oxidoreductase subunit B11
HECA	0.92	2.56	ENSG00000112406	hdc homolog, cell cycle regulator
RP11-14N7.2	0.92	2.23	ENSG00000232527	N/A
TTC39A	0.92	1.80	ENSG00000085831	tetratricopeptide repeat domain 39A
SYTL4	0.92	2.94	ENSG00000102362	synaptotagmin like 4
FBXL8	0.92	1.91	ENSG00000135722	F-box and leucine rich repeat protein 8
NLGN3	0.92	1.91	ENSG00000196338	neuroligin 3
ZNF358	0.92	7.19	ENSG00000198816	zinc finger protein 358
ROBO3	0.92	1.35	ENSG00000154134	roundabout guidance receptor 3
GCNT3	0.92	5.15	ENSG00000140297	glucosaminyl (N-acetyl) transferase 3, mucin type
ACSF2	0.92	6.24	ENSG00000167107	acyl-CoA synthetase family member 2
SERPINF2	0.92	8.71	ENSG00000167711	serpin family F member 2
TXNDC16	0.92	1.63	ENSG00000087301	thioredoxin domain containing 16
NEDD9	0.92	5.65	ENSG00000111859	neural precursor cell expressed, developmentally down-regulated 9

SLC6A12	0.91	5.41	ENSG00000111181	solute carrier family 6 member 12
FKBP1A	0.91	8.50	ENSG00000088832	FK506 binding protein 1A
CRELD1	0.91	5.11	ENSG00000163703	cysteine rich with EGF like domains 1
TMEM151A	0.91	5.44	ENSG00000179292	transmembrane protein 151A
AKR1B1	0.91	4.67	ENSG00000085662	aldo-keto reductase family 1 member B
ZNF93	0.91	1.11	ENSG00000184635	zinc finger protein 93
TMSB4X	0.91	7.61	ENSG00000205542	thymosin beta 4, X-linked
PLOD2	0.91	4.92	ENSG00000152952	procollagen-lysine,2-oxoglutarate 5-dioxygenase 2
NEIL1	0.91	3.54	ENSG00000140398	nei like DNA glycosylase 1
NUCB1	0.91	9.61	ENSG00000104805	nucleobindin 1
SLC16A2	0.91	5.02	ENSG00000147100	solute carrier family 16 member 2
EVA1B	0.91	4.49	ENSG00000142694	eva-1 homolog B
GCKR	0.91	4.49	ENSG00000084734	glucokinase regulator
RP11-103J17.2	0.91	1.29	ENSG00000261761	N/A
STOX1	0.91	1.29	ENSG00000165730	storkhead box 1
AC079922.3	0.91	1.44	ENSG00000237753	N/A
PSMB9	0.91	1.44	ENSG00000239836	proteasome subunit beta 9
NRTN	0.91	5.15	ENSG00000171119	neurturin
DOC2A	0.91	1.58	ENSG00000149927	double C2 domain alpha
C16orf52	0.91	2.91	ENSG00000185716	chromosome 16 open reading frame 52
ANTXR2	0.91	3.65	ENSG00000163297	anthrax toxin receptor 2
TMEM150B	0.91	3.28	ENSG00000180061	transmembrane protein 150B
DLX4	0.91	2.22	ENSG00000108813	distal-less homeobox 4
RP1-159A19.3	0.91	2.22	ENSG00000235912	N/A
MST1L	0.91	2.30	ENSG00000186715	macrophage stimulating 1 like
BAIAP3	0.91	4.33	ENSG00000007516	BAI1 associated protein 3
PLIN2	0.90	7.47	ENSG00000147872	perilipin 2
CST3	0.90	8.44	ENSG00000101439	cystatin C
ARHGEF28	0.90	4.76	ENSG00000214944	Rho guanine nucleotide exchange factor 28
RP11-150O12.3	0.90	2.27	ENSG00000254290	N/A
SNCA	0.90	1.89	ENSG00000145335	synuclein alpha

LINC00944	0.90	1.66	ENSG00000256128	long intergenic non-protein coding RNA 944
PPFIA4	0.90	1.66	ENSG00000143847	PTPRF interacting protein alpha 4
BCAN	0.90	1.38	ENSG00000132692	brevican
C14orf159	0.90	1.38	ENSG00000133943	chromosome 14 open reading frame 159
AGPAT2	0.90	7.59	ENSG00000169692	1-acylglycerol-3-phosphate O-acyltransferase 2
HLA-E	0.90	7.29	ENSG00000225201	major histocompatibility complex, class I, E
TLE2	0.90	5.43	ENSG00000065717	transducin like enhancer of split 2
YIPF1	0.90	5.94	ENSG00000058799	Yip1 domain family member 1
HCAR2	0.90	1.04	ENSG00000182782	hydroxycarboxylic acid receptor 2
PRORSD1P	0.90	1.04	ENSG00000162997	prolyl-tRNA synthetase associated domain containing 1, pseudogene
RP11-359B12.2	0.90	1.04	ENSG00000250132	N/A
PTPRH	0.90	4.65	ENSG00000080031	protein tyrosine phosphatase, receptor type H
RASL11A	0.90	4.22	ENSG00000122035	RAS like family 11 member A
HSD11B1L	0.90	4.83	ENSG00000167733	hydroxysteroid 11-beta dehydrogenase 1 like
DGKA	0.89	3.79	ENSG00000065357	diacylglycerol kinase alpha
FOXD2-AS1	0.89	3.97	ENSG00000237424	FOXD2 antisense RNA 1 (head to head)
TSPEAR-AS1	0.89	1.74	ENSG00000235890	TSPEAR antisense RNA 1
FAM71E1	0.89	1.61	ENSG00000142530	family with sequence similarity 71 member E1
NAT14	0.89	6.40	ENSG00000090971	N-acetyltransferase 14 (putative)
HLA-A	0.89	9.04	ENSG00000224320	major histocompatibility complex, class I, A
FN3K	0.89	5.68	ENSG00000167363	fructosamine 3 kinase
BMP6	0.89	5.71	ENSG00000153162	bone morphogenetic protein 6
RP11-452L6.5	0.89	2.21	ENSG00000260267	N/A
VAMP2	0.89	5.15	ENSG00000220205	vesicle associated membrane protein 2
ZNF880	0.89	1.33	ENSG00000221923	zinc finger protein 880
RP11-404P21.3	0.89	4.34	ENSG00000258793	N/A

CPNE1	0.89	8.64	ENSG00000214078	copine 1
GRN	0.89	9.46	ENSG00000030582	granulin precursor
PCYOX1L	0.89	2.49	ENSG00000145882	prenylcysteine oxidase 1 like
ASAP3	0.89	4.85	ENSG00000282854	ArfGAP with SH3 domain, ankyrin repeat and PH domain 3
PTGR2	0.88	2.34	ENSG00000140043	prostaglandin reductase 2
RP11-274B21.3	0.88	2.66	ENSG00000243679	N/A
AC010524.2	0.88	1.69	ENSG00000268686	N/A
FZD7	0.88	3.27	ENSG00000155760	frizzled class receptor 7
ZFAS1	0.88	6.67	ENSG00000177410	ZNFX1 antisense RNA 1
NDUFA9	0.88	4.91	ENSG00000139180	NADH:ubiquinone oxidoreductase subunit A9
SLC15A1	0.88	5.65	ENSG00000088386	solute carrier family 15 member 1
HLA-C	0.88	8.37	ENSG00000204525	major histocompatibility complex, class I, C
IFT80	0.88	1.56	ENSG00000068885	intraflagellar transport 80
MOB3A	0.88	6.38	ENSG00000172081	MOB kinase activator 3A
FLNA	0.88	9.88	ENSG00000196924	filamin A
AAMDC	0.88	3.47	ENSG00000087884	adipogenesis associated Mth938 domain containing
FABP1	0.88	6.28	ENSG00000163586	fatty acid binding protein 1
ENTPD3-AS1	0.88	1.98	ENSG00000223797	ENTPD3 antisense RNA 1
CCNB1IP1	0.88	5.94	ENSG00000100814	cyclin B1 interacting protein 1
SRP14-AS1	0.88	3.56	ENSG00000248508	SRP14 antisense RNA1 (head to head)
TMEM161B-AS1	0.88	2.57	ENSG00000247828	TMEM161B antisense RNA 1
COL7A1	0.88	4.61	ENSG00000114270	collagen type VII alpha 1 chain
BMP2	0.88	2.23	ENSG00000125845	bone morphogenetic protein 2
PODXL	0.88	3.45	ENSG00000128567	podocalyxin like
RCN1	0.88	5.59	ENSG00000049449	reticulocalbin 1
CCDC17	0.87	1.26	ENSG00000159588	coiled-coil domain containing 17
DMKN	0.87	5.00	ENSG00000161249	dermokine
NEK3	0.87	3.48	ENSG00000136098	NIMA related kinase 3
LAMP3	0.87	2.36	ENSG00000078081	lysosomal associated membrane protein 3

ATP5E	0.87	8.26	ENSG00000124172	ATP synthase, H+ transporting, mitochondrial F1 complex, epsilon subunit
FAT4	0.87	1.09	ENSG00000196159	FAT atypical cadherin 4
ADPRM	0.87	2.48	ENSG00000170222	ADP-ribose/CDP-alcohol diphosphatase, manganese dependent
PRAF2	0.87	6.47	ENSG00000243279	PRA1 domain family member 2
ATP5O	0.87	7.02	ENSG00000241837	ATP synthase, H+ transporting, mitochondrial F1 complex, O subunit
MYO1A	0.87	2.81	ENSG00000166866	myosin IA
CNTN1	0.87	8.06	ENSG00000018236	contactin 1
DHRS4L2	0.87	2.10	ENSG00000187630	dehydrogenase/reductase 4 like 2
ITGAV	0.87	5.56	ENSG00000138448	integrin subunit alpha V
SKAP1	0.87	2.33	ENSG00000141293	src kinase associated phosphoprotein 1
RILP	0.87	3.47	ENSG00000274145	Rab interacting lysosomal protein
GSTO1	0.87	7.25	ENSG00000148834	glutathione S-transferase omega 1
MT1X	0.87	1.72	ENSG00000187193	metallothionein 1X
SHC2	0.87	5.38	ENSG00000129946	SHC adaptor protein 2
ZFHX2	0.87	2.93	ENSG00000136367	zinc finger homeobox 2
TUSC3	0.86	5.81	ENSG00000104723	tumor suppressor candidate 3
RP11-395G23.3	0.86	1.60	ENSG00000254615	N/A
SEMA4B	0.86	7.19	ENSG00000185033	semaphorin 4B
B3GAT3	0.86	7.50	ENSG00000149541	beta-1,3-glucuronyltransferase 3
MRPL23	0.86	6.74	ENSG00000214026	mitochondrial ribosomal protein L23
RP11-421F16.3	0.86	3.20	ENSG00000247903	N/A
RPL3P4	0.86	2.22	ENSG00000232573	ribosomal protein L3 pseudogene 4
FGL1	0.86	4.90	ENSG00000104760	fibrinogen like 1
RHOBTB1	0.86	4.92	ENSG00000072422	Rho related BTB domain containing 1
AFAP1L1	0.86	2.35	ENSG00000157510	actin filament associated protein 1 like 1
HMGCL	0.86	5.93	ENSG00000117305	3-hydroxymethyl-3-methylglutaryl-CoA lyase
OR7E14P	0.86	3.11	ENSG00000184669	olfactory receptor family 7 subfamily E member 14 pseudogene
KIRREL	0.86	1.02	ENSG00000183853	kin of IRRE like (Drosophila)

PALD1	0.86	1.02	ENSG00000107719	phosphatase domain containing, paladin 1
HOXC8	0.86	2.84	ENSG00000037965	homeobox C8
DSE	0.86	3.62	ENSG00000111817	dermatan sulfate epimerase
MTND4P12	0.86	11.37	ENSG00000247627	mitochondrially encoded NADH:ubiquinone oxidoreductase core subunit 4 pseudogene 12
HIST2H2BE	0.85	1.30	ENSG00000184678	histone cluster 2 H2B family member e
EPS8L1	0.85	4.96	ENSG00000131037	EPS8 like 1
FOXO6	0.85	1.55	ENSG00000281518	forkhead box O6
A4GALT	0.85	4.35	ENSG00000128274	alpha 1,4-galactosyltransferase
RNF103	0.85	4.86	ENSG00000239305	ring finger protein 103
C16orf86	0.85	2.09	ENSG00000159761	chromosome 16 open reading frame 86
AKR1C1	0.85	6.61	ENSG00000187134	aldo-keto reductase family 1 member C1
CTSB	0.85	8.45	ENSG00000164733	cathepsin B
GPR137B	0.85	3.96	ENSG00000077585	G protein-coupled receptor 137B
AQP7	0.85	1.40	ENSG00000165269	aquaporin 7
C5orf63	0.85	1.40	ENSG00000164241	chromosome 5 open reading frame 63
GPNMB	0.85	1.40	ENSG00000136235	glycoprotein nmb
ZSCAN30	0.85	1.63	ENSG00000186814	zinc finger and SCAN domain containing 30
GOLGA8B	0.85	4.13	ENSG00000215252	golgin A8 family member B
CTBS	0.84	3.97	ENSG00000117151	chitobiase
ST3GAL5	0.84	4.65	ENSG00000115525	ST3 beta-galactoside alpha-2,3-sialyltransferase 5
ENO3	0.84	5.21	ENSG00000108515	enolase 3
NCALD	0.84	2.34	ENSG00000104490	neurocalcin delta
SCARB2	0.84	7.71	ENSG00000138760	scavenger receptor class B member 2
SGCB	0.84	2.20	ENSG00000163069	sarcoglycan beta
C11orf68	0.84	6.30	ENSG00000175573	chromosome 11 open reading frame 68
RNF208	0.84	4.09	ENSG00000212864	ring finger protein 208
ITGA7	0.84	4.76	ENSG00000135424	integrin subunit alpha 7

MPZL3	0.84	5.13	ENSG00000160588	myelin protein zero like 3
SYNGR3	0.84	4.40	ENSG00000127561	synaptogyrin 3
CRIPT	0.84	4.32	ENSG00000119878	CXXC repeat containing interactor of PDZ3 domain
RTN2	0.84	5.98	ENSG00000125744	reticulon 2
AK7	0.84	2.75	ENSG00000140057	adenylate kinase 7
CDKN2D	0.84	2.75	ENSG00000129355	cyclin dependent kinase inhibitor 2D
XCL1	0.83	1.58	ENSG00000143184	X-C motif chemokine ligand 1
ITGA6	0.83	3.80	ENSG00000091409	integrin subunit alpha 6
PIM1	0.83	3.75	ENSG00000137193	Pim-1 proto-oncogene, serine/threonine kinase
FUT6	0.83	1.34	ENSG00000156413	fucosyltransferase 6
TMEM56	0.83	5.79	ENSG00000152078	transmembrane protein 56
NEAT1	0.83	7.68	ENSG00000245532	nuclear paraspeckle assembly transcript 1 (non-protein coding)
MALAT1	0.83	6.63	ENSG00000278217	Metastasis associated lung adenocarcinoma transcript 1
C11orf74	0.83	4.58	ENSG00000166352	chromosome 11 open reading frame 74
RP11-539L10.3	0.83	1.85	ENSG00000251580	N/A
MICA	0.83	5.96	ENSG00000231225	MHC class I polypeptide-related sequence A
RP11-734K2.4	0.83	1.06	ENSG00000270344	N/A
STXBP2	0.83	6.73	ENSG00000076944	syntaxin binding protein 2
NKD1	0.83	1.66	ENSG00000140807	naked cuticle homolog 1
PRSS23	0.83	6.30	ENSG00000150687	protease, serine 23
NR1D2	0.83	2.08	ENSG00000174738	nuclear receptor subfamily 1 group D member 2
CASC15	0.83	4.40	ENSG00000272168	cancer susceptibility candidate 15 (non-protein coding)
CD24P4	0.83	6.40	ENSG00000185275	CD24 molecule pseudogene 4
STYXL1	0.83	5.63	ENSG00000127952	serine/threonine/tyrosine interacting like 1
SELENBP1	0.83	5.15	ENSG00000143416	selenium binding protein 1
NDN	0.83	3.76	ENSG00000182636	necdin, MAGE family member
RP11-33B1.1	0.83	2.61	ENSG00000245958	N/A
YPEL5	0.82	5.69	ENSG00000119801	yippee like 5

RP11-392P7.6	0.82	2.50	ENSG00000247498	N/A
NIPAL2	0.82	2.65	ENSG00000104361	NIPA like domain containing 2
RAPGEF3	0.82	2.38	ENSG00000079337	Rap guanine nucleotide exchange factor 3
CPQ	0.82	4.78	ENSG00000104324	carboxypeptidase Q
MEGF6	0.82	3.76	ENSG00000162591	multiple EGF like domains 6
CCDC167	0.82	7.13	ENSG00000198937	coiled-coil domain containing 167
SMARCD3	0.82	3.08	ENSG00000082014	SWI/SNF related, matrix associated, actin dependent regulator of chromatin, subfamily d, member 3
TEKT4P2	0.82	1.28	ENSG00000188681	tektin 4 pseudogene 2
SYTL5	0.82	3.18	ENSG00000147041	synaptotagmin like 5
RARRES2P8	0.82	1.61	ENSG00000259996	retinoic acid receptor responder 2 pseudogene 8
ASTN2	0.82	2.10	ENSG00000148219	astrotactin 2
ANKRD24	0.82	2.76	ENSG00000089847	ankyrin repeat domain 24
TMEM101	0.82	5.68	ENSG00000091947	transmembrane protein 101
FAM47E-STBD1	0.82	2.51	ENSG00000272414	FAM47E-STBD1 readthrough
ARHGEF4	0.82	2.35	ENSG00000136002	Rho guanine nucleotide exchange factor 4
ANKHD1	0.81	1.69	ENSG00000131503	ankyrin repeat and KH domain containing 1
ASIC1	0.81	4.76	ENSG00000110881	acid sensing ion channel subunit 1
CCDC113	0.81	3.09	ENSG00000103021	coiled-coil domain containing 113
FHL1	0.81	3.09	ENSG00000022267	four and a half LIM domains 1
RAB40B	0.81	2.21	ENSG00000141542	RAB40B, member RAS oncogene family
TUBA4A	0.81	8.32	ENSG00000127824	tubulin alpha 4a
PDLIM7	0.81	7.48	ENSG00000196923	PDZ and LIM domain 7
H2AFY2	0.81	3.45	ENSG00000099284	H2A histone family member Y2
THNSL1	0.81	2.68	ENSG00000185875	threonine synthase like 1
CALCOCO1	0.81	5.80	ENSG00000012822	calcium binding and coiled-coil domain 1
RP11-390F4.6	0.81	1.84	ENSG00000236924	N/A
PRRG4	0.81	1.11	ENSG00000135378	proline rich and Gla domain 4
LTBP3	0.81	6.16	ENSG00000168056	latent transforming growth factor beta binding protein 3

RP11-23P13.6	0.81	2.13	ENSG00000174171	N/A
JRKL	0.81	1.56	ENSG00000183340	JRK-like
LINC01011	0.81	1.56	ENSG00000244041	long intergenic non-protein coding RNA 1011
PCP4L1	0.81	1.56	ENSG00000248485	Purkinje cell protein 4 like 1
NDUFB7	0.81	8.14	ENSG00000099795	NADH:ubiquinone oxidoreductase subunit B7
APOA2	0.80	11.79	ENSG00000158874	apolipoprotein A2
MORN4	0.80	3.71	ENSG00000171160	MORN repeat containing 4
B3GALNT1	0.80	1.22	ENSG00000169255	beta-1,3-N-acetylgalactosaminyltransferase 1 (globoside blood group)
PRKAR2A-AS1	0.80	1.64	ENSG00000224424	PRKAR2A antisense RNA 1
FKBP7	0.80	4.41	ENSG00000079150	FK506 binding protein 7
TRAPPC1	0.80	7.86	ENSG00000170043	trafficking protein particle complex 1
PROM1	0.80	2.24	ENSG00000007062	prominin 1
SPAG16	0.80	3.36	ENSG00000144451	sperm associated antigen 16
KLHDC8B	0.80	4.68	ENSG00000185909	kelch domain containing 8B
GALNT12	0.80	3.15	ENSG00000119514	polypeptide N-acetylgalactosaminyltransferase 12
SLC4A7	0.80	4.23	ENSG00000033867	solute carrier family 4 member 7
FCGRT	0.80	8.16	ENSG00000104870	Fc fragment of IgG receptor and transporter
DLGAP3	0.80	3.74	ENSG00000116544	DLG associated protein 3
CCDC69	0.80	3.20	ENSG00000198624	coiled-coil domain containing 69
HAP1	0.80	3.07	ENSG00000173805	huntingtin associated protein 1
KIAA1324L	0.80	3.76	ENSG00000164659	KIAA1324 like
RP11-216L13.19	0.80	2.34	ENSG00000273066	N/A
USP49	0.80	2.34	ENSG00000164663	ubiquitin specific peptidase 49
NRM	0.80	6.02	ENSG00000228854	nurim (nuclear envelope membrane protein)
ZNF766	0.80	3.48	ENSG00000196214	zinc finger protein 766
IL1RAP	0.80	2.59	ENSG00000196083	interleukin 1 receptor accessory protein
LINC00993	0.80	1.79	ENSG00000235687	long intergenic non-protein coding RNA 993
TNFAIP8	0.80	1.79	ENSG00000145779	TNF alpha induced protein 8

AGAP2-AS1	0.80	2.80	ENSG00000255737	AGAP2 antisense RNA 1
LTBP1	0.80	3.98	ENSG00000049323	latent transforming growth factor beta binding protein 1
GALC	0.80	3.42	ENSG00000054983	galactosylceramidase
AAK1	0.80	2.15	ENSG00000115977	AP2 associated kinase 1
AF277315.13	0.80	1.42	ENSG00000226141	N/A
SMPD3	0.80	1.42	ENSG00000103056	sphingomyelin phosphodiesterase 3
UQCC2	0.80	7.06	ENSG00000137288	ubiquinol-cytochrome c reductase complex assembly factor 2
RP11-425L10.1	0.80	2.43	ENSG00000244313	N/A
CD151	0.80	9.12	ENSG00000177697	CD151 molecule (Raph blood group)
JAK1	0.80	7.75	ENSG00000162434	Janus kinase 1
TRIM16L	0.79	1.86	ENSG00000108448	tripartite motif containing 16-like
RGS3	0.79	2.87	ENSG00000138835	regulator of G-protein signaling 3
MGMT	0.79	5.02	ENSG00000170430	O-6-methylguanine-DNA methyltransferase
TMEM160	0.79	6.57	ENSG00000130748	transmembrane protein 160
CGNL1	0.79	3.29	ENSG00000128849	cingulin like 1
OGFOD3	0.79	5.55	ENSG00000181396	2-oxoglutarate and iron dependent oxygenase domain containing 3
DSCR4	0.79	4.14	ENSG00000184029	Down syndrome critical region 4
CREBRF	0.79	2.06	ENSG00000164463	CREB3 regulatory factor
CALN1	0.79	1.04	ENSG00000183166	calneuron 1
ITM2A	0.79	1.04	ENSG00000078596	integral membrane protein 2A
MVP	0.79	7.14	ENSG00000013364	major vault protein
ARMC12	0.79	2.40	ENSG00000157343	armadillo repeat containing 12
TMEM38A	0.79	4.63	ENSG00000072954	transmembrane protein 38A
SLC27A2	0.79	3.27	ENSG00000140284	solute carrier family 27 member 2
CIRBP	0.79	5.97	ENSG00000099622	cold inducible RNA binding protein
ADAM15	0.79	7.23	ENSG00000143537	ADAM metallopeptidase domain 15
ADHFE1	0.79	3.48	ENSG00000147576	alcohol dehydrogenase, iron containing 1
COL27A1	0.79	4.26	ENSG00000196739	collagen type XXVII alpha 1 chain
GPR161	0.79	3.99	ENSG00000143147	G protein-coupled receptor 161
ECHDC3	0.79	1.16	ENSG00000134463	enoyl-CoA hydratase domain containing 3
SMIM19	0.78	3.57	ENSG00000176209	small integral membrane protein 19

SPDYC	0.78	3.08	ENSG00000204710	speedy/RINGO cell cycle regulator family member C
SP110	0.78	3.44	ENSG00000135899	SP110 nuclear body protein
NUAK2	0.78	5.47	ENSG00000163545	NUAK family kinase 2
CSPG5	0.78	3.89	ENSG00000114646	chondroitin sulfate proteoglycan 5
PLA2G4C	0.78	3.10	ENSG00000105499	phospholipase A2 group IVC
SPAG6	0.78	3.79	ENSG00000077327	sperm associated antigen 6
PAIP2B	0.78	3.16	ENSG00000124374	poly(A) binding protein interacting protein 2B
RASSF4	0.78	4.90	ENSG00000107551	Ras association domain family member 4
TTC21A	0.78	1.96	ENSG00000168026	tetratricopeptide repeat domain 21A
RGAG4	0.78	3.99	ENSG00000242732	retrotransposon gag domain containing 4
RP11-968A15.2	0.78	1.36	ENSG00000257596	N/A
ZNF350	0.78	1.36	ENSG00000256683	zinc finger protein 350
FAM50A	0.78	8.71	ENSG00000071859	family with sequence similarity 50 member A
NDUFA3	0.78	8.27	ENSG00000276061	NADH:ubiquinone oxidoreductase subunit A3
ZSWIM5	0.78	3.32	ENSG00000162415	zinc finger SWIM-type containing 5
TRANK1	0.78	2.08	ENSG00000168016	tetratricopeptide repeat and ankyrin repeat containing 1
FAM127A	0.78	8.17	ENSG00000134590	family with sequence similarity 127 member A
TJP3	0.78	4.45	ENSG00000105289	tight junction protein 3
ITIH2	0.78	9.06	ENSG00000151655	inter-alpha-trypsin inhibitor heavy chain 2
F13B	0.78	1.54	ENSG00000143278	coagulation factor XIII B chain
SSPO	0.78	1.54	ENSG00000197558	SCO-spondin
CRHR1-IT1	0.78	4.08	ENSG00000282171	CRHR1 intronic transcript 1
DLG4	0.78	5.10	ENSG00000132535	discs large MAGUK scaffold protein 4
RIMS3	0.78	3.98	ENSG00000117016	regulating synaptic membrane exocytosis 3
CDON	0.78	2.98	ENSG00000064309	cell adhesion associated, oncogene regulated
SLC25A35	0.77	5.08	ENSG00000125434	solute carrier family 25 member 35

AC062029.1	0.77	1.70	ENSG00000234028	N/A
SIL1	0.77	6.58	ENSG00000120725	SIL1 nucleotide exchange factor
KRT20	0.77	2.35	ENSG00000171431	keratin 20
MYRF	0.77	6.00	ENSG00000124920	myelin regulatory factor
RASGEF1A	0.77	3.88	ENSG00000198915	RasGEF domain family member 1A
SERPINB6	0.77	8.08	ENSG00000124570	serpin family B member 6
ZSWIM6	0.77	1.92	ENSG00000130449	zinc finger SWIM-type containing 6
MTND1P23	0.77	10.30	ENSG00000225972	mitochondrially encoded NADH:ubiquinone oxidoreductase core subunit 1 pseudogene 23
RP11-446N19.1	0.77	1.98	ENSG00000272369	N/A
ZNF846	0.77	1.98	ENSG00000196605	zinc finger protein 846
RP11-660L16.2	0.77	3.36	ENSG00000254682	N/A
CAMTA1	0.77	5.83	ENSG00000171735	calmodulin binding transcription activator 1
KIAA1462	0.77	3.05	ENSG00000165757	KIAA1462
HES4	0.77	6.02	ENSG00000188290	hes family bHLH transcription factor 4
ADCK1	0.77	4.59	ENSG00000063761	aarF domain containing kinase 1
CAPS	0.77	3.19	ENSG00000105519	calcyphosine
DNM1P35	0.77	1.09	ENSG00000246877	dynamin 1 pseudogene 35
RP1-140A9.1	0.77	1.09	ENSG00000231050	N/A
RP11-888D10.4	0.77	1.09	ENSG00000273284	N/A
C4orf19	0.77	1.20	ENSG00000154274	chromosome 4 open reading frame 19
LGALS8-AS1	0.77	1.20	ENSG00000223776	LGALS8 antisense RNA 1
FAM129A	0.76	1.30	ENSG00000135842	family with sequence similarity 129 member A
DDR1	0.76	6.86	ENSG00000230456	discoidin domain receptor tyrosine kinase 1
SPTBN4	0.76	3.85	ENSG00000160460	spectrin beta, non-erythrocytic 4
HIST1H2BK	0.76	3.09	ENSG00000197903	histone cluster 1 H2B family member k
PSMB10	0.76	1.40	ENSG00000205220	proteasome subunit beta 10
THEMIS2	0.76	1.40	ENSG00000130775	thymocyte selection associated family member 2
MIR940	0.76	1.49	ENSG00000260778	microRNA 940

LMBR1L	0.76	5.76	ENSG00000139636	limb development membrane protein 1 like
MT2A	0.76	3.75	ENSG00000125148	metallothionein 2A
ATP8B3	0.76	2.71	ENSG00000130270	ATPase phospholipid transporting 8B3
WDR83OS	0.76	6.64	ENSG00000105583	WD repeat domain 83 opposite strand
SYBU	0.76	1.66	ENSG00000147642	syntabulin
PLBD1	0.76	6.24	ENSG00000121316	phospholipase B domain containing 1
REEP5	0.76	7.48	ENSG00000129625	receptor accessory protein 5
WNT10B	0.76	3.56	ENSG00000169884	Wnt family member 10B
TESC	0.76	8.21	ENSG00000088992	tescalcin
INCA1	0.76	1.94	ENSG00000196388	inhibitor of CDK, cyclin A1 interacting protein 1
WAS	0.76	1.94	ENSG00000015285	Wiskott-Aldrich syndrome
WWTR1	0.76	3.74	ENSG00000018408	WW domain containing transcription regulator 1
FAM213B	0.76	5.05	ENSG00000275125	family with sequence similarity 213 member B
MAPK11	0.76	5.06	ENSG00000185386	mitogen-activated protein kinase 11
ISCA2	0.76	4.85	ENSG00000165898	iron-sulfur cluster assembly 2
MIR24-2	0.76	4.28	ENSG00000267519	microRNA 24-2
FNDC3B	0.76	7.60	ENSG00000075420	fibronectin type III domain containing 3B
SHISA4	0.76	4.68	ENSG00000198892	shisa family member 4
RABL2A	0.76	2.23	ENSG00000144134	RAB, member of RAS oncogene family-like 2A
EXD3	0.76	3.39	ENSG00000187609	exonuclease 3'-5' domain containing 3
FBXL2	0.76	2.29	ENSG00000153558	F-box and leucine rich repeat protein 2
SMOC1	0.76	2.34	ENSG00000198732	SPARC related modular calcium binding 1
RP5-882C2.2	0.76	2.38	ENSG00000260793	N/A
OCEL1	0.76	5.98	ENSG00000099330	occludin/ELL domain containing 1
SP140L	0.76	4.69	ENSG00000185404	SP140 nuclear body protein like

PNPLA8	0.76	3.67	ENSG00000135241	patatin like phospholipase domain containing 8
DSCR10	0.76	2.68	ENSG00000233316	Down syndrome critical region 10 (non-protein coding)
CTSZ	0.75	5.26	ENSG00000101160	cathepsin Z
NT5M	0.75	2.80	ENSG00000205309	5',3'-nucleotidase, mitochondrial
PCMTD1	0.75	2.90	ENSG00000168300	protein-L-isoaspartate (D-aspartate) O-methyltransferase domain containing 1
AHNAK2	0.75	4.18	ENSG00000185567	AHNAK nucleoprotein 2
CD59	0.75	6.57	ENSG00000085063	CD59 molecule
ADM	0.75	4.44	ENSG00000148926	adrenomedullin
SUPT3H	0.75	3.45	ENSG00000196284	SPT3 homolog, SAGA and STAGA complex component
CEBPA-AS1	0.75	3.58	ENSG00000267296	CEBPA antisense RNA 1 (head to head)
RPS27L	0.75	6.42	ENSG00000185088	ribosomal protein S27 like
AKR1C2	0.75	7.70	ENSG00000151632	aldo-keto reductase family 1 member C2
FAM114A1	0.75	5.61	ENSG00000197712	family with sequence similarity 114 member A1
ISG20	0.75	5.67	ENSG00000172183	interferon stimulated exonuclease gene 20
MARCH2	0.75	5.25	ENSG00000099785	membrane associated ring-CH-type finger 2
TYRO3	0.75	4.85	ENSG00000092445	TYRO3 protein tyrosine kinase
RMI2	0.75	4.56	ENSG00000175643	RecQ mediated genome instability 2
GPSM1	0.75	4.12	ENSG00000160360	G-protein signaling modulator 1
COX7B2	0.75	3.71	ENSG00000170516	cytochrome c oxidase subunit 7B2
GDPD5	0.75	3.57	ENSG00000158555	glycerophosphodiester phosphodiesterase domain containing 5
ITGA2	0.75	2.03	ENSG00000164171	integrin subunit alpha 2
RHPN1-AS1	0.75	1.97	ENSG00000254389	RHPN1 antisense RNA 1 (head to head)
RP1-150O5.3	0.75	1.90	ENSG00000282990	N/A
COPG2	0.75	1.76	ENSG00000158623	coatomer protein complex subunit gamma 2

CTD-2227E11.1	0.75	1.76	ENSG00000270933	N/A
C1QL4	0.75	5.46	ENSG00000186897	complement C1q like 4
NPW	0.75	1.69	ENSG00000183971	neuropeptide W
ZNF837	0.75	1.69	ENSG00000152475	zinc finger protein 837
BBS12	0.75	1.53	ENSG00000181004	Bardet-Biedl syndrome 12
RP11-658F2.8	0.75	1.44	ENSG00000258297	N/A
ZGLP1	0.75	1.44	ENSG00000220201	zinc finger, GATA-like protein 1
DLG3	0.75	1.34	ENSG00000082458	discs large MAGUK scaffold protein 3
TMEM191A	0.75	1.34	ENSG00000226287	transmembrane protein 191A (pseudogene)
IDH1-AS1	0.75	1.13	ENSG00000231908	IDH1 antisense RNA 1
GINM1	0.75	5.71	ENSG00000055211	glycoprotein integral membrane 1
ALB	0.74	6.06	ENSG00000163631	albumin
GTF3C6	0.74	6.70	ENSG00000155115	general transcription factor IIIC subunit 6
ARSA	0.74	6.12	ENSG00000100299	arylsulfatase A
KCNC4	0.74	3.96	ENSG00000116396	potassium voltage-gated channel subfamily C member 4
NIPSNAP3A	0.74	3.05	ENSG00000136783	nipsnap homolog 3A
G6PC3	0.74	7.76	ENSG00000141349	glucose-6-phosphatase catalytic subunit 3
TAGLN	0.74	2.93	ENSG00000149591	transgelin
RELN	0.74	8.84	ENSG00000189056	reelin
CSRP1	0.74	5.50	ENSG00000159176	cysteine and glycine rich protein 1
AIF1L	0.74	2.55	ENSG00000126878	allograft inflammatory factor 1 like
DYNLRB1	0.74	9.00	ENSG00000125971	dynein light chain roadblock-type 1
SPATA20	0.74	5.23	ENSG00000006282	spermatogenesis associated 20
AP2S1	0.74	8.83	ENSG00000042753	adaptor related protein complex 2 sigma 1 subunit
PPM1M	0.74	4.21	ENSG00000164088	protein phosphatase, Mg2+/Mn2+ dependent 1M
ERRFI1	0.74	5.48	ENSG00000116285	ERBB receptor feedback inhibitor 1
NPHP1	0.74	2.22	ENSG00000144061	nephrocystin 1
RP11-793H13.11	0.74	2.11	ENSG00000270175	N/A
HLA-DMB	0.74	2.91	ENSG00000241674	major histocompatibility complex, class II, DM beta

RPS23P8	0.74	1.99	ENSG00000230629	ribosomal protein S23 pseudogene 8
AC007773.2	0.74	2.80	ENSG00000267213	N/A
DNAJC15	0.74	3.95	ENSG00000120675	DnaJ heat shock protein family (Hsp40) member C15
ZNF83	0.74	2.69	ENSG00000167766	zinc finger protein 83
ZNF774	0.74	1.79	ENSG00000196391	zinc finger protein 774
RDM1	0.73	2.61	ENSG00000278023	RAD52 motif containing 1
EIF4BP7	0.73	1.72	ENSG00000225031	eukaryotic translation initiation factor 4B pseudogene 7
PIK3AP1	0.73	3.51	ENSG00000155629	phosphoinositide-3-kinase adaptor protein 1
FGG	0.73	11.50	ENSG00000171557	fibrinogen gamma chain
CD47	0.73	4.50	ENSG00000196776	CD47 molecule
RP1-197B17.3	0.73	1.56	ENSG00000257433	N/A
KDSR	0.73	5.11	ENSG00000119537	3-ketodihydrosphingosine reductase
TST	0.73	7.00	ENSG00000128311	thiosulfate sulfurtransferase
FNDC4	0.73	4.96	ENSG00000115226	fibronectin type III domain containing 4
TMEM205	0.73	7.93	ENSG00000105518	transmembrane protein 205
HSD17B14	0.73	6.90	ENSG00000087076	hydroxysteroid 17-beta dehydrogenase 14
CPE	0.73	4.39	ENSG00000109472	carboxypeptidase E
PRDX5	0.73	9.66	ENSG00000126432	peroxiredoxin 5
HOGA1	0.73	2.82	ENSG00000241935	4-hydroxy-2-oxoglutarate aldolase 1
RP4-625H18.2	0.73	1.38	ENSG00000228412	N/A
TSPAN9	0.73	5.69	ENSG00000011105	tetraspanin 9
YIPF2	0.73	6.42	ENSG00000130733	Yip1 domain family member 2
PALM	0.73	6.55	ENSG00000099864	paralemmin
RP11-138A9.2	0.73	1.28	ENSG00000273319	N/A
TMEM121	0.73	1.28	ENSG00000184986	transmembrane protein 121
PSME1	0.73	7.02	ENSG00000092010	proteasome activator subunit 1
PPP2R3A	0.73	2.63	ENSG00000073711	protein phosphatase 2 regulatory subunit B"alpha
EREG	0.73	5.76	ENSG00000124882	epiregulin
NTAN1	0.73	5.61	ENSG00000275779	N-terminal asparagine amidase
SLC8A2	0.73	2.02	ENSG00000118160	solute carrier family 8 member A2
FBXO10	0.73	4.96	ENSG00000147912	F-box protein 10

PHEX	0.73	2.50	ENSG00000102174	phosphate regulating endopeptidase homolog, X-linked
SPC24	0.73	5.04	ENSG00000161888	SPC24, NDC80 kinetochore complex component
DYNLT1	0.73	7.20	ENSG00000146425	dynein light chain Tctex-type 1
RPL38	0.72	9.39	ENSG00000172809	ribosomal protein L38
ENKD1	0.72	4.55	ENSG00000124074	enkurin domain containing 1
ANKRD34A	0.72	3.89	ENSG00000272031	ankyrin repeat domain 34A
OAS3	0.72	4.85	ENSG00000111331	2'-5'-oligoadenylate synthetase 3
TIPARP	0.72	4.45	ENSG00000163659	TCDD inducible poly(ADP-ribose) polymerase
SEMA3B	0.72	2.98	ENSG00000012171	semaphorin 3B
TRADD	0.72	4.99	ENSG00000102871	TNFRSF1A associated via death domain
DUSP23	0.72	4.77	ENSG00000158716	dual specificity phosphatase 23
BAIAP2-AS1	0.72	4.17	ENSG00000226137	BAIAP2 antisense RNA 1 (head to head)
FAM46A	0.72	6.24	ENSG00000112773	family with sequence similarity 46 member A
COCH	0.72	2.60	ENSG00000100473	cochlin
ABHD14A	0.72	5.05	ENSG00000248487	abhydrolase domain containing 14A
HOXB9	0.72	2.16	ENSG00000170689	homeobox B9
ANXA6	0.72	5.17	ENSG00000197043	annexin A6
GATA3-AS1	0.72	2.52	ENSG00000197308	GATA3 antisense RNA 1
HPX	0.72	7.17	ENSG00000110169	hemopexin
DNAH17	0.72	1.59	ENSG00000187775	dynein axonemal heavy chain 17
SOX4	0.72	5.10	ENSG00000124766	SRY-box 4
ERAP2	0.72	3.99	ENSG00000164308	endoplasmic reticulum aminopeptidase 2
ASB4	0.72	3.39	ENSG00000005981	ankyrin repeat and SOCS box containing 4
SERPINB1	0.72	6.68	ENSG00000021355	serpin family B member 1
TRIM29	0.72	1.51	ENSG00000137699	tripartite motif containing 29
MEF2B	0.72	2.38	ENSG00000213999	myocyte enhancer factor 2B
VOPP1	0.72	4.31	ENSG00000154978	vesicular, overexpressed in cancer, prosurvival protein 1

FAM229B	0.72	4.21	ENSG00000203778	family with sequence similarity 229 member B
MCTP1	0.72	1.98	ENSG00000175471	multiple C2 and transmembrane domain containing 1
KIF12	0.72	5.61	ENSG00000136883	kinesin family member 12
ASMTL	0.72	7.29	ENSG00000169093	acetylserotonin O-methyltransferase-like
ATP6V1F	0.72	8.22	ENSG00000128524	ATPase H+ transporting V1 subunit F
TCF21	0.72	4.91	ENSG00000118526	transcription factor 21
TPT1	0.72	9.09	ENSG00000133112	tumor protein, translationally-controlled 1
SMCO4	0.71	5.50	ENSG00000166002	single-pass membrane protein with coiled-coil domains 4
LEPROTL1	0.71	5.90	ENSG00000104660	leptin receptor overlapping transcript-like 1
RP11-465N4.4	0.71	3.03	ENSG00000234678	N/A
LGALS3	0.71	5.48	ENSG00000131981	galectin 3
RP11-36C20.1	0.71	2.23	ENSG00000240376	N/A
CCDC28B	0.71	3.35	ENSG00000160050	coiled-coil domain containing 28B
UGGT2	0.71	4.55	ENSG00000102595	UDP-glucose glycoprotein glucosyltransferase 2
FAM214B	0.71	6.08	ENSG00000005238	family with sequence similarity 214 member B
C21orf59	0.71	6.20	ENSG00000159079	chromosome 21 open reading frame 59
TMEM167B	0.71	4.53	ENSG00000215717	transmembrane protein 167B
MDK	0.71	9.12	ENSG00000110492	midkine (neurite growth-promoting factor 2)
CITF22-92A6.1	0.71	2.49	ENSG00000273145	N/A
GALK1	0.71	6.40	ENSG00000108479	galactokinase 1
IRS2	0.71	3.30	ENSG00000185950	insulin receptor substrate 2
KCTD13	0.71	5.06	ENSG00000174943	potassium channel tetramerization domain containing 13
ST3GAL3	0.71	3.10	ENSG00000126091	ST3 beta-galactoside alpha-2,3-sialyltransferase 3
LIN7B	0.71	4.31	ENSG00000104863	lin-7 homolog B, crumbs cell polarity complex component

AHNAK	0.71	7.76	ENSG00000124942	AHNAK nucleoprotein
CAHM	0.71	1.22	ENSG00000270419	colon adenocarcinoma hypermethylated (non-protein coding)
PHF11	0.71	3.07	ENSG00000136147	PHD finger protein 11
ARHGAP4	0.71	1.70	ENSG00000089820	Rho GTPase activating protein 4
TMEM107	0.71	2.64	ENSG00000179029	transmembrane protein 107
RHPN1	0.71	5.58	ENSG00000158106	rhophilin Rho GTPase binding protein 1
ATP9A	0.71	5.27	ENSG00000054793	ATPase phospholipid transporting 9A (putative)
GS1-393G12.12	0.71	2.07	ENSG00000254690	N/A
GADD45A	0.71	3.90	ENSG00000116717	growth arrest and DNA damage inducible alpha
C20orf96	0.71	4.23	ENSG00000196476	chromosome 20 open reading frame 96
DBNDD1	0.71	6.01	ENSG00000003249	dysbindin domain containing 1
FKBP2	0.71	8.23	ENSG00000173486	FK506 binding protein 2
ZNF524	0.71	4.77	ENSG00000171443	zinc finger protein 524
CTD-2015H6.3	0.71	2.60	ENSG00000249042	N/A
CRTAP	0.71	8.45	ENSG00000170275	cartilage associated protein
COX7A2	0.71	8.33	ENSG00000112695	cytochrome c oxidase subunit 7A2
CPED1	0.71	1.11	ENSG00000106034	cadherin like and PC-esterase domain containing 1
KB-1440D3.13	0.71	1.11	ENSG00000272954	N/A
ARHGEF17	0.70	2.73	ENSG00000110237	Rho guanine nucleotide exchange factor 17
SORT1	0.70	6.56	ENSG00000134243	sortilin 1
ABCD1	0.70	7.10	ENSG00000101986	ATP binding cassette subfamily D member 1
MYL6	0.70	9.83	ENSG00000092841	myosin light chain 6
TMEM234	0.70	3.95	ENSG00000160055	transmembrane protein 234
TMA7	0.70	4.24	ENSG00000232112	translation machinery associated 7 homolog
RBM20	0.70	1.54	ENSG00000203867	RNA binding motif protein 20
KCNMB4	0.70	2.20	ENSG00000135643	potassium calcium-activated channel subfamily M regulatory beta subunit 4

ITIH5	0.70	4.20	ENSG00000123243	inter-alpha-trypsin inhibitor heavy chain family member 5
PIGU	0.70	6.97	ENSG00000101464	phosphatidylinositol glycan anchor biosynthesis class U
MLF1	0.70	4.37	ENSG00000178053	myeloid leukemia factor 1
FKBP8	0.70	9.30	ENSG00000105701	FK506 binding protein 8
TUB	0.70	2.61	ENSG00000166402	tubby bipartite transcription factor
EHHADH	0.70	2.15	ENSG00000113790	enoyl-CoA hydratase and 3-hydroxyacyl CoA dehydrogenase
TFEB	0.70	3.58	ENSG00000112561	transcription factor EB
FUOM	0.70	5.94	ENSG00000148803	fucose mutarotase
DHRS1	0.70	5.61	ENSG00000157379	dehydrogenase/reductase 1
COX6C	0.70	6.81	ENSG00000164919	cytochrome c oxidase subunit 6C
CFDP1	0.70	5.73	ENSG00000153774	craniofacial development protein 1
BX322557.10	0.70	1.81	ENSG00000215447	N/A
TBC1D19	0.70	1.81	ENSG00000109680	TBC1 domain family member 19
COMMD1	0.70	5.10	ENSG00000173163	copper metabolism domain containing 1
OLFM2	0.70	6.30	ENSG00000105088	olfactomedin 2
CYP2U1	0.70	2.09	ENSG00000155016	cytochrome P450 family 2 subfamily U member 1
ZNF493	0.70	2.09	ENSG00000196268	zinc finger protein 493
CREG1	0.70	6.41	ENSG00000143162	cellular repressor of E1A stimulated genes 1
PPP1R14A	0.70	3.41	ENSG00000167641	protein phosphatase 1 regulatory inhibitor subunit 14A
C19orf81	0.70	4.22	ENSG00000235034	chromosome 19 open reading frame 81
SHFM1	0.70	6.72	ENSG00000127922	split hand/foot malformation (ectrodactyly) type 1
FGF12	0.70	4.05	ENSG00000114279	fibroblast growth factor 12
CPAMD8	0.70	1.36	ENSG00000160111	C3 and PZP like, alpha-2-macroglobulin domain containing 8
B3GNT4	0.70	2.67	ENSG00000176383	UDP-GlcNAc:betaGal beta-1,3-N-acetylglucosaminyltransferase 4
PAGE2	0.70	4.15	ENSG00000234068	PAGE family member 2
ARL6IP5	0.69	4.88	ENSG00000144746	ADP ribosylation factor like GTPase 6 interacting protein 5

HOXA-AS2	0.69	5.16	ENSG00000253552	HOXA cluster antisense RNA 2
ADAMTSL4	0.69	6.65	ENSG00000143382	ADAMTS like 4
FSTL3	0.69	6.54	ENSG00000070404	follistatin like 3
CCDC92	0.69	5.65	ENSG00000275035	coiled-coil domain containing 92
PLCG2	0.69	3.05	ENSG00000197943	phospholipase C gamma 2
BLOC1S1	0.69	6.31	ENSG00000135441	biogenesis of lysosomal organelles complex 1 subunit 1
CLTB	0.69	8.27	ENSG00000175416	clathrin light chain B
ADAM19	0.69	6.01	ENSG00000135074	ADAM metallopeptidase domain 19
CLDN12	0.69	4.59	ENSG00000157224	claudin 12
ADPRHL1	0.69	3.96	ENSG00000153531	ADP-ribosylhydrolase like 1
DHRS12	0.69	3.09	ENSG00000102796	dehydrogenase/reductase 12
C8A	0.69	1.58	ENSG00000157131	complement C8 alpha chain
ENTPD8	0.69	1.58	ENSG00000188833	ectonucleoside triphosphate diphosphohydrolase 8
KCNH6	0.69	1.58	ENSG00000173826	potassium voltage-gated channel subfamily H member 6
RNF11	0.69	4.47	ENSG00000123091	ring finger protein 11
MRPL28	0.69	7.73	ENSG00000086504	mitochondrial ribosomal protein L28
KPTN	0.69	4.84	ENSG00000118162	kaptin, actin binding protein
ASB13	0.69	5.69	ENSG00000196372	ankyrin repeat and SOCS box containing 13
ANKZF1	0.69	5.87	ENSG00000163516	ankyrin repeat and zinc finger domain containing 1
TMEM59	0.69	6.89	ENSG00000116209	transmembrane protein 59
SEMA4D	0.69	3.70	ENSG00000187764	semaphorin 4D
RP11-73M18.8	0.69	2.64	ENSG00000269958	N/A
NDRG4	0.69	2.29	ENSG00000103034	NDRG family member 4
RTBDN	0.69	1.83	ENSG00000132026	retbindin
LGALS9	0.69	1.15	ENSG00000168961	galectin 9
ZFYVE1	0.69	4.18	ENSG00000165861	zinc finger FYVE-type containing 1
SLC9A5	0.69	2.05	ENSG00000135740	solute carrier family 9 member A5
INSR	0.69	6.05	ENSG00000171105	insulin receptor
LIPC	0.69	6.42	ENSG00000166035	lipase C, hepatic type
CRYL1	0.69	4.64	ENSG00000165475	crystallin lambda 1
ORMDL2	0.69	6.24	ENSG00000123353	ORMDL sphingolipid biosynthesis regulator 2

COMMD3	0.69	4.20	ENSG00000148444	COMM domain containing 3
TMEM25	0.69	1.49	ENSG00000149582	transmembrane protein 25
SLC22A18	0.69	7.70	ENSG00000110628	solute carrier family 22 member 18
PLD1	0.69	6.36	ENSG00000075651	phospholipase D1
FLRT2	0.69	2.24	ENSG00000185070	fibronectin leucine rich transmembrane protein 2
PRRT1	0.69	2.24	ENSG00000229071	proline rich transmembrane protein 1
SGPP1	0.69	3.55	ENSG00000126821	sphingosine-1-phosphate phosphatase 1
CYB561D1	0.68	2.56	ENSG00000174151	cytochrome b561 family member D1
DHRS4	0.68	1.99	ENSG00000157326	dehydrogenase/reductase 4
HEXDC	0.68	5.04	ENSG00000169660	hexosaminidase D
FADS3	0.68	6.04	ENSG00000221968	fatty acid desaturase 3
NOMO1	0.68	6.50	ENSG00000274779	NODAL modulator 1
FDXR	0.68	4.94	ENSG00000161513	ferredoxin reductase
PLAC8	0.68	4.71	ENSG00000145287	placenta specific 8
TMED3	0.68	8.20	ENSG00000166557	transmembrane p24 trafficking protein 3
RP4-568F9.6	0.68	1.04	ENSG00000230010	N/A
MYL6B	0.68	6.73	ENSG00000196465	myosin light chain 6B
THYN1	0.68	5.65	ENSG00000151500	thymocyte nuclear protein 1
SLC25A27	0.68	2.13	ENSG00000153291	solute carrier family 25 member 27
BTN3A2	0.68	4.54	ENSG00000186470	butyrophilin subfamily 3 member A2
IMPDH1	0.68	6.42	ENSG00000106348	inosine monophosphate dehydrogenase 1
TBC1D17	0.68	7.61	ENSG00000104946	TBC1 domain family member 17
RAB3A	0.68	3.08	ENSG00000105649	RAB3A, member RAS oncogene family
TTLL7	0.68	3.08	ENSG00000137941	tubulin tyrosine ligase like 7
TPGS1	0.68	3.99	ENSG00000141933	tubulin polyglutamylase complex subunit 1
FKBP10	0.68	8.68	ENSG00000141756	FK506 binding protein 10
NBPF10	0.68	2.43	ENSG00000271425	neuroblastoma breakpoint family member 10
MLPH	0.68	7.98	ENSG00000115648	melanophilin
RPL36AL	0.68	8.17	ENSG00000165502	ribosomal protein L36a like
HFE2	0.68	1.30	ENSG00000168509	hemochromatosis type 2 (juvenile)

BICC1	0.68	3.45	ENSG00000122870	BicC family RNA binding protein 1
CHCHD1	0.68	5.97	ENSG00000172586	coiled-coil-helix-coiled-coil-helix domain containing 1
RP11-223I10.1	0.68	2.38	ENSG00000261829	N/A
RP11-49I11.1	0.67	4.15	ENSG00000260552	N/A
DSCAM-AS1	0.67	8.27	ENSG00000235123	DSCAM antisense RNA 1
NDUFA1	0.67	7.41	ENSG00000125356	NADH:ubiquinone oxidoreductase subunit A1
ATP5I	0.67	6.93	ENSG00000169020	ATP synthase, H+ transporting, mitochondrial Fo complex subunit E
CACNA1H	0.67	7.05	ENSG00000196557	calcium voltage-gated channel subunit alpha1 H
CNKSR1	0.67	1.52	ENSG00000142675	connector enhancer of kinase suppressor of Ras 1
LINC00910	0.67	1.52	ENSG00000188825	long intergenic non-protein coding RNA 910
EXOC4	0.67	5.43	ENSG00000131558	exocyst complex component 4
C11orf49	0.67	5.93	ENSG00000149179	chromosome 11 open reading frame 49
HIPK3	0.67	4.15	ENSG00000110422	homeodomain interacting protein kinase 3
LHFPL2	0.67	4.85	ENSG00000145685	lipoma HMGIC fusion partner-like 2
PLP2	0.67	8.17	ENSG00000102007	proteolipid protein 2
UCN	0.67	3.11	ENSG00000163794	urocortin
ABCA2	0.67	6.39	ENSG00000107331	ATP binding cassette subfamily A member 2
SCARB1	0.67	10.05	ENSG00000073060	scavenger receptor class B member 1
ZNF333	0.67	1.95	ENSG00000160961	zinc finger protein 333
FAM171B	0.67	3.27	ENSG00000144369	family with sequence similarity 171 member B
TMEM86B	0.67	6.16	ENSG00000180089	transmembrane protein 86B
SMPDL3B	0.67	4.38	ENSG00000130768	sphingomyelin phosphodiesterase acid like 3B
GSTM4	0.67	6.25	ENSG00000168765	glutathione S-transferase mu 4
RBPMS-AS1	0.67	5.80	ENSG00000254109	RBPMS antisense RNA 1
MUC1	0.67	2.40	ENSG00000185499	mucin 1, cell surface associated
FGFR1	0.67	5.58	ENSG00000077782	fibroblast growth factor receptor 1

COX7B	0.67	8.39	ENSG00000131174	cytochrome c oxidase subunit 7B
MSI1	0.67	4.75	ENSG00000135097	musashi RNA binding protein 1
KRCC1	0.67	2.23	ENSG00000172086	lysine rich coiled-coil 1
C9orf16	0.67	7.04	ENSG00000171159	chromosome 9 open reading frame 16
MLLT3	0.67	2.83	ENSG00000171843	MLLT3, super elongation complex subunit
CCS	0.67	6.09	ENSG00000173992	copper chaperone for superoxide dismutase
C1D	0.67	2.61	ENSG00000197223	C1D nuclear receptor corepressor
CALM2	0.67	9.43	ENSG00000143933	calmodulin 2
GPR137C	0.67	2.35	ENSG00000180998	G protein-coupled receptor 137C
WDR5B	0.67	2.35	ENSG00000196981	WD repeat domain 5B
HSPA1A	0.67	6.96	ENSG00000234475	heat shock protein family A (Hsp70) member 1A
TMEM141	0.67	8.06	ENSG00000244187	transmembrane protein 141
NLRX1	0.67	5.10	ENSG00000160703	NLR family member X1
ALKBH6	0.67	3.45	ENSG00000239382	alkB homolog 6
CTSF	0.66	5.62	ENSG00000174080	cathepsin F
NFIC	0.66	5.69	ENSG00000141905	nuclear factor I C
ANAPC16	0.66	6.23	ENSG00000166295	anaphase promoting complex subunit 16
RTN4R	0.66	3.43	ENSG00000040608	reticulon 4 receptor
PTK7	0.66	3.06	ENSG00000112655	protein tyrosine kinase 7 (inactive)
NUDT12	0.66	2.57	ENSG00000112874	nudix hydrolase 12
PSENEN	0.66	7.03	ENSG00000205155	presenilin enhancer gamma-secretase subunit
TBX6	0.66	2.30	ENSG00000149922	T-box 6
TCEB2	0.66	8.02	ENSG00000103363	transcription elongation factor B subunit 2
PFN1	0.66	10.72	ENSG00000108518	profilin 1
ABCA1	0.66	5.04	ENSG00000165029	ATP binding cassette subfamily A member 1
HHAT	0.66	1.34	ENSG00000280680	hedgehog acyltransferase
ZNF287	0.66	1.34	ENSG00000141040	zinc finger protein 287
PDLIM2	0.66	2.84	ENSG00000120913	PDZ and LIM domain 2

UQCRB	0.66	7.87	ENSG00000156467	ubiquinol-cytochrome c reductase binding protein
CYB5D2	0.66	3.23	ENSG00000167740	cytochrome b5 domain containing 2
RAB8B	0.66	4.46	ENSG00000166128	RAB8B, member RAS oncogene family
TMEM106B	0.66	4.98	ENSG00000106460	transmembrane protein 106B
TMEM30A	0.66	6.06	ENSG00000112697	transmembrane protein 30A
DYNLT3	0.66	4.93	ENSG00000165169	dynein light chain Tctex-type 3
MXD3	0.66	5.16	ENSG00000213347	MAX dimerization protein 3
IL13RA1	0.66	6.48	ENSG00000131724	interleukin 13 receptor subunit alpha 1
SIAE	0.66	4.09	ENSG00000110013	sialic acid acetylesterase
CD99	0.66	9.52	ENSG00000002586	CD99 molecule
FUCA1	0.66	5.90	ENSG00000179163	fucosidase, alpha-L- 1, tissue
GNS	0.66	7.67	ENSG00000135677	glucosamine (N-acetyl)-6-sulfatase
SPRYD3	0.66	6.52	ENSG00000167778	SPRY domain containing 3
IPP	0.66	1.24	ENSG00000197429	intracisternal A particle-promoted polypeptide
UNC13D	0.66	1.24	ENSG00000092929	unc-13 homolog D
IRF9	0.65	1.47	ENSG00000213928	interferon regulatory factor 9
EMP2	0.65	4.79	ENSG00000213853	epithelial membrane protein 2
SIRT4	0.65	2.00	ENSG00000089163	sirtuin 4
ST20	0.65	3.46	ENSG00000180953	suppressor of tumorigenicity 20
CHID1	0.65	6.60	ENSG00000177830	chitinase domain containing 1
IDH2	0.65	8.55	ENSG00000182054	isocitrate dehydrogenase (NADP(+)) 2, mitochondrial
SEMA6C	0.65	5.25	ENSG00000143434	semaphorin 6C
AMOT	0.65	3.07	ENSG00000126016	angiomotin
P4HA2	0.65	5.48	ENSG00000072682	prolyl 4-hydroxylase subunit alpha 2
NID1	0.65	8.40	ENSG00000116962	nidogen 1
PYY	0.65	1.78	ENSG00000131096	peptide YY
GABBR1	0.65	6.67	ENSG00000237112	gamma-aminobutyric acid type B receptor subunit 1
MORN2	0.65	3.95	ENSG00000188010	MORN repeat containing 2
YJEFN3	0.65	3.95	ENSG00000250067	YjeF N-terminal domain containing 3
RABAC1	0.65	8.61	ENSG00000105404	Rab acceptor 1

SDHAF2	0.65	4.69	ENSG00000167985	succinate dehydrogenase complex assembly factor 2
ABHD16B	0.65	3.34	ENSG00000183260	abhydrolase domain containing 16B
FMNL2	0.65	4.76	ENSG00000157827	formin like 2
DNAJB5	0.65	3.99	ENSG00000137094	DnaJ heat shock protein family (Hsp40) member B5
VWA5A	0.64	4.76	ENSG00000110002	von Willebrand factor A domain containing 5A
UROS	0.64	6.16	ENSG00000188690	uroporphyrinogen III synthase
PLOD1	0.64	9.37	ENSG00000083444	procollagen-lysine,2-oxoglutarate 5-dioxygenase 1
SPTLC2	0.64	4.48	ENSG00000100596	serine palmitoyltransferase long chain base subunit 2
RPS29	0.64	6.78	ENSG00000213741	ribosomal protein S29
EPDR1	0.64	3.76	ENSG00000086289	ependymin related 1
AGBL3	0.64	1.51	ENSG00000146856	ATP/GTP binding protein like 3
RASSF5	0.64	3.44	ENSG00000266094	Ras association domain family member 5
CYSTM1	0.64	7.77	ENSG00000120306	cysteine rich transmembrane module containing 1
PPP1R1A	0.64	2.24	ENSG00000135447	protein phosphatase 1 regulatory inhibitor subunit 1A
TRIOBP	0.64	4.61	ENSG00000100106	TRIO and F-actin binding protein
AIG1	0.64	6.71	ENSG00000146416	androgen induced 1
OTUB2	0.64	3.02	ENSG00000277276	OTU deubiquitinase, ubiquitin aldehyde binding 2
SLFN5	0.64	3.02	ENSG00000166750	schlafen family member 5
LRRC23	0.64	4.43	ENSG00000010626	leucine rich repeat containing 23
RASA3	0.64	1.28	ENSG00000280477	RAS p21 protein activator 3
MAGEH1	0.64	3.85	ENSG00000187601	MAGE family member H1
SH3BP1	0.64	2.86	ENSG00000100092	SH3 domain binding protein 1
CYP24A1	0.64	6.01	ENSG00000019186	cytochrome P450 family 24 subfamily A member 1
EXOC3L4	0.64	5.38	ENSG00000205436	exocyst complex component 3 like 4
CNTNAP4	0.64	2.31	ENSG00000152910	contactin associated protein like 4
PCBD1	0.64	7.81	ENSG00000166228	pterin-4 alpha-carbinolamine dehydratase 1

PODXL2	0.64	6.40	ENSG00000114631	podocalyxin like 2
SERPINA1	0.64	13.65	ENSG00000197249	serpin family A member 1
AC074212.5	0.64	2.49	ENSG00000259605	N/A
CDK5RAP3	0.64	6.58	ENSG00000108465	CDK5 regulatory subunit associated protein 3
CLUAP1	0.64	3.68	ENSG00000103351	clusterin associated protein 1
ING4	0.64	4.33	ENSG00000111653	inhibitor of growth family member 4
TMEM175	0.64	5.59	ENSG00000127419	transmembrane protein 175
RP11-295G20.2	0.64	5.44	ENSG00000233461	N/A
TP53I3	0.64	5.82	ENSG00000115129	tumor protein p53 inducible protein 3
SGSM1	0.64	2.88	ENSG00000167037	small G protein signaling modulator 1
GS1-358P8.4	0.63	4.88	ENSG00000260822	N/A
CYB5RL	0.63	1.42	ENSG00000215883	cytochrome b5 reductase like
OSBPL10	0.63	1.90	ENSG00000144645	oxysterol binding protein like 10
PROX1-AS1	0.63	2.26	ENSG00000272167	N/A
LRRC27	0.63	3.34	ENSG00000148814	leucine rich repeat containing 27
TCIRG1	0.63	7.51	ENSG00000110719	T-cell immune regulator 1, ATPase H+ transporting V0 subunit a3
SPAG4	0.63	4.03	ENSG00000061656	sperm associated antigen 4
AMN	0.63	6.08	ENSG00000166126	amnion associated transmembrane protein
PMF1	0.63	5.96	ENSG00000160783	polyamine modulated factor 1
PLSCR1	0.63	2.84	ENSG00000188313	phospholipid scramblase 1
MSTO2P	0.63	1.73	ENSG00000203761	misato family member 2, pseudogene
ZNF514	0.63	1.73	ENSG00000144026	zinc finger protein 514
NSMCE1	0.63	6.29	ENSG00000169189	NSE1 homolog, SMC5-SMC6 complex component
BSG	0.63	10.79	ENSG00000172270	basigin (Ok blood group)
MRPL17	0.63	6.93	ENSG00000158042	mitochondrial ribosomal protein L17
DGCR5	0.63	3.74	ENSG00000237517	DiGeorge syndrome critical region gene 5 (non-protein coding)
OR7E38P	0.63	3.74	ENSG00000183444	olfactory receptor family 7 subfamily E member 38 pseudogene
MPZL2	0.63	4.98	ENSG00000149573	myelin protein zero like 2
ATP11C	0.63	3.73	ENSG00000101974	ATPase phospholipid transporting 11C

CYBA	0.63	10.08	ENSG00000051523	cytochrome b-245 alpha chain
SPPL2B	0.63	6.26	ENSG00000005206	signal peptide peptidase like 2B
C1R	0.63	2.21	ENSG00000159403	complement C1r
EDARADD	0.63	2.21	ENSG00000186197	EDAR associated death domain
CCDC96	0.63	1.54	ENSG00000173013	coiled-coil domain containing 96
CTC-308K20.1	0.63	1.54	ENSG00000204758	N/A
METRNL	0.63	3.12	ENSG00000275031	meteorin like, glial cell differentiation regulator
ZNF165	0.63	2.40	ENSG00000197279	zinc finger protein 165
ACOT13	0.63	6.13	ENSG00000112304	acyl-CoA thioesterase 13
CEP57L1	0.63	3.26	ENSG00000183137	centrosomal protein 57 like 1
JAM3	0.63	2.08	ENSG00000166086	junctional adhesion molecule 3
SHKBP1	0.63	8.22	ENSG00000160410	SH3KBP1 binding protein 1
PDGFC	0.63	3.20	ENSG00000145431	platelet derived growth factor C
ITGB1BP2	0.63	1.32	ENSG00000147166	integrin subunit beta 1 binding protein 2
PDGFD	0.63	1.32	ENSG00000170962	platelet derived growth factor D
TNRC6C-AS1	0.63	1.32	ENSG00000204282	TNRC6C antisense RNA 1
GABARAPL2	0.63	5.46	ENSG00000034713	GABA type A receptor associated protein like 2
PMVK	0.63	6.39	ENSG00000163344	phosphomevalonate kinase
CKAP4	0.63	8.61	ENSG00000136026	cytoskeleton associated protein 4
ELOVL4	0.63	1.93	ENSG00000118402	ELOVL fatty acid elongase 4
ARRDC3	0.63	2.35	ENSG00000113369	arrestin domain containing 3
FAM86JP	0.63	2.35	ENSG00000171084	family with sequence similarity 86 member J, pseudogene
BAZ2B	0.63	2.68	ENSG00000123636	bromodomain adjacent to zinc finger domain 2B
ANKRD42	0.62	2.82	ENSG00000137494	ankyrin repeat domain 42
NPAS2	0.62	4.13	ENSG00000170485	neuronal PAS domain protein 2
C14orf166	0.62	7.11	ENSG00000087302	chromosome 14 open reading frame 166
FLOT2	0.62	8.01	ENSG00000132589	flotillin 2
RBPJ	0.62	5.32	ENSG00000168214	recombination signal binding protein for immunoglobulin kappa J region
D2HGDH	0.62	3.60	ENSG00000180902	D-2-hydroxyglutarate dehydrogenase
ENO4	0.62	1.76	ENSG00000188316	enolase family member 4

NIPAL3	0.62	3.25	ENSG00000001461	NIPA like domain containing 3
ECHDC2	0.62	5.94	ENSG00000121310	enoyl-CoA hydratase domain containing 2
PEX11A	0.62	3.15	ENSG00000166821	peroxisomal biogenesis factor 11 alpha
CHD6	0.62	4.26	ENSG00000124177	chromodomain helicase DNA binding protein 6
AMT	0.62	1.45	ENSG00000145020	aminomethyltransferase
COQ7	0.62	2.92	ENSG00000167186	coenzyme Q7, hydroxylase
RP11-403I13.8	0.62	2.92	ENSG00000272419	N/A
KLRC2	0.62	1.06	ENSG00000205809	killer cell lectin like receptor C2
PSPN	0.62	2.48	ENSG00000125650	persephin
KPNA5	0.62	3.08	ENSG00000196911	karyopherin subunit alpha 5
CAMK2N1	0.62	7.01	ENSG00000162545	calcium/calmodulin dependent protein kinase II inhibitor 1
HID1	0.62	7.41	ENSG00000167861	HID1 domain containing
EFHC1	0.62	3.97	ENSG00000096093	EF-hand domain containing 1
HLA-L	0.62	2.84	ENSG00000243753	major histocompatibility complex, class I, L (pseudogene)
CORO1A	0.62	2.10	ENSG00000102879	coronin 1A
MAPKAPK5-AS1	0.62	5.34	ENSG00000234608	MAPKAPK5 antisense RNA 1
MID1	0.62	7.91	ENSG00000101871	midline 1
HOXA2	0.62	1.86	ENSG00000105996	homeobox A2
GSTM3	0.62	6.92	ENSG00000134202	glutathione S-transferase mu 3
PPP1R14BP3	0.62	3.48	ENSG00000179967	protein phosphatase 1 regulatory inhibitor subunit 14B pseudogene 3
EAPP	0.62	4.52	ENSG00000129518	E2F associated phosphoprotein
GOLGA8A	0.62	3.16	ENSG00000175265	golgin A8 family member A
FHL2	0.62	1.57	ENSG00000115641	four and a half LIM domains 2
TCP11L1	0.62	3.30	ENSG00000176148	t-complex 11 like 1
PPP3CB	0.62	4.19	ENSG00000107758	protein phosphatase 3 catalytic subunit beta
PRSS12	0.62	4.49	ENSG00000164099	protease, serine 12
KDELR3	0.62	3.96	ENSG00000100196	KDEL endoplasmic reticulum protein retention receptor 3
PNRC2	0.62	1.22	ENSG00000189266	proline rich nuclear receptor coactivator 2

RP11-108M9.4	0.62	1.22	ENSG00000238142	N/A
TMEM45A	0.62	4.40	ENSG00000181458	transmembrane protein 45A
PLEKHA4	0.62	4.29	ENSG00000105559	pleckstrin homology domain containing A4
VPS37D	0.62	2.97	ENSG00000176428	VPS37D, ESCRT-I subunit
YIPF4	0.62	4.90	ENSG00000119820	Yip1 domain family member 4
SLC25A14	0.62	4.28	ENSG00000102078	solute carrier family 25 member 14
PTRHD1	0.62	5.65	ENSG00000184924	peptidyl-tRNA hydrolase domain containing 1
CTC-548K16.1	0.62	1.69	ENSG00000267147	N/A
ALG3	0.62	9.09	ENSG00000214160	ALG3, alpha-1,3-mannosyltransferase
LRP1	0.62	8.37	ENSG00000123384	LDL receptor related protein 1
ZNF487	0.61	2.04	ENSG00000243660	zinc finger protein 487
PLXNA3	0.61	4.84	ENSG00000130827	plexin A3
C14orf105	0.61	4.28	ENSG00000100557	chromosome 14 open reading frame 105
SHF	0.61	4.35	ENSG00000138606	Src homology 2 domain containing F
LUCAT1	0.61	2.76	ENSG00000248323	lung cancer associated transcript 1 (non-protein coding)
PFDN4	0.61	5.79	ENSG00000101132	prefoldin subunit 4
SH3BGR	0.61	1.36	ENSG00000185437	SH3 domain binding glutamate rich protein
MNS1	0.61	2.39	ENSG00000138587	meiosis specific nuclear structural 1
ZNF91	0.61	2.81	ENSG00000167232	zinc finger protein 91
MAGEA10	0.61	3.14	ENSG00000124260	MAGE family member A10
VASH1	0.61	3.41	ENSG00000071246	vasohibin 1
TLE6	0.61	4.97	ENSG00000104953	transducin like enhancer of split 6
RAB31	0.61	6.12	ENSG00000168461	RAB31, member RAS oncogene family
CCDC71L	0.61	3.03	ENSG00000253276	coiled-coil domain containing 71-like
CCDC134	0.61	2.67	ENSG00000100147	coiled-coil domain containing 134
AGTR1	0.61	5.13	ENSG00000144891	angiotensin II receptor type 1
NMB	0.61	3.90	ENSG00000197696	neuromedin B
MUC2	0.61	2.91	ENSG00000278466	mucin 2, oligomeric mucus/gel-forming

LTBP4	0.61	7.87	ENSG00000090006	latent transforming growth factor beta binding protein 4
ALPK1	0.61	1.49	ENSG00000073331	alpha kinase 1
RP13-890H12.2	0.61	1.49	ENSG00000267288	N/A
CERS5	0.61	5.90	ENSG00000139624	ceramide synthase 5
TM7SF2	0.61	7.92	ENSG00000149809	transmembrane 7 superfamily member 2
CD99L2	0.61	5.91	ENSG00000102181	CD99 molecule like 2
SERPINH1	0.61	5.45	ENSG00000149257	serpin family H member 1
ATRAID	0.61	8.17	ENSG00000138085	all-trans retinoic acid induced differentiation factor
POLD4	0.61	4.90	ENSG00000175482	DNA polymerase delta 4, accessory subunit
C16orf74	0.61	1.61	ENSG00000154102	chromosome 16 open reading frame 74
CFI	0.61	3.97	ENSG00000205403	complement factor I
OXLD1	0.61	5.98	ENSG00000204237	oxidoreductase like domain containing 1
OXTR	0.61	1.11	ENSG00000180914	oxytocin receptor
RPS6KA6	0.61	1.11	ENSG00000072133	ribosomal protein S6 kinase A6
BRE	0.61	6.41	ENSG00000158019	brain and reproductive organ-expressed (TNFRSF1A modulator)
PLEKHN1	0.61	1.72	ENSG00000187583	pleckstrin homology domain containing N1
TSPAN33	0.61	5.30	ENSG00000158457	tetraspanin 33
SNTA1	0.61	6.84	ENSG00000101400	syntrophin alpha 1
FAM210B	0.61	5.90	ENSG00000124098	family with sequence similarity 210 member B
SBK1	0.60	3.01	ENSG00000188322	SH3 domain binding kinase 1
RHBDD2	0.60	7.16	ENSG00000005486	rhomboid domain containing 2
CELSR2	0.60	4.91	ENSG00000143126	cadherin EGF LAG seven-pass G-type receptor 2
KRBOX1	0.60	1.26	ENSG00000240747	KRAB box domain containing 1
SNAI3	0.60	1.26	ENSG00000185669	snail family transcriptional repressor 3
TP73	0.60	1.26	ENSG00000078900	tumor protein p73
CASP4	0.60	4.45	ENSG00000196954	caspase 4

TFPI2	0.60	1.91	ENSG00000105825	tissue factor pathway inhibitor 2
HOMER3	0.60	3.95	ENSG00000051128	homer scaffolding protein 3
MPND	0.60	4.84	ENSG00000008382	MPN domain containing
HDDC2	0.60	6.51	ENSG00000111906	HD domain containing 2
ARHGAP42	0.60	2.00	ENSG00000165895	Rho GTPase activating protein 42
TMCC1-AS1	0.60	2.00	ENSG00000271270	TMCC1 antisense RNA 1 (head to head)
MPP7	0.60	1.40	ENSG00000150054	membrane palmitoylated protein 7
ARHGEF10	0.60	2.81	ENSG00000274726	Rho guanine nucleotide exchange factor 10
ELF2	-0.60	2.89	ENSG00000109381	E74 like ETS transcription factor 2
BOD1	-0.60	5.93	ENSG00000145919	biorientation of chromosomes in cell division 1
HNRNPH1	-0.60	7.75	ENSG00000169045	heterogeneous nuclear ribonucleoprotein H1 (H)
NAMPT	-0.60	4.02	ENSG00000105835	nicotinamide phosphoribosyltransferase
HIRA	-0.60	3.85	ENSG00000100084	histone cell cycle regulator
RAD51D	-0.60	3.16	ENSG00000185379	RAD51 paralog D
METTL21A	-0.60	4.70	ENSG00000144401	methyltransferase like 21A
ZMYM5	-0.60	1.38	ENSG00000132950	zinc finger MYM-type containing 5
ZNF891	-0.60	1.38	ENSG00000214029	zinc finger protein 891
SBDS	-0.60	4.74	ENSG00000126524	SBDS ribosome assembly guanine nucleotide exchange factor
USP34	-0.60	4.62	ENSG00000115464	ubiquitin specific peptidase 34
NUDT15	-0.60	4.06	ENSG00000136159	nudix hydrolase 15
SKI	-0.60	5.43	ENSG00000157933	SKI proto-oncogene
CAD	-0.60	7.71	ENSG00000084774	carbamoyl-phosphate synthetase 2, aspartate transcarbamylase, and dihydroorotase
SMARCD1	-0.60	7.43	ENSG00000066117	SWI/SNF related, matrix associated, actin dependent regulator of chromatin, subfamily d, member 1
ADCY3	-0.60	3.95	ENSG00000138031	adenylate cyclase 3
PHF12	-0.60	5.84	ENSG00000109118	PHD finger protein 12
C21orf91	-0.61	2.98	ENSG00000154642	chromosome 21 open reading frame 91

PRR22	-0.61	4.04	ENSG00000212123	proline rich 22
DCLRE1A	-0.61	3.45	ENSG00000198924	DNA cross-link repair 1A
PDE4D	-0.61	4.74	ENSG00000113448	phosphodiesterase 4D
CHML	-0.61	4.18	ENSG00000203668	CHM like, Rab escort protein 2
ANKRD17	-0.61	5.99	ENSG00000132466	ankyrin repeat domain 17
KIF23	-0.61	5.66	ENSG00000137807	kinesin family member 23
DPH2	-0.61	6.10	ENSG00000132768	DPH2 homolog
USP19	-0.61	5.96	ENSG00000172046	ubiquitin specific peptidase 19
DBF4B	-0.61	5.91	ENSG00000161692	DBF4 zinc finger B
TMEM183A	-0.61	3.80	ENSG00000163444	transmembrane protein 183A
WDR55	-0.61	5.92	ENSG00000120314	WD repeat domain 55
MAGEA4	-0.61	6.90	ENSG00000147381	MAGE family member A4
CDK5R1	-0.61	1.85	ENSG00000176749	cyclin dependent kinase 5 regulatory subunit 1
ADM5	-0.61	2.68	ENSG00000224420	adrenomedullin 5 (putative)
RRP15	-0.61	4.83	ENSG00000067533	ribosomal RNA processing 15 homolog
KLF16	-0.61	6.62	ENSG00000129911	Kruppel like factor 16
IRS1	-0.61	4.53	ENSG00000169047	insulin receptor substrate 1
NOP58	-0.61	6.93	ENSG00000055044	NOP58 ribonucleoprotein
RP11-364L4.1	-0.61	1.04	ENSG00000228981	N/A
RP11-488C13.5	-0.61	1.04	ENSG00000258301	N/A
MAPK1IP1L	-0.61	6.49	ENSG00000168175	mitogen-activated protein kinase 1 interacting protein 1 like
TSPAN12	-0.61	2.62	ENSG00000106025	tetraspanin 12
DGCR8	-0.61	5.31	ENSG00000128191	DGCR8, microprocessor complex subunit
LSS	-0.61	7.27	ENSG00000281289	lanosterol synthase (2,3-oxidosqualene-lanosterol cyclase)
NCL	-0.61	9.17	ENSG00000115053	nucleolin
FAM189B	-0.61	7.53	ENSG00000160767	family with sequence similarity 189 member B
TOR4A	-0.61	3.49	ENSG00000198113	torsin family 4 member A
MED30	-0.61	2.21	ENSG00000164758	mediator complex subunit 30
EPB41L4A-AS1	-0.61	3.08	ENSG00000224032	EPB41L4A antisense RNA 1
WDR35	-0.61	3.08	ENSG00000118965	WD repeat domain 35

ZBTB37	-0.61	3.08	ENSG00000185278	zinc finger and BTB domain containing 37
DAGLA	-0.61	3.18	ENSG00000134780	diacylglycerol lipase alpha
ANAPC1	-0.61	3.76	ENSG00000153107	anaphase promoting complex subunit 1
GATA3	-0.61	4.06	ENSG00000107485	GATA binding protein 3
POLD1	-0.61	6.81	ENSG00000062822	DNA polymerase delta 1, catalytic subunit
MED14	-0.61	5.57	ENSG00000180182	mediator complex subunit 14
PCGF6	-0.61	2.65	ENSG00000156374	polycomb group ring finger 6
CCNK	-0.61	6.36	ENSG00000090061	cyclin K
GPC3	-0.61	8.80	ENSG00000147257	glypican 3
CEP97	-0.61	1.89	ENSG00000182504	centrosomal protein 97
RHNO1	-0.62	5.43	ENSG00000171792	RAD9-HUS1-RAD1 interacting nuclear orphan 1
CASP3	-0.62	2.73	ENSG00000164305	caspase 3
NOP14	-0.62	6.17	ENSG00000087269	NOP14 nucleolar protein
ETV3	-0.62	3.56	ENSG00000117036	ETS variant 3
CLK4	-0.62	3.05	ENSG00000113240	CDC like kinase 4
ABCA8	-0.62	4.14	ENSG00000141338	ATP binding cassette subfamily A member 8
RC3H1	-0.62	4.04	ENSG00000135870	ring finger and CCCH-type domains 1
POLR2A	-0.62	7.69	ENSG00000181222	RNA polymerase II subunit A
DDX28	-0.62	3.79	ENSG00000182810	DEAD-box helicase 28
PITHD1	-0.62	5.36	ENSG00000057757	PITH domain containing 1
SLC26A1	-0.62	5.28	ENSG00000145217	solute carrier family 26 member 1
TIMM44	-0.62	6.26	ENSG00000104980	translocase of inner mitochondrial membrane 44
IFRD1	-0.62	5.96	ENSG00000006652	interferon related developmental regulator 1
GATC	-0.62	5.04	ENSG00000257218	glutamyl-tRNA amidotransferase subunit C
DDX31	-0.62	4.71	ENSG00000125485	DEAD-box helicase 31
OTUD4	-0.62	3.54	ENSG00000164164	OTU deubiquitinase 4
TRIM4	-0.62	4.31	ENSG00000146833	tripartite motif containing 4
ZNF583	-0.62	2.15	ENSG00000198440	zinc finger protein 583

CENPF	-0.62	5.96	ENSG00000117724	centromere protein F
GNL3L	-0.62	2.61	ENSG00000130119	G protein nucleolar 3 like
SHC1	-0.62	8.49	ENSG00000160691	SHC adaptor protein 1
PNMA1	-0.62	4.34	ENSG00000176903	paraneoplastic Ma antigen 1
E2F5	-0.62	4.71	ENSG00000133740	E2F transcription factor 5
ARMCX5	-0.62	3.38	ENSG00000125962	armadillo repeat containing, X-linked 5
ASNSD1	-0.62	5.12	ENSG00000138381	asparagine synthetase domain containing 1
MGA	-0.62	4.25	ENSG00000174197	MGA, MAX dimerization protein
UBE3D	-0.62	3.19	ENSG00000118420	ubiquitin protein ligase E3D
L3HYPDH	-0.62	2.27	ENSG00000126790	trans-L-3-hydroxyproline dehydratase
ABL1	-0.62	5.86	ENSG00000097007	ABL proto-oncogene 1, non-receptor tyrosine kinase
KIAA1549	-0.62	5.16	ENSG00000122778	KIAA1549
MAK16	-0.62	4.78	ENSG00000198042	MAK16 homolog
CCDC138	-0.62	3.04	ENSG00000163006	coiled-coil domain containing 138
DHX30	-0.62	6.74	ENSG00000132153	DExH-box helicase 30
HMGN2	-0.62	5.15	ENSG00000198830	high mobility group nucleosomal binding domain 2
SLC7A6OS	-0.62	2.18	ENSG00000103061	solute carrier family 7 member 6 opposite strand
TEF	-0.62	1.82	ENSG00000167074	TEF, PAR bZIP transcription factor
MAGEA6	-0.63	5.70	ENSG00000197172	MAGE family member A6
CCNB1	-0.63	7.24	ENSG00000134057	cyclin B1
UBTF	-0.63	7.39	ENSG00000108312	upstream binding transcription factor, RNA polymerase I
MED12	-0.63	6.54	ENSG00000184634	mediator complex subunit 12
TRPM7	-0.63	4.54	ENSG00000092439	transient receptor potential cation channel subfamily M member 7
CTCF	-0.63	5.19	ENSG00000102974	CCCTC-binding factor
ARL6IP6	-0.63	3.47	ENSG00000177917	ADP ribosylation factor like GTPase 6 interacting protein 6
DDX19A	-0.63	5.76	ENSG00000168872	DEAD-box helicase 19A
CDKN2B	-0.63	1.54	ENSG00000147883	cyclin dependent kinase inhibitor 2B
NOMO2	-0.63	3.56	ENSG00000185164	NODAL modulator 2

WDR45B	-0.63	7.49	ENSG00000141580	WD repeat domain 45B
INTS9	-0.63	3.86	ENSG00000104299	integrator complex subunit 9
WDR90	-0.63	4.40	ENSG00000161996	WD repeat domain 90
ZNF131	-0.63	3.76	ENSG00000172262	zinc finger protein 131
MESDC1	-0.63	6.26	ENSG00000140406	mesoderm development candidate 1
FAN1	-0.63	2.89	ENSG00000276787	FANCD2/FANCI-associated nuclease 1
SFSWAP	-0.63	5.94	ENSG00000061936	splicing factor SWAP homolog
FKBP11	-0.63	5.98	ENSG00000134285	FK506 binding protein 11
CHCHD2P2	-0.63	2.33	ENSG00000275346	coiled-coil-helix-coiled-coil-helix domain containing 2 pseudogene 2
SART3	-0.63	6.07	ENSG00000075856	squamous cell carcinoma antigen recognized by T-cells 3
FAM103A1	-0.63	2.76	ENSG00000169612	family with sequence similarity 103 member A1
NFX1	-0.63	5.15	ENSG00000086102	nuclear transcription factor, X-box binding 1
PTBP1	-0.63	8.96	ENSG00000011304	polypyrimidine tract binding protein 1
SPRED2	-0.63	5.58	ENSG00000198369	sprouty related EVH1 domain containing 2
SMAD1	-0.63	1.39	ENSG00000170365	SMAD family member 1
SORD	-0.63	3.64	ENSG00000140263	sorbitol dehydrogenase
R3HDM1	-0.63	6.16	ENSG00000048991	R3H domain containing 1
DOPEY1	-0.63	2.46	ENSG00000083097	dopey family member 1
SNX27	-0.63	5.90	ENSG00000143376	sorting nexin family member 27
INCENP	-0.64	5.32	ENSG00000149503	inner centromere protein
PURA	-0.64	2.72	ENSG00000185129	purine rich element binding protein A
TGS1	-0.64	4.73	ENSG00000137574	trimethylguanosine synthase 1
RELL2	-0.64	4.91	ENSG00000164620	RELT like 2
STAM	-0.64	4.46	ENSG00000136738	signal transducing adaptor molecule
IRAK1	-0.64	8.60	ENSG00000184216	interleukin 1 receptor associated kinase 1
AGO2	-0.64	5.09	ENSG00000123908	argonaute 2, RISC catalytic component
EXO1	-0.64	4.93	ENSG00000174371	exonuclease 1
LRRC14	-0.64	5.00	ENSG00000160959	leucine rich repeat containing 14

ZIC5	-0.64	4.82	ENSG00000139800	Zic family member 5
C12orf73	-0.64	3.69	ENSG00000204954	chromosome 12 open reading frame 73
ARHGEF11	-0.64	6.33	ENSG00000132694	Rho guanine nucleotide exchange factor 11
USP36	-0.64	6.15	ENSG00000055483	ubiquitin specific peptidase 36
RBMXL1	-0.64	4.28	ENSG00000213516	RNA binding motif protein, X-linked like 1
HS6ST1	-0.64	3.84	ENSG00000136720	heparan sulfate 6-O-sulfotransferase 1
PATZ1	-0.64	5.16	ENSG00000100105	POZ/BTB and AT hook containing zinc finger 1
RP11-116D2.1	-0.64	4.48	ENSG00000261012	N/A
IDH3A	-0.64	3.98	ENSG00000166411	isocitrate dehydrogenase 3 (NAD(+)) alpha
RNF34	-0.64	5.65	ENSG00000170633	ring finger protein 34
VHL	-0.64	4.05	ENSG00000134086	von Hippel-Lindau tumor suppressor
U2AF2	-0.64	8.27	ENSG00000063244	U2 small nuclear RNA auxiliary factor 2
ABHD16A	-0.64	1.45	ENSG00000230475	abhydrolase domain containing 16A
GIT2	-0.64	4.03	ENSG00000139436	GIT ArfGAP 2
ZXDC	-0.64	4.12	ENSG00000070476	ZXD family zinc finger C
HNF1B	-0.64	6.06	ENSG00000275410	HNF1 homeobox B
ZNF263	-0.64	5.30	ENSG00000006194	zinc finger protein 263
PGS1	-0.64	4.52	ENSG00000087157	phosphatidylglycerophosphate synthase 1
FBXO31	-0.64	5.60	ENSG00000283277	F-box protein 31
DOHH	-0.64	4.81	ENSG00000129932	deoxyhypusine hydroxylase/monooxygenase
IGSF3	-0.64	5.19	ENSG00000143061	immunoglobulin superfamily member 3
RNF31	-0.64	4.25	ENSG00000092098	ring finger protein 31
CTSV	-0.64	4.55	ENSG00000136943	cathepsin V
MPC1	-0.64	5.43	ENSG00000060762	mitochondrial pyruvate carrier 1
MAP2K3	-0.64	6.79	ENSG00000034152	mitogen-activated protein kinase kinase 3
AMER1	-0.64	3.76	ENSG00000184675	APC membrane recruitment protein 1

SBNO1	-0.64	4.69	ENSG00000139697	strawberry notch homolog 1
KDM2B	-0.64	5.10	ENSG00000089094	lysine demethylase 2B
PIK3R1	-0.65	2.34	ENSG00000145675	phosphoinositide-3-kinase regulatory subunit 1
RP11-196G18.22	-0.65	2.11	ENSG00000261716	N/A
NAT10	-0.65	6.33	ENSG00000135372	N-acetyltransferase 10
GRWD1	-0.65	6.78	ENSG00000105447	glutamate rich WD repeat containing 1
POU4F1	-0.65	1.84	ENSG00000152192	POU class 4 homeobox 1
ESCO2	-0.65	2.95	ENSG00000171320	establishment of sister chromatid cohesion N-acetyltransferase 2
AC004980.7	-0.65	1.68	ENSG00000205485	N/A
LINC00641	-0.65	1.68	ENSG00000258441	long intergenic non-protein coding RNA 641
ATXN2L	-0.65	8.34	ENSG00000168488	ataxin 2 like
WDR3	-0.65	5.03	ENSG00000065183	WD repeat domain 3
CSTF2T	-0.65	4.37	ENSG00000177613	cleavage stimulation factor subunit 2 tau variant
HHEX	-0.65	5.70	ENSG00000152804	hematopoietically expressed homeobox
POLG	-0.65	5.15	ENSG00000140521	DNA polymerase gamma, catalytic subunit
SORBS2	-0.65	1.30	ENSG00000154556	sorbin and SH3 domain containing 2
ZNF229	-0.65	1.30	ENSG00000278318	zinc finger protein 229
TOM1L1	-0.65	5.06	ENSG00000141198	target of myb1 like 1 membrane trafficking protein
RP13-270P17.2	-0.65	2.36	ENSG00000265399	N/A
PCDH9	-0.65	2.14	ENSG00000184226	protocadherin 9
SPTLC1	-0.65	4.16	ENSG00000090054	serine palmitoyltransferase long chain base subunit 1
PRMT6	-0.65	4.64	ENSG00000198890	protein arginine methyltransferase 6
PKMYT1	-0.65	5.59	ENSG00000127564	protein kinase, membrane associated tyrosine/threonine 1
RBM38	-0.65	5.61	ENSG00000132819	RNA binding motif protein 38
RNF123	-0.65	4.56	ENSG00000164068	ring finger protein 123
UPF2	-0.65	5.17	ENSG00000151461	UPF2 regulator of nonsense transcripts homolog (yeast)

RPRD2	-0.65	5.41	ENSG00000163125	regulation of nuclear pre-mRNA domain containing 2
NOL6	-0.65	7.06	ENSG00000165271	nucleolar protein 6
ST3GAL4	-0.65	6.37	ENSG00000110080	ST3 beta-galactoside alpha-2,3-sialyltransferase 4
ELMSAN1	-0.65	3.59	ENSG00000156030	ELM2 and Myb/SANT domain containing 1
HAUS6	-0.66	3.15	ENSG00000147874	HAUS augmin like complex subunit 6
FBXO43	-0.66	2.05	ENSG00000156509	F-box protein 43
TOE1	-0.66	4.32	ENSG00000132773	target of EGR1, member 1 (nuclear)
PEX12	-0.66	2.79	ENSG00000108733	peroxisomal biogenesis factor 12
DDX55	-0.66	5.07	ENSG00000111364	DEAD-box helicase 55
NDRG2	-0.66	5.32	ENSG00000165795	NDRG family member 2
OTUD1	-0.66	2.32	ENSG00000165312	OTU deubiquitinase 1
ATP11A	-0.66	5.02	ENSG00000068650	ATPase phospholipid transporting 11A
RABGEF1	-0.66	1.77	ENSG00000154710	RAB guanine nucleotide exchange factor 1
ACTRT3	-0.66	1.13	ENSG00000184378	actin related protein T3
RFC5	-0.66	5.21	ENSG00000111445	replication factor C subunit 5
MYO18A	-0.66	6.36	ENSG00000196535	myosin XVIIIA
MAFG	-0.66	5.69	ENSG00000197063	MAF bZIP transcription factor G
HNRNPU	-0.66	8.38	ENSG00000153187	heterogeneous nuclear ribonucleoprotein U
AASDHPPT	-0.66	4.61	ENSG00000149313	aminoadipate-semialdehyde dehydrogenase-phosphopantetheinyl transferase
PDS5B	-0.66	4.14	ENSG00000083642	PDS5 cohesin associated factor B
EIF4G1	-0.66	9.42	ENSG00000114867	eukaryotic translation initiation factor 4 gamma 1
ZNF180	-0.66	2.08	ENSG00000167384	zinc finger protein 180
MCM4	-0.66	6.50	ENSG00000104738	minichromosome maintenance complex component 4
FUS	-0.66	7.73	ENSG00000089280	FUS RNA binding protein
CHDH	-0.66	3.73	ENSG00000016391	choline dehydrogenase
ZBTB1	-0.66	2.57	ENSG00000126804	zinc finger and BTB domain containing 1

ABCC2	-0.66	8.06	ENSG00000023839	ATP binding cassette subfamily C member 2
CDK14	-0.66	3.60	ENSG00000058091	cyclin dependent kinase 14
BRD1	-0.66	3.21	ENSG00000100425	bromodomain containing 1
CLP1	-0.66	4.35	ENSG00000172409	cleavage and polyadenylation factor I subunit 1
KLF5	-0.66	4.87	ENSG00000102554	Kruppel like factor 5
SHROOM1	-0.66	4.93	ENSG00000164403	shroom family member 1
ATF4	-0.66	8.66	ENSG00000128272	activating transcription factor 4
UBN1	-0.67	5.58	ENSG00000118900	ubinuclein 1
PI4KAP2	-0.67	2.50	ENSG00000183506	phosphatidylinositol 4-kinase alpha pseudogene 2
QSOX2	-0.67	5.61	ENSG00000165661	quiescin sulfhydryl oxidase 2
CARD9	-0.67	1.20	ENSG00000187796	caspase recruitment domain family member 9
CTD-2201E18.3	-0.67	1.20	ENSG00000177738	N/A
MBD1	-0.67	5.48	ENSG00000141644	methyl-CpG binding domain protein 1
BRCC3	-0.67	4.10	ENSG00000185515	BRCA1/BRCA2-containing complex subunit 3
RNF24	-0.67	4.20	ENSG00000101236	ring finger protein 24
SALL4	-0.67	3.99	ENSG00000101115	spalt like transcription factor 4
RPS2P46	-0.67	2.15	ENSG00000189343	ribosomal protein S2 pseudogene 46
PDXDC2P	-0.67	1.85	ENSG00000196696	pyridoxal dependent decarboxylase domain containing 2, pseudogene
STARD7	-0.67	7.15	ENSG00000084090	StAR related lipid transfer domain containing 7
MAU2	-0.67	7.10	ENSG00000129933	MAU2 sister chromatid cohesion factor
UPF1	-0.67	7.63	ENSG00000005007	UPF1, RNA helicase and ATPase
AIM1	-0.67	1.47	ENSG00000112297	absent in melanoma 1
MANEAL	-0.67	5.28	ENSG00000185090	mannosidase endo-alpha like
MTDH	-0.67	6.35	ENSG00000147649	metadherin
MAGEA12	-0.67	6.29	ENSG00000213401	MAGE family member A12
PCBP1	-0.67	7.87	ENSG00000169564	poly(rC) binding protein 1
VAV2	-0.67	5.39	ENSG00000160293	vav guanine nucleotide exchange factor 2

PABPC1	-0.67	9.23	ENSG00000070756	poly(A) binding protein cytoplasmic 1
ACBD5	-0.67	5.29	ENSG00000107897	acyl-CoA binding domain containing 5
SUGP2	-0.67	6.53	ENSG00000064607	SURP and G-patch domain containing 2
CCDC97	-0.67	6.20	ENSG00000142039	coiled-coil domain containing 97
RNF44	-0.67	6.58	ENSG00000146083	ring finger protein 44
SLPI	-0.67	5.61	ENSG00000124107	secretory leukocyte peptidase inhibitor
CPT2	-0.67	3.65	ENSG00000157184	carnitine palmitoyltransferase 2
SMAP1	-0.67	2.95	ENSG00000112305	small ArfGAP 1
RBM15B	-0.67	6.46	ENSG00000259956	RNA binding motif protein 15B
NOTCH1	-0.68	3.90	ENSG00000148400	notch 1
ELK1	-0.68	6.40	ENSG00000126767	ELK1, ETS transcription factor
MYO19	-0.68	6.86	ENSG00000278259	myosin XIX
EEF1DP1	-0.68	1.74	ENSG00000228887	eukaryotic translation elongation factor 1 delta pseudogene 1
PLK1	-0.68	7.16	ENSG00000166851	polo like kinase 1
WDR37	-0.68	3.40	ENSG00000047056	WD repeat domain 37
CCZ1	-0.68	3.13	ENSG00000122674	CCZ1 homolog, vacuolar protein trafficking and biogenesis associated
CCNE1	-0.68	4.99	ENSG00000105173	cyclin E1
DHX35	-0.68	3.88	ENSG00000101452	DEAH-box helicase 35
DYNC1H1	-0.68	8.24	ENSG00000197102	dynein cytoplasmic 1 heavy chain 1
PDHX	-0.68	5.27	ENSG00000110435	pyruvate dehydrogenase complex component X
ESRRA	-0.68	5.92	ENSG00000173153	estrogen related receptor alpha
HMGN5	-0.68	3.81	ENSG00000198157	high mobility group nucleosome binding domain 5
USP42	-0.68	4.03	ENSG00000106346	ubiquitin specific peptidase 42
NEFH	-0.68	4.71	ENSG00000100285	neurofilament heavy polypeptide
KAT5	-0.68	4.66	ENSG00000172977	lysine acetyltransferase 5
STIL	-0.68	3.32	ENSG00000123473	SCL/TAL1 interrupting locus
ZNF544	-0.68	3.32	ENSG00000198131	zinc finger protein 544
ERCC3	-0.68	6.01	ENSG00000163161	ERCC excision repair 3, TFIIH core complex helicase subunit

ADH4	-0.68	2.67	ENSG00000198099	alcohol dehydrogenase 4 (class II), pi polypeptide
PPRC1	-0.68	7.20	ENSG00000148840	peroxisome proliferator-activated receptor gamma, coactivator-related 1
NOL8	-0.69	4.64	ENSG00000198000	nucleolar protein 8
AUNIP	-0.69	3.48	ENSG00000127423	aurora kinase A and ninein interacting protein
ACVR1B	-0.69	5.99	ENSG00000135503	activin A receptor type 1B
CLUH	-0.69	8.10	ENSG00000132361	clustered mitochondria homolog
SLC7A5	-0.69	8.75	ENSG00000103257	solute carrier family 7 member 5
FTLP2	-0.69	2.69	ENSG00000232368	ferritin light chain pseudogene 2
PPIP5K2	-0.69	3.62	ENSG00000145725	diphosphoinositol pentakisphosphate kinase 2
CPEB1	-0.69	1.09	ENSG00000277445	cytoplasmic polyadenylation element binding protein 1
RP11-114H7.3	-0.69	1.09	ENSG00000248955	N/A
ZNF17	-0.69	1.09	ENSG00000186272	zinc finger protein 17
ZNF585B	-0.69	2.19	ENSG00000245680	zinc finger protein 585B
IGF2BP3	-0.69	3.09	ENSG00000136231	insulin like growth factor 2 mRNA binding protein 3
ZNF222	-0.69	1.86	ENSG00000159885	zinc finger protein 222
SLC25A30	-0.69	2.92	ENSG00000174032	solute carrier family 25 member 30
RASA1	-0.69	3.26	ENSG00000145715	RAS p21 protein activator 1
TTLL12	-0.69	7.27	ENSG00000100304	tubulin tyrosine ligase like 12
TCEB3	-0.69	6.46	ENSG00000011007	transcription elongation factor B subunit 3
CDR2L	-0.69	6.72	ENSG00000109089	cerebellar degeneration related protein 2 like
TYMS	-0.69	5.71	ENSG00000176890	thymidylate synthetase
CHMP1B	-0.69	5.26	ENSG00000255112	charged multivesicular body protein 1B
RHOF	-0.69	3.92	ENSG00000139725	ras homolog family member F, filopodia associated
KIAA1522	-0.69	7.03	ENSG00000162522	KIAA1522
ZNRF3	-0.69	5.05	ENSG00000183579	zinc and ring finger 3
PPM1D	-0.69	2.94	ENSG00000170836	protein phosphatase, Mg2+/Mn2+ dependent 1D

MRPS26	-0.69	6.29	ENSG00000125901	mitochondrial ribosomal protein S26
BCDIN3D	-0.69	2.07	ENSG00000186666	BCDIN3 domain containing RNA methyltransferase
EXO5	-0.69	2.07	ENSG00000164002	exonuclease 5
RNPC3	-0.69	2.07	ENSG00000185946	RNA binding region (RNP1, RRM) containing 3
MIS18A	-0.69	4.27	ENSG00000159055	MIS18 kinetochore protein A
MYCBP2	-0.69	3.84	ENSG00000005810	MYC binding protein 2, E3 ubiquitin protein ligase
EXOSC6	-0.69	3.79	ENSG00000223496	exosome component 6
MDC1	-0.69	6.67	ENSG00000228575	mediator of DNA damage checkpoint 1
ZNF236	-0.69	2.39	ENSG00000130856	zinc finger protein 236
SNAPC4	-0.69	5.47	ENSG00000165684	small nuclear RNA activating complex polypeptide 4
NLGN4X	-0.69	4.25	ENSG00000146938	neuroligin 4, X-linked
DGKD	-0.70	4.00	ENSG00000280873	diacylglycerol kinase delta
CHAMP1	-0.70	4.63	ENSG00000198824	chromosome alignment maintaining phosphoprotein 1
STAG1	-0.70	3.23	ENSG00000118007	stromal antigen 1
ZNF587B	-0.70	3.52	ENSG00000269343	zinc finger protein 587B
PCNT	-0.70	5.95	ENSG00000160299	pericentrin
TICRR	-0.70	4.91	ENSG00000140534	TOPBP1 interacting checkpoint and replication regulator
HELLS	-0.70	4.19	ENSG00000119969	helicase, lymphoid-specific
CEBPD	-0.70	4.99	ENSG00000221869	CCAAT/enhancer binding protein delta
ZNF296	-0.70	4.82	ENSG00000170684	zinc finger protein 296
RFX5	-0.70	5.49	ENSG00000143390	regulatory factor X5
NUP35	-0.70	4.43	ENSG00000163002	nucleoporin 35
FOXD3	-0.70	1.48	ENSG00000187140	forkhead box D3
PDIA4	-0.70	8.79	ENSG00000155660	protein disulfide isomerase family A member 4
INO80	-0.70	5.34	ENSG00000128908	INO80 complex subunit
HSPA8	-0.70	10.43	ENSG00000109971	heat shock protein family A (Hsp70) member 8

TIMM10B	-0.70	4.26	ENSG00000132286	translocase of inner mitochondrial membrane 10B
YRDC	-0.70	4.55	ENSG00000196449	yrdC N6-threonylcarbamoyltransferase domain containing
PAX9	-0.70	1.75	ENSG00000198807	paired box 9
TTC32	-0.70	1.75	ENSG00000183891	tetratricopeptide repeat domain 32
NEK8	-0.70	2.31	ENSG00000160602	NIMA related kinase 8
MRPL38	-0.70	2.84	ENSG00000204316	mitochondrial ribosomal protein L38
CEBPA	-0.70	6.86	ENSG00000245848	CCAAT/enhancer binding protein alpha
SBDSP1	-0.70	3.58	ENSG00000225648	Shwachman-Bodian-Diamond syndrome pseudogene 1
FBXL19	-0.70	5.90	ENSG00000099364	F-box and leucine rich repeat protein 19
CRIPAK	-0.70	4.12	ENSG00000179979	cysteine rich PAK1 inhibitor
CDC25A	-0.70	5.16	ENSG00000164045	cell division cycle 25A
ZC3H7A	-0.70	3.71	ENSG00000122299	zinc finger CCCH-type containing 7A
MRPL49	-0.70	6.33	ENSG00000149792	mitochondrial ribosomal protein L49
DNAJA3	-0.70	6.89	ENSG00000276726	DnaJ heat shock protein family (Hsp40) member A3
CEBPB	-0.70	7.44	ENSG00000172216	CCAAT/enhancer binding protein beta
ZNF384	-0.70	5.93	ENSG00000126746	zinc finger protein 384
GPATCH4	-0.70	6.44	ENSG00000160818	G-patch domain containing 4
AC006273.5	-0.70	2.01	ENSG00000267530	N/A
PILRB	-0.70	2.01	ENSG00000121716	paired immunoglobin-like type 2 receptor beta
CXorf56	-0.71	3.68	ENSG00000018610	chromosome X open reading frame 56
PHF5A	-0.71	5.49	ENSG00000100410	PHD finger protein 5A
CHEK1	-0.71	5.72	ENSG00000149554	checkpoint kinase 1
ZZEF1	-0.71	5.11	ENSG00000074755	zinc finger ZZ-type and EF-hand domain containing 1
RPL7L1	-0.71	6.04	ENSG00000146223	ribosomal protein L7 like 1
LDB1	-0.71	5.86	ENSG00000198728	LIM domain binding 1
KIF15	-0.71	2.78	ENSG00000280610	kinesin family member 15

RAI1	-0.71	4.31	ENSG00000108557	retinoic acid induced 1
AKAP17A	-0.71	6.33	ENSG00000197976	A-kinase anchoring protein 17A
FBXL6	-0.71	5.30	ENSG00000182325	F-box and leucine rich repeat protein 6
TRUB1	-0.71	3.59	ENSG00000165832	TruB pseudouridine synthase family member 1
TICAM1	-0.71	5.76	ENSG00000127666	toll like receptor adaptor molecule 1
TUBA1C	-0.71	7.02	ENSG00000167553	tubulin alpha 1c
NFRKB	-0.71	5.77	ENSG00000170322	nuclear factor related to kappaB binding protein
AGAP1	-0.71	4.34	ENSG00000157985	ArfGAP with GTPase domain, ankyrin repeat and PH domain 1
RCAN1	-0.71	5.40	ENSG00000159200	regulator of calcineurin 1
AC074117.10	-0.71	3.75	ENSG00000234072	N/A
ATP1B3	-0.71	6.38	ENSG00000069849	ATPase Na+/K+ transporting subunit beta 3
DNA2	-0.71	3.06	ENSG00000138346	DNA replication helicase/nuclease 2
ISG20L2	-0.71	6.14	ENSG00000143319	interferon stimulated exonuclease gene 20 like 2
ORC6	-0.71	3.50	ENSG00000091651	origin recognition complex subunit 6
PGAM5	-0.71	7.07	ENSG00000247077	PGAM family member 5, mitochondrial serine/threonine protein phosphatase
KANSL1	-0.71	4.81	ENSG00000120071	KAT8 regulatory NSL complex subunit 1
DLG5	-0.71	4.35	ENSG00000151208	discs large MAGUK scaffold protein 5
SGTB	-0.72	2.99	ENSG00000197860	small glutamine rich tetratricopeptide repeat containing beta
RRP12	-0.72	7.23	ENSG00000052749	ribosomal RNA processing 12 homolog
RAP2B	-0.72	4.68	ENSG00000181467	RAP2B, member of RAS oncogene family
NR1I3	-0.72	1.39	ENSG00000143257	nuclear receptor subfamily 1 group I member 3
GATAD2A	-0.72	6.77	ENSG00000167491	GATA zinc finger domain containing 2A

C1orf131	-0.72	3.94	ENSG00000143633	chromosome 1 open reading frame 131
GINS3	-0.72	4.16	ENSG00000181938	GINS complex subunit 3
ABCE1	-0.72	5.87	ENSG00000164163	ATP binding cassette subfamily E member 1
HIC2	-0.72	5.09	ENSG00000169635	HIC ZBTB transcriptional repressor 2
TAF2	-0.72	4.22	ENSG00000064313	TATA-box binding protein associated factor 2
DDX20	-0.72	2.92	ENSG00000064703	DEAD-box helicase 20
PEG10	-0.72	7.88	ENSG00000242265	paternally expressed 10
POLR1A	-0.72	7.06	ENSG00000068654	RNA polymerase I subunit A
SHMT2	-0.72	7.85	ENSG00000182199	serine hydroxymethyltransferase 2
POGK	-0.72	6.05	ENSG00000143157	pogo transposable element with KRAB domain
CDK12	-0.72	5.70	ENSG00000167258	cyclin dependent kinase 12
SLC52A3	-0.72	1.99	ENSG00000101276	solute carrier family 52 member 3
DHX38	-0.72	5.73	ENSG00000140829	DEAH-box helicase 38
SLX4	-0.72	4.21	ENSG00000188827	SLX4 structure-specific endonuclease subunit
C2orf48	-0.72	1.45	ENSG00000163009	chromosome 2 open reading frame 48
MTPAP	-0.72	4.40	ENSG00000107951	mitochondrial poly(A) polymerase
MPP6	-0.72	3.96	ENSG00000105926	membrane palmitoylated protein 6
JUNB	-0.72	6.47	ENSG00000171223	JunB proto-oncogene, AP-1 transcription factor subunit
E2F8	-0.72	2.58	ENSG00000129173	E2F transcription factor 8
PRMT5	-0.73	6.03	ENSG00000100462	protein arginine methyltransferase 5
ACAT2	-0.73	6.17	ENSG00000120437	acetyl-CoA acetyltransferase 2
RAPGEF6	-0.73	1.81	ENSG00000158987	Rap guanine nucleotide exchange factor 6
ZNF674-AS1	-0.73	2.06	ENSG00000230844	ZNF674 antisense RNA 1 (head to head)
ZNF354A	-0.73	2.27	ENSG00000169131	zinc finger protein 354A
RBM14	-0.73	7.24	ENSG00000239306	RNA binding motif protein 14
MUS81	-0.73	5.97	ENSG00000172732	MUS81 structure-specific endonuclease subunit

GAN	-0.73	3.44	ENSG00000261609	gigaxonin
ZXDB	-0.73	2.80	ENSG00000198455	zinc finger, X-linked, duplicated B
USP22	-0.73	6.46	ENSG00000124422	ubiquitin specific peptidase 22
PSMA6	-0.73	3.07	ENSG00000100902	proteasome subunit alpha 6
RP11-566K19.6	-0.73	1.17	ENSG00000274253	N/A
DNAJC14	-0.73	3.30	ENSG00000135392	DnaJ heat shock protein family (Hsp40) member C14
RNF4	-0.73	6.55	ENSG00000063978	ring finger protein 4
KIFC1	-0.73	6.24	ENSG00000237649	kinesin family member C1
HPD	-0.73	4.82	ENSG00000158104	4-hydroxyphenylpyruvate dioxygenase
WDR81	-0.73	5.80	ENSG00000167716	WD repeat domain 81
ZC3H18	-0.73	6.43	ENSG00000158545	zinc finger CCCH-type containing 18
CNNM4	-0.73	4.32	ENSG00000158158	cyclin and CBS domain divalent metal cation transport mediator 4
TMEM5	-0.73	4.38	ENSG00000118600	transmembrane protein 5
WNT3	-0.73	4.12	ENSG00000277626	Wnt family member 3
KCTD10	-0.74	5.50	ENSG00000110906	potassium channel tetramerization domain containing 10
RP11-1246C19.1	-0.74	2.39	ENSG00000273230	N/A
ZBED6CL	-0.74	3.94	ENSG00000188707	ZBED6 C-terminal like
SLC10A4	-0.74	4.13	ENSG00000145248	solute carrier family 10 member 4
BRSK2	-0.74	5.17	ENSG00000174672	BR serine/threonine kinase 2
NNMT	-0.74	7.33	ENSG00000166741	nicotinamide N-methyltransferase
SEC24D	-0.74	4.73	ENSG00000150961	SEC24 homolog D, COPII coat complex component
NUDT4	-0.74	2.66	ENSG00000173598	nudix hydrolase 4
TRRAP	-0.74	6.12	ENSG00000196367	transformation/transcription domain associated protein
LARP1	-0.74	8.49	ENSG00000155506	La ribonucleoprotein domain family member 1
OTUD3	-0.74	3.36	ENSG00000169914	OTU deubiquitinase 3
FANCM	-0.74	2.28	ENSG00000187790	Fanconi anemia complementation group M
ZBTB2	-0.74	4.05	ENSG00000181472	zinc finger and BTB domain containing 2

MAGEA3	-0.74	6.03	ENSG00000221867	MAGE family member A3
DNAJC2	-0.74	5.66	ENSG00000105821	DnaJ heat shock protein family (Hsp40) member C2
UVSSA	-0.74	3.69	ENSG00000163945	UV stimulated scaffold protein A
FAM200A	-0.75	2.07	ENSG00000221909	family with sequence similarity 200 member A
ZNF124	-0.75	2.07	ENSG00000196418	zinc finger protein 124
DHX9	-0.75	6.83	ENSG00000135829	DExH-box helicase 9
PIP5K1A	-0.75	5.52	ENSG00000143398	phosphatidylinositol-4-phosphate 5-kinase type 1 alpha
PIGO	-0.75	6.37	ENSG00000165282	phosphatidylinositol glycan anchor biosynthesis class O
ZYG11A	-0.75	2.75	ENSG00000203995	zyg-11 family member A, cell cycle regulator
GK5	-0.75	2.11	ENSG00000175066	glycerol kinase 5 (putative)
CEBPZ	-0.75	4.63	ENSG00000115816	CCAAT/enhancer binding protein zeta
TRMT44	-0.75	4.12	ENSG00000155275	tRNA methyltransferase 44 homolog (S. cerevisiae)
NPC1	-0.75	6.21	ENSG00000141458	NPC intracellular cholesterol transporter 1
ARID1B	-0.75	5.01	ENSG00000049618	AT-rich interaction domain 1B
MCM9	-0.75	2.37	ENSG00000111877	minichromosome maintenance 9 homologous recombination repair factor
ACLY	-0.75	7.65	ENSG00000131473	ATP citrate lyase
SNRNP200	-0.75	8.16	ENSG00000144028	small nuclear ribonucleoprotein U5 subunit 200
SALL1	-0.75	4.10	ENSG00000103449	spalt like transcription factor 1
ZNF407	-0.75	2.81	ENSG00000215421	zinc finger protein 407
ESPL1	-0.75	6.48	ENSG00000135476	extra spindle pole bodies like 1, separase
PCBP1-AS1	-0.75	3.72	ENSG00000179818	PCBP1 antisense RNA 1
PDF	-0.75	3.02	ENSG00000258429	peptide deformylase (mitochondrial)
RBM12B	-0.75	2.67	ENSG00000183808	RNA binding motif protein 12B
TGFBRAP1	-0.75	4.91	ENSG00000135966	transforming growth factor beta receptor associated protein 1
GSE1	-0.75	5.50	ENSG00000131149	Gse1 coiled-coil protein

BRPF1	-0.75	4.09	ENSG00000156983	bromodomain and PHD finger containing 1
PDCD7	-0.75	4.10	ENSG00000090470	programmed cell death 7
SF1	-0.76	8.82	ENSG00000168066	splicing factor 1
MGAT5B	-0.76	1.94	ENSG00000167889	mannosyl (alpha-1,6-)-glycoprotein beta-1,6-N-acetyl-glucosaminyltransferase, isozyme B
PIGW	-0.76	2.50	ENSG00000275600	phosphatidylinositol glycan anchor biosynthesis class W
MTBP	-0.76	2.26	ENSG00000172167	MDM2 binding protein
DDX12P	-0.76	2.53	ENSG00000214826	DEAD/H-box helicase 12, pseudogene
PTMAP4	-0.76	1.98	ENSG00000231503	prothymosin, alpha pseudogene 4
KLF15	-0.76	4.71	ENSG00000163884	Kruppel like factor 15
MSL3	-0.76	4.92	ENSG00000005302	male-specific lethal 3 homolog (Drosophila)
DBR1	-0.76	2.55	ENSG00000138231	debranching RNA lariats 1
ZBTB39	-0.76	3.54	ENSG00000166860	zinc finger and BTB domain containing 39
FAM111B	-0.76	4.19	ENSG00000189057	family with sequence similarity 111 member B
PAK1IP1	-0.76	5.67	ENSG00000111845	PAK1 interacting protein 1
ZBTB14	-0.76	3.32	ENSG00000198081	zinc finger and BTB domain containing 14
ZNF777	-0.76	5.03	ENSG00000196453	zinc finger protein 777
CBX4	-0.76	6.49	ENSG00000141582	chromobox 4
UPF3B	-0.76	4.58	ENSG00000125351	UPF3 regulator of nonsense transcripts homolog B (yeast)
C16orf58	-0.76	4.86	ENSG00000140688	chromosome 16 open reading frame 58
SMG8	-0.76	3.83	ENSG00000167447	SMG8, nonsense mediated mRNA decay factor
HEATR1	-0.76	5.92	ENSG00000119285	HEAT repeat containing 1
MYBBP1A	-0.76	7.73	ENSG00000132382	MYB binding protein 1a
LIMS1	-0.76	4.83	ENSG00000169756	LIM zinc finger domain containing 1
WBP11	-0.76	6.87	ENSG00000084463	WW domain binding protein 11
ENTPD7	-0.76	3.75	ENSG00000198018	ectonucleoside triphosphate diphosphohydrolase 7

PPP1R8	-0.76	5.25	ENSG00000117751	protein phosphatase 1 regulatory subunit 8
AURKA	-0.76	7.88	ENSG00000087586	aurora kinase A
CELF1	-0.77	4.30	ENSG00000149187	CUGBP, Elav-like family member 1
NGRN	-0.77	5.14	ENSG00000182768	neugrin, neurite outgrowth associated
HK2	-0.77	2.15	ENSG00000159399	hexokinase 2
NUP153	-0.77	5.75	ENSG00000124789	nucleoporin 153
FAM53B	-0.77	6.49	ENSG00000189319	family with sequence similarity 53 member B
MORN1	-0.77	2.94	ENSG00000116151	MORN repeat containing 1
MCM10	-0.77	5.49	ENSG00000065328	minichromosome maintenance 10 replication initiation factor
RP5-1112D6.8	-0.77	1.79	ENSG00000272356	N/A
VCAM1	-0.77	2.74	ENSG00000162692	vascular cell adhesion molecule 1
NOB1	-0.77	5.31	ENSG00000141101	NIN1/PSMD8 binding protein 1 homolog
NVL	-0.77	3.97	ENSG00000143748	nuclear VCP-like
FUBP1	-0.77	6.23	ENSG00000162613	far upstream element binding protein 1
TCF19	-0.77	5.24	ENSG00000224379	transcription factor 19
C9orf40	-0.77	3.76	ENSG00000135045	chromosome 9 open reading frame 40
CNOT4	-0.77	3.98	ENSG00000080802	CCR4-NOT transcription complex subunit 4
RRP1B	-0.77	6.51	ENSG00000160208	ribosomal RNA processing 1B
RRN3P3	-0.77	1.91	ENSG00000257122	RRN3 homolog, RNA polymerase I transcription factor pseudogene 3
SIK2	-0.77	7.20	ENSG00000170145	salt inducible kinase 2
RDH16	-0.77	2.33	ENSG00000139547	retinol dehydrogenase 16 (all-trans)
PSPC1	-0.78	4.36	ENSG00000121390	paraspeckle component 1
OIP5	-0.78	3.31	ENSG00000104147	Opa interacting protein 5
PRIM2	-0.78	2.66	ENSG00000146143	primase (DNA) subunit 2
RP11-1020A11.2	-0.78	1.43	ENSG00000269982	N/A
RAB8A	-0.78	5.58	ENSG00000167461	RAB8A, member RAS oncogene family

RABEP1	-0.78	4.40	ENSG00000029725	rabaptin, RAB GTPase binding effector protein 1
MTFP1	-0.78	2.68	ENSG00000242114	mitochondrial fission process 1
LCMT2	-0.78	3.67	ENSG00000168806	leucine carboxyl methyltransferase 2
GORAB	-0.78	2.96	ENSG00000120370	golgin, RAB6 interacting
MNX1	-0.78	4.16	ENSG00000130675	motor neuron and pancreas homeobox 1
IRF2BPL	-0.78	4.80	ENSG00000119669	interferon regulatory factor 2 binding protein like
POLR1B	-0.78	5.64	ENSG00000125630	RNA polymerase I subunit B
URB2	-0.78	4.31	ENSG00000135763	URB2 ribosome biogenesis 2 homolog (S. cerevisiae)
CDC42EP1	-0.78	9.23	ENSG00000128283	CDC42 effector protein 1
FBXL18	-0.78	3.08	ENSG00000155034	F-box and leucine rich repeat protein 18
TIGD5	-0.78	4.90	ENSG00000179886	tigger transposable element derived 5
NOL11	-0.78	5.34	ENSG00000130935	nucleolar protein 11
UAP1L1	-0.79	3.89	ENSG00000197355	UDP-N-acetylglucosamine pyrophosphorylase 1 like 1
SKA3	-0.79	4.59	ENSG00000165480	spindle and kinetochore associated complex subunit 3
FAM173B	-0.79	2.67	ENSG00000150756	family with sequence similarity 173 member B
LYAR	-0.79	5.75	ENSG00000145220	Ly1 antibody reactive
FANCE	-0.79	4.46	ENSG00000112039	Fanconi anemia complementation group E
NAF1	-0.79	3.84	ENSG00000145414	nuclear assembly factor 1 ribonucleoprotein
POLR3E	-0.79	5.65	ENSG00000058600	RNA polymerase III subunit E
FAM20C	-0.79	5.39	ENSG00000177706	FAM20C, golgi associated secretory pathway kinase
NELFA	-0.79	5.93	ENSG00000185049	negative elongation factor complex member A
ISOC1	-0.79	4.68	ENSG00000066583	isochorismatase domain containing 1
TERT	-0.79	4.12	ENSG00000164362	telomerase reverse transcriptase
EAF1	-0.79	3.63	ENSG00000144597	ELL associated factor 1
TFB2M	-0.80	4.59	ENSG00000162851	transcription factor B2, mitochondrial

ZNF778	-0.80	2.00	ENSG00000170100	zinc finger protein 778
GLDC	-0.80	5.36	ENSG00000178445	glycine decarboxylase
UCHL5	-0.80	5.76	ENSG00000116750	ubiquitin C-terminal hydrolase L5
RNF138	-0.80	3.01	ENSG00000134758	ring finger protein 138
HMOX1	-0.80	8.65	ENSG00000100292	heme oxygenase 1
CEP72	-0.80	3.63	ENSG00000112877	centrosomal protein 72
SFPQ	-0.80	7.76	ENSG00000116560	splicing factor proline and glutamine rich
GORASP1	-0.80	5.50	ENSG00000114745	golgi reassembly stacking protein 1
LINC00680	-0.80	2.35	ENSG00000215190	long intergenic non-protein coding RNA 680
ONECUT1	-0.80	4.09	ENSG00000169856	one cut homeobox 1
KDM3B	-0.80	5.17	ENSG00000120733	lysine demethylase 3B
FOXD2	-0.81	2.38	ENSG00000186564	forkhead box D2
CACNB2	-0.81	4.13	ENSG00000165995	calcium voltage-gated channel auxiliary subunit beta 2
AC093724.2	-0.81	1.60	ENSG00000281848	N/A
ZNF746	-0.81	4.51	ENSG00000181220	zinc finger protein 746
BMP2K	-0.81	1.69	ENSG00000138756	BMP2 inducible kinase
IBA57	-0.81	3.18	ENSG00000181873	IBA57 homolog, iron-sulfur cluster assembly
SCAF4	-0.81	6.33	ENSG00000156304	SR-related CTD associated factor 4
CLDN1	-0.81	9.23	ENSG00000163347	claudin 1
PTPN1	-0.81	5.08	ENSG00000196396	protein tyrosine phosphatase, non-receptor type 1
PFAS	-0.81	5.84	ENSG00000178921	phosphoribosylformylglycinamidine synthase
YBX1	-0.81	8.26	ENSG00000065978	Y-box binding protein 1
NARS2	-0.81	4.32	ENSG00000137513	asparaginyl-tRNA synthetase 2, mitochondrial (putative)
WDR76	-0.81	4.32	ENSG00000092470	WD repeat domain 76
SSTR2	-0.81	2.81	ENSG00000180616	somatostatin receptor 2
RP11-443B20.1	-0.81	3.36	ENSG00000271936	N/A
SETD1A	-0.81	6.30	ENSG00000099381	SET domain containing 1A
HS3ST3B1	-0.82	4.21	ENSG00000125430	heparan sulfate-glucosamine 3-sulfotransferase 3B1
KMT2D	-0.82	6.23	ENSG00000167548	lysine methyltransferase 2D

TTLL11	-0.82	2.15	ENSG00000175764	tubulin tyrosine ligase like 11
RNF169	-0.82	2.21	ENSG00000166439	ring finger protein 169
PID1	-0.82	3.12	ENSG00000153823	phosphotyrosine interaction domain containing 1
SLC35E2B	-0.82	3.71	ENSG00000189339	solute carrier family 35 member E2B
BCOR	-0.82	4.49	ENSG00000183337	BCL6 corepressor
MAZ	-0.82	5.93	ENSG00000103495	MYC associated zinc finger protein
ZNF274	-0.82	4.02	ENSG00000171606	zinc finger protein 274
TONSL	-0.82	6.90	ENSG00000160949	tonsoku-like, DNA repair protein
AKAP1	-0.82	6.30	ENSG00000121057	A-kinase anchoring protein 1
PER3	-0.82	2.59	ENSG00000049246	period circadian clock 3
EIF3A	-0.82	7.25	ENSG00000107581	eukaryotic translation initiation factor 3 subunit A
AFG3L1P	-0.82	3.64	ENSG00000223959	AFG3 like matrix AAA peptidase subunit 1, pseudogene
NSMAF	-0.82	3.64	ENSG00000035681	neutral sphingomyelinase activation associated factor
HIVEP1	-0.82	2.75	ENSG00000095951	human immunodeficiency virus type I enhancer binding protein 1
MAT2A	-0.82	7.28	ENSG00000168906	methionine adenosyltransferase 2A
FAT1	-0.83	6.17	ENSG00000083857	FAT atypical cadherin 1
MROH6	-0.83	3.95	ENSG00000277781	maestro heat like repeat family member 6
EP300	-0.83	4.86	ENSG00000100393	E1A binding protein p300
MAFF	-0.83	5.01	ENSG00000185022	MAF bZIP transcription factor F
MAP3K14	-0.83	5.20	ENSG00000006062	mitogen-activated protein kinase kinase kinase 14
SETD2	-0.83	5.28	ENSG00000181555	SET domain containing 2
CASP9	-0.83	4.86	ENSG00000132906	caspase 9
MTX1P1	-0.83	1.11	ENSG00000263037	metaxin 1 pseudogene 1
SPDL1	-0.83	4.47	ENSG00000040275	spindle apparatus coiled-coil protein 1
RTEL1-TNFRSF6B	-0.83	1.30	ENSG00000026036	RTEL1-TNFRSF6B readthrough (NMD candidate)
EFCAB7	-0.83	1.91	ENSG00000203965	EF-hand calcium binding domain 7
RP11-332H14.2	-0.83	1.99	ENSG00000272994	N/A

MARK2	-0.83	5.39	ENSG00000072518	microtubule affinity regulating kinase 2
MRGPRF	-0.83	2.19	ENSG00000172935	MAS related GPR family member F
NUFIP1	-0.83	2.37	ENSG00000083635	NUFIP1, FMR1 interacting protein 1
MCM8	-0.83	3.64	ENSG00000125885	minichromosome maintenance 8 homologous recombination repair factor
TGIF2	-0.84	5.53	ENSG00000118707	TGFB induced factor homeobox 2
BAG3	-0.84	6.23	ENSG00000151929	BCL2 associated athanogene 3
BRPF3	-0.84	4.77	ENSG00000096070	bromodomain and PHD finger containing 3
BAHD1	-0.84	4.69	ENSG00000140320	bromo adjacent homology domain containing 1
SRCAP	-0.84	6.89	ENSG00000080603	Snf2 related CREBBP activator protein
TOMM40L	-0.84	5.43	ENSG00000158882	translocase of outer mitochondrial membrane 40 like
FDPS	-0.84	6.81	ENSG00000160752	farnesyl diphosphate synthase
LMNB1	-0.84	4.72	ENSG00000113368	lamin B1
APC	-0.84	3.12	ENSG00000134982	APC, WNT signaling pathway regulator
NCOA6	-0.84	5.21	ENSG00000198646	nuclear receptor coactivator 6
FOXE1	-0.84	4.18	ENSG00000178919	forkhead box E1
TUBA1B	-0.84	6.84	ENSG00000123416	tubulin alpha 1b
SRGAP3	-0.84	2.85	ENSG00000196220	SLIT-ROBO Rho GTPase activating protein 3
NR2F2	-0.84	5.50	ENSG00000185551	nuclear receptor subfamily 2 group F member 2
MINA	-0.84	3.86	ENSG00000170854	MYC induced nuclear antigen
TOP1	-0.85	5.10	ENSG00000198900	topoisomerase (DNA) I
ZCCHC8	-0.85	4.59	ENSG00000033030	zinc finger CCHC-type containing 8
FAM222A	-0.85	5.74	ENSG00000139438	family with sequence similarity 222 member A
RAB35	-0.85	6.54	ENSG00000111737	RAB35, member RAS oncogene family
RP11-253E3.3	-0.85	1.89	ENSG00000250899	N/A
GLTSCR1	-0.85	5.19	ENSG00000063169	glioma tumor suppressor candidate region gene 1

HEXIM1	-0.86	6.82	ENSG00000186834	hexamethylene bisacetamide inducible 1
CASP8AP2	-0.86	2.77	ENSG00000118412	caspase 8 associated protein 2
GSG2	-0.86	2.77	ENSG00000177602	germ cell associated 2, haspin
NDOR1	-0.86	5.46	ENSG00000283590	NADPH dependent diflavin oxidoreductase 1
PPID	-0.86	5.20	ENSG00000171497	peptidylprolyl isomerase D
ZNF765	-0.86	1.63	ENSG00000196417	zinc finger protein 765
RP11-64K7.1	-0.86	1.58	ENSG00000240854	N/A
TMPO-AS1	-0.86	3.63	ENSG00000257167	TMPO antisense RNA 1
ZNF786	-0.86	2.57	ENSG00000197362	zinc finger protein 786
FBXL14	-0.86	3.49	ENSG00000171823	F-box and leucine rich repeat protein 14
NR4A3	-0.86	2.36	ENSG00000119508	nuclear receptor subfamily 4 group A member 3
CTD-2589H19.6	-0.86	1.32	ENSG00000271781	N/A
PGAM1	-0.86	4.91	ENSG00000171314	phosphoglycerate mutase 1
MPHOSPH10	-0.86	4.78	ENSG00000124383	M-phase phosphoprotein 10
RP11-1148L6.5	-0.87	1.19	ENSG00000228343	N/A
APCDD1	-0.87	1.13	ENSG00000154856	APC down-regulated 1
XPC	-0.87	4.17	ENSG00000154767	XPC complex subunit, DNA damage recognition and repair factor
QRICH2	-0.87	3.14	ENSG00000129646	glutamine rich 2
PTMA	-0.87	7.43	ENSG00000187514	prothymosin, alpha
SQSTM1	-0.87	10.38	ENSG00000161011	sequestosome 1
MIR22HG	-0.87	3.65	ENSG00000282800	MIR22 host gene
ARID1A	-0.87	5.96	ENSG00000117713	AT-rich interaction domain 1A
HNRNPF	-0.87	6.58	ENSG00000169813	heterogeneous nuclear ribonucleoprotein F
LINC00342	-0.87	1.90	ENSG00000232931	long intergenic non-protein coding RNA 342
ACO2	-0.87	6.20	ENSG00000100412	aconitase 2
RPS6KB1	-0.88	3.86	ENSG00000108443	ribosomal protein S6 kinase B1
F11	-0.88	2.37	ENSG00000088926	coagulation factor XI
UBE2O	-0.88	5.69	ENSG00000175931	ubiquitin conjugating enzyme E2 O
PNN	-0.88	6.17	ENSG00000100941	pinin, desmosome associated protein
GLUD2	-0.88	4.15	ENSG00000182890	glutamate dehydrogenase 2

STX11	-0.88	3.63	ENSG00000135604	syntaxin 11
TFDP1	-0.88	6.31	ENSG00000198176	transcription factor Dp-1
ZNF830	-0.88	3.55	ENSG00000198783	zinc finger protein 830
NSRP1	-0.89	4.00	ENSG00000126653	nuclear speckle splicing regulatory protein 1
SETD1B	-0.89	5.38	ENSG00000139718	SET domain containing 1B
UTP14A	-0.89	6.05	ENSG00000156697	UTP14A small subunit processome component
LARS2	-0.89	4.48	ENSG00000011376	leucyl-tRNA synthetase 2, mitochondrial
NEB	-0.89	4.90	ENSG00000183091	nebulin
ZC3H7B	-0.89	5.42	ENSG00000100403	zinc finger CCCH-type containing 7B
KIAA1524	-0.89	4.17	ENSG00000163507	KIAA1524
LRIG3	-0.89	3.33	ENSG00000139263	leucine rich repeats and immunoglobulin like domains 3
ZNF512B	-0.89	5.65	ENSG00000196700	zinc finger protein 512B
DNAJC9	-0.90	3.07	ENSG00000213551	DnaJ heat shock protein family (Hsp40) member C9
KHSRP	-0.90	8.17	ENSG00000088247	KH-type splicing regulatory protein
POLE3	-0.90	5.99	ENSG00000148229	DNA polymerase epsilon 3, accessory subunit
REEP4	-0.90	6.36	ENSG00000168476	receptor accessory protein 4
GLE1	-0.90	4.57	ENSG00000119392	GLE1, RNA export mediator
BASP1	-0.90	5.49	ENSG00000176788	brain abundant membrane attached signal protein 1
RP11-521B24.3	-0.90	1.75	ENSG00000251602	N/A
IRAK2	-0.91	4.38	ENSG00000134070	interleukin 1 receptor associated kinase 2
DDX21	-0.91	6.24	ENSG00000165732	DExD-box helicase 21
HCFC1	-0.91	7.70	ENSG00000172534	host cell factor C1
PDPK1	-0.91	2.85	ENSG00000140992	3-phosphoinositide dependent protein kinase 1
ODC1	-0.91	10.33	ENSG00000115758	ornithine decarboxylase 1
APOA1	-0.91	8.62	ENSG00000118137	apolipoprotein A1
FOXC1	-0.91	3.59	ENSG00000054598	forkhead box C1

ESRP2	-0.91	6.09	ENSG00000103067	epithelial splicing regulatory protein 2
MED17	-0.91	4.08	ENSG00000042429	mediator complex subunit 17
NOLC1	-0.91	7.60	ENSG00000166197	nucleolar and coiled-body phosphoprotein 1
NSUN2	-0.91	5.82	ENSG00000037474	NOP2/Sun RNA methyltransferase family member 2
CPSF6	-0.91	6.30	ENSG00000111605	cleavage and polyadenylation specific factor 6
RP11-384K6.6	-0.91	1.56	ENSG00000260404	N/A
PHYH	-0.91	6.40	ENSG00000107537	phytanoyl-CoA 2-hydroxylase
GNPNAT1	-0.91	5.52	ENSG00000100522	glucosamine-phosphate N-acetyltransferase 1
KMT2B	-0.91	6.83	ENSG00000272333	lysine methyltransferase 2B
KCTD5	-0.91	4.76	ENSG00000167977	potassium channel tetramerization domain containing 5
ACOX2	-0.91	5.60	ENSG00000168306	acyl-CoA oxidase 2
TOB2	-0.91	4.88	ENSG00000183864	transducer of ERBB2, 2
HDAC4	-0.91	3.20	ENSG00000068024	histone deacetylase 4
MYO5B	-0.92	1.88	ENSG00000167306	myosin VB
HMCN1	-0.92	1.46	ENSG00000143341	hemicentin 1
CDCA8	-0.92	5.31	ENSG00000134690	cell division cycle associated 8
COL5A3	-0.92	4.06	ENSG00000080573	collagen type V alpha 3 chain
CCNJ	-0.92	3.14	ENSG00000107443	cyclin J
MCM2	-0.92	6.37	ENSG00000073111	minichromosome maintenance complex component 2
MAFK	-0.92	6.40	ENSG00000198517	MAF bZIP transcription factor K
C14orf169	-0.92	4.27	ENSG00000170468	chromosome 14 open reading frame 169
TET3	-0.92	3.98	ENSG00000187605	tet methylcytosine dioxygenase 3
MAPK7	-0.92	4.63	ENSG00000166484	mitogen-activated protein kinase 7
POP1	-0.92	4.30	ENSG00000104356	POP1 homolog, ribonuclease P/MRP subunit
SPRY2	-0.93	1.72	ENSG00000136158	sprouty RTK signaling antagonist 2
EEF2	-0.93	11.15	ENSG00000167658	eukaryotic translation elongation factor 2
PRPF4	-0.93	5.68	ENSG00000136875	pre-mRNA processing factor 4

SMCR8	-0.93	1.29	ENSG00000176994	Smith-Magenis syndrome chromosome region, candidate 8
SRRM1	-0.93	5.26	ENSG00000133226	serine and arginine repetitive matrix 1
DIS3L	-0.93	4.68	ENSG00000166938	DIS3 like exosome 3'-5' exoribonuclease
WHAMM	-0.93	4.15	ENSG00000156232	WAS protein homolog associated with actin, golgi membranes and microtubules
SMARCC2	-0.93	6.35	ENSG00000139613	SWI/SNF related, matrix associated, actin dependent regulator of chromatin subfamily c member 2
YOD1	-0.93	3.40	ENSG00000180667	YOD1 deubiquitinase
ZNF319	-0.93	3.65	ENSG00000166188	zinc finger protein 319
ZBTB12	-0.94	2.57	ENSG00000204366	zinc finger and BTB domain containing 12
CDHR3	-0.94	2.16	ENSG00000128536	cadherin related family member 3
RCC1	-0.94	6.60	ENSG00000180198	regulator of chromosome condensation 1
PAXIP1	-0.94	3.72	ENSG00000157212	PAX interacting protein 1
FAM83D	-0.94	5.42	ENSG00000101447	family with sequence similarity 83 member D
LHX2	-0.94	4.79	ENSG00000106689	LIM homeobox 2
EI24P2	-0.94	1.53	ENSG00000236257	EI24, autophagy associated transmembrane protein pseudogene 2
RP11-47A8.5	-0.94	2.09	ENSG00000272933	N/A
LONRF3	-0.94	3.79	ENSG00000175556	LON peptidase N-terminal domain and ring finger 3
ALPL	-0.94	2.06	ENSG00000162551	alkaline phosphatase, liver/bone/kidney
DUSP7	-0.94	4.25	ENSG00000164086	dual specificity phosphatase 7
ZNF276	-0.95	3.74	ENSG00000158805	zinc finger protein 276
FBXL19-AS1	-0.95	1.04	ENSG00000260852	FBXL19 antisense RNA 1 (head to head)
DBH-AS1	-0.95	2.85	ENSG00000225756	DBH antisense RNA 1
UTP20	-0.95	5.09	ENSG00000120800	UTP20, small subunit processome component
HLF	-0.95	4.88	ENSG00000108924	HLF, PAR bZIP transcription factor

SPHK2	-0.95	5.16	ENSG00000063176	sphingosine kinase 2
FOXA1	-0.95	4.26	ENSG00000129514	forkhead box A1
TGDS	-0.95	2.94	ENSG00000088451	TDP-glucose 4,6-dehydratase
FAM13B	-0.95	2.81	ENSG00000031003	family with sequence similarity 13 member B
IFRD2	-0.95	6.50	ENSG00000214706	interferon related developmental regulator 2
B3GNT9	-0.95	2.13	ENSG00000237172	UDP-GlcNAc:betaGal beta-1,3-N-acetylglucosaminyltransferase 9
CCSAP	-0.95	3.70	ENSG00000154429	centriole, cilia and spindle associated protein
GPS2	-0.96	3.11	ENSG00000132522	G protein pathway suppressor 2
ICAM1	-0.96	7.58	ENSG00000090339	intercellular adhesion molecule 1
KLF2	-0.96	2.98	ENSG00000127528	Kruppel like factor 2
STK35	-0.96	5.02	ENSG00000125834	serine/threonine kinase 35
SLC7A11	-0.96	4.35	ENSG00000151012	solute carrier family 7 member 11
DFFB	-0.96	2.45	ENSG00000169598	DNA fragmentation factor subunit beta
PRMT3	-0.96	3.89	ENSG00000185238	protein arginine methyltransferase 3
DNMT3B	-0.96	5.15	ENSG00000088305	DNA methyltransferase 3 beta
PCF11	-0.96	3.56	ENSG00000165494	PCF11 cleavage and polyadenylation factor subunit
FASTKD5	-0.96	4.47	ENSG00000215251	FAST kinase domains 5
EIF3C	-0.96	1.25	ENSG00000184110	eukaryotic translation initiation factor 3 subunit C
CLSPN	-0.96	3.30	ENSG00000092853	claspin
OSGIN1	-0.97	7.33	ENSG00000140961	oxidative stress induced growth inhibitor 1
RRS1	-0.97	6.58	ENSG00000179041	ribosome biogenesis regulator homolog
MRPS24	-0.97	2.74	ENSG00000062582	mitochondrial ribosomal protein S24
MKRN4P	-0.97	1.50	ENSG00000238222	makorin ring finger protein 4, pseudogene
BSCL2	-0.97	1.70	ENSG00000168000	BSCL2, seipin lipid droplet biogenesis associated
PSMC3IP	-0.97	3.26	ENSG00000131470	PSMC3 interacting protein
RP11-274B21.4	-0.98	1.13	ENSG00000230715	N/A

BRCA2	-0.98	2.54	ENSG00000139618	BRCA2, DNA repair associated
POM121C	-0.98	3.97	ENSG00000272391	POM121 transmembrane nucleoporin C
PDSS1	-0.98	3.91	ENSG00000148459	prenyl (decaprenyl) diphosphate synthase, subunit 1
KLF13	-0.98	6.24	ENSG00000275746	Kruppel like factor 13
DNMT1	-0.98	6.60	ENSG00000130816	DNA methyltransferase 1
DHRS13	-0.98	3.80	ENSG00000167536	dehydrogenase/reductase 13
TOB1	-0.98	5.47	ENSG00000141232	transducer of ERBB2, 1
CREBBP	-0.99	4.97	ENSG00000005339	CREB binding protein
ETF1	-0.99	6.76	ENSG00000120705	eukaryotic translation termination factor 1
DHX37	-0.99	6.97	ENSG00000150990	DEAH-box helicase 37
DCPS	-0.99	7.13	ENSG00000110063	decapping enzyme, scavenger
NCAPH	-0.99	5.87	ENSG00000121152	non-SMC condensin I complex subunit H
TSSK6	-0.99	1.76	ENSG00000178093	testis specific serine kinase 6
EIF4H	-0.99	6.53	ENSG00000106682	eukaryotic translation initiation factor 4H
P2RY11	-0.99	1.93	ENSG00000244165	purinergic receptor P2Y11
DEPDC5	-0.99	3.49	ENSG00000100150	DEP domain containing 5
ORC1	-0.99	4.80	ENSG00000085840	origin recognition complex subunit 1
MEX3D	-0.99	4.41	ENSG00000181588	mex-3 RNA binding family member D
PPIF	-1.00	6.96	ENSG00000108179	peptidylprolyl isomerase F
PLAGL2	-1.00	5.45	ENSG00000126003	PLAG1 like zinc finger 2
ASS1P11	-1.00	2.20	ENSG00000225308	argininosuccinate synthetase 1 pseudogene 11
BCL9L	-1.00	7.02	ENSG00000186174	B-cell CLL/lymphoma 9-like
NOC2L	-1.00	7.20	ENSG00000188976	NOC2 like nucleolar associated transcriptional repressor
ZNF598	-1.01	6.83	ENSG00000167962	zinc finger protein 598
GATA2	-1.01	4.18	ENSG00000179348	GATA binding protein 2
SRFBP1	-1.01	1.98	ENSG00000151304	serum response factor binding protein 1
PLEKHA7	-1.01	3.78	ENSG00000166689	pleckstrin homology domain containing A7

NTS	-1.01	8.20	ENSG00000133636	neurotensin
SARM1	-1.01	3.88	ENSG00000004139	sterile alpha and TIR motif containing 1
NCOA5	-1.01	5.56	ENSG00000124160	nuclear receptor coactivator 5
TSR1	-1.02	5.39	ENSG00000167721	TSR1, ribosome maturation factor
CCDC86	-1.02	6.44	ENSG00000110104	coiled-coil domain containing 86
RP11-517B11.7	-1.02	1.08	ENSG00000261167	N/A
NUDT19	-1.02	4.79	ENSG00000213965	nudix hydrolase 19
TRIM56	-1.02	2.28	ENSG00000169871	tripartite motif containing 56
MORF4L1P1	-1.02	1.29	ENSG00000218283	mortality factor 4 like 1 pseudogene 1
RP11-473I1.9	-1.02	1.29	ENSG00000263244	N/A
ZNF689	-1.02	4.24	ENSG00000156853	zinc finger protein 689
FOXG1	-1.03	2.82	ENSG00000176165	forkhead box G1
MDM1	-1.03	2.55	ENSG00000111554	Mdm1 nuclear protein
FAM21C	-1.03	1.96	ENSG00000172661	family with sequence similarity 21 member C
RCC2	-1.03	5.55	ENSG00000281540	regulator of chromosome condensation 2
SHPK	-1.03	3.65	ENSG00000197417	sedoheptulokinase
SLC4A2	-1.03	8.26	ENSG00000164889	solute carrier family 4 member 2
AMOTL2	-1.03	4.88	ENSG00000114019	angiomotin like 2
NFE2L2	-1.04	7.64	ENSG00000116044	nuclear factor, erythroid 2 like 2
DDC	-1.04	4.30	ENSG00000132437	dopa decarboxylase
HSP90AB3P	-1.04	2.15	ENSG00000183199	heat shock protein 90 alpha family class B member 3, pseudogene
RP11-550F7.1	-1.04	2.26	ENSG00000240809	N/A
ZNF629	-1.04	3.47	ENSG00000102870	zinc finger protein 629
OR2I1P	-1.04	1.37	ENSG00000231973	olfactory receptor family 2 subfamily I member 1 pseudogene
C1orf106	-1.04	4.81	ENSG00000163362	chromosome 1 open reading frame 106
MRI1	-1.05	3.32	ENSG00000037757	methylthioribose-1-phosphate isomerase 1
F2RL2	-1.05	3.06	ENSG00000164220	coagulation factor II thrombin receptor like 2

C20orf24	-1.05	6.24	ENSG00000101084	chromosome 20 open reading frame 24
MYC	-1.05	6.89	ENSG00000136997	v-myc avian myelocytomatosis viral oncogene homolog
AKIRIN1	-1.06	5.81	ENSG00000174574	akirin 1
MICAL3	-1.06	3.39	ENSG00000243156	microtubule associated monooxygenase, calponin and LIM domain containing 3
AARS2	-1.06	4.89	ENSG00000124608	alanyl-tRNA synthetase 2, mitochondrial
PRR12	-1.06	6.18	ENSG00000126464	proline rich 12
LYZ	-1.06	4.56	ENSG00000090382	lysozyme
C17orf51	-1.06	3.44	ENSG00000212719	chromosome 17 open reading frame 51
RP11-251G23.5	-1.06	1.03	ENSG00000272604	N/A
C9orf64	-1.07	3.93	ENSG00000165118	chromosome 9 open reading frame 64
CYTH3	-1.07	3.89	ENSG00000008256	cytohesin 3
HAL	-1.08	6.46	ENSG00000084110	histidine ammonia-lyase
TAF5L	-1.08	4.31	ENSG00000135801	TATA-box binding protein associated factor 5 like
PMM2	-1.08	4.63	ENSG00000140650	phosphomannomutase 2
DOK3	-1.08	2.61	ENSG00000146094	docking protein 3
KLHL21	-1.08	4.68	ENSG00000162413	kelch like family member 21
FAM53C	-1.09	4.88	ENSG00000120709	family with sequence similarity 53 member C
EGR1	-1.09	2.70	ENSG00000120738	early growth response 1
BCL2L2	-1.10	3.60	ENSG00000129473	BCL2 like 2
TNPO3	-1.10	5.28	ENSG00000064419	transportin 3
XXYLT1	-1.10	5.22	ENSG00000173950	xyloside xylosyltransferase 1
SYDE2	-1.11	1.21	ENSG00000097096	synapse defective Rho GTPase homolog 2
PHLDB1	-1.11	5.19	ENSG00000019144	pleckstrin homology like domain family B member 1
MIR600HG	-1.12	2.28	ENSG00000236901	MIR600 host gene
FCF1P2	-1.12	1.15	ENSG00000228638	FCF1 pseudogene 2
CTB-131K11.1	-1.12	2.74	ENSG00000266469	N/A

RNF152	-1.12	3.70	ENSG00000176641	ring finger protein 152
DNAJC11	-1.12	5.30	ENSG00000007923	DnaJ heat shock protein family (Hsp40) member C11
CCDC85C	-1.12	4.54	ENSG00000205476	coiled-coil domain containing 85C
ATF4P4	-1.13	1.89	ENSG00000256167	activating transcription factor 4 pseudogene 4
SF3A1	-1.13	6.94	ENSG00000099995	splicing factor 3a subunit 1
EDC4	-1.13	5.36	ENSG00000038358	enhancer of mRNA decapping 4
ZNF485	-1.13	1.08	ENSG00000198298	zinc finger protein 485
ERF	-1.14	8.01	ENSG00000105722	ETS2 repressor factor
CCDC88C	-1.14	4.00	ENSG00000015133	coiled-coil domain containing 88C
GINS4	-1.14	3.67	ENSG00000147536	GINS complex subunit 4
CA2	-1.15	6.41	ENSG00000104267	carbonic anhydrase 2
SMPD4	-1.16	6.75	ENSG00000136699	sphingomyelin phosphodiesterase 4
C1orf109	-1.16	3.18	ENSG00000116922	chromosome 1 open reading frame 109
S100A9	-1.17	5.18	ENSG00000163220	S100 calcium binding protein A9
SLC30A10	-1.17	1.39	ENSG00000196660	solute carrier family 30 member 10
CHRNB3	-1.17	1.25	ENSG00000147432	cholinergic receptor nicotinic beta 3 subunit
LINC00176	-1.17	1.10	ENSG00000196421	long intergenic non-protein coding RNA 176
DANCR	-1.17	7.88	ENSG00000226950	differentiation antagonizing non-protein coding RNA
RP11-500C11.3	-1.17	2.05	ENSG00000272269	N/A
RP11-613M10.6	-1.17	2.38	ENSG00000234160	N/A
MARS2	-1.18	3.24	ENSG00000247626	methionyl-tRNA synthetase 2, mitochondrial
VAV3	-1.19	1.03	ENSG00000134215	vav guanine nucleotide exchange factor 3
UHRF1	-1.19	3.39	ENSG00000276043	ubiquitin like with PHD and ring finger domains 1
TGM2	-1.20	8.39	ENSG00000198959	transglutaminase 2
ZC3H4	-1.20	5.38	ENSG00000130749	zinc finger CCCH-type containing 4
ENPP2	-1.20	2.23	ENSG00000136960	ectonucleotide pyrophosphatase/phosphodiesterase 2

TGFB3	-1.20	2.33	ENSG00000119699	transforming growth factor beta 3
SAPCD2	-1.20	4.38	ENSG00000186193	suppressor APC domain containing 2
RP11-498C9.15	-1.21	2.04	ENSG00000263731	N/A
PITX3	-1.23	4.46	ENSG00000107859	paired like homeodomain 3
EIF4A1	-1.23	1.67	ENSG00000161960	eukaryotic translation initiation factor 4A1
ZNF230	-1.23	1.67	ENSG00000159882	zinc finger protein 230
EVC	-1.23	3.95	ENSG00000072840	EvC ciliary complex subunit 1
MAML1	-1.24	4.97	ENSG00000161021	mastermind like transcriptional coactivator 1
CDK2AP1	-1.24	4.83	ENSG00000111328	cyclin dependent kinase 2 associated protein 1
CPLX2	-1.26	5.03	ENSG00000145920	complexin 2
RAVER1	-1.26	5.38	ENSG00000161847	ribonucleoprotein, PTB binding 1
C17orf67	-1.26	1.74	ENSG00000214226	chromosome 17 open reading frame 67
KIF18B	-1.26	6.11	ENSG00000186185	kinesin family member 18B
DLGAP1	-1.28	3.22	ENSG00000170579	DLG associated protein 1
SPEN	-1.28	6.22	ENSG00000065526	spen family transcriptional repressor
FOXM1	-1.29	7.20	ENSG00000111206	forkhead box M1
SAA4	-1.29	2.95	ENSG00000148965	serum amyloid A4, constitutive
FABP5	-1.30	1.19	ENSG00000164687	fatty acid binding protein 5
TRIP13	-1.30	5.53	ENSG00000071539	thyroid hormone receptor interactor 13
DLX2	-1.31	2.15	ENSG00000115844	distal-less homeobox 2
PCSK9	-1.33	4.83	ENSG00000169174	proprotein convertase subtilisin/kexin type 9
PER2	-1.33	2.55	ENSG00000132326	period circadian clock 2
BRF2	-1.34	2.28	ENSG00000104221	BRF2, RNA polymerase III transcription initiation factor 50 kDa subunit
GTSE1	-1.35	4.96	ENSG00000075218	G2 and S-phase expressed 1
FES	-1.37	2.15	ENSG00000182511	FES proto-oncogene, tyrosine kinase
MAN2A2	-1.37	6.23	ENSG00000196547	mannosidase alpha class 2A member 2
RP11-449P15.2	-1.39	1.40	ENSG00000273151	N/A

POM121	-1.40	3.66	ENSG00000196313	POM121 transmembrane nucleoporin
ZNF850	-1.42	1.56	ENSG00000267041	zinc finger protein 850
DDN	-1.42	1.62	ENSG00000181418	dendrin
SKP2	-1.42	3.82	ENSG00000145604	S-phase kinase associated protein 2
MLLT1	-1.43	5.15	ENSG00000130382	MLLT1, super elongation complex subunit
TXNDC5	-1.44	3.59	ENSG00000239264	thioredoxin domain containing 5
CHERP	-1.44	5.60	ENSG00000085872	calcium homeostasis endoplasmic reticulum protein
DUSP9	-1.46	7.84	ENSG00000130829	dual specificity phosphatase 9
CHGA	-1.46	1.45	ENSG00000100604	chromogranin A
PDXP	-1.47	1.38	ENSG00000241360	pyridoxal phosphatase
NR4A2	-1.50	5.12	ENSG00000153234	nuclear receptor subfamily 4 group A member 2
HSP90AB2P	-1.50	1.62	ENSG00000205940	heat shock protein 90 alpha family class B member 2, pseudogene
NEIL3	-1.52	3.86	ENSG00000109674	nei like DNA glycosylase 3
TIMP3	-1.52	3.79	ENSG00000100234	TIMP metallopeptidase inhibitor 3
AC141586.5	-1.55	1.01	ENSG00000215154	N/A
RFFL	-1.56	5.28	ENSG00000092871	ring finger and FYVE like domain containing E3 ubiquitin protein ligase
CASZ1	-1.60	1.75	ENSG00000130940	castor zinc finger 1
DLL4	-1.61	3.96	ENSG00000128917	delta like canonical Notch ligand 4
SLC25A25	-1.61	3.96	ENSG00000148339	solute carrier family 25 member 25
NR4A1	-1.65	7.12	ENSG00000123358	nuclear receptor subfamily 4 group A member 1
LRP2	-1.67	5.17	ENSG00000081479	LDL receptor related protein 2
ZNF316	-1.68	2.84	ENSG00000205903	zinc finger protein 316
KBTBD4	-1.69	1.62	ENSG00000123444	kelch repeat and BTB domain containing 4
BHLHA15	-1.70	2.89	ENSG00000180535	basic helix-loop-helix family member a15
CNN2	-1.75	6.78	ENSG00000064666	calponin 2
SRXN1	-1.77	1.26	ENSG00000271303	sulfiredoxin 1
ATF5	-1.78	6.26	ENSG00000169136	activating transcription factor 5

G6PC	-1.85	3.41	ENSG00000131482	glucose-6-phosphatase catalytic subunit
RP11-242D8.1	-1.95	2.77	ENSG00000267002	N/A
SERBP1	-1.98	8.54	ENSG00000142864	SERPINE1 mRNA binding protein 1
DGCR6	-2.00	5.52	ENSG00000183628	DiGeorge syndrome critical region gene 6
AFP	-2.11	5.99	ENSG00000081051	alpha fetoprotein
USP18	-2.46	3.66	ENSG00000184979	ubiquitin specific peptidase 18
IL1RN	-2.58	1.79	ENSG00000136689	interleukin 1 receptor antagonist

Supplementary Table 5

Gene symbol	Gene ID	Gene symbol	Gene ID	Gene symbol	Gene ID
TSPAN33	ENSG00000158457	CALCOCO1	ENSG00000012822	ARHGAP40	ENSG00000124143
BRE	ENSG00000158019	STYXL1	ENSG00000127952	C2CD4C	ENSG00000183186
CERS5	ENSG00000139624	CASC15	ENSG00000272168	RP11-620J15.3	ENSG00000257698
ALPK1	ENSG00000073331	NKD1	ENSG00000140807	P2RX4	ENSG00000135124
CCDC71L	ENSG00000253276	C11orf74	ENSG00000166352	PLEKHB1	ENSG00000021300
MNS1	ENSG00000138587	MALAT1	ENSG00000278217	NBL1	ENSG00000158747
C14orf105	ENSG00000100557	RTN2	ENSG00000125744	QSOX1	ENSG00000116260
PLEKHA4	ENSG00000105559	ITGA7	ENSG00000135424	NXPH4	ENSG00000182379
TMEM45A	ENSG00000181458	SGCB	ENSG00000163069	PXDC1	ENSG00000168994
KDELR3	ENSG00000100196	GPR137B	ENSG00000077585	PGM2L1	ENSG00000165434
PRSS12	ENSG00000164099	RNF103	ENSG00000239305	TMEM182	ENSG00000170417
HLA-L	ENSG00000243753	A4GALT	ENSG00000128274	NEBL	ENSG00000078114
HID1	ENSG00000167861	DSE	ENSG00000111817	REC8	ENSG00000100918
CAMK2N1	ENSG00000162545	RHOBTB1	ENSG00000072422	HERC6	ENSG00000138642
RP11-403I13.8	ENSG00000272419	RP11-421F16.3	ENSG00000247903	C15orf52	ENSG00000188549
CKAP4	ENSG00000136026	ZFHX2	ENSG00000136367	STK39	ENSG00000198648
C1R	ENSG00000159403	COL7A1	ENSG00000114270	CYP27A1	ENSG00000135929
LRRC27	ENSG00000148814	SRP14-AS1	ENSG00000248508	RP11-977G19.5	ENSG00000258199
PROX1-AS1	ENSG00000272167	FABP1	ENSG00000163586	EFCAB12	ENSG00000172771
CYB5RL	ENSG00000215883	HLA-C	ENSG00000204525	LTB	ENSG00000223448
GS1-358P8.4	ENSG00000260822	GRN	ENSG00000030582	GARNL3	ENSG00000136895
TP53I3	ENSG00000115129	VAMP2	ENSG00000220205	SLC27A1	ENSG00000130304
SERPINA1	ENSG00000197249	HLA-A	ENSG00000224320	LGALS3BP	ENSG00000108679
LRRC23	ENSG00000010626	DGKA	ENSG00000065357	ALDH3B1	ENSG00000006534
SLFN5	ENSG00000166750	PTPRH	ENSG00000080031	F10	ENSG00000126218
TRIOBP	ENSG00000100106	HLA-E	ENSG00000225201	KCNH3	ENSG00000135519
CYSTM1	ENSG00000120306	DLX4	ENSG00000108813	UBE2L6	ENSG00000156587
RASSF5	ENSG00000266094	RP11-103J17.2	ENSG00000261761	ITGA3	ENSG00000005884
AGBL3	ENSG00000146856	ZNF93	ENSG00000184635	UGT2B7	ENSG00000171234
EPDR1	ENSG00000086289	TMEM151A	ENSG00000179292	IFITM3	ENSG00000142089
PLOD1	ENSG00000083444	ACSF2	ENSG00000167107	CACNB3	ENSG00000167535
MORN2	ENSG00000188010	SYTL4	ENSG00000102362	REEP2	ENSG00000132563
YJEFN3	ENSG00000250067	HECA	ENSG00000112406	DKK3	ENSG00000050165
GABBR1	ENSG00000237112	NPNT	ENSG00000168743	CAPN5	ENSG00000149260

SEMA6C	ENSG00000143434	RAB4B	ENSG00000167578	CAV2	ENSG00000105971
FUCA1	ENSG00000179163	MR1	ENSG00000153029	C1QTNF1	ENSG00000173918
CD99	ENSG00000002586	S100A11	ENSG00000163191	HLA-F	ENSG00000229698
DYNLT3	ENSG00000165169	SH3RF2	ENSG00000156463	TMSB10P1	ENSG00000228499
ZNF287	ENSG00000141040	FZD2	ENSG00000180340	AC005355.2	ENSG00000251169
PSENEN	ENSG00000205155	PRRG2	ENSG00000126460	SPOCK2	ENSG00000107742
CTSF	ENSG00000174080	RIMKLA	ENSG00000177181	SOCS2	ENSG00000120833
HSPA1A	ENSG00000234475	IGIP	ENSG00000182700	NEURL3	ENSG00000163121
FGFR1	ENSG00000077782	FMO5	ENSG00000131781	LRRC8C	ENSG00000171488
MUC1	ENSG00000185499	MAPRE3	ENSG00000084764	HLA-DMA	ENSG00000243215
SMPDL3B	ENSG00000130768	TSPAN15	ENSG00000099282	GBP2	ENSG00000162645
C11orf49	ENSG00000149179	RTN4RL1	ENSG00000185924	PBXIP1	ENSG00000163346
TTLL7	ENSG00000137941	PCSK1N	ENSG00000102109	HIST1H2BD	ENSG00000158373
BTN3A2	ENSG00000186470	SPA17	ENSG00000064199	SP100	ENSG00000067066
FLRT2	ENSG00000185070	VMAC	ENSG00000187650	DENND6B	ENSG00000205593
PRRT1	ENSG00000229071	ZNF362	ENSG00000160094	CCDC88B	ENSG00000168071
TMEM25	ENSG00000149582	PARM1	ENSG00000169116	PCAT6	ENSG00000228288
CRYL1	ENSG00000165475	ANG	ENSG00000214274	HLA-B	ENSG00000206450
ZFYVE1	ENSG00000165861	TCEAL3	ENSG00000196507	CTD-2545H1.2	ENSG00000262445
NDRG4	ENSG00000103034	NPDC1	ENSG00000107281	VNN3	ENSG00000093134
DHRS12	ENSG00000102796	SLC44A2	ENSG00000129353	DNAH5	ENSG00000039139
ADPRHL1	ENSG00000153531	OSER1-AS1	ENSG00000223891	TCF7L1	ENSG00000152284
CLTB	ENSG00000175416	NDUFA13	ENSG00000186010	CAV1	ENSG00000105974
BLOC1S1	ENSG00000135441	FAXDC2	ENSG00000170271	VIM	ENSG00000026025
FSTL3	ENSG00000070404	C20orf196	ENSG00000171984	TMEM54	ENSG00000121900
SHFM1	ENSG00000127922	HLA-G	ENSG00000235680	TMEM37	ENSG00000171227
TBC1D19	ENSG00000109680	RP11-218M22.1	ENSG00000177406	RASSF2	ENSG00000101265
TMA7	ENSG00000232112	FOXQ1	ENSG00000164379	EMB	ENSG00000170571
ATP9A	ENSG00000054793	FCHSD1	ENSG00000197948	F2R	ENSG00000181104
FAM214B	ENSG00000005238	ATP2B4	ENSG00000058668	PPP1R3C	ENSG00000119938
CCDC28B	ENSG00000160050	CLVS1	ENSG00000177182	RP11-284F21.9	ENSG00000272068
LGALS3	ENSG00000131981	RPL39L	ENSG00000163923	DAPK2	ENSG00000035664
RP11-465N4.4	ENSG00000234678	LIPH	ENSG00000163898	LGALS1	ENSG00000100097
FAM229B	ENSG00000203778	HDX	ENSG00000165259	HPSE	ENSG00000173083
ANXA6	ENSG00000197043	THBS3	ENSG00000169231	SCN1B	ENSG00000105711
SEMA3B	ENSG00000012171	ZSWIM4	ENSG00000132003	CLU	ENSG00000120885
OAS3	ENSG00000111331	PLXNA2	ENSG00000076356	TIMP2	ENSG00000035862

NTAN1	ENSG00000275779	GPX8	ENSG00000164294	CCNG2	ENSG00000138764
FNDC4	ENSG00000115226	SERPING1	ENSG00000149131	IFITM2	ENSG00000185201
CD47	ENSG00000196776	SLC30A3	ENSG00000115194	BMF	ENSG00000104081
PIK3AP1	ENSG00000155629	CYB5R2	ENSG00000166394	LAMB3	ENSG00000196878
NPHP1	ENSG00000144061	STAT1	ENSG00000115415	VNN2	ENSG00000112303
TAGLN	ENSG00000149591	COMMD6	ENSG00000188243	SERPINA5	ENSG00000188488
GINM1	ENSG00000055211	MYL9	ENSG00000101335	TMEM86A	ENSG00000151117
BBS12	ENSG00000181004	ZDHHC1	ENSG00000159714	SERPINE2	ENSG00000135919
ITGA2	ENSG00000164171	FAM174B	ENSG00000185442	C10orf10	ENSG00000165507
GDPD5	ENSG00000158555	APLP1	ENSG00000105290	F2RL1	ENSG00000164251
FAM114A1	ENSG00000197712	TUBA1A	ENSG00000167552	CDKN1A	ENSG00000124762
CD59	ENSG00000085063	PDGFB	ENSG00000100311	PRAP1	ENSG00000165828
PCMTD1	ENSG00000168300	DHRS3	ENSG00000162496	LINC00694	ENSG00000225873
SP140L	ENSG00000185404	AGPAT4	ENSG00000026652	GALNT6	ENSG00000139629
SMOC1	ENSG00000198732	ORAI3	ENSG00000175938	NDRG1	ENSG00000104419
RABL2A	ENSG00000144134	CSF1	ENSG00000184371	PAQR8	ENSG00000170915
SHISA4	ENSG00000198892	SAMD9	ENSG00000205413	ENO2	ENSG00000111674
MIR24-2	ENSG00000267519	MMP19	ENSG00000123342	SLC5A12	ENSG00000148942
WWTR1	ENSG00000018408	NT5E	ENSG00000135318	NFATC4	ENSG00000100968
TESC	ENSG00000088992	SAT1	ENSG00000130066	CEACAM1	ENSG00000079385
DDR1	ENSG00000230456	FBXO2	ENSG00000116661	LINC00511	ENSG00000227036
C4orf19	ENSG00000154274	BNIP3L	ENSG00000104765	CDK18	ENSG00000117266
CAPS	ENSG00000105519	RRAS	ENSG00000126458	PIGZ	ENSG00000119227
KIAA1462	ENSG00000165757	GPR153	ENSG00000158292	FAM131C	ENSG00000185519
MTND1P23	ENSG00000225972	RP11-244H3.1	ENSG00000241014	CFB	ENSG00000243649
CDON	ENSG00000064309	MTMR11	ENSG00000014914	CD74	ENSG00000019582
TJP3	ENSG00000105289	GOLM1	ENSG00000135052	MOXD1	ENSG00000079931
ZSWIM5	ENSG00000162415	APBB1	ENSG00000166313	TIMP1	ENSG00000102265
RASSF4	ENSG00000107551	RASSF6	ENSG00000169435	CD7	ENSG00000173762
PLA2G4C	ENSG00000105499	MFSD6	ENSG00000151690	CA9	ENSG00000107159
SP110	ENSG00000135899	CPM	ENSG00000135678	EMP3	ENSG00000142227
GPR161	ENSG00000143147	SLC6A16	ENSG00000063127	S100A4	ENSG00000196154
MVP	ENSG00000013364	FLVCR2	ENSG00000119686	OLFML2A	ENSG00000185585
JAK1	ENSG00000162434	UACA	ENSG00000137831	TMEM59L	ENSG00000105696
TNFAIP8	ENSG00000145779	TUBB3	ENSG00000258947	QPCT	ENSG00000115828
MORN4	ENSG00000171160	TP53TG1	ENSG00000182165	IFI6	ENSG00000126709

Supplementary Table 6

Biological Process	number of gene	p-value	Gene symbol
GO:0060337~type I interferon signaling pathway	14	8.57E-10	SP100, IFITM2, IFITM3, OAS3, HLA-A, HLA-C, HLA-B, STAT1, HLA-E, HLA-G, HLA-F, JAK1, IFI6, GBP2
GO:0002480~antigen processing and presentation of exogenous peptide antigen via MHC class I, TAP-independent	6	5.32E-07	HLA-A, HLA-C, HLA-B, HLA-E, HLA-G, HLA-F
GO:0060333~interferon-gamma-mediated signaling pathway	11	2.75E-06	SP100, OAS3, HLA-A, JAK1, HLA-C, HLA-B, HLA-E, STAT1, GBP2, HLA-G, HLA-F
GO:0002474~antigen processing and presentation of peptide antigen via MHC class I	7	5.44E-05	HLA-A, HLA-C, MR1, HLA-B, HLA-E, HLA-G, HLA-F
GO:0019882~antigen processing and presentation	7	4.36E-04	HLA-A, HLA-C, MR1, HLA-B, HLA-E, CD74, HLA-G
GO:0002486~antigen processing and presentation of endogenous peptide antigen via MHC class I via ER pathway, TAP-independent	3	1.37E-03	HLA-A, HLA-C, HLA-B
GO:0048661~positive regulation of smooth muscle cell proliferation	7	3.43E-03	HDAC4, PDGFB, SKP2, TGM2, ITGA2, RPS6KB1, STAT1
GO:0030198~extracellular matrix organization	12	3.59E-03	DDR1, CD47, LAMB3, PDGFB, COL7A1, HPSE, SPOCK2, NPNT, ITGA7, OLFML2A, ITGA2, ITGA3
GO:0010951~negative regulation of endopeptidase activity	9	4.71E-03	SERPINE2, COL7A1, SPOCK2, SERPINA5, SERPING1, SERPINA1, PCSK1N, TIMP2, TIMP1
GO:0006469~negative regulation of protein kinase activity	8	5.90E-03	FLRT2, TESC, SOCS2, RTN4RL1, WWTR1, CAMK2N1, CEACAM1, DNAJA3
GO:0042270~protection from natural killer cell mediated cytotoxicity	3	6.57E-03	HLA-A, HLA-B, HLA-E
GO:0002576~platelet degranulation	8	6.92E-03	LGALS3BP, APOA1, PDGFB, CLU, SERPING1, SERPINA1, QSOX1, TIMP1

GO:0008285~negative regulation of cell proliferation	18	7.80E-03	CEBPA, TESC, FBXO2, GABBR1, S100A11, TIMP2, ATF5, DDR1, HDAC4, RASSF5, CDKN1A, SERPINE2, NDRG1, EMP3, UTP20, QSOX1, DNAJA3, F2R
GO:0043410~positive regulation of MAPK cascade	7	8.55E-03	CAV2, FGFR1, PDGFB, CDON, C1QTNF1, TIMP2, F2R
GO:0034340~response to type I interferon	3	9.06E-03	TRIM56, SHMT2, SP100
GO:0006955~immune response	18	1.01E-02	IFITM2, IFITM3, OAS3, HLA-A, HLA-C, C1R, HLA-B, HLA-E, HLA-DMA, HLA-G, CD74, HLA-F, MR1, MAP3K14, LTB, GBP2, IFI6, CD7
GO:0002479~antigen processing and presentation of exogenous peptide antigen via MHC class I, TAP-dependent	6	1.15E-02	HLA-A, HLA-C, HLA-B, HLA-E, HLA-G, HLA-F
GO:0046324~regulation of glucose import	3	1.19E-02	HK2, RPS6KB1, RTN2
GO:0051899~membrane depolarization	4	1.44E-02	P2RX4, CAV1, SCN1B, CACNB3
GO:0007160~cell-matrix adhesion	7	1.46E-02	EPDR1, HPSE, NPNT, ITGA7, ITGA2, ITGA3, THBS3
GO:0035456~response to interferon-beta	3	1.51E-02	IFITM2, IFITM3, STAT1
GO:0046339~diacylglycerol metabolic process	3	1.51E-02	DGKA, PGS1, DGKD
GO:0034341~response to interferon-gamma	4	1.61E-02	SP100, IFITM2, IFITM3, DNAJA3
GO:0014911~positive regulation of smooth muscle cell migration	4	1.61E-02	HDAC4, PDGFB, ITGA2, RPS6KB1
GO:0050900~leukocyte migration	8	1.73E-02	CD47, CAV1, F2RL1, ITGA3, SLC7A5, CEACAM1, CD74, SLC7A11
GO:0007596~blood coagulation	10	1.92E-02	P2RX4, F10, SERPINE2, CD59, SERPINA5, F2RL1, ITGA2, SERPINA1, MAFK, F2R
GO:0090331~negative regulation of platelet aggregation	3	2.24E-02	SERPINE2, C1QTNF1, CEACAM1
GO:0060445~branching involved in salivary gland morphogenesis	3	2.24E-02	FGFR1, TGM2, ESRP2
GO:0006464~cellular protein modification process	7	2.61E-02	QPCT, PLOD1, FBXO2, UBE2L6, TTLL7, RIMKLA, TTLL11
GO:0001916~positive regulation of T cell mediated cytotoxicity	3	3.09E-02	HLA-A, HLA-B, HLA-E

GO:0071318~cellular response to ATP	3	3.09E-02	P2RX4, P2RY11, PDXP
GO:0051604~protein maturation	3	3.56E-02	TESC, TSPAN33, TSPAN15
GO:0009607~response to biotic stimulus	3	3.56E-02	IFITM2, IFITM3, PRRT1
GO:0050919~negative chemotaxis	4	3.63E-02	FLRT2, APOA1, SEMA6C, SEMA3B
GO:0002931~response to ischemia	4	3.91E-02	PPIF, CAV1, HK2, EEF2
GO:0042981~regulation of apoptotic process	10	4.29E-02	RASSF5, TP53I3, RASSF6, LGALS1, SERBP1, BNIP3L, SKP2, NDRG1, DAPK2, STAT1
GO:0006695~cholesterol biosynthetic process	4	4.81E-02	APOA1, FDPS, ACLY, LSS

Supplementary Table 7

Altered pathways	Number of gene	p-value	Gene symbol
hsa05416:Viral myocarditis	9	7.31E-05	CAV1, HLA-A, HLA-C, HLA-B, HLA-E, HLA-DMA, HLA-G, SGCB, HLA-F
hsa05332:Graft-versus-host disease	7	1.38E-04	HLA-A, HLA-C, HLA-B, HLA-E, HLA-DMA, HLA-G, HLA-F
hsa05166:HTLV-I infection	18	1.98E-04	ADCY3, PDGFB, HLA-A, FDPS, HLA-C, HLA-B, FZD2, HLA-E, HLA-DMA, HLA-G, HLA-F, CDKN1A, WNT3, JAK1, RRAS, NFATC4, MAP3K14, TERT
hsa05169:Epstein-Barr virus infection	15	2.56E-04	SHFM1, VIM, HLA-A, SKP2, HLA-C, HSPA1A, HLA-B, HLA-E, POLR3E, HLA-G, HLA-F, HDAC4, CDKN1A, JAK1, MAP3K14
hsa05330:Allograft rejection	7	2.67E-04	HLA-A, HLA-C, HLA-B, HLA-E, HLA-DMA, HLA-G, HLA-F
hsa04940:Type I diabetes mellitus	7	5.43E-04	HLA-A, HLA-C, HLA-B, HLA-E, HLA-DMA, HLA-G, HLA-F
hsa04612:Antigen processing and presentation	9	5.58E-04	HLA-A, HLA-C, HSPA1A, HLA-B, HLA-E, HLA-DMA, CD74, HLA-G, HLA-F
hsa04145:Phagosome	12	0.0014	HLA-A, ITGA2, HLA-C, C1R, HLA-B, HLA-E, TUBA1A, HLA-DMA, THBS3, TUBB3, HLA-G, HLA-F
hsa04610:Complement and coagulation cascades	8	0.0015	F10, CFB, CD59, SERPINA5, SERPING1, C1R, SERPINA1, F2R
hsa05320:Autoimmune thyroid disease	7	0.0017	HLA-A, HLA-C, HLA-B, HLA-E, HLA-DMA, HLA-G, HLA-F
hsa05168:Herpes simplex infection	13	0.0020	SP100, OAS3, SKP2, HLA-A, HLA-C, HLA-B, STAT1, HLA-E, HLA-DMA, HLA-G, CD74, HLA-F, JAK1
hsa05203:Viral carcinogenesis	13	0.005	HIST1H2BD, SP100, HLA-A, SKP2, HLA-C, HLA-B, HLA-E, HLA-G, HLA-F, HDAC4, CDKN1A, JAK1, DNAJA3
hsa01130:Biosynthesis of antibiotics	12	0.016	ODC1, SHMT2, FAXDC2, ENO2, FDPS, HK2, ACLY, LSS, UAP1L1, ACAT2, IDH3A, GLDC
hsa04514:Cell adhesion molecules (CAMs)	9	0.025	HLA-A, CLDN1, CD99, HLA-C, HLA-B, HLA-E, HLA-DMA, HLA-G, HLA-F
hsa00520:Amino sugar and nucleotide sugar metabolism	5	0.031	CYB5R2, GNPNAT1, HK2, UAP1L1, CYB5RL

hsa05412:Arrhythmogenic right ventricular cardiomyopathy (ARVC)	6	0.031	ITGA7, ITGA2, CACNB3, ITGA3, TCF7L1, SGCB
hsa05200:Pathways in cancer	17	0.035	CEBPA, ADCY3, FGFR1, PDGFB, SKP2, ITGA2, ITGA3, FZD2, STAT1, DAPK2, TCF7L1, RASSF5, LAMB3, CDKN1A, WNT3, JAK1, F2R

Supplementary Table 8

**Diseases or Functions Annotation**	**p-value**
non-melanoma solid tumor	3.32E-12
cancer	6.61E-12
malignant solid tumor	1.2E-11
tumorigenesis of tissue	1.71E-10
neoplasia of epithelial tissue	2.17E-10
epithelial cancer	3.5E-10
abdominal neoplasm	6.02E-10
abdominal cancer	6.32E-10
cell proliferation of tumor cell lines	3.43E-09
digestive system cancer	1.13E-08
digestive organ tumor	2.28E-08
adenocarcinoma	2.51E-08
invasion of cells	0.000000125
proliferation of cells	0.000000183
cell proliferation of carcinoma cell lines	0.0000002
apoptosis	0.000000202
cell death	0.00000023
apoptosis of tumor cell lines	0.000000252
psoriasis	0.000000314
necrosis	0.000000318
invasion of tumor cell lines	0.000000379
cell movement	0.000000612
lymphocytic neoplasm	0.00000101
cell movement of tumor cell lines	0.0000011
migration of tumor cell lines	0.00000118
gastrointestinal tract cancer	0.00000139
respiratory system tumor	0.00000164
lung tumor	0.00000188
size of lesion	0.00000201
lung cancer	0.00000243
Gastrointestinal Tract Cancer and Tumors	0.0000025
large intestine neoplasm	0.00000291
progression of tumor	0.00000306
morphology of lesion	0.00000339
malignant neoplasm of large intestine	0.00000453

cell death of tumor cell lines	0.0000047
activation of cells	0.00000532
atherosclerosis	0.00000565
advanced malignant tumor	0.00000649
proliferation of lung cancer cell lines	0.00000656
cell death of immune cells	0.00000715
vaso-occlusion	0.0000073
lichen planus	0.00000748
proliferation of liver cells	0.0000077
nonmedullary thyroid cancer	0.0000078
occlusion of blood vessel	0.00000796
lymphocytic cancer	0.00000862
differentiated thyroid cancer	0.00000884
metastasis	0.00000942
cell death of breast cancer cell lines	0.00000986
proliferation of stomach cancer cell lines	0.0000116
gastrointestinal carcinoma	0.0000122
papillary carcinoma	0.0000132
migration of mononuclear leukocytes	0.0000132
leukocyte migration	0.0000138
cell death of blood cells	0.0000139
lymphohematopoietic neoplasia	0.0000175
infection of vascular endothelial cells	0.0000183
neoplasia of prostate cancer cell lines	0.0000197
papillary adenocarcinoma	0.0000203
migration of cells	0.0000204
Lymphoid Cancer and Tumors	0.0000214
synthesis of DNA	0.0000243
outgrowth of neurons	0.0000251
differentiation of cells	0.0000252
outgrowth of cells	0.000027
non-Hodgkin disease	0.0000286
regulation of dendritic cells	0.0000312
experimentally-induced diabetes	0.0000318
migration of monocytes	0.0000318
hematological neoplasia	0.0000355
neoplasia of leukocytes	0.000037

progressive motor neuropathy	0.0000392
growth of tumor	0.0000413
adhesion of blood cells	0.0000417
cytostasis of prostate cancer cell lines	0.0000463
hematologic cancer of cells	0.0000465
proliferation of tumor cells	0.0000469
papillary thyroid cancer	0.0000469
cell movement of myeloid cells	0.0000481
glucose metabolism disorder	0.0000483
arrest in interphase of tumor cells	0.0000485
T-cell lymphoproliferative disorder	0.000049
large intestine carcinoma	0.0000499
HER2 negative hormone receptor negative breast cancer	0.00005
anoikis	0.0000532
breast or colorectal cancer	0.0000549
cell movement of leukocytes	0.000055
outgrowth of neurites	0.0000581
prostatic intraepithelial neoplasia	0.0000613
activation of blood cells	0.0000632
cell proliferation of breast cancer cell lines	0.0000638
Infarction	0.0000644
lymphohematopoietic cancer	0.000065
binding of tumor cell lines	0.0000654
insulin-dependent diabetes mellitus	0.0000656
endothelial cell development	0.0000665
binding of cells	0.0000674
apoptosis of breast cancer cell lines	0.0000684
metastasis of prostate cancer cell lines	0.0000684
fibrosis of liver	0.0000824
inflammation of organ	0.0000833
malignant neoplasm of endocrine gland	0.0000893
systemic autoimmune syndrome	0.0000903
colony formation of tumor cell lines	0.0000914
accumulation of antigen presenting cells	0.0000935
proliferation of pericytes	0.0000952
homing of cells	0.000098
epithelial thyroid cancer	0.0000984

Pathological Cyst	0.0000986
cell death of carcinoma cell lines	0.000102
carcinoma in lung	0.000102
inhibition of lesion	0.000113
thyroid cancer	0.000118
exfoliative glaucoma	0.000119
cell proliferation of hepatoma cell lines	0.00012
cell movement of mononuclear leukocytes	0.00012
hematologic cancer	0.000122
melanoma	0.000129
cell movement of phagocytes	0.000133
cell movement of monocytes	0.000136
cell death of muscle cells	0.000148
genital tract cancer	0.000148
cell death of lymphoma cell lines	0.000157
Fibrosis	0.00016
growth of epithelial tissue	0.000161
cytolysis of tumor cell lines	0.000168
cell movement of endothelial cells	0.000169
development of epithelial tissue	0.000171
synthesis of phosphatidylcholine	0.000174
endocrine gland tumor	0.000176
arrest in interphase of cancer cells	0.00018
tumorigenesis of genital organ	0.000186
female genital tract serous carcinoma	0.00019
migration of lymphatic system cells	0.000191
genital tumor	0.000191
chemotaxis	0.000193
neuroendocrine tumor	0.000196
amyotrophic lateral sclerosis	0.000198
apoptosis of connective tissue cells	0.000207
cell death of lung cancer cell lines	0.000219
large intestine adenocarcinoma	0.000223
head and neck neoplasia	0.000227
Lymphocyte migration	0.00023
incorporation of monounsaturated fatty acids	0.000236
abdominal adenocarcinoma	0.000239

adhesion of immune cells	0.000244
adhesion of epithelial cell lines	0.000258
binding of fatty acid	0.000261
calcification of brain	0.000261
release of alpha granules	0.000261
size of infarct	0.000271
colony formation of cells	0.00028
regulation of blood cells	0.000281
synthesis of lipid	0.000282
thyroid gland tumor	0.000287
pelvic cancer	0.000288
autosomal dominant disease	0.00029
lymphoid cancer	0.000291
cell cycle progression of hematopoietic cell lines	0.000293
T-cell non-Hodgkin disease	0.000297
apoptosis of lymphoma cell lines	0.000312
synthesis of sterol	0.000323
killing of lymphocytes	0.000329
fibrosarcoma	0.000341
synthesis of cholesterol	0.000341
proliferation of endothelial cells	0.000373
growth of embryonic tissue	0.00039
accumulation of putrescine	0.000398
depletion of spermidine	0.000398
differentiation of chronic myelogenous leukemia cells	0.000398
high grade renal clear cell adenocarcinoma	0.000398
regulation of spermidine	0.000398
hepatocellular carcinoma	0.000399
growth of neurites	0.000405
quantity of blood cells	0.000408
proliferation of muscle cell lines	0.00041
cell transformation	0.00041
G2 phase of cancer cells	0.000411
endoreduplication of cells	0.000411
release of oleic acid	0.000411
cell movement of breast cancer cell lines	0.000423
inhibition of tumor	0.000449

abdominal carcinoma	0.000453
proliferation of hepatic stellate cells	0.000463
memory	0.000478
frequency of tumor	0.00048
skin tumor	0.000488
colorectal neoplasia	0.000489
colony formation of carcinoma cell lines	0.00049
infarction of heart	0.000496
accumulation of phagocytes	0.000499
differentiation of muscle cell lines	0.000504
skin lesion	0.000518
incidence of liver tumor	0.000534
metabolism of cholesterol	0.000539
adenoma	0.000565
tauopathy	0.000575
differentiation of connective tissue cells	0.000576
malignant neoplasm of male genital organ	0.00058
synthesis of steroid hormone	0.000582
quantity of leukocytes	0.000586
function of blood cells	0.00059
synthesis of steroid	0.000603
colony formation	0.000604
activation of leukocytes	0.000607
proliferation of urothelial cells	0.000608
colony formation of prostate cancer cell lines	0.000615
prostate cancer	0.000637
metastatic solid tumor	0.000639
tumorigenesis of reproductive tract	0.000643
acute coronary syndrome	0.000646
Prostate Cancer and Tumors	0.000652
necrosis of epithelial tissue	0.000652
quantity of epithelial tissue	0.000662
synthesis of terpenoid	0.000664
female genital neoplasm	0.000669
proliferation of hepatocytes	0.000673
female genital tract cancer	0.000676
formation of muscle	0.000686

T cell migration	0.000691
Viral Infection	0.000696
Dementia	0.000698
density of synapse	0.000703
migration of bladder cancer cell lines	0.000703
re-entry into S phase	0.000703
formation of osteoclast precursor cells	0.000717
amyloidosis	0.000726
intraepithelial neoplasia	0.000731
invasion of carcinoma cell lines	0.00076
male genital neoplasm	0.000764
volume of lesion	0.000777
regulation of leukocytes	0.000777
proliferation of lymphatic system cells	0.000795
small-cell carcinoma	0.000844
formation of caveolae	0.000855
abnormal function of skin	0.000863
urogenital cancer	0.000865
quantity of blood vessel	0.000872
skin cancer	0.000893
cytotoxicity of cytotoxic T cells	0.000906
neoplasia of tumor cell lines	0.000907
breast cancer	0.000916
apoptosis of carcinoma cell lines	0.000929
size of animal	0.000937
incidence of hepatocellular carcinoma	0.000941
neoplasia of cells	0.00095
cell death of lymphocytes	0.000956
cell death of connective tissue cells	0.000968
advanced malignant solid tumor	0.000969
inflammatory response	0.000979
cell proliferation of ovarian cancer cell lines	0.000984
